# Iron Porphyrins and Iron Salens as Highly Enantioselective Catalysts for the Ring-Expansion Reaction of Epoxides to Tetrahydrofurans

**DOI:** 10.1021/acs.orglett.5c00767

**Published:** 2025-03-20

**Authors:** Mehmet Ulutürk, Mehmet Göllü, Tahir Tilki, Erkan Ertürk

**Affiliations:** † TÜBİTAK Marmara Research Center,41470 Gebze, Kocaeli, Türkiye; ‡ Department of Chemistry, Süleyman Demirel University, 32260 Isparta, Türkiye; § Department of Chemistry, Middle East Technical University, 06531 Ankara, Türkiye; ∥ Department of Chemistry, Gebze Technical University, 41400 Gebze, Kocaeli Türkiye

## Abstract

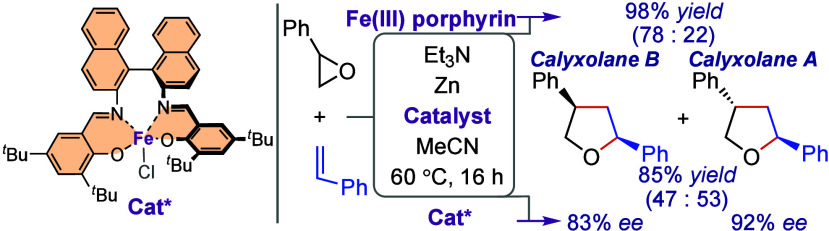

Iron porphyrins have been found to be efficient catalysts for the ring-expansion reaction of epoxides with alkenes to give the corresponding tetrahydrofurans; very low loadings (down to 1 mol %) are sufficient to achieve high yields (up to 98%). Additionally, an iron(III) salen complex based on the axially chiral 1,1-binaphthalene backbone has been shown to catalyze the reaction with high enantioselectivity (*e.g*., 92% *ee* for calyxolane A).

Substituted tetrahydrofuran (THF) rings are frequently encountered in a large number of biologically active compounds and natural products, such as annonaceous acetogenins, lignans, polyether ionophores, macrodiolides etc., which exhibit antitumor, antimicrobial, antimalarial, and antiprotozoal activities.^1−7^ Consequently, considerable efforts have been devoted to developing methods for the stereoselective construction of substituted THFs.^8−11^ Hilt and co-workers recently disclosed the iron-catalyzed ring-expansion reaction of epoxides (**1**) with alkenes (**2**) via the single electron transfer (SET) mechanism to give the corresponding THF compounds **3** and **4** (Scheme 1A).^12−15^ After screening a small library of iron(II) complexes, *e.g.*, iron(II) phosphine, iron(II) bipyridine, and iron(II) *N*-heterocyclic carbene-complexes, they identified the basic iron(II) salen complex [Fe^II^(salen)] as the most effective catalyst among those tested for this reaction.^13,15^ The proposed reaction mechanism includes the following steps: (i) reduction of the *in situ* formed Fe^II^(salen) complex to Fe^I^(salen) by metallic zinc, (ii) reductive ring-opening of the epoxide (**1**) via SET mediated by Fe^I^(salen) to form an iron(II) alkoxide complex possessing a benzylic radical (**5**), (iii) radical coupling of **5** with the alkene **2** to form another radical intermediate (**6**), and (iv) homolytic iron–oxygen bond cleavage initiated by the benzylic radical of **6** onto the oxygen atom, thus affording THFs **3** and **4**.^15^ Subsequently, two research groups developed Lewis acid-catalyzed and 1,1,1,3,3,3-hexafluoro-2-propanol (HFIP) mediated versions of this redox-neutral reaction (Scheme 1B).^16,17^ These reactions were reported to proceed either through direct nucleophilic attack of the alkene onto activated epoxide **7** or through nucleophilic attack of the alkene to ionic intermediate **8**, depending on the substitution pattern of the epoxide ring, thus forming common intermediate **9** for the THF product. Returning to the iron-catalyzed radical process developed by Hilt and colleagues (Scheme 1A), it is evident that despite its notable characteristics the method has several limitations. The amount of Fe^II^(salen) used (20 mol %) is not genuinely catalytic but rather substoichiometric. It is limited to the reactions of conjugated alkenes and epoxides. Additionally, its enantioselective version has yet to be developed. Given the rich redox properties of metal porphyrin complexes^18−20^ and their successful catalytic applications in certain redox reactions,^21−29^ we became intrigued by the possibility of catalyzing radical cyclization between epoxides and alkenes using metal porphyrins. Herein, we report that iron porphyrins exhibit excellent performance in the radical cyclization reaction between epoxides and alkenes (Scheme 1C). Furthermore, we describe a highly enantioselective variant of this transformation by using a bench-stable chiral iron salen-type complex as the catalyst.

**Scheme 1:**
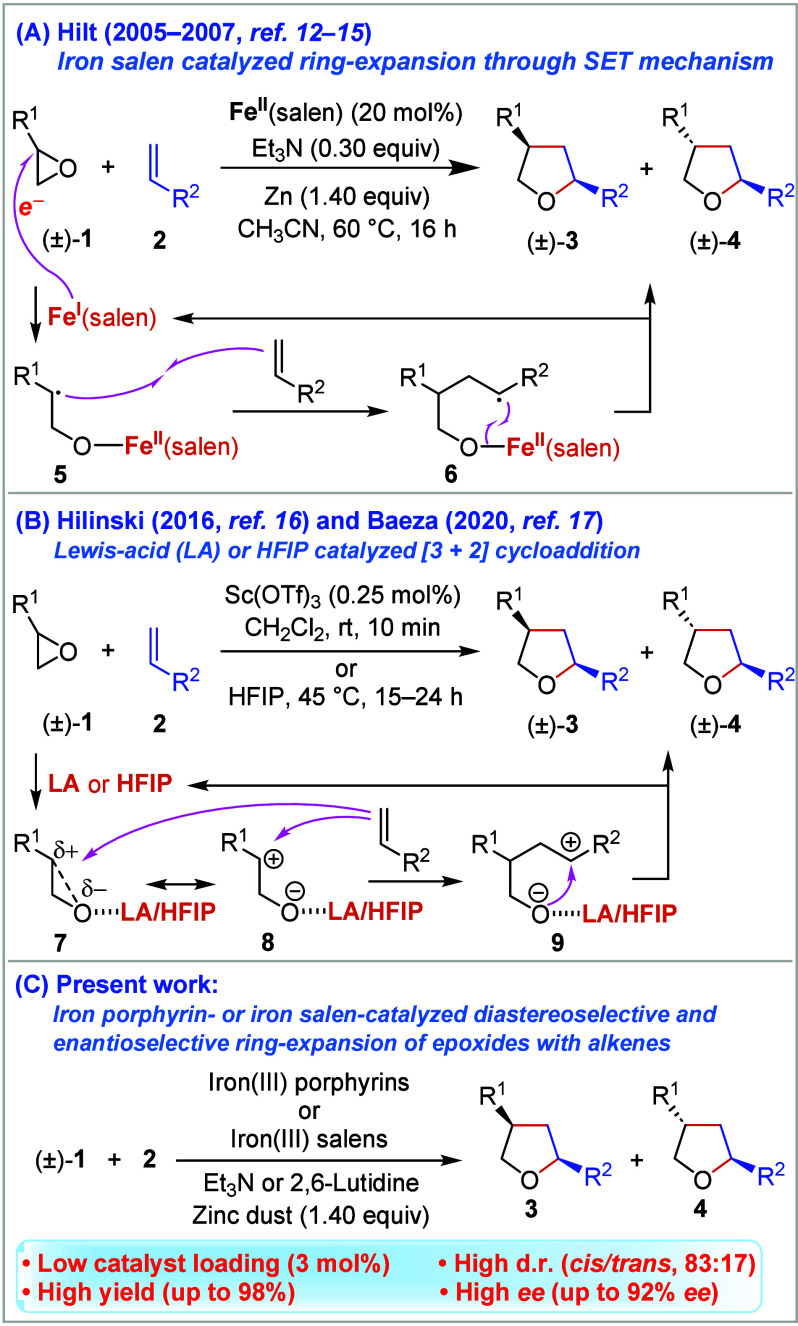
Synthesis of Tetrahydrofurans (THFs) through the Ring-Expansion Reaction of Epoxides with Alkenes

We began our studies by assessing the catalytic potential of selected metal complexes of *meso*-tetraphenylporpyhrin (**10**) for the cyclization of styrene oxide ((±)-**1a**) with styrene (**2a**) using acetonitrile as the solvent, zinc dust as the terminal reducing agent, and triethylamine as an additive at 60 °C for 16 h (Table 1). Details of the optimization studies are provided in the Supporting Information (Tables S1–S5). Under the standard condition, 5 mol % FeTPPCl (**11**) gave *cis*- and *trans*-2,4-diphenyltetrahydrofuran ((±)-**3aa** and (±)-**4aa**, respectively) in 98% yield and 78:22 dr (entry 1). Monitoring of the reaction by TLC or GC revealed that 5 mol % Fe(TPP)Cl already completed the conversion within 4 h (entry 2). Even lower loadings of Fe(TPP)Cl down to 1 mol % enabled the formation of the products in high yields (entries 3–5), demonstrating that the turnover number of Fe(TPP)Cl is roughly 2 orders of magnitude higher than those of iron salens and iron salophenes. Additionally, CoTPP (**12**), Mn(TPP)Cl (**13**), and Cr(TPP)Cl (**14**) were also tested, but these did not lead to any conversion of styrene oxide (entries 6–8). At this point, readily available Fe(III) complexes of some salophene ligands, *i.e.*, **15**–**20**, were taken into consideration due to their extended π-conjugation and hence potentially improved redox behavior.^28,30^ A loading of 5 mol % delivered the ring-expansion products (±)-**3aa** and (±)-**4aa** in similar yields (ranging from 33% to 40%) and dr values, although **16** exhibited slightly higher catalytic activity and diastereoselectivity (entries 9–11). The iron salen complex **22** (5 mol %), generated *in situ* from **21** and FeCl_2_, however, resulted in a significantly lower yield of 18% (entry 12). Additionally, based on the methodology reported by Hilt and colleagues,^15^ 20 mol % of the pre-formed iron(II) salen complex **22** was used, resulting in the formation of (±)-**3aa** and (±)-**4aa** with a total yield of 79% and a *cis/trans* ratio of 70:30 (entry 13). It should be noted that the yield of the products ((±)-**3aa** and (±)-**4aa**) decreases proportionally as the amount of the **22** is decreased (entries 12 and 13). No conversion of styrene oxide was observed when metallic manganese was used instead of zinc (entry 14). The catalytic potential of titanocene dichloride (Cp_2_TiCl_2_) was also examined;^31,32^ however, no conversion of (±)-**1a** was observed. The effect of the coligand, terminal reducing agent, and solvent, as well as temperature and stoichiometry, was also investigated (see Tables S1–S5).

**Table 1:**
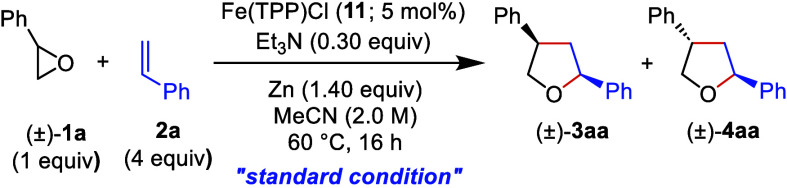
Effect of Reaction Parameters on the Cyclization of Styrene Oxide with Styrene^a^

entry	deviation from the “standard condition”	C (Y) (%)^b^	d.r.^c^
1	none	quant (98)	78:22
2	4 h instead of 16 h	quant (98)	78:22
3	3 mol % **11** instead of 5 mol % **11**, 6 h	quant (98)	78:22
4	2 mol % **11** instead of 5 mol % **11**	95 (85)	78:22
5	1 mol % **11** instead of 5 mol % **11**	90 (75)	78:22
6	**12** instead of **11**	NC^d^	
7	**13** instead of **11**	NC	
8	**14** instead of **11**	NC	
9	**16** instead of **11**	42 (40)	69:31
10	**18** instead of **11**	36 (33)	60:40
11	**20** instead of **11**	35 (33)	67:33
12	**21**·FeCl_2_ instead of **11**	19 (18)	70:30
13	**22** (20 mol %) instead of **11** (5 mol %)	ND^e^ (79)	70:30
14	Mn instead of Zn	NC	

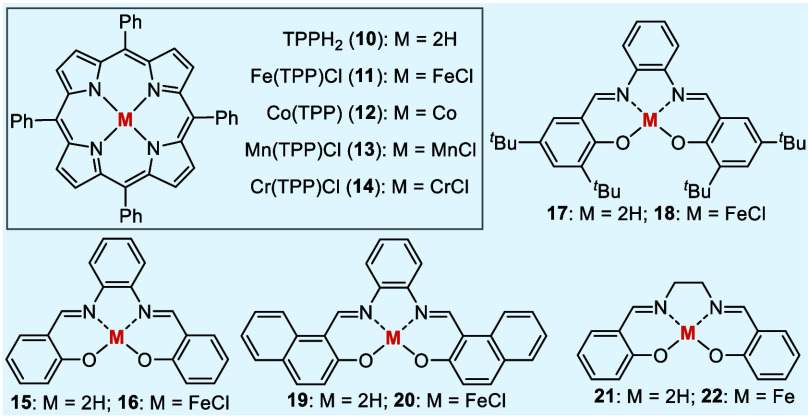

a Reactions were carried out under nitrogen atmosphere using 1.0 mmol racemic styrene oxide ((±)-**1a**).

b C: Conversion, determined by GC analysis using diphenyl ether as internal standard. Y: Isolated yield.

c d.r.: Diastereomeric ratio, determined by ^1^H NMR spectroscopy.

d NC: No conversion.

e ND: Not determined.

Next, the scope of the iron porphyrin-catalyzed intermolecular ring-expansion of epoxides with alkenes was examined for both substrates by employing 3 mol % Fe(TPP)Cl (Table 2). The reaction of (±)-**1a** with styrene derivatives carrying electronically different substituents at the *para*-position (**2a–f**) gave the corresponding 2,4-disubstituted THFs in high yields (Table 2, entries 1–6). Styrene oxide ((±)-**1a**) could also be reacted with α-methylstyrene (**2g**), 1,1-diphenylethylene (**2h**), and indene (**2i**) to produce the corresponding THFs in high yields (entries 7–9). Reaction of (±)-*trans*-stilbene oxide ((±)-**1b**), (±)-*trans*-β-methylstyrene oxide ((±)-**1c**), and *trans*-chalcone (**2j**) with styrene (**2a**) provided the corresponding 2,3,5-trisubstituted THFs in high yields (entries 10–12). The relative configurations of three-substituted THFs were elucidated from nuclear Overhauser effect (NOE), ^3^*J*_H–H_ coupling constants, and chemical shift values (see SI).^15,33−35^ When enantiopure (*R*)-styrene oxide ((*R*)-**1a**) was reacted with styrene (**2a**) via iron *meso*-tetrapheynlporphyrin catalysis, **3aa** and **4aa** (calyxolane B and calyxolane A, respectively, isolated from the Caribbean marine sponge *Calyx podatypa*)^36^ were obtained in their racemic forms (entry 13). This result not only indicated that the reaction proceeds through the formation of a stable intermediate that is susceptible to racemization but also raised a question whether its enantioselective version would be possible via a dynamic kinetic asymmetric transformation (DyKAT)^37^ if chiral Fe(III) porphyrins were used as catalysts. As the representative nonconjugated epoxides, (±)-1-octene oxide, cyclohexene oxide, and methylene cyclohexane oxide were all treated with styrene (**2a**) using 5–10 mol % FeTPPCl. However, no conversions of the respective epoxides were observed. Similarly, the treatment of styrene oxide ((±)-**1a**) with 1-octene did not lead to any conversion of styrene oxide.

**Table 2:**
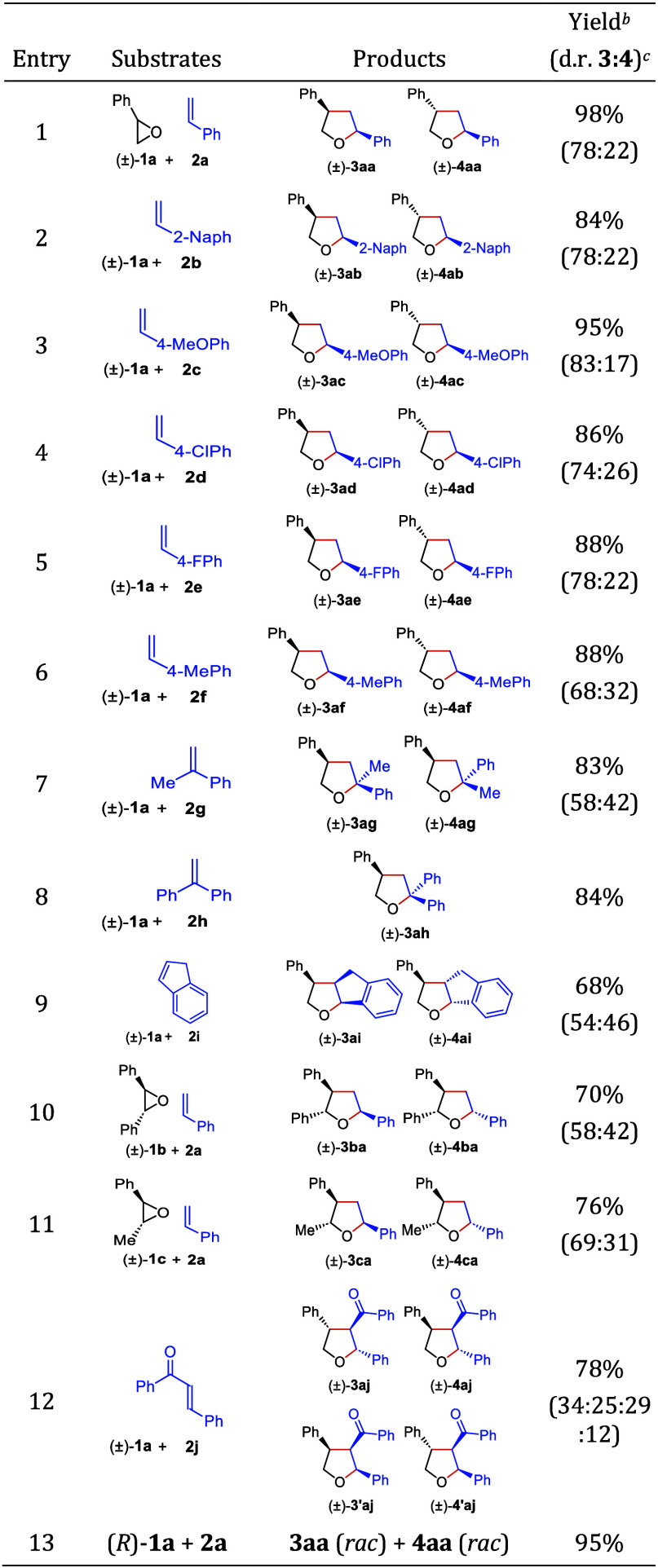
Scope of the Iron Porphyrin-Catalyzed Ring-Expansion Reaction of Epoxides with Alkenes^a^

a **1** (1.0 mmol), **2** (4.0 mmol), Fe(TPP)Cl (3 mol %), Et_3_N (0.3 mmol), Zn (1.4 mmol), MeCN (0.5 mL), 60 °C, 16 h, under N_2_.

b Isolated yield.

c Determined by ^1^H NMR spectroscopy.

Some well-known chiral porphyrin and salen ligands (**23**–**32**) were evaluated in the ring-expansion reaction of epoxides with alkenes (Table 3). Among the chiral Fe(III) porphyrin complexes (**24**, **26**, **28**) used in the reaction between styrene oxide ((±)-**1a**) and styrene (**2a**), the Halterman Fe(III) porphyrin-complex (**26**)^38^ proved to be the best in terms of enantioselectivity, producing the THFs **3aa** and **4aa** in 74% *ee* and 72% *ee*, respectively, while the Gross Fe(III) porphyrin complex (**24**)^39,40^ and the Zhang Fe(III) porphyrin complex (**28**)^41^ provided significantly lower enantiomeric excesses (entries 1–3). Interestingly, the pre-formed Fe(III) complex (**30**)^42^ of the Jacobsen salen ligand (**29**) did not exhibited any catalytic activity (entry 4). On the contrary, the combination of the Jacobsen ligand (**29**) with FeCl_2_ delivered the products in a modest yield (45%) with low enantiomeric excess (entry 5). Eventually, it turned out that the Fe(III) salen complex (**32**)^43−45^ based on the axially chiral 1,1′-binaphthalene backbone catalyzed the reaction with high activity and excellent enantioselectivity; it gave the products **3aa** and **4aa** in 85% total yield and with a dr of 47:53, furnishing 83% *ee* for **3aa** and 92% *ee* for **4aa** (entry 6). This success achieved with **32** prompted us to examine other styrene derivatives (entries 7–9). 2-Vinylnaphthalene (**2b**), 4-methoxystyrene (**2c**), and 4-chlorostyrene (**2d**) could be reacted with styrene oxide to obtain the expected THF products **3** and **4ab**–**ad** in good yields. Although the *cis/trans* selectivities of these reactions were not noteworthy, the THF products, *trans*-diastereomers in particular, were obtained with very high enantiopurities. 1,1-Diphenylethylene (**2h**) was the final alkene coupled with styrene oxide (entry 10). Although the product (**3ah**) was obtained in good yield (76%), its enantiomeric excess was not satisfactory (12% *ee*). Next, we conducted some experiments not only to obtain clues about the reaction mechanism but also to synthesize **3ah** in higher enantiomeric excesses (Scheme 2). When enantiopure styrene oxide ((*R*)-**1a**, >99% *ee*) was reacted with 1,1-diphenylethylene (**2h**) in the presence of **32**, THF **3ah** was obtained in 78% yield, but with very low enantiomeric excess (12% *ee*, Scheme 2, eq 1). Based on this result, we questioned whether **3ah** would be synthesized in higher enantiomeric excesses if different-type iron complexes were employed. Thus, FeTPPCl and the basic iron salophene **16** were used for the reaction between (*R*)-**1a** and **2h**; however, **3ah** was obtained in 8% *ee* in both cases (eqs 2 and 3). These results indicate that rapid racemization takes place during this reaction. HPLC analyses on a chiral column determined that the reactions in eqs 2 and 3 yielded the same enantiomer in excess. The absolute configuration of **3ah** is expected to be the same as that of (*R*)-styrene oxide. The chiral iron porphyrin **28** was also tested for the reaction between (±)-**1a** and **2h**. However, this yielded **3ah** in its racemic form (eq 4). Next, we subjected (*R*)-**1a** to the standard reaction conditions in the presence of 10 mol % FeTPPCl (eq 5). Interestingly, almost all (*R*)-styrene oxide was recovered in its enantiopure form; no racemization was observed.

**Table 3:**
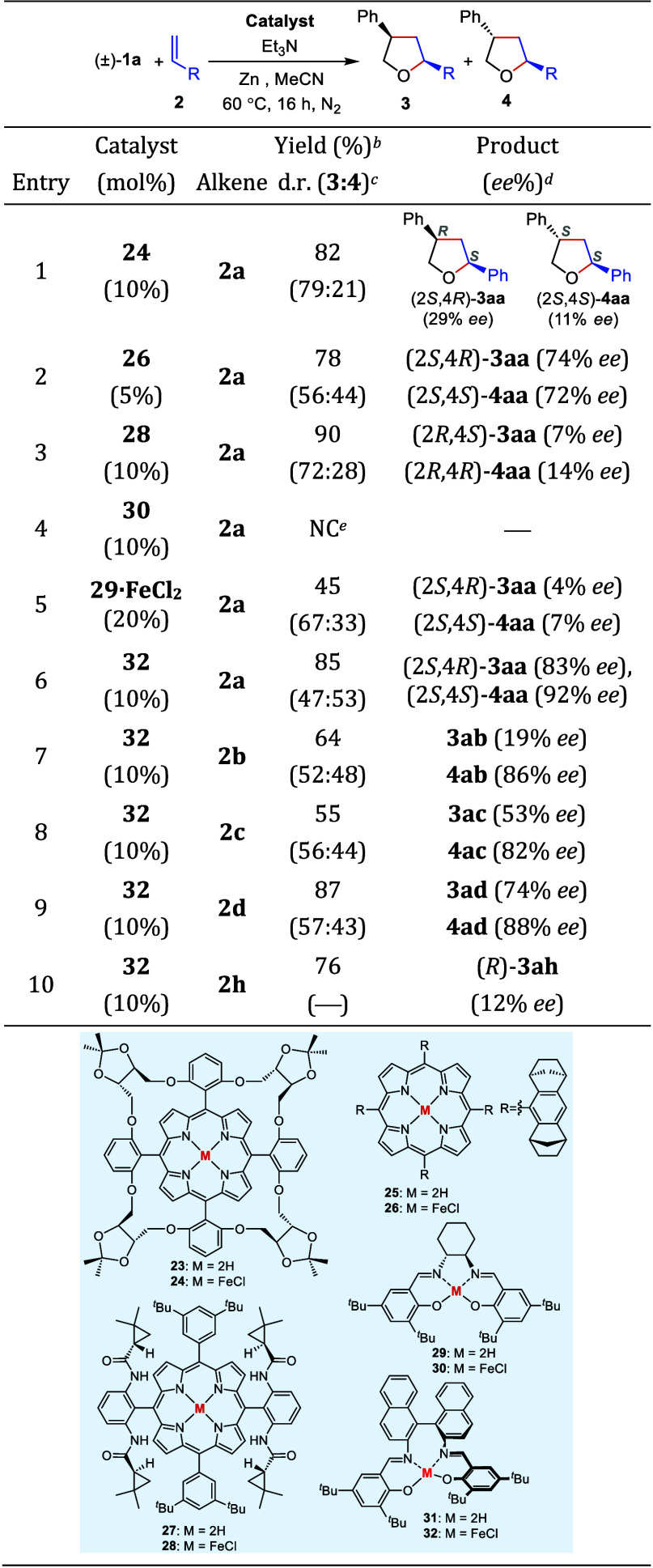
Iron Porphyrin- and Iron Salen-Catalyzed Enantioselective Synthesis of Tetrahydrofurans^a^

a 1 mL of MeCN (1 mL), 1.0 mmol (±)-**1a**, 4.0 mmol styrene (**2a–d**, **2h**), 0.3 mmol Et_3_N, and 1.4 mmol Zn were used.

b Isolated yield.

c d.r.: Determined by ^1^H NMR spectroscopy.

d Enantiomeric excesses (*ee*) were determined by HPLC analysis on a chiral phase.

e NC: No conversion.

**Scheme 2:**
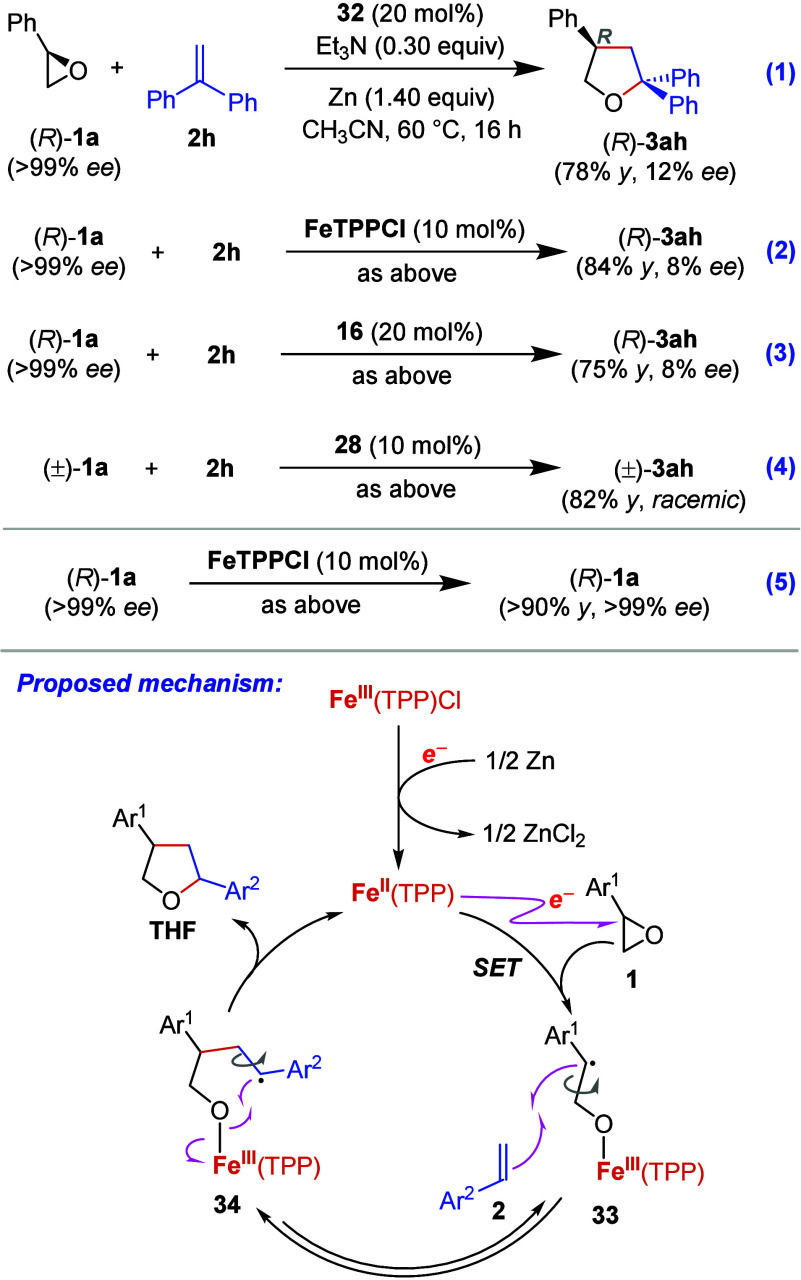
Mechanistic Studies

Experimental results of the present study and previous findings on the mechanism of metal porphyrin-catalyzed oxidation reactions^46,47^ have led us to propose a tentative mechanism for the iron porphyrin-catalyzed ring-expansion reaction of aryl epoxides, as depicted in the lower part of Scheme 2: (i) Fe^III^(TPP)Cl, whose axial coordination site is occupied by a triethylamine or 2,6-lutidine molecule, is reduced to Fe^II^(TPP) by metallic zinc. (ii) The resulting Fe^II^(TPP), likely harboring a triethylamine molecule as a coligand, initiates the ring-expansion reaction by reductively opening the aryl epoxide **1** through SET, thus forming the iron porphyrin-bound benzyl radical intermediate **33**, (iii) which then undergoes coupling with the styrene derivative **2** (radical acceptor) to give another stable iron porphyrin-bound benzyl radical intermediate **34**. According to the stereochemical outcomes of the reactions in Scheme 2 as well as Table 2 entry 13, there should be an equilibrium between **33** and **34** where racemization occurs. (iv) Homolytic cleavage of the iron–oxygen bond and radical ring-closure produce the corresponding THF, thereby making iron(II) porphyrin available for the next catalytic cycle. Interestingly, this mechanism resembles the mode of action of the cytochrome P450 enzymes in the alkene oxidation.^48^ However, unlike **Int1** formed in the P450-catalyzed alkene oxidation, where the iron atom is in its 4+ oxidation sate, formation of the stable iron(III) porphyrin radical intermediate **33** appears to be the key to its radical coupling with styrene derivatives and to obtaining corresponding THF products with excellent selectivity over the epoxide or aldehyde product.

We have shown that readily available and bench-stable iron(III) porphyrins and iron(III) salophens perform excellently in the intermolecular ring-expansion reaction of aryl epoxides with styrenes. In particular, iron porphyrins exhibited excellent catalytic activity, at least 2 orders of magnitude higher than the previously reported iron(II) salens. The chiral Fe(III) salen **32** based on the axially chiral 1,1′-binaphthalene backbone yielded the corresponding THF compounds with very high enantiomeric excesses in certain instances. Iron porphyrin catalysis is believed to take place via the Fe^3+^—Fe^2+^ redox cycle of iron porphyrins. In contrast to the hitherto broad use of iron and other metal porphyrins as biomimetic oxidation catalysts, our findings suggest new potentially valuable applications in reductive radical reactions.

## Data Availability

The data underlying this study are available in the published article and its online Supporting Information.

## Supporting Information Available

Optimization studies in detail, experimental procedures, spectroscopic data (^1^H and ^13^C NMR), and HPLC chromatograms on chiral phase (PDF)

## Dedication

This article is dedicated to the memory of Prof. Dr. Ayhan S. DEMİR (1950–2012).
