# Supplementary material for: Iron Porphyrins and Iron Salens as Highly Enantioselective Catalysts for the Ring-Expansion Reaction of Epoxides to Tetrahydrofurans
Source: Org Lett. 2025 Mar 20;27(12):3083–8. doi: 10.1021/acs.orglett.5c00767 (PMC11959611; doi:10.1021/acs.orglett.5c00767)
Supplement: Supplementary file 1 — ol5c00767_si_001.pdf [file ol5c00767_si_001.pdf]

Iron Porphyrins and Iron Salens as Highly Enantioselective Catalysts for the Ring-Expansion Reaction of Epoxides to Tetrahydrofurans

Mehmet Ulutürk,[†,‡] Mehmet Göllü,[§] Tahir Tilki[‡] and Erkan Ertürk*[,†,∥]

[†] TÜBİTAK Marmara Research Center, 41470 Gebze, Kocaeli, Türkiye

[‡] Department of Chemistry, Süleyman Demirel University, 32260 Isparta, Türkiye

[§] Department of Chemistry, Middle East Technical University, 06531 Ankara, Türkiye

[∥] Department of Chemistry, Gebze Technical University, 41400 Gebze, Kocaeli, Türkiye

E-mail of the corresponding author: erkan.erturk@tubitak.gov.tr

Table of Contents

1. General information.......... 5

2. Optimization studies.......... 5

2.1 General procedure for the optimization studies with Fe(TPP)Cl.......... 5

Table S1. Effect of co-ligand on the iron porphyrin-catalyzed cyclization of styrene oxide ((±)-1a) with styrene ((±)-2a).... 6

Table S2. Effect of styrene amount on the iron porphyrin-catalyzed cyclization of styrene oxide ((±)-1a) with styrene ((±)-2a).......... 6

Table S3. Effect of solvent on the iron porphyrin-catalyzed cyclization of styrene oxide ((±)-1a) with styrene ((±)-2a)....... 7

Table S4. Effect of temperature on the iron porphyrin-catalyzed cyclization of styrene oxide ((±)-1a) with styrene ((±)-2a). 7

Table S5. Effect of terminal reducing agent on the iron porphyrin-catalyzed cyclization of styrene oxide ((±)-1a) with styrene ((±)-2a). 7

3. Procedure for the kinetic studies on the iron porphyrin-catalyzed ring-expansion reaction of styrene oxide ((±)-1a) with styrene (2a).......... 8

Figure S1. Kinetic profile of the iron porphyrin-catalyzed intermolecular ring-expansion reaction of styrene oxide ((±)-1a) with styrene (2a).......... 8

4. Synthetic applications.......... 8

Figure S2. Iron catalysts used in synthetic applications. 8

4.1 *cis*-2,4-Diphenyltetrahydrofuran ((±)-3aa) and *trans*-2,4-diphenyltetrahydrofuran ((±)-4aa) (General Procedure 1) 9

4.2 (2*S*,4*R*)-2,4-Diphenyltetrahydrofuran ((2*S*,4*R*)-3aa, *Calyxolane B*) and (2*S*,4*S*)-2,4-diphenyltetrahydrofuran ((2*S*,4*S*)-4aa, *Calyxolane A*) (General Procedure 2) 10

4.3 *cis*-2-(2-Naphthyl)-4-phenyltetrahydrofuran ((±)-3ab) and *trans*-2-(2-naphthyl)-4-phenyltetrahydrofuran ((±)-4ab) 10

4.4 (2*S*,4*R*)-2-(2-Nahpthyl)-4-phenyltetrahydrofuran ((2*S*,4*R*)-3ab) and (2*S*,4*S*)-2-(2-nahpthyl)-4-phenyltetrahydrofuran ((2*S*,4*S*)-4ab) 11

4.5 *cis*-2-(4-Methoxyphenyl)-4-phenyltetrahydrofuran ((±)-3ac) and *trans*-2-(4-metoxyphenyl)-4-phenyltetrahydrofuran ((±)-4ac) 12

4.6 (2*S*,4*R*)-2-(4-Methoxyphenyl)-4-phenyltetrahydrofuran ((2*S*,4*R)*-3ac) and (2*S*,4*S*)-2-(4-methoxyphenyl)-4-phenyltetrahydrofuran ((2*S*,4*S*)-4ac).......... 12

4.7 *cis*-2-(4-Chlorophenyl)-4-phenyltetrahydrofuran ((±)-3ad) and *trans*-2-(4-chlorophenyl)-4-phenyltetrahydrofuran ((±)-4ad).......... 13

4.8 (2*S*,4*R*)-2-(4-Chlorophenyl)-4-phenyltetrahydrofuran ((2*S*,4*R*)-3ad) and (2*S*,4*S*)-2-(4-chlorophenyl)-4-phenyltetrahydrofuran ((2*S*,4*S*)-4ad).......... 14

4.9 *cis*-2-(4-Fluorophenyl)-4-phenyltetrahydrofuran ((±)-3ae) and *trans*-2-(4-fluorophenyl)-4-phenyltetrahydrofuran ((±)-4ae).......... 15

4.10 *cis*-2-(4-Methylphenyl)-4-phenyltetrahydrofuran ((±)-3af) and *trans*-2-(4-methylphenyl)-4-phenyltetrahydrofuran ((±)-4af).......... 15

4.11 *cis*-2-Methyl-2,4-diphenyltetrahydrofuran ((±)-3ag) and *trans*-2-methyl-2,4-diphenyltetrahydrofuran ((±)-4ag)......... 16

4.12 2,2,4-Triphenyltetrahydrofuran ((±)-3ah) 18

4.13 (4*R*)-2,2,4-Triphenyltetrahydrofuran ((4*R*)-3ah) 18

4.14 *cis*-3-Phenyl-3,3a,4,8b-tetrahydro-2*H*-indeno[1,2-*b*]furan ((±)-3ai) and *trans*-3-phenyl-3,3a,4,8b-tetrahydro-2*H*-indeno[1,2-*b*]furan ((±)-4ai) 19

4.15 (±)-*trans*:*cis*-2,3,5-Triphenyltetrahydrofuran ((±)-3ba) and (±)-*trans*:*trans*-2,3,5-triphenyltetrahydrofuran ((±)-4ba).. 19

4.16 (±)-*trans*:*cis*-2-Methyl-3,5-diphenyltetrahydrofuran ((±)-3ca) and (±)-*trans:trans*-2-methyl-3,5-diphenyltetrahydrofuran ((±)-4ca).......... 21

4.17 (±)-*trans*:*trans*-2,4-Diphenyltetrahydrofuran-3-yl-phenylmethanone ((±)-3aj), (±)-*trans*:*cis*-2,4-Diphenyltetrahydrofuran-3-yl-phenylmethanone ((±)-4aj), (±)-*cis*:*cis*-2,4-Diphenyltetrahydrofuran-3-yl-phenylmethanone ((±)-3'aj) and (±)-*cis*:*trans*-2,4-Diphenyltetrahydrofuran-3-yl-phenylmethanone ((±)-4'aj).......... 23

5. References.......... 26

6. NMR Spectra.......... 27

Figure S3. ^{1}H NMR Spectrum of (±)-3aa and (±)-4aa (500 MHz, $CDCl_3$).......... 27

Figure S4. $^{13}C\{^{1}H\}$ NMR Spectrum of (±)-3aa and (±)-4aa (APT, 125 MHz, $CDCl_3$). 28

Figure S5. ^{1}H NMR Spectrum of (±)-3aa (500 MHz, $CDCl_3$). 29

Figure S6. $^{13}C\{^1H\}$ NMR Spectrum of (±)-3aa (APT, 125 MHz, $CDCl_3$). 30
Figure S7. ^1H NMR Spectrum of (±)-4aa (500 MHz, $CDCl_3$). 31
Figure S8. $^{13}C\{^1H\}$ NMR Spectrum of (±)-4aa (APT, 125 MHz, $CDCl_3$). 32
Figure S9. ^1H NMR Spectrum of (±)-3ab and (±)-4ab (500 MHz, $CDCl_3$). 33
Figure S10. $^{13}C\{^1H\}$ NMR Spectrum of (±)-3ab and (±)-4ab (APT, 125 MHz, $CDCl_3$). 34
Figure S11. ^1H NMR Spectrum of (±)-3ab (500 MHz, $CDCl_3$). 35
Figure S12. $^{13}C\{^1H\}$ NMR Spectrum of (±)-3ab (APT, 125 MHz, $CDCl_3$). 36
Figure S13. ^1H NMR Spectrum of (±)-4ab (500 MHz, $CDCl_3$). 37
Figure S14. $^{13}C\{^1H\}$ NMR Spectrum of (±)-4ab (APT, 125 MHz, $CDCl_3$). 38
Figure S15. ^1H NMR Spectrum of (±)-3ac and (±)-4ac (500 MHz, $CDCl_3$). 39
Figure S16. $^{13}C\{^1H\}$ NMR Spectrum of (±)-3ac and (±)-4ac (APT, 125 MHz, $CDCl_3$). 40
Figure S17. ^1H NMR Spectrum of (±)-3ac (500 MHz, $CDCl_3$). 41
Figure S18. $^{13}C\{^1H\}$ NMR Spectrum of (±)-3ac (APT, 125 MHz, $CDCl_3$). 42
Figure S19. ^1H NMR Spectrum of (±)-4ac (500 MHz, $CDCl_3$). 43
Figure S20. $^{13}C\{^1H\}$ NMR Spectrum of (±)-4ac (APT, 125 MHz, $CDCl_3$). 44
Figure S21. ^1H NMR Spectrum of (±)-3ad and (±)-4ad (500 MHz, $CDCl_3$). 45
Figure S22. $^{13}C\{^1H\}$ NMR Spectrum of (±)-3ad and (±)-4ad (APT, 125 MHz, $CDCl_3$). 46
Figure S23. ^1H NMR Spectrum of (±)-3ad (500 MHz, $CDCl_3$). 47
Figure S24. $^{13}C\{^1H\}$ NMR Spectrum of (±)-3ad (APT, 125 MHz, $CDCl_3$). 48
Figure S25. ^1H NMR Spectrum of (±)-4ad (500 MHz, $CDCl_3$). 49
Figure S26. $^{13}C\{^1H\}$ NMR Spectrum of (±)-3ad (APT, 125 MHz, $CDCl_3$). 50
Figure S27. ^1H NMR Spectrum of (±)-3ae and (±)-4ae (600 MHz, $CDCl_3$). 51
Figure S28. $^{13}C\{^1H\}$ NMR Spectrum of (±)-3ae and (±)-4ae (APT, 150 MHz, $CDCl_3$). 52
Figure S29. ^1H NMR Spectrum of (±)-3af and (±)-4af (600 MHz, $CDCl_3$). 53
Figure S30. $^{13}C\{^1H\}$ NMR Spectrum of (±)-3af and (±)-4af (APT, 150 MHz, $CDCl_3$). 54
Figure S31. ^1H NMR Spectrum of (±)-3ag and (±)-4ag (500 MHz, $CDCl_3$). 55
Figure S32. $^{13}C\{^1H\}$ NMR Spectrum of (±)-3ag and (±)-4ag (APT, 125 MHz, $CDCl_3$). 56
Figure S33. ^1H NMR Spectrum of (±)-3ah (500 MHz, $CDCl_3$). 57
Figure S34. $^{13}C\{^1H\}$ NMR Spectrum of (±)-3ah (APT, 125 MHz, $CDCl_3$). 58
Figure S35. ^1H NMR Spectrum of (±)-3ai and (±)-4ai (500 MHz, $CDCl_3$). 59
Figure S36. $^{13}C\{^1H\}$ NMR Spectrum of (±)-3ai and (±)-4ai (APT, 125 MHz, $CDCl_3$). 60
Figure S37. ^1H NMR Spectrum of (±)-3ba and (±)-4ba (600 MHz, $CDCl_3$). 61
Figure S38. $^{13}C\{^1H\}$ NMR Spectrum of (±)-3ba and (±)-4ba (APT, 150 MHz, $CDCl_3$). 62
Figure S39. COSY NMR Spectrum of (±)-3ba and (±)-4ba ($CDCl_3$). 63
Figure S40. HSQC NMR Spectrum of (±)-3ba and (±)-4ba ($CDCl_3$). 64
Figure S41. NOE-DIFF spectrum of the (±)-3ba and (±)-4ba ($CDCl_3$); increase in H-5, H-4a and H-3 signals after irradiation of the C*H* (H-2) signal at 5.00 ppm. 65
Figure S42. NOE-DIFF spectrum of the (±)-3ba and (±)-4ba ($CDCl_3$); increase in H-2 and H-4a signals after irradiation of the C*H* (H-5) signal at 5.37 ppm. 66
Figure S43. NOE-DIFF spectrum of the (±)-3ba and (±)-4ba ($CDCl_3$); increase in H-3 and H-5 signals after irradiation of the C*H* (H-2) signal at 5.41 ppm. 67
Figure S44. ^1H NMR Spectrum of (±)-3ca and (±)-4ca (600 MHz, $CDCl_3$). 68
Figure S45. $^{13}C\{^1H\}$ NMR Spectrum of (±)-3ca and (±)-4ca (APT, 150 MHz, $CDCl_3$). 69
Figure S46. COSY NMR Spectrum of (±)-3ca and (±)-4ca ($CDCl_3$). 70
Figure S47. HSQC NMR Spectrum of (±)-3ca and (±)-4ca ($CDCl_3$). 71
Figure S48. NOE-DIFF spectrum of the (±)-3ca and (±)-4ca ($CDCl_3$); increase in H-6, H-5, H-4a and H-3 signals after irradiation of the C*H* (H-2) signal at 4.11-4.07 ppm. 72
Figure S49. NOE-DIFF spectrum of the (±)-3ca and (±)-4ca ($CDCl_3$); increase in H-4a, H-4b and H-2 signals after irradiation of the C*H* (H-5) signal at 5.20 ppm. 73

Figure S50. NOE-DIFF spectrum of the (±)-3ca and (±)-4ca ($CDCl_3$); increase in H-6, H-5 and H-3 signals after irradiation of the C*H* (H-2) signal at 4.49-4.44 ppm. 74
Figure S51. NOE-DIFF spectrum of the (±)-3ca and (±)-4ca ($CDCl_3$); increase in H-4a, H-4b, H-3 and H-2 signals after irradiation of the C*H* (H-5) signal at 5.01 ppm. 75
Figure S52. ^{1}H NMR Spectrum of (±)-3aj and (±)-4aj (600 MHz, $CDCl_3$). 76
Figure S53. ^{13}C{^{1}H} NMR Spectrum of (±)-3aj and (±)-4aj (APT, 150 MHz, $CDCl_3$). 77
Figure S54. ^{1}H NMR Spectrum of (±)-3'aj and (±)-4'aj (600 MHz, $CDCl_3$). 78
Figure S55. ^{13}C{^{1}H} NMR Spectrum of (±)-3'aj and (±)-4'aj (APT, 150 MHz, $CDCl_3$). 79
Figure S56. COSY NMR Spectrum of (±)-3aj and (±)-4aj ($CDCl_3$). 80
Figure S57. HSQC NMR Spectrum of (±)-3aj and (±)-4aj ($CDCl_3$). 81
Figure S58. COSY NMR Spectrum of (±)-3'aj and (±)-4'aj ($CDCl_3$). 82
Figure S59. HSQC NMR Spectrum of (±)-3'aj and (±)-4'aj ($CDCl_3$). 83
Figure S60. NOE-DIFF spectrum of the (±)-3aj and (±)-4aj ($CDCl_3$); increase in H-3, H-4 and H-5b signals after irradiation of the C*H* (H-2) signal at 5.35 ppm. 84
Figure S61. NOE-DIFF spectrum of the (±)-3aj and (±)-4aj ($CDCl_3$); increase in H-2 and H-4 signals after irradiation of the C*H* (H-3) signal at 4.08 ppm. 85
Figure S62. NOE-DIFF spectrum of the (±)-3aj and (±)-4aj ($CDCl_3$); increase in H-2, H-3, H-5a and H-5b signals after irradiation of the C*H* (H-4) signal at 3.91 ppm. 86
Figure S63. NOE-DIFF spectrum of the (±)-3aj and (±)-4aj ($CDCl_3$); no increase in signals after irradiation of the C*H* (H-2) signal at 5.81 ppm. 87
Figure S64. NOE-DIFF spectrum of the (±)-3aj and (±)-4aj ($CDCl_3$); increase in H-4 signal after irradiation of the C*H* (H-3) signal at 4.28 ppm. 88
Figure S65. NOE-DIFF spectrum of the (±)-3aj and (±)-4aj ($CDCl_3$); increase in H-3, H-5a and H-5b signals after irradiation of the C*H* (H-4) signal at 4.03 ppm. 89
Figure S66. NOE-DIFF spectrum of the (±)-3'aj and (±)-4'aj ($CDCl_3$); no increase in signals after irradiation of the C*H* (H-2) signal at 5.70 ppm. 90
Figure S67. NOE-DIFF spectrum of the (±)-3'aj and (±)-4'aj ($CDCl_3$); increase in H-2, H-4, H-5a and H-5b signals after irradiation of the C*H* (H-3) signal at 3.93 ppm. 91
Figure S68. NOE-DIFF spectrum of the (±)-3'aj and (±)-4'aj ($CDCl_3$); increase in H-2, H-3, H-5a and H-5b signals after irradiation of the C*H* (H-4) signal at 3.83 ppm. 92
Figure S69. NOE-DIFF spectrum of the (±)-3'aj and (±)-4'aj ($CDCl_3$); no increase in signals after irradiation of the C*H* (H-2) signal at 5.43 ppm. 93
Figure S70. NOE-DIFF spectrum of the (±)-3'aj and (±)-4'aj ($CDCl_3$); increase in H-2 and H-5a signals after irradiation of the C*H* (H-3) signal at 3.80 ppm. 94
Figure S71. NOE-DIFF spectrum of the (±)-3'aj and (±)-4'aj ($CDCl_3$); increase in H-5a signal after irradiation of the C*H* (H-4) signal at 3.73 ppm. 95
7. HPLC Chromatograms 96
Figure S72. HPLC Chromatograms of (±)-3aa and (2*S*,4*R*)-3aa. 96
Figure S73. HPLC Chromatograms of (±)-4aa and (2*S*,4*S*)-4aa. 97
Figure S74. HPLC Chromatograms of (±)-3ab and (2*S*,4*R*)-3ab. 98
Figure S75. HPLC Chromatograms of (±)-4ab and (2*S*,4*S*)-4ab. 99
Figure S76. HPLC Chromatograms of (±)-3ac and (2*S*,4*R*)-3ac. 100
Figure S77. HPLC Chromatograms of (±)-4ac and (2*S*,4*S*)-4ac. 101
Figure S78. HPLC Chromatograms of (±)-3ad and (2*S*,4*R*)-3ad. 102
Figure S79. HPLC Chromatograms of (±)-4ad and (2*S*,4*S*)-4ad. 103
Figure S80. HPLC Chromatograms of (±)-3ah and (4*R*)-3ah. 104
Authorship Contribution Statement 105

1. General information

All air-sensitive reactions were performed under an inert atmosphere of dry nitrogen (N_2) using oven-dried glassware. All reagents and solvents were transferred using gas-tight syringe and cannula techniques under N_2. Reactions were monitored by thin layer chromatography (TLC) on aluminum sheets that were pre-coated with silica gel *SIL G/UV*$_{254}$ from MN GmbH & Co., in which the spots were visualized in UV-light (λ = 254 nm) and/or by staining with phosphomolybdic acid solution in EtOH (10%, w/v). Chromatographic separations were performed using silica gel (MN-silicagel 60, 230-400 mesh). All melting points were determined in open glass capillary tube by means of a BÜCHI Melting Point B-540 apparatus and values are uncorrected. Infrared (FT-IR) spectra were recorded on a PerkinElmer Spectrum One FT-IR spectrometer, $\tilde{\nu}_{max}$ in cm^{-1}. Bands are characterized as broad (br), strong (s), medium (m), and weak (w). ^{1}H and ^{13}C NMR spectra were recorded on a 500 MHz or 600 MHz NMR spectrometer. Chemical shifts (δ) are reported in parts per million (ppm) relative to the residual protons in the NMR solvent ($CHCl_3$: δ 7.26) and carbon resonance of the solvent ($CDCl_3$: δ 77.00). NMR peak multiplicities were given as follows: s = singlet, d = doublet, t = triplet, q = quartet, m = multiplet, br = broad. Structural assignments were made with additional information from gCOSY, gHSQC, and gHMBC experiments. Mass spectra were recorded on a gas chromatography with mass sensitive detector from Agilent Technologies 6890N Network GC System (EI, 70 eV) using Solvent Method (Column: HP-5MSI, 30 m, 0.25 mm ID, 0.25 µm film tickness; Inlet: 280 °C (Split Modus 1:100); Detector: 290 °C; He, 1 mL/min (Constant Flow Modus); Oven: 40 °C (5 min), 5 °C/min, 100 °C (1 min), 10 °C/min, 270 °C (20 min)). Gas Chromatography (GC) analysis were obtained using the Agilent Technologies 7890A GC System (FID) using Mehmet-1 method (Column: HP-5, 30 m, 0.32 mm ID; Inlet: 270 °C (Split Modus 1:50); Detector (FID): 300 °C; He, 1 mL/min (constant flow modus); Oven: 100 °C (10 min), 10 °C/min, 200 °C (10 min), 10 °C/min, 280 °C (5 min)). High resolution electrospray ionization mass spectra (HR-ESI-MS) were obtained with MeOH on a Bruker micrOTOF-Q. The specific rotations ([α]) were measured on a Optical Activity Ltd. AA-65 polarimeter using 5 cm cell with a 0.5 dm path length and the sample concentrations are given in g/100 mL unit. Enantiomeric excesses were determined by high-performance liquid chromatography (HPLC) using chiral columns, CHIRALCEL OD-H, CHIRALPAK AD-H.

Acetonitrile was distilled from CaH_2 under N_2 or purchased from Acros (Acetonitrile 99.9%, ExtraDry, AcroSeal®). Tetrahydrofuran (THF), diethyl ether (Et_2O) and toluene were freshly distilled from sodium/benzophenone prior to use under nitrogen atmosphere. *N,N*-Dimethylformamide (DMF) was distilled under reduced pressure from CaH_2. Dichloromethane (CH_2Cl_2) and ethanol-free chloroform ($CHCl_3$) were dried over CaH_2 and distilled under N_2. Triethylamine was first distilled from CaH_2 under nitrogen atmosphere and stored over freshly activated bead molecular sieves (MS 4Å). 2,6-Lutidine, 4-dimethylaminopyridine (DMAP), and *N*-methylimidazole (NMI) were provided from commercial suppliers and used as received. Styrene and liquid styrene derivatives were filtered through a short column of neutral aluminum oxide before use. Metallic zinc (particle size <10 µm) and manganese (particle size ~325 mesh) dust were purchased and used as received.

Tetraphenylporphyrin ($TPPH_2$, **10**)[1], iron(III) tetraphenylporphyrin chloride (Fe(TPP)Cl, **11**)[2], cobalt(II) tetraphenylporphyrin (Co(TPP), **12**)[2,3], manganese(III) tetraphenylporphyrin chloride (Mn(TPP)Cl, **13**)[2], chromium(III) tetraphenylporphyrin chloride (Cr(TPP)Cl, **14**)[4], iron(III) salophene chlorides (**16**, **18**, **20**)[5,6], iron(II) salen **22**[7,8], chiral iron(III) porphyrin complexes (*S,S*)-**24**[2,9,10], (*1S,4R,5R,8S*)-**26**[11], (*S*)-**28**[12,13], iron(III) Jacobsen-salen chloride ((*R,R*)-**30**)[14], and the iron(III) salen complex (*R*)-**32**[15–17] all were prepared according to literature procedures. Commercial Fe(TPP)Cl was also used for comparison, and no differences in catalytic activities were determined between commercial Fe(TPP)Cl and homemade Fe(TPP)Cl.

2. Optimization studies

2.1 General procedure for the optimization studies with Fe(TPP)Cl

An oven-dried 10 mL Schlenk tube that was equipped with a magnetic stirring bar and a glass stopper was charged with Fe(TPP)Cl (**11**) (for instance, 35 mg, 50 µmol, 5 mol%) and terminal reducing agent (Zn or Mn powder). The tube was evacuated for 20 minutes, filled back with dry nitrogen, the glass stopper was replaced with a rubber septum under positive pressure of nitrogen. The required amount of the solvent to be tested was added into the reaction tube by a gas-tight syringe. Then, co-ligand, styrene (**2a**) and styrene oxide ((±)-**1a**; 120 mg, ca. 114 µL, 1.0 mmol, 1.00 equiv) were added to the reaction mixture sequentially. Under positive pressure of nitrogen, the rubber septum was replaced with a joint glass stopper again. The tube was gently evacuated and quickly back-filled with nitrogen rapidly using the vacuum-gas line system. If necessary, the mixture was heated to 60 °C in an oil bath under nitrogen for 16 hours. The progress of the reaction was followed by TLC. After stirring at the indicated temperature for 16 h, the mixture was allowed cool to ambient temperature and filtered through a short plug of silica gel (about 5 cm) using Et_2O (ca. 100 mL) as the eluent. Solvents were removed by rotary evaporation under reduced pressure. The residue was purified by flash column chromatography on silica gel eluting with *n*-hexane/CH_2Cl_2 (1:1, v/v) to give the products (±)-**3aa** and (±)-**4aa** as a mixture of diastereomers. The *cis/trans* diastereomeric ratio ((±)-**3aa** : (±)-**4aa**) was determined by ^{1}H NMR spectroscopy.

Table S1. Effect of co-ligand on the iron porphyrin-catalyzed cyclization of styrene oxide ((±)-**1a**) with styrene ((±)-**2a**).

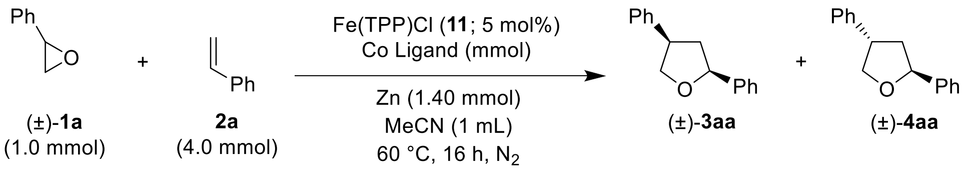

Entry	Co-ligand (mmol)	Yield [%][a]	d.r.[b] (±)-3aa : (±)-4aa
1	DMAP (0.30)	NC[c]	—
2	NMI (0.30)	NC	—
3	2,6-Lutidine (0.30)	84	77:23
**4**	**Et_3N (0.30)**	**94**	**78:22**
5	Et_3N (0.20)	80	78:22
6	Et_3N (0.075)	36	59:41
7	Et_3N (0.30), **in the dark**	94	78:22
8	Et_3N (0.30), **in the absence of Zn**	NC	—

[a] Y: Yield. Yields refer to isolated yields of NMR-pure products. [b] d.r.: Diastereomeric ratio. Diastereomeric ratios (***cis/trans***; (±)-**3aa**:(±)-**4aa**) are based on ^{1}H NMR spectroscopy of the crude reaction mixture. [c] NC: No conversion.

Table S2. Effect of styrene amount on the iron porphyrin-catalyzed cyclization of styrene oxide ((±)-**1a**) with styrene ((±)-**2a**).

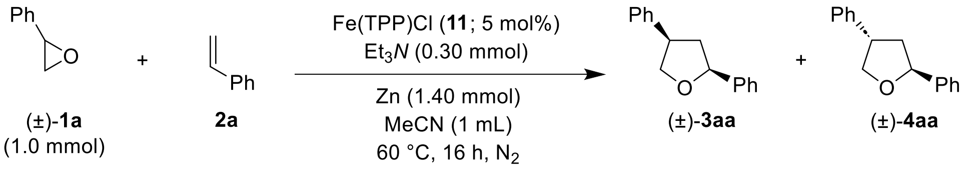

Entry	Styrene (2a, mmol)	Yield [%][a]	d.r.[b] (±)-3aa : (±)-4aa
**1**	**4.0**	**94**	**78:22**
2	3.0	76	78:22
3	2.0	29	78:22
4	1.5	<10	ND[c]

[a] Y: Yield. Yields refer to isolated yields of NMR-pure products. [b] d.r.: Diastereomeric ratio. Diastereomeric ratios (***cis/trans***; (±)-**3aa**:(±)-**4aa**) are based on ^{1}H NMR spectroscopy of the crude reaction mixture. [c] ND: Not determined.

Table S3. Effect of solvent on the iron porphyrin-catalyzed cyclization of styrene oxide ((±)-**1a**) with styrene ((±)-**2a**).

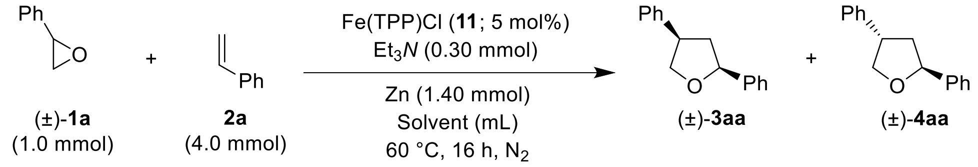

Entry	Solvent (mL)	Yield [%][a]	d.r.[b] (±)-**3aa** : (±)-**4aa**
1	Toluene (1 mL)	<10	ND[d]
2	THF (1 mL)	NC[c]	ND[d]
3	MeCN (1 mL)	94	78:22
**4**	**MeCN (0.5 mL)**	**98**	**78:22**

[a] Y: Yield. Yields refer to isolated yields of NMR-pure products. [b] d.r.: Diastereomeric ratio. Diastereomeric ratios (***cis/trans***; (±)-**3aa**:(±)-**4aa**) are based on ^{1}H NMR spectroscopy of the crude reaction mixture. [c] NC: No conversion. [d] ND: Not determined.

Table S4. Effect of temperature on the iron porphyrin-catalyzed cyclization of styrene oxide ((±)-**1a**) with styrene ((±)-**2a**).

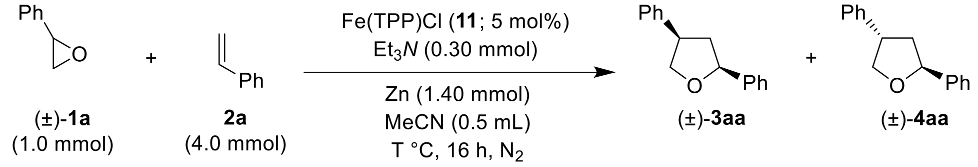

Entry	Temperature (°C)	Yield [%][a]	d.r.[b] (±)-**3aa** : (±)-**4aa**
**1**	**60**	**98**	**78:22**
2	rt	80	78:22

[a] Y: Yield. Yields refer to isolated yields of NMR-pure products. [b] d.r.: Diastereomeric ratio. Diastereomeric ratios (***cis/trans***; (±)-**3aa**:(±)-**4aa**) are based on ^{1}H NMR spectroscopy of the crude reaction mixture.

Table S5. Effect of terminal reducing agent on the iron porphyrin-catalyzed cyclization of styrene oxide ((±)-**1a**) with styrene ((±)-**2a**).

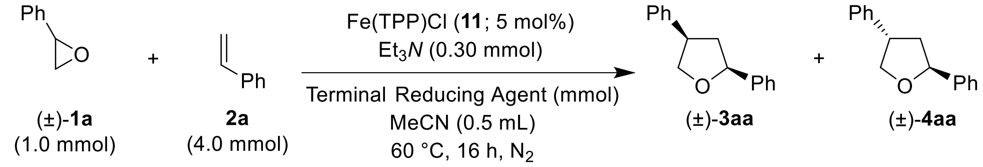

Entry	Terminal Reducing Agent (mmol)	Yield [%][a]	d.r.[b] (±)-**3aa** : (±)-**4aa**
**1**	**Zn (1.40)**	**98**	**78:22**
2	Zn (0.50	Low conversion	ND[c]
3	Mn (1.40)	Low conversion	ND[c

[a] Y: Yield. Yields refer to isolated yields of NMR-pure products. [b] d.r.: Diastereomeric ratio. Diastereomeric ratios (***cis/trans***; (±)-**3aa**:(±)-**4aa**) are based on ^{1}H NMR spectroscopy of the crude reaction mixture. [c] ND: Not determined.

3. Procedure for the kinetic studies on the iron porphyrin-catalyzed ring-expansion reaction of styrene oxide ((±)-1a) with styrene (2a)

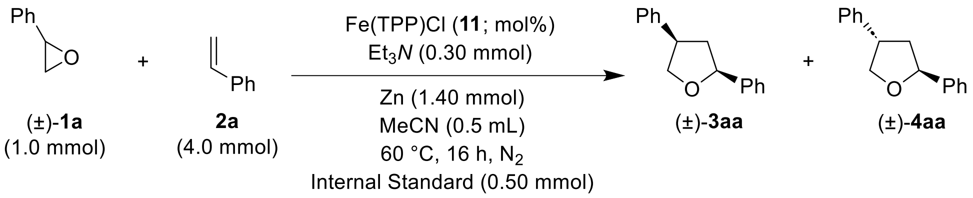

The reactions were carried out in the presence of varying amounts of Fe(TPPP)Cl: In an oven-dired 10 mL Schlenk tube were placed Fe(TPP)Cl (**11**) and zinc dust (92 mg, 1.4 mmol, 1.40 equiv). The tube was evacuated for 20 minutes, filled back with dry nitrogen, the glass stopper was replaced with a rubber septum under positive pressure of nitrogen. 0.5 mL dry acetonitrile, triethylamine (30.4 mg, ca. 42 µL, 0.3 mmol, 0.30 equiv), styrene (**2a**, 417 mg, ca. 460 µL, 4.0 mmol, 4.00 equiv), styrene oxide ((±)-**1a**), 120 mg, ca. 114 µL, 1.0 mmol, 1.00 equiv), and diphenyl ether (as internal standard, 85 mg, 79 µL, 0.50 mmol, 0.50 equiv) were added sequentially. Under positive pressure of nitrogen, the rubber septum was replaced with a joint glass stopper again. The tube was gently evacuated and quickly back-filled with nitrogen rapidly using the vacuum-gas line system. The mixture was heated to 60 °C in an oil bath under nitrogen for 16 hours. The progress of the reaction was monitored by GC.

GC: Method: Mehmet-1; HP-5 Column, 30 m, 0.32 mm ID; Inlet: 270 °C (Split Modus 1:50); Detector (FID): 300 °C, He, 1 ml/min (constant flow modus); Oven: 100 °C (10 min), 10 °C/min, 200 °C (10 min), 10 °C/min, 280 °C (5 min); t_R = 3.4 min (**2a**, styrene), t_R = 6.2 min ((±)-**1a**, styrene oxide), t_R = 16.2 min (diphenyl ether),t_R = 24.3 min ((±)-**3aa**), t_R = 24.5 min ((±)-**4aa**)

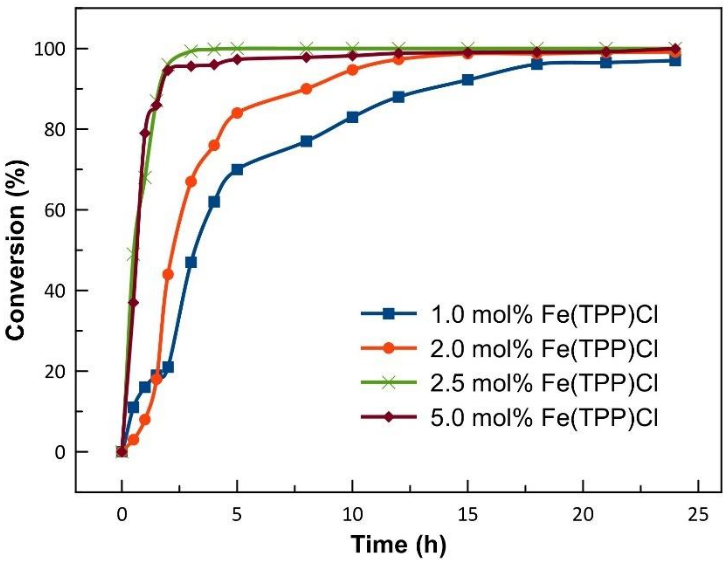

Figure S1. Kinetic profile of the iron porphyrin-catalyzed intermolecular ring-expansion reaction of styrene oxide ((±)-1a) with styrene (2a).

4. Synthetic applications

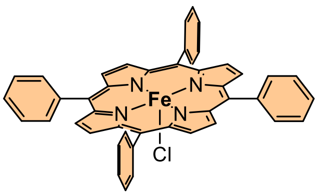

Fe(TPP)Cl (**11**)

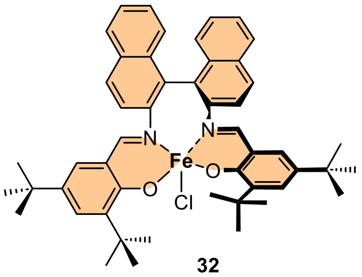

32

Figure S2. Iron catalysts used in synthetic applications.

4.1 *cis*-2,4-Diphenyltetrahydrofuran ((±)-3aa) and *trans*-2,4-diphenyltetrahydrofuran ((±)-4aa) (General Procedure 1)

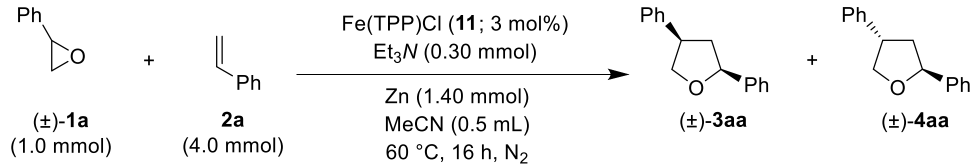

An oven-dried 10 mL Schlenk tube that was equipped with a magnetic stirring bar and a glass stopper was charged with Fe(TPP)Cl (**11**; 21 mg, 30 µmol, 3 mol%) and zinc dust (92 mg, 1.4 mmol, 1.40 equiv). The tube was evacuated for 20 minutes, filled back with dry nitrogen, the glass stopper was replaced with a rubber septum under positive pressure of nitrogen. Dry acetonitrile (0.5 mL), triethylamine (30.4 mg, ca. 42 µL, 0.3 mmol, 0.30 equiv), styrene (**2a**; 417 mg, ca. 460 µL, 4.0 mmol, 4.00 equiv) and styrene oxide ((±)-**1a**; 120 mg, 114 µL, 1.0 mmol, 1.00 equiv) were sequentially added into the reaction tube by means of gas-tight syringes. Under positive pressure of nitrogen, the rubber septum was replaced with a joint glass stopper again. The tube was gently evacuated and quickly back-filled with nitrogen using the vacuum-gas line system. The mixture was heated to 60 °C in an oil bath under nitrogen for 16 hours. The progress of the reaction was followed by TLC. The mixture was allowed cool to ambient temperature and filtered through a short plug of silica gel (about 5 cm) using Et_2O (ca. 100 mL) as the eluent. Solvents were removed by rotary evaporation under reduced pressure. The residue was purified by flash column chromatography on silica gel eluting with *n*-hexane/CH_2Cl_2 (1:1, v/v) to give the products (±)-**3aa** (*(±)-Calyxolane B*) and (±)-**4aa** (*(±)-Calyxolane A*) as a mixture of diastereomers (220 mg, 0.98 mmol, 98%), as a colorless oil. The *cis/trans* diastereomeric ratio ((±)-**3aa** : (±)-**4aa**) was determined to be 78:22 by ^{1}H NMR spectroscopy.

TLC: R_f = 0.50 (silica gel; *n*-hexane/CH_2Cl_2, 1:1, v/v) (±)-**3aa** and (±)-**4aa**

R_f = 0.28 (silica gel; *n*-hexane/EtOAc, 10:0.4, v/v) (±)-**3aa**

R_f = 0.31 (silica gel; *n*-hexane/EtOAc, 10:0.4, v/v) (±)-**4aa**

FTIR (KBr): $\tilde{\nu}_{max}$ (cm^{-1}) = 3085 (w), 3062 (m), 3029 (m), 2970 (m), 2937 (m), 2864 (m), 1603 (m), 1495 (s), 1452 (m), 136 (w), 1308 (w), 1287 (w), 1214 (w), 1156 (w), 1086 (m), 1064 (s), 1049 (s), 1029 (m), 993 (m), 923 (m), 845 (w), 754 (s), 699 (s), 661 (w), 597 (w), 534 (m).

GCMS: t_R = 30.16 min ((±)-**3aa**), t_R = 30.28 min ((±)-**4aa**); *m/z* (%) = 224 ([M]$^+$, 28), 193 ([M−31]$^+$, 100), 179 ([M−45]$^+$, 49), 117 (96), 91 (45), 77 (28).

GC: t_R = 24.3 min ((±)-**3aa**), t_R = 24.5 min ((±)-**4aa**).

HRMS (ESI): *m/z* calcd for $C_{16}H_{16}NaO$ ([M + Na]$^+$): 247.1099; found: 247.1092.

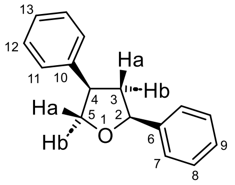

(±)-**3aa**

^{1}H NMR (500 MHz, $CDCl_3$): δ = 7.42–7.21 (m, 10H, H_{aryl}), 5.07 (dd, *J* = 10.2 Hz, *J* = 5.7 Hz, 1H, H2), 4.36 (t, *J* = 8.2 Hz, 1H, $H5_a$), 4.02 (t, *J* = 8.5 Hz, 1H, $H5_b$), 3.67–3.60 (m, 1H, H4), 2.78–2.73 (m, 1H, $H3_a$), 2.02 (dd, *J* = 22.9 Hz, *J* = 10.5 Hz, 1H, $H3_b$).

^{13}C NMR (APT, 125 MHz, $CDCl_3$): δ = 142.6 (C, C6), 141.7 (C, C10), 128.6 (CH, C7), 128.4 (CH, C11), 127.4 (CH, C9), 127.2 (CH, C8), 126.6 (CH, C13), 125.7 (CH, C12), 81.8 (CH, C2), 75.1 (CH_2, C5), 46.0 (CH, C4),43.7 (CH_2, C3).

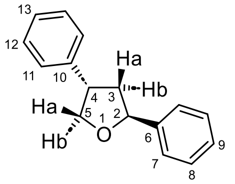

(±)-**4aa**

^{1}H NMR (500 MHz, $CDCl_3$): δ = 7.38–7.22 (m, 10H, H_{aryl}), 5.23 (dd, *J* = 7.7 Hz, *J* = 5.9 Hz, 1H, H2), 4.47 (t, *J* = 8.0 Hz, 1H, $H5_b$), 3.95 (t, *J* = 8.3 Hz, 1H, $H5_a$), 3.56–3.50 (m, 1H, H4), 2.50–2.45 (m, 1H, $H3_b$), 2.36–2.31 (m, 1H, $H3_a$).

^{13}C NMR (APT, 125 MHz, CDCl$_3$): δ = 143.6 (C, C6), 142.0 (C, C10), 128.6 (CH, C7), 128.4 (CH, C11), 127.3 (CH, C8), 127.2 (CH, C9), 126.6 (CH, C13), 125.5 (CH, C12), 80.6 (CH, C2), 75.1 (CH$_2$, C5), 44.4 (CH, C4), 42.7 (CH$_2$, C3).

4.2 (2*S*,4*R*)-2,4-Diphenyltetrahydrofuran ((2*S*,4*R*)-3aa, *Calyxolane B*) and (2*S*,4*S*)-2,4-diphenyltetrahydrofuran ((2*S*,4*S*)-4aa, *Calyxolane A*) (General Procedure 2)

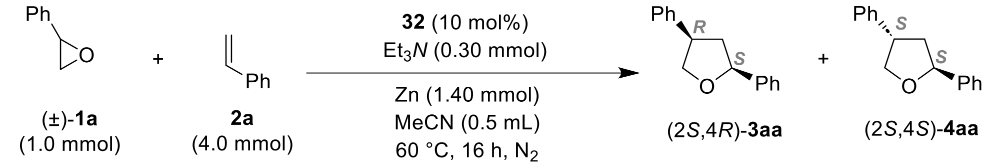

An oven-dried 10 mL Schlenk tube that was equipped with a magnetic stirring bar and a glass stopper was charged with **catalyst 32** (81 mg, 100 µmol, 10 mol%) and zinc dust (92 mg, 1.4 mmol, 1.40 equiv). The tube was evacuated for 20 minutes, filled back with dry nitrogen, the glass stopper was replaced with a rubber septum under positive pressure of nitrogen. Dry acetonitrile (0.5 mL), triethylamine (30.4 mg, ca. 42 µL, 0.3 mmol, 0.30 equiv), styrene (**2a**; 417 mg, ca. 460 µL, 4.0 mmol, 4.00 equiv) and styrene oxide ((±)-**1a**; 120 mg, 114 µL, 1.0 mmol, 1.00 equiv) were sequentially added into the reaction tube by means of gas-tight syringes. Under positive pressure of nitrogen, the rubber septum was replaced with a joint glass stopper again. The tube was gently evacuated and quickly back-filled with nitrogen using the vacuum-gas line system. The mixture was heated to 60 °C in an oil bath under nitrogen for 16 hours. The progress of the reaction was followed by TLC. The mixture was allowed cool to ambient temperature and filtered through a short plug of silica gel (about 5 cm) using Et$_2$O (ca. 100 mL) as the eluent. *cis/trans* (**3aa**/**4aa**) diastereomeric ratio was determined to be 47:53 by GC and GCMS analysis. Solvents were removed by rotary evaporation under reduced pressure. The residue was purified by flash column chromatography on silica gel eluting with *n*-hexane/CH$_2$Cl$_2$ (1:1, v/v), thus, the products were obtained as a mixture of the diastereomers (2*S*,4*R*)-**3aa** and (2*S*,4*S*)-**4aa** (190 mg, 0.85 mmol, 85%). Isolation of individual diastereomers were then performed by flash column chromatography on silica gel using *n*-hexane/EtOAc mixture (10:0.4, v/v) as eluent. Thus, enantiomerically enriched (2*S*,4*R*)-(–)-2,4-diphenyltetrahydrofuran ((2*S*,4*R*)-**3aa**; *Calyxolane B*, 83% *ee*) and (2*S*,4*S*)-(+)-2,4-diphenyltetrahydrofuran ((2*S*,4*S*)-**4aa**; *Calyxolane A*, 92% *ee*) were obtained as colorless oils.

HPLC: Column: CHIRALCEL OD-H, 25 cm, 0.46 cm ID; Eluent: *n*-hexane/*i*-PrOH (95:5); Flow rate: 0.5 mL/min; Pressure: 29 bar; Detection: 254 nm.

t_R = 13.8 min ((2*R*,4*S*)-**3aa**)

t_R = 18.3 min ((2*S*,4*R*)-**3aa**)

t_R = 14.1 min ((2*R*,4*R*)-**4aa**)

t_R = 15.1 min ((2*S*,4*S*)-**4aa**)

*Assignments of the absolute configurations of **3aa** and **4aa** were made by comparing the signs of their optical rotations with those reported in the literature.*[18–21]

Specific Rotation: (2*S*,4*R*)-**3aa** (*Calyxolane B*, 83% *ee*): $[\alpha]_D^{24}$ = –41.1 (*c* = 2.4, CHCl$_3$). For comparison, see, references.[19,20]

(2*S*,4*S*)-**4aa** (*Calyxolane A*, 92% *ee*): $[\alpha]_D^{24}$ = +17.8 (*c* = 2.1, CHCl$_3$). For comparison, see, references.[18,20]

4.3 *cis*-2-(2-Naphthyl)-4-phenyltetrahydrofuran ((±)-3ab) and *trans*-2-(2-naphthyl)-4-phenyltetrahydrofuran ((±)-4ab)

According to General Procedure 1, styrene oxide ((±)-**1a**; 120 mg, 114 µL, 1.0 mmol, 1.00 equiv) was treated with 2-vinylnaphthalene (**2b**; 617mg, 4.0 mmol, 4.00 equiv), Fe(TPP)Cl (21 mg, 30 µmol, 3 mol%), Zn dust (92 mg, 1.4 mmol, 1.40 equiv) and Et$_3$N (30.4 mg, ca. 42 µL, 0.3 mmol, 0.30 equiv) in anhydrous MeCN (0.5 mL), at 60 °C for 16 h under N$_2$. The crude product was purified by flash column chromatography on silica gel to afford (±)-**3ab** and (±)-**4ab** as a diasteromeric mixture (230 mg, 0.84 mmol, 84%), as a colorless and slightly viscous liquid. The *cis/trans* diastereomeric ratio ((±)-**3ab** : (±)-**4ab**) was determined to be 78:22 by ^{1}H NMR spectroscopy.

TLC: R_f = 0.50 (silica gel; *n*-hexane/CH$_2$Cl$_2$, 1:1, v/v) (±)-**3ab** and (±)-**4ab**

R_f = 0.25 (silica gel; *n*-hexane/EtOAc, 10:0.4, v/v) (±)-**3ab**

R_f = 0.30 (silica gel; *n*-hexane/EtOAc, 10:0.4, v/v) (±)-**4ab**

FTIR (KBr): $\tilde{\nu}_{max}$ (cm^{-1}) = 3435 (br), 3056 (m), 3026 (m), 2964 (m), 2938 (m), 2863 (m), 1947 (w), 1692 (w), 1633 (w), 1600 (m), 1491 (m), 1454 (m), 1371 (m), 1327 (m), 1273 (w), 1181 (w), 1095 (m), 1067 (m), 1043 (s), 990 (m), 962 (m), 914 (m), 864 (m), 855 (m), 819 (s), 757 (s), 699 (s), 568 (w), 520(m), 476 (s).

GCMS: t_R = 34.66 min ((±)-**3ab**), t_R = 34.88 min ((±)-**4ab**); *m/z* (%) = 274 ($[M]^+$, 70), 244 ($[M-30]^+$, 14), 229 ($[M-45]^+$, 12), 207 (100), 191 (13), 155 (50), 141 (14), 117 (26), 91 (20), 44 (25), 28 (56).

GC: t_R = 44.6 min ((±)-**3ab**), t_R = 44.8 min ((±)-**4ab**).

HRMS (ESI): *m/z* calcd for $C_{20}H_{18}NaO$ ($[M+Na]^+$): 297.1358; found: 297.1352.

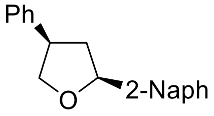

(±)-**3ab**

Mp: 97–98 °C.

^{1}H NMR (500 MHz, $CDCl_3$): δ = 7.87–7.82 (m, 4H), 7.53–7.44 (m, 3H), 7.33–7.28 (m, 4H), 7.24–7.21 (m, 1H), 5.25 (dd, *J* = 10.1 Hz, *J* = 5.8 Hz, 1H), 4.42 (t, *J* = 8.2 Hz, 1H), 4.09 (t, *J* = 8.5 Hz, 1H), 3.73–3.66 (m, 1H), 2.86–2.81 (m, 1H), 2.10 (dd, *J* = 23.4 Hz, *J* = 10.5 Hz, 1H).

^{13}C NMR (APT, 125 MHz, $CDCl_3$): δ = 141.6 (C), 140.1 (C), 133.3 (C), 132.9 (C), 128.6 (CH), 128.3 (CH), 127.9 (CH), 127.7 (CH), 127.3 (CH), 126.7 (CH), 126.1 (CH), 125.7 (CH), 124.2 (CH), 124.0 (CH), 81.9 (CH), 75.2 (CH_2), 46.1 (CH), 43.7 (CH_2).

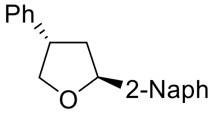

(±)-**4ab**

Mp: 75–76 °C.

^{1}H NMR (500 MHz, $CDCl_3$): δ = 7.85–7.82 (m, 4H), 7.49–7.44 (m, 3H), 7.35–7.30 (m, 4H), 7.26–7.23 (m, 1H), 5.38 (dd, *J* = 7.6 Hz, *J* = 1.6 Hz, 1H), 4.54 (t, *J* = 7.6 Hz, 1H), 4.01 (t, *J* = 8.3 Hz, 1H), 3.60–3.54 (m, 1H), 2.57–2.51 (m, 1H), 2.43–2.38 (m, 1H).

^{13}C NMR (APT, 125 MHz, $CDCl_3$): δ = 141.9 (C), 140.9 (C), 133.8 (C), 132.8 (C), 128.6 (CH), 128.2 (CH), 127.9 (CH), 127.6 (CH), 127.3 (CH), 126.6 (CH), 126.1 (CH), 125.7 (CH), 123.9 (CH), 123.8 (CH), 80.7 (CH), 75.2 (CH_2), 44.4 (CH), 42.6 (CH_2).

4.4 (2*S*,4*R*)-2-(2-Nahpthyl)-4-phenyltetrahydrofuran ((2*S*,4*R*)-3ab) and (2*S*,4*S*)-2-(2-nahpthyl)-4-phenyltetrahydrofuran ((2*S*,4*S*)-4ab)

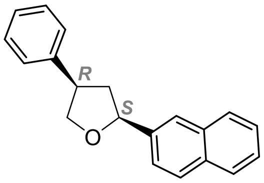

(2*S*,4*R*)-**3ab**

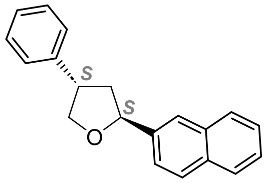

(2*S*,4*S*)-**4ab**

According to General Procedure 2, styrene oxide ((±)-**1a**; 120 mg, 114 µL, 1.0 mmol, 1.00 equiv) was treated with 2-vinylnaphthalene (**2b**; 617mg, 4.0 mmol, 4.00 equiv), **32** (81 mg, 100 µmol, 10 mol%), Zn dust (92 mg, 1.4 mmol, 1.40 equiv) and Et_3N (30.4 mg, ca. 42 µL, 0.3 mmol, 0.30 equiv) in anhydrous MeCN (0.5 mL), at 60 °C for 16 h under N_2. The *cis/trans* diastereomeric ratio (**3ab** : **4ab**) was determined to be 52:48 by GC and GCMS analysis of the crude product. The crude product was purified by flash column chromatography on silica gel to afford (2*S*,4*R*)-**3ab** and (2*S*,4*S*)-**4ab** as a diasteromeric mixture (175 mg, 0.64 mmol, 64%), as a colorless solid. Isolation of individual diastereomers were then carried out by flash column chromatography on silica gel using *n*-hexane/EtOAc mixture (10:0.4, v/v) as eluent. Thus, enantiomerically enriched (2*S*,4*R*)-**3ab** (19% *ee*) and (2*S*,4*S*)-**4ab** (86% *ee*) were obtained as colorless solids.

HPLC: Column: Chiralcel OD-H, 25 cm, 0.46 cm ID; Eluent: *n*-hexane/*i*-PrOH (95:5); Flow rate: 1.0 mL/min; Pressure: 42 bar; Detection: 254 nm.

t_R = 8.8 min ((2*S*,4*R*)-**3ab**, the major enantiomer of **3ab**)

t_R = 15.3 min ((2*R*,4*S*)-**3ab**, the minor enantiomer of **3ab**)

t_R = 8.7 min ((2*S*,4*S*)-**4ab**, the major enantiomer of **4ab**)

t_R = 10.1 min ((2*R*,4*R*)-**4ab**, the minor enantiomer of **4ab**)

*The proposed absolute configurations of **3ab** and **4ab** have not been supported by any chiroptical technique and are based entirely on the results obtained for **3aa** and **4aa** by the same catalytic system.*

Specific Rotation: (2*S*,4*R*)-**3ab** (19% *ee*): $[\alpha]_D^{24}$ = –2.7 (*c* = 0.5, $CHCl_3$)

(2*S*,4*S*)-**4ab** (86% *ee*): $[\alpha]_D^{24}$ = +14.2 (*c* = 0.5, $CHCl_3$)

4.5 *cis*-2-(4-Methoxyphenyl)-4-phenyltetrahydrofuran ((±)-3ac) and *trans*-2-(4-metoxyphenyl)-4-phenyltetrahydrofuran ((±)-4ac)

According to General Procedure 1, styrene oxide ((±)-**1a**; 120 mg, 114 μL, 1.0 mmol, 1.00 equiv) was treated with 4-methoxystyrene (**2c**; 537 mg, ca. 536 μL, 4.0 mmol, 4.00 equiv), Fe(TPP)Cl (21 mg, 30 μmol, 3 mol%), Zn dust (92 mg, 1.4 mmol, 1.40 equiv) and Et_3N (30.4 mg, ca. 42 μL, 0.3 mmol, 0.30 equiv) in anhydrous MeCN (0.5 mL), at 60 °C for 16 h under N_2. The crude product was purified by flash column chromatography on silica gel to afford (±)-**3ac** and (±)-**4ac** as a diasteromeric mixture (242 mg, 0.95 mmol, 95%), as a colorless and slightly viscous liquid. The *cis/trans* diastereomeric ratio ((±)-**3ac** : (±)-**4ac**) was determined to be 83:17 by ^{1}H NMR spectroscopy.

TLC: R_f = 0.79 (silica gel; CH_2Cl_2, 100%) (±)-**3ac** and (±)-**4ac**

R_f = 0.27 (silica gel; *n*-hexane/CH_2Cl_2, 1:1, v/v) (±)-**3ac** and (±)-**4ac**

R_f = 0.18 (silica gel; *n*-hexane/EtOAc, 10:0.4, v/v) (±)-**3ac**

R_f = 0.20 (silica gel; *n*-hexane/EtOAc, 10:0.4, v/v) (±)-**4ac**

FTIR (KBr): $\tilde{\nu}_{max}$ (cm^{-1}) = 3061 (m), 3028 (m), 3001 (m), 2935 (m), 2862 (m), 2836 (m), 1613 (s), 1585 (m), 1514 (s), 1495 (s), 1455 (s), 1365 (m), 1303 (s), 1248 (s), 1173 (s), 1111 (m), 1037 (s), 995 (m), 921 (m), 830 (s), 759 (s), 701 (s), 655 (w), 579 (m), 531 (m).

GCMS: t_R = 32.87 min ((±)-**3ac**), t_R = 33.00 min ((±)-**4ac**); *m/z* (%) = 254 ([M]$^+$, 83), 224 ([M−30]$^+$, 69), 207 ([M−47]$^+$, 50), 193 ([M−61]$^+$, 36), 163 (20), 135 (100), 117 (58), 104 (23), 91 (59), 77 (25), 63 (15), 44 (21), 28 (77).

GC: t_R = 33.2 min ((±)-**3ac**), t_R = 33.6 min ((±)-**4ac**).

HRMS (ESI): *m/z* calcd for $[C_{17}H_{18}NaO_2]([M+Na]^+)$: 277.1204; found: 277.1222.

(±)-**3ac**

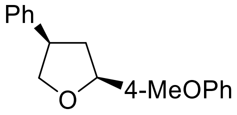

Mp: 45 °C.

^{1}H NMR (500 MHz, $CDCl_3$): *δ* = 7.35–7.21 (m, 7H), 6.90 (d, *J* = 8.7 Hz, 2H), 5.01 (dd, *J* = 10.2 Hz, *J* = 5.6 Hz, 1H), 4.33 (t, *J* = 8.3 Hz, 1H), 4.01 (t, *J* = 8.4 Hz, 1H), 3.81 (s, 3H), 3.66-3.59 (m, 1H), 2.73–2.68 (m, 1H), 2.00 (dd, *J* = 22.9 Hz, *J* = 10.4 Hz, 1H).

^{13}C NMR (APT, 125 MHz, $CDCl_3$): *δ* = 159.0 (C), 142.0 (C), 134.5 (C), 128.6 (CH), 127.2 (CH), 127.1 (CH), 126.6 (CH), 113.8 (CH), 81.6 (CH), 74.9 (CH_2), 55.3 (CH_3), 46.0 (CH), 43.7 (CH_2).

(±)-**4ac**

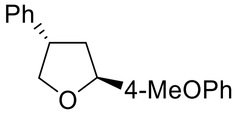

^{1}H NMR (500 MHz, $CDCl_3$): *δ* = 7.35–7.28 (m, 6H), 7.25–7.22 (m, 1H) 6.90 (d, *J* = 8.7 Hz, 2H), 5.17 (t, *J* = 7.1 Hz, 1H), 4.45 (dd, *J* = 8.4 Hz, *J* = 7.5 Hz, 1H), 3.92 (t, *J* = 8.2 Hz, 1H), 3.81 (s, 3H), 3.58-3.51 (m, 1H), 2.46–2.40 (m, 1H), 2.34–2.29 (m, 1H).

^{13}C NMR (APT, 125 MHz, $CDCl_3$): *δ* = 158.9 (C), 142.2 (C), 135.5 (C), 128.6 (CH), 127.3 (CH), 126.8 (CH), 126.6 (CH), 113.8 (CH), 80.4 (CH), 75.0 (CH_2), 55.3 (CH_3), 44.6 (CH), 42.6 (CH_2).

4.6 (2*S*,4*R*)-2-(4-Methoxyphenyl)-4-phenyltetrahydrofuran ((2*S*,4*R)*-3ac) and (2*S*,4*S*)-2-(4-methoxyphenyl)-4-phenyltetrahydrofuran ((2*S*,4*S*)-4ac)

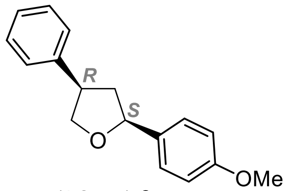

(2*S*,4*R*)-**3ac**

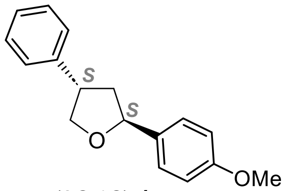

(2*S*,4*S*)-**4ac**

According to General Procedure 2, styrene oxide ((±)-**1a**; 120 mg, 114 μL, 1.0 mmol, 1.00 equiv) was treated with 4-methoxystyrene (**2c**; 537 mg, ca. 536 μL, 4.0 mmol, 4.00 equiv), **32** (81 mg, 100 μmol, 10 mol%), Zn dust (92 mg, 1.4 mmol, 1.40 equiv) and Et_3N (30.4 mg, ca. 42 μL, 0.3 mmol, 0.30 equiv) in anhydrous MeCN (0.5 mL), at 60 °C for 16 h under N_2. The *cis/trans* diastereomeric ratio (**3ac** : **4ac**) was determined to be 56:44 by GC and GCMS analysis of the crude product. The crude product was purified by flash column chromatography on silica gel to afford (2*S*,4*R*)-**3ac** and (2*S*,4*S*)-**4ac** as a diasteromeric mixture (140 mg, 0.55 mmol, 55%). Isolation of individual diastereomers were then carried out by flash column chromatography on silica gel using *n*-hexane/EtOAc mixture (10:0.4, v/v) as eluent. Thus, enantiomerically enriched (2*S*,4*R*)-**3ac** (53% *ee*) and (2*S*,4*S*)-**4ac** (82% *ee*) were obtained.

HPLC: Column: Chiralcel OD-H, 25 cm, 0.46 cm ID; Eluent: *n*-hexane/*i*-PrOH (99:1); Flow rate: 1.0 mL/min; Pressure: 38 bar; Detection: 254 nm.

t_R = 24.1 min ((2*R*,4*S*)-**3ac**, the minor enantiomer of **3ac**)

t_R = 25.7 min ((2*S*,4*R*)-**3ac**, the major enantiomer of **3ac**)

Column: Chiralcel AD-H, 25 cm, 0.46 cm ID; Eluent: *n*-hexane/*i*-PrOH (99:1); Flow rate: 1.0 mL/min; Pressure: 42 bar; Detection: 254 nm.

t_R = 13.8 min ((2*R*,4*R*)-**4ac**, the minor enantiomer of **4ac**)

t_R = 17.3 min ((2*S*,4*S*)-**4ac**, the major enantiomer of **4ac**)

*The proposed absolute configurations of **3ac** and **4ac** have not been supported by any chiroptical technique and are based entirely on the results obtained for **3aa** and **4aa** by the same catalytic system.*

Specific Rotation: (2*S*,4*R*)-**3ac** (53% *ee*): $[\alpha]_D^{24}$ = –8.4 (*c* = 0.4, $CHCl_3$)

(2*S*,4*S*)-**4ac** (82% *ee*): $[\alpha]_D^{24}$ = +11.3 (*c* = 0.5, $CHCl_3$)

4.7 *cis*-2-(4-Chlorophenyl)-4-phenyltetrahydrofuran ((±)-3ad) and *trans*-2-(4-chlorophenyl)-4-phenyltetrahydrofuran ((±)-4ad)

According to General Procedure 1, styrene oxide ((±)-**1a**; 120 mg, 114 μL, 1.0 mmol, 1.00 equiv) was treated with 4-chlorostyrene (**2d**; 554 mg, 480 μL, 4.0 mmol, 4.00 equiv), Fe(TPP)Cl (21 mg, 30 μmol, 3 mol%), Zn dust (92 mg, 1.4 mmol, 1.40 equiv) and Et_3N (30.4 mg, ca. 42 μL, 0.3 mmol, 0.30 equiv) in anhydrous MeCN (0.5 mL), at 60 °C for 16 h under N_2. The crude product was purified by flash column chromatography on silica gel to afford (±)-**3ad** and (±)-**4ad** as a diasteromeric mixture (223 mg, 0.86 mmol, 86%), as a colorless and slightly viscous liquid. The *cis/trans* diastereomeric ratio ((±)-**3ad** : (±)-**4ad**) was determined to be 74:26 by ^1H NMR spectroscopy.

TLC: R_f = 0.66 (silica gel; *n*-hexane/CH_2Cl_2, 1:1, v/v) (±)-**3ad** and (±)-**4ad**

R_f = 0.23 (silica gel; *n*-hexane/EtOAc, 10:0.4, v/v) (±)-**3ad**

R_f = 0.28 (silica gel; *n*-hexane/EtOAc, 10:0.4, v/v) (±)-**4ad**

FTIR (KBr): $\tilde{\nu}_{max}$ (cm^{-1}) = 3307 (br w), 3086 (w), 3062 (w), 3029 (w), 2971 (w), 2941 (w), 2870 (m), 1686 (w), 1602 (m), 1493 (s), 1455 (m), 1400 (m), 1357 (m), 1297 (m), 1261 (w), 1174 (w), 1091 (s), 1066 (s), 1047 (s), 1014 (s), 922 (w), 827 (s), 759 (s), 700 (s), 631 (w), 514 (s).

GCMS: t_R = 32.18 min ((±)-**3ad**), t_R = 32.31 min ((±)-**4ad**); *m/z* (%) = 258 ($[M]^+$, 13), 228 ($[M-30]^+$, 33), 207 ($[M-51]^+$, 66), 193 ($[M-65]^+$, 100), 178 (24), 167 (25), 139 (29), 117 (64), 103 (32), 91 (42), 77 (23), 44 (19), 28 (83).

GC: t_R = 31.7 min ((±)-**3ad**), t_R = 32.1 min ((±)-**4ad**).

HRMS (ESI): *m/z* calcd for $[C_{16}H_{15}ClNaO]([M+Na]^+)$: 281.0709; found: 281.0707.

(±)-**3ad**

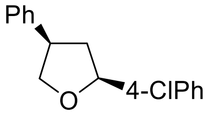

Mp: 55–56 °C.

^{1}H NMR (500 MHz, $CDCl_3$): δ = 7.34–7.29 (m, 6H), 7.25–7.20 (m, 3H), 5.02 (dd, J = 10.1 Hz, J = 5.8 Hz, 1H), 4.34 (t, J = 8.2 Hz, 1H), 3.99 (t, J = 8.5 Hz, 1H), 3.65–3.58 (m, 1H), 2.75–2.70 (m, 1H), 1.97–1.90 (m, 1H).

^{13}C NMR (APT, 125 MHz, $CDCl_3$): δ = 141.4 (C), 141.2 (C), 133.0 (C), 128.6 (CH), 128.5 (CH), 127.1 (CH), 127.0 (CH), 126.7 (CH), 81.1 (CH), 75.0 (CH_2), 45.9 (CH), 43.7 (CH_2).

(±)-**4ad**

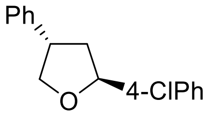

^{1}H NMR (500 MHz, $CDCl_3$): δ = 7.35–7.23 (m, 9H), 5.19 (dd, J = 7.7 Hz, J = 5.8 Hz, 1H), 4.45 (dd, J = 8.5 Hz, J = 7.4 Hz, 1H), 3.94 (t, J = 8.4 Hz, 1H), 3.54–3.47 (m, 1H), 2.49–2.44 (m, 1H), 2.30–2.24 (m, 1H).

^{13}C NMR (APT, 125 MHz, $CDCl_3$): δ = 142.1 (C), 141.7 (C), 132.8 (C), 128.7 (CH), 128.5 (CH), 127.3 (CH), 126.9 (CH), 126.7 (CH), 79.9 (CH), 75.1 (CH_2), 44.3 (CH), 42.7 (CH_2).

4.8 (2*S*,4*R*)-2-(4-Chlorophenyl)-4-phenyltetrahydrofuran ((2*S*,4*R*)-3ad) and (2*S*,4*S*)-2-(4-chlorophenyl)-4-phenyltetrahydrofuran ((2*S*,4*S*)-4ad)

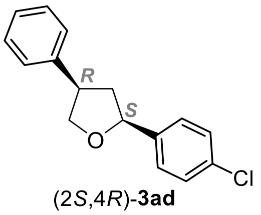

(2*S*,4*R*)-**3ad**

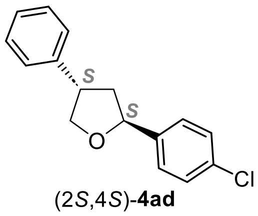

(2*S*,4*S*)-**4ad**

According to General Procedure 2, styrene oxide ((±)-**1a**; 120 mg, 114 µL, 1.0 mmol, 1.00 equiv) was treated with 4-chlorostyrene (**2d**; 554 mg, 480 µL, 4.0 mmol, 4.00 equiv), **32** (81 mg, 100 µmol, 10 mol%), Zn dust (92 mg, 1.4 mmol, 1.40 equiv) and Et_3N (30.4 mg, ca. 42 µL, 0.3 mmol, 0.30 equiv) in anhydrous MeCN (0.5 mL), at 60 °C for 16 h under N_2. The *cis/trans* diastereomeric ratio (**3ad** : **4ad**) was determined to be 57:43 by GC and GCMS analysis of the crude product. The crude product was purified by flash column chromatography on silica gel to afford (2*S*,4*R*)-**3ad** and (2*S*,4*S*)-**4ad** as a diasteromeric mixture (225 mg, 0.87 mmol, 87%), as a colorless oil. Isolation of individual diastereomers were then carried out by flash column chromatography on silica gel using *n*-hexane/EtOAc mixture (10:0.4, v/v) as eluent. Thus, enantiomerically enriched (2*S*,4*R*)-**3ad** (74% *ee*) and (2*S*,4*S*)-**4ad** (88% *ee*) were obtained.

HPLC: Column: Chiralcel OD-H, 25 cm, 0.46 cm ID; Eluent: *n*-hexane/*i*-PrOH (95:5); Flow rate: 1.0 mL/min; Pressure: 42 bar; Detection: 254 nm.

t_R = 5.9 min ((2*S*,4*R*)-**3ad**, the major enantiomer of **3ad**)

t_R = 6.5 min ((2*R*,4*S*)-**3ad**, the minor enantiomer of **3ad**)

t_R = 6.1 min ((2*S*,4*S*)-**4ad**, the major enantiomer of **4ad**)

t_R = 6.6 min ((2*R*,4*R*)-**4ad**, the minor enantiomer of **4ad**)

*The proposed absolute configurations of **3ad** and **4ad** have not been supported by any chiroptical technique and are based basically on the results obtained for **3aa** and **4aa** by the same catalytic system. (2S,4R)-**3ad** and (2R,4S)-**3ad** are known in the literature.[22] The order of the reported HPLC retention times of the enantiomers of **3ad** recorded using a Ciralcel OD column in the literature and ours obtained using a Ciralcel OD-H column prove the proposed absolute configuration of **3ad**, (2S,4R).*

Specific Rotation: (2*S*,4*R*)-**3ad** (74% *ee*): $[\alpha]_D^{24}$ = –25.7 (c = 0.4, $CHCl_3$)

(2*S*,4*S*)-**4ad** (88% *ee*): $[\alpha]_D^{24}$ = +13.6 (c = 0.4, $CHCl_3$)

4.9 *cis*-2-(4-Fluorophenyl)-4-phenyltetrahydrofuran ((±)-3ae) and *trans*-2-(4-fluorophenyl)-4-phenyltetrahydrofuran ((±)-4ae)

According to General Procedure 1, styrene oxide ((±)-**1a**; 120 mg, 114 µL, 1.0 mmol, 1.00 equiv) was treated with 4-fluorostyrene (**2e**; 488 mg, 477 µL, 4.0 mmol, 4.00 equiv), Fe(TPP)Cl (21 mg, 30 µmol, 3 mol%), Zn dust (92 mg, 1.4 mmol, 1.40 equiv) and Et_3N (30.4 mg, ca. 42 µL, 0.3 mmol, 0.30 equiv) in anhydrous MeCN (0.5 mL), at 60 °C for 16 h under N_2. The crude product was purified by flash column chromatography on silica gel to afford (±)-**3ae** and (±)-**4ae** as a diasteromeric mixture (213 mg, 0.88 mmol, 88%), as a colorless and slightly viscous liquid. The *cis/trans* diastereomeric ratio ((±)-**3ae** : (±)-**4ae**) was determined to be 78:22 by ^{1}H NMR spectroscopy.

TLC: R_f = 0.64 (silica gel; *n*-hexane/CH_2Cl_2, 1:1, v/v) (±)-**3ae** and (±)-**4ae**

R_f = 0.20 (silica gel; *n*-hexane/EtOAc, 10:0.4, v/v) (±)-**3ae**

R_f = 0.25 (silica gel; *n*-hexane/EtOAc, 10:0.4, v/v) (±)-**4ae**

FTIR (KBr): $\tilde{\nu}_{max}$ (cm^{-1}) = 3063 (m), 3030 (m), 2971 (m), 2939 (m), 2869 (m), 1605 (s), 1510 (s), 1496 (s), 1455 (m), 1410 (w), 1350 (w), 1296 (m), 1224 (s), 1156 (s), 1066 (s), 1048 (s), 1015 (m), 994 (m), 922 (w), 834 (s), 759 (s), 700 (s), 655 (w), 561 (m), 532 (m).

GCMS: t_R = 30.06 min ((±)-**3ae**), t_R = 33.18 min ((±)-**4ae**); *m/z* (%) = 242 ($[M]^+$, 36), 212 ($[M-30]^+$, 100), 197 ($[M-45]^+$, 61), 164 (51), 151 (79), 138 (66), 117 (99), 103 (37), 91 (62), 77 (21).

HRMS (ESI): *m/z* calcd for $[C_{16}H_{15}FNaO]([M+Na]^+)$: 265.1005; found: 265.0984 (error:5.6 ppm).

(±)-**3ae**

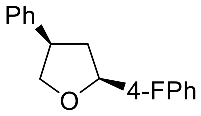

^{1}H NMR (600 MHz, $CDCl_3$): δ = 7.37–7.18 (m, 7H), 7.04-7.00 (m, 2H), 5.01 (dd, *J* = 10.2 Hz, *J* = 5.7 Hz, 1H), 4.32 (t, *J* = 8.3 Hz, 1H), 4.00 (t, *J* = 8.4 Hz, 1H), 3.63–3.57 (m, 1H), 2.72–2.68 (m, 1H), 1.95 (q, *J* = 10.4 Hz, 1H).

^{13}C NMR (APT, 150 MHz, $CDCl_3$): δ = {162.87, 161.25} (CH, *J* = 245.1 Hz, C), 141.6 (C), {138.27, 138.25} (CH, *J* = 3.0 Hz, C), 128.6 (CH), {127.33, 127.28} (CH, *J* = 8.0 Hz, CH), 127.1 (CH), 126.6 (CH), {115.21, 115.07} (CH, *J* = 21.3 Hz, CH), 81.2 (CH), 74.9 (CH_2), 45.9 (CH), 43.7 (CH_2).

(±)-**4ae**

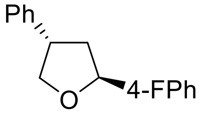

^{1}H NMR (600 MHz, $CDCl_3$): δ = 7.37–7.18 (m, 7H), 7.02 (t, *J* = 8.8 Hz, 2H), 5.17 (t, *J* = 6.6 Hz, 1H), 4.43 (t, *J* = 7.6 Hz, 1H), 3.91 (t, *J* = 8.4 Hz, 1H), 3.53–3.47 (m, 1H), 2.45–2.41 (m, 1H), 2.28–2.23 (m, 1H).

^{13}C NMR (APT, 150 MHz, $CDCl_3$): δ = {162.75, 161.13} (CH, *J* = 245.1 Hz, C), 141.8 (C), {139.16, 138.18} (CH, *J* = 3.0 Hz, C), 128.6 (CH), {127.21, 127.16} (CH, *J* = 8.0 Hz, CH), 127.0 (CH), 126.6 (CH), {115.15, 115.00} (CH, *J* = 21.3 Hz, CH), 79.9 (CH), 75.0 (CH_2), 44.3 (CH), 42.6 (CH_2).

4.10 *cis*-2-(4-Methylphenyl)-4-phenyltetrahydrofuran ((±)-3af) and *trans*-2-(4-methylphenyl)-4-phenyltetrahydrofuran ((±)-4af)

According to General Procedure 1, styrene oxide ((±)-**1a**; 120 mg, 114 µL, 1.0 mmol, 1.00 equiv) was treated with 4-methylstyrene (**2f**; 473 mg, 527 µL, 4.0 mmol, 4.00 equiv), Fe(TPP)Cl (21 mg, 30 µmol, 3 mol%), Zn dust (92 mg, 1.4 mmol, 1.40 equiv) and Et_3N (30.4 mg, ca. 42 µL, 0.3 mmol, 0.30 equiv) in anhydrous MeCN (0.5 mL), at 60 °C for 16 h under N_2. The crude product was purified by flash column chromatography on silica gel to afford (±)-**3af** and (±)-**4af** as a diasteromeric mixture (210 mg, 0.88 mmol, 88%), as a colorless and slightly viscous liquid. The *cis/trans* diastereomeric ratio ((±)-**3af** : (±)-**4af**) was determined to be 68:32 by ^{1}H NMR spectroscopy.

TLC: R_f = 0.57 (silica gel; *n*-hexane/CH_2Cl_2, 1:1, v/v) (±)-**3af** and (±)-**4af**

R_f = 0.28 (silica gel; *n*-hexane/EtOAc, 10:0.4, v/v) (±)-**3af**

R_f = 0.31 (silica gel; *n*-hexane/EtOAc, 10:0.4, v/v) (±)-**4af**

FTIR (KBr): $\tilde{\nu}_{max}$ (cm^{-1}) = 3060 (m), 3028 (s), 2970 (s), 2924 (s), 2863 (s), 1948 (w), 1902 (w), 1805 (w), 1716 (s), 1603 (m), 1515 (s), 1495 (s), 1454 (s), 1360 (m), 1307 (m), 1273 (s), 1208 (w), 1178 (m), 1110 (s), 1067 (s), 1021 (s), 998 (m), 922 (m), 842 (w), 815 (s), 756 (s), 700 (s), 655 (w), 565 (w), 532 (m).

GCMS: t_R = 31.30 min ((±)-**3af**), t_R = 31.43 min ((±)-**4af**); *m/z* (%) = 238 ($[M]^+$, 55), 223 ($[M-15]^+$, 14), 208 ($[M-30]^+$, 55), 193 ($[M-45]^+$, 100), 178 (20), 147 (26), 117 (91), 105 (35), 91 (64), 77 (17).

HRMS (ESI): *m/z* calcd for $[C_{17}H_{18}NaO]([M+Na]^+)$: 261.1255; found: 261.1246.

(±)-**3af**

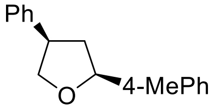

^{1}H NMR (600 MHz, $CDCl_3$): δ = 7.33–7.25 (m, 5H), 7.23–7.19 (m, 2H), 7.16 (d, *J* = 8.0 Hz, 2H), 5.02 (dd, *J* = 10.2 Hz, *J* = 5.7 Hz, 1H), 4.33 (t, *J* = 8.3 Hz, 1H), 4.00 (t, *J* = 8.5 Hz, 1H), 3.64–3.58 (m, 1H), 2.73–2.69 (m, 1H), 2.34 (s, 3H), 2.02–1.97 (m, 1H).

^{13}C NMR (APT, 150 MHz, $CDCl_3$): δ = 141.8 (C), 139.5 (C), 137.0 (C), 129.5 (CH), 129.0 (CH), 128.5 (CH), 127.2 (CH), 126.6 (CH), 125.7 (CH), 81.7 (CH), 75.0 (CH_2), 46.0 (CH), 43.7 (CH_2), 21.1 (CH_3).

(±)-**4af**

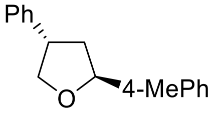

^{1}H NMR (600 MHz, $CDCl_3$): δ = 7.31–7.25 (m, 5H), 7.23–7.19 (m, 2H), 7.16 (d, *J* = 8.0 Hz, 2 H), 5.19 (t, *J* = 6.3 Hz, 1H), 4.44 (t, *J* = 7.5 Hz, 1H), 3.92 (t, *J* = 8.1 Hz, 1H), 3.55–3.49 (m, 1H), 2.46–2.41 (m, 1H), 2.37 (s, 3H), 2.32–2.29 (m, 1H).

^{13}C NMR (APT, 150 MHz, $CDCl_3$): δ = 142.1 (C), 140.5 (C), 136.7 (C), 129.0 (CH), 128.6 (CH), 128.4 (CH), 127.3 (CH), 126.5 (CH), 125.4 (CH), 80.5 (CH), 75.0 (CH_2), 44.4 (CH), 42.6 (CH_2), 21.6 (CH_3).

4.11 *cis*-2-Methyl-2,4-diphenyltetrahydrofuran ((±)-3ag) and *trans*-2-methyl-2,4-diphenyltetrahydrofuran ((±)-4ag)

According to General Procedure 1, styrene oxide ((±)-**1a**; 120 mg, 114 µL, 1.0 mmol, 1.00 equiv) was treated with α-methylstyrene (**2g**; 473 mg, ca. 520 µL, 4.0 mmol, 4.00 equiv), Fe(TPP)Cl (21 mg, 30 µmol, 3 mol%), Zn dust (92 mg, 1.4 mmol, 1.40 equiv) and Et_3N (30.4 mg, ca. 42 µL, 0.3 mmol, 0.30 equiv) in anhydrous MeCN (0.5 mL), at 60 °C for 16 h under N_2. The crude product was purified by flash column chromatography on silica gel to afford (±)-**3ag** and (±)-**4ag** as a diasteromeric mixture (198 mg, 0.83 mmol, 83%), as a colorless and slightly viscous liquid. The *cis/trans* diastereomeric ratio ((±)-**3ag** : (±)-**4ag**) was determined to be 58:42 by ^{1}H NMR spectroscopy.

TLC: R_f = 0.62 (silica gel; *n*-hexane/CH_2Cl_2, 1:1, v/v)

R_f = 0.40 (silica gel; *n*-hexane/EtOAc, 10:0.4, v/v)

FTIR (KBr): $\tilde{\nu}_{max}$ (cm^{-1}) = 3084 (m), 3060 (m), 3027 (m), 2971 (s), 2927 (m), 2865 (m), 1950 (w), 1882 (w), 1808 (w), 1602 (m), 1583 (w), 1494 (s), 1445 (s), 1370 (w), 1310 (m), 1272 (m), 1204 (m), 1124 (s), 1096 (w), 1069 (s), 1047 (s), 908 (w), 850 (w), 764 (s), 700 (s), 655 (w), 562 (m), 534 (m).

GCMS: t_R = 29.75 min ((±)-**3ag**), t_R = 29.82 min ((±)-**4ag**); *m/z* (%) = 223 ($[M]^+$, 83), 193 ($[M-30]^+$, 9), 117 (28), 105 (100), 91 (19), 77 (22), 43 (7).

HRMS (ESI): *m/z* calcd for $[C_{17}H_{18}NaO]([M+Na]^+)$: 261.1358; found: 261.1268.

(±)-**3ag**

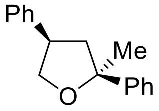

^{1}H NMR (500 MHz, $CDCl_3$): δ = 7.46–7.45 (m, 2H), 7.37–7.33 (m, 2H), 7.30–7.14 (m, 6H), 4.38 (t, *J* = 7.9 Hz, 1H), 3.81 (dd, *J* = 10.0 Hz, *J* = 8.4 Hz, 1H), 3.71–3.63 (m, 1H), 2.63 (dd, *J* = 12.4 Hz, *J* = 8.0 Hz, 1H), 2.30 (dd, *J* = 12.4 Hz, *J* = 10.7 Hz, 1H), 1.60 (s, 3H).

^{13}C NMR (APT, 125 MHz, $CDCl_3$): δ = 148.9 (C), 140.8 (C), 128.5 (CH), 128.3 (CH), 127.4 (CH), 126.6 (CH), 126.4 (CH), 124.5 (CH), 84.9 (C), 74.4 (CH_2), 48.0 (CH_2), 45.8 (CH), 30.6 (CH_3).

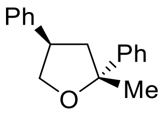

(±)-**4ag**

^{1}H NMR (500 MHz, $CDCl_3$): δ = 7.46–7.45 (m, 2H), 7.38–7.33 (m, 2H), 7.30–7.14 (m, 6H), 4.32 (t, J = 8.4 Hz, 1H), 3.97 (t, J = 8.7 Hz, 1H), 3.32–3.24 (m, 1H), 2.71 (dd, J = 12.2 Hz, J = 7.1 Hz, 1H), 2.18 (t, J = 11.6 Hz, 1H), 1.65 (s, 3H).

^{13}C NMR (APT, 125 MHz, $CDCl_3$): δ = 147.6 (C), 141.6 (C), 128.5 (CH), 128.2 (CH), 127.3 (CH), 126.5 (CH), 126.4 (CH), 124.7 (CH), 85.4 (C), 73.9 (CH_2), 48.2 (CH_2), 44.6 (CH), 30.1 (CH_3).

4.12 2,2,4-Triphenyltetrahydrofuran ((±)-3ah)

According to General Procedure 1, styrene oxide ((±)-**1a**; 120 mg, 114 µL, 1.0 mmol, 1.00 equiv) was treated with 1,1-diphenylethylene (**2h**; 721 mg, 706 µL, 4.0 mmol, 4.00 equiv), Fe(TPP)Cl (21 mg, 30 µmol, 3 mol%), Zn dust (92 mg, 1.4 mmol, 1.40 equiv) and Et_3N (30.4 mg, ca. 42 µL, 0.3 mmol, 0.30 equiv) in anhydrous MeCN (0.5 mL), at 60 °C for 16 h under N_2. The crude product was purified by flash column chromatography on silica gel to afford (±)-**3ah** (252 mg, 0.84 mmol, 84%) as a colorless solid.

(±)-**3ah**

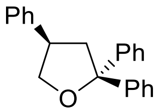

Mp: 68-69 °C.

TLC: R_f = 0.69 (silica gel; *n*-hexane/CH_2Cl_2, 1:1, v/v)

R_f = 0.44 (silica gel; *n*-hexane/EtOAc, 10:0.4, v/v)

FTIR (KBr): $\tilde{\nu}_{max}$ (cm^{-1}) = 3434 (br), 3056 (m), 3026 (s), 2923 (m), 2853 (m), 2311 (w), 1946 (w), 1871 (w), 1804 (w), 1599 (m), 1583 (m), 1490 (s), 1445 (s), 1331 (w), 1313 (w), 1249 (m), 1218 (m), 1061 (s), 1038 (s), 999 (m), 952 (m), 841 (w), 779 (s), 753 (s), 732 (s), 695 (s), 614 (m), 547 (m), 535 (m).

GCMS: t_R = 34.97 min ((±)-**3ah**); *m/z* (%) = 300 ($[M]^+$, 38), 270 ($[M-30]^+$, 11), 223 (78), 192 (19), 105 (100), 91 (16), 77 (32).

HRMS (ESI): *m/z* calcd for $[C_{22}H_{20}NaO]([M+Na]^+)$: 323.1514; found: 323.1518.

^{1}H NMR (500 MHz, $CDCl_3$): δ = 7.51-7.45 (m, 4H), 7.34–7.18 (m, 11H), 4.41 (t, *J* = 8.4 Hz, 1H), 4.01 (t, *J* = 8.7 Hz, 1H), 3.48–3.41 (m, 1H), 3.17 (dd, *J* = 12.3 Hz, *J* = 7.1 Hz, 1H), 2.62 (t, *J* = 11.9 Hz, 1H).

^{13}C NMR (APT, 125 MHz, $CDCl_3$): δ = 146.6 (C), 146.0 (C), 141.2 (C), 128.5 (CH), 128.3 (CH), 128.2 (CH), 127.3 (CH), 126.8 (CH), 126.7 (CH), 126.6 (CH), 125.7 (CH), 125.6 (CH), 88.8 (C), 74.2 (CH_2), 47.3 (CH_2), 44.8 (CH).

4.13 (4*R*)-2,2,4-Triphenyltetrahydrofuran ((4*R*)-3ah)

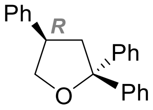

(4*R*)-**3ah**

According to General Procedure 2, styrene oxide ((±)-**1a**; 120 mg, 114 µL, 1.0 mmol, 1.00 equiv) was treated with 1,1-diphenylethylene (**2h**; 721 mg, 706 µL, 4.0 mmol, 4.00 equiv), **32** (81 mg, 100 µmol, 10 mol%), Zn dust (92 mg, 1.4 mmol, 1.40 equiv) and Et_3N (30.4 mg, ca. 42 µL, 0.3 mmol, 0.30 equiv) in anhydrous MeCN (0.5 mL), at 60 °C for 16 h under N_2. The crude product was purified by flash column chromatography on silica gel to afford (4*R*)-**3ah** (228 mg, 0.76 mmol, 76%, 12% *ee*), as a colorless solid.

*The proposed absolute configuration of **3ah** was not supported by any chiroptical technique. The absolute configuration of the major enantiomer was deduced from the stereochemical outcome of the reaction of Entry 10 in Table 4 and the stereochemical outcomes of the reactions in Scheme 2 (the Equations 1–3). According to HPLC analyses, all those reactions gave the same enantiomer in excess. After the reactions in Equations 2 and 3 (Scheme 2), the absolute configuration of (R)-styrene oxide is expected to remain unchanged.*

HPLC: Column: Chiralcel AD-H, 25 cm, 0.46 cm ID; Eluent: *n*-hexane/*i*-PrOH (95:5); Flow rate: 0.5 mL/min; Pressure: 42 bar; Detection: 254 nm.

t_R = 9.6 min ((4*R*)-**3ah**, the major enantiomer of **3ah**)

t_R = 10.2 min ((4*S*)-**3ah**, the minor enantiomer of **3ah**)

4.14 *cis*-3-Phenyl-3,3a,4,8b-tetrahydro-2*H*-indeno[1,2-*b*]furan ((±)-3ai) and *trans*-3-phenyl-3,3a,4,8b-tetrahydro-2*H*-indeno[1,2-*b*]furan ((±)-4ai)

According to General Procedure 1, styrene oxide ((±)-**1a**; 120 mg, 114 µL, 1.0 mmol, 1.00 equiv) was treated with indene (**2i**; 465 mg, ca. 470 µL, 4.0 mmol, 4.00 equiv), Fe(TPP)Cl (21 mg, 30 µmol, 3 mol%), Zn dust (92 mg, 1.4 mmol, 1.40 equiv) and Et_3N (30.4 mg, ca. 42 µL, 0.3 mmol, 0.30 equiv) in anhydrous MeCN (0.5 mL), at 60 °C for 16 h under N_2. The crude product was purified by flash column chromatography on silica gel to afford (±)-**3ai** and (±)-**4ai** as a diasteromeric mixture (161 mg, 0.68 mmol, 68%), as a colorless and slightly viscous liquid. The *cis/trans* diastereomeric ratio ((±)-**3ai** : (±)-**4ai**) was determined to be 54:46 by ^{1}H NMR spectroscopy.

TLC: R_f = 0.39 (silica gel; *n*-hexane/CH_2Cl_2, 1:1, v/v) (±)-**3ai**

R_f = 0.43 (silica gel; *n*-hexane/CH_2Cl_2, 1:1, v/v) (±)-**4ai**

FTIR (KBr): $\tilde{\nu}_{max}$ (cm^{-1}) = 3650 (w), 3276 (br), 3062 (m), 3027 (s), 2941 (s), 2850 (s), 1951 (w), 1875 (w), 1807 (w), 1711 (w), 1603 (m), 1586 (w), 1496 (s), 1480 (s), 1458 (s), 1326 (m), 1211 (w), 1176 (m), 1095 (m), 1061 (s), 1041 (s), 987 (m), 938 (m), 845 (w), 751 (s), 699 (s), 545 (m), 511 (w).

GCMS: t_R = 31.33 min ((±)-**3ai**), t_R = 31.04 min ((±)-**4ai**); *m/z* (%) = 236 ([M]$^+$, 33), 206 ([M−30]$^+$, 100), 128 (21), 115 (30), 91 (87).

HRMS (ESI): *m/z* calcd for [$C_{17}H_{16}NaO$]([M+Na]$^+$): 259.12; found: 259.1112.

(±)-**3ai** 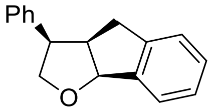

^{1}H NMR (500 MHz, CDCl$_3$): δ = 7.48–7.45 (m, 1H), 7.35–7.21 (m, 8H), 5.69 (d, *J* = 7.1 Hz, 1H), 4.09 (dd, *J* = 8.5 Hz, *J* = 6.8 Hz, 1H), 3.89 (t, *J* = 8.4 Hz, 1H), 3.21–3.14 (m, 2H), 3.05–3.01 (m, 1H), 2.95–2.90 (m, 1H).

^{13}C NMR (APT, 125 MHz, CDCl$_3$): δ = 142.4 (C), 141.9 (C), 141.7 (C), 128.6 (CH), 128.3 (CH), 127.4 (CH), 127.1 (CH), 126.7 (CH), 125.5 (CH), 125.5 (CH), 87.7 (CH), 74.6 (CH_2), 53.3 (CH), 50.0 (CH), 36.5 (CH_2).

(±)-**4ai** 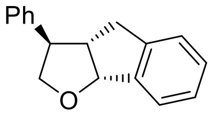

^{1}H NMR (500 MHz, CDCl$_3$): δ = 7.48–7.45 (m, 1H), 7.35–7.21 (m, 5H), 7.09–7.05 (m, 3H), 5.65 (d, *J* = 6.8 Hz, 1H), 4.23–4.17 (m, 1H), 3.76–3.70 (m, 2H), 3.51–3.46 (m, 1H), 2.79 (dd, *J* = 17.3 Hz, *J* = 9.4 Hz, 1H), 2.57 (dd, *J* = 17.3 Hz, *J* = 4.7 Hz 1H).

^{13}C NMR (APT, 125 MHz, CDCl$_3$): δ = 143.9 (C), 141.8 (C), 139.0 (C), 128.7 (CH), 128.6 (CH), 128.3 (CH), 126.9 (CH), 126.4 (CH), 125.2 (CH), 124.3 (CH), 87.9 (CH), 69.4 (CH_2), 48.5 (CH), 45.5 (CH), 33.2 (CH_2).

4.15 (±)-*trans*:*cis*-2,3,5-Triphenyltetrahydrofuran ((±)-3ba) and (±)-*trans*:*trans*-2,3,5-triphenyltetrahydrofuran ((±)-4ba)

According to General Procedure 1, stilbene oxide ((±)-**1b**; 196 mg, 1.0 mmol, 1.00 equiv) was treated with styrene (**2a**; 417 mg, ca. 460 µL, 4.0 mmol, 4.00 equiv), Fe(TPP)Cl (21 mg, 30 µmol, 3 mol%), Zn dust (92 mg, 1.4 mmol, 1.40 equiv) and Et_3N (30.4 mg, ca. 42 µL, 0.3 mmol, 0.30 equiv) in anhydrous MeCN (0.5 mL), at 60 °C for 16 h under N_2. The crude product was purified by flash column chromatography on silica gel to afford (±)-**3ba** and (±)-**4ba** as a diasteromeric mixture (210 mg, 0.70 mmol, 70%), as a colorless and slightly viscous liquid. The diastereomeric ratio ((±)-**3ba** : (±)-**4ba**) was determined to be 58:42 by ^{1}H NMR spectroscopy.

TLC: R_f = 0.66 (silica gel; *n*-hexane/CH_2Cl_2, 1:1, v/v) (±)-**3ba** and (±)-**4ba**

FTIR (KBr): $\tilde{\nu}_{max}$ (cm^{-1}) = 3382 (br), 3087 (m), 3062 (s), 3030 (s), 2876 (m), 1951 (w), 1880 (w), 1809 (w), 1720 (s), 1667 (m), 1602 (m), 1584 (w), 1495 (s), 1451 (s), 1315 (m), 1271 (s), 1211 (m), 1176 (m), 1110 (m), 1083 (m), 1054 (s), 1026 (s), 911 (m), 848 (w), 756 (s), 699 (s), 645 (w), 618 (w), 596 (w), 521 (w).

GCMS: t_R = 35.33 min ((±)-**3ba**), t_R = 34.43 min ((±)-**4ba**); *m/z* (%) = 194 ([M−106]$^+$, 100), 179 (29), 165 (8), 115 (22), 91 (9), 77 (8).

HRMS (ESI): *m/z* calcd for [$C_{22}H_{20}NaO$]([M+Na]$^+$): 323.1412; found: 323.1418.

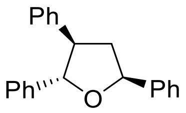

(±)-**3ba**

^{1}H NMR (600 MHz, CDCl$_3$): δ = 7.52 (d, J = 7.4, 2H), 7.45–7.38 (m, 2H), 7.31-7.28 (m, 4H), 7.24-7.20 (m, 3H), 7.05-6.98 (m, 3H), 6.86-6.83 (m, 1H), 5.37 (dd, J = 6.3 Hz, J = 1.2 Hz, 1H), 5.00 (d, J = 8.7 Hz, 1H), 3.36 (q, J = 8.7 Hz, 1H), 2.72–2.67 (m, 1H), 2.54–2.49 (m, 1H).

^{13}C NMR (APT, 150 MHz, CDCl$_3$): δ = 143.1 (C), 141.0 (C), 139.9 (C), 128.6 (CH), 128.4 (CH), 128.2 (CH), 127.6 (CH), 127.3 (CH), 126.6 (CH), 126.2 (CH), 125.9 (CH), 88.4 (CH), 80.3 (CH), 53.4 (CH), 43.6 (CH$_2$).

1D-NOE-NMR of (±)-**3ba:**

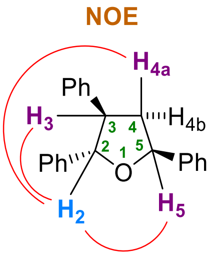

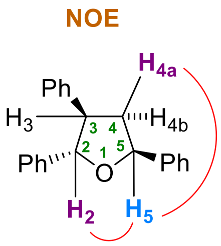

C#	δ_C	δ_H	Mult.	COSY	NOE-DIFF
**1**	-	-	-	-	-
**2**	88.4	5.00 (d, J = 8.7 Hz, 1H) (*H*-2)	CH	*H*-3	*H*-5, *H*-4a, *H*-3
**3**	53.4	3.36 (dd, J = 17.5 Hz, J = 8.7 Hz, 1H) (*H*-3)	CH	*H*-2, *H*-4a	-
**4**	43.6	2.72–2.67 (m, 1H) (*H*-4a)<br>2.54–2.49 (m, 1H) (*H*-4b)	CH$_2$	*H*-5, *H*-4b, *H*-3<br>*H*-5, *H*-4a, *H*-3	-
**5**	80.3	5.37 (dd, J = 6.3 Hz, J = 1.2 Hz, 1H) (*H*-5)	CH	*H*-4a, *H*-4b	*H*-4a, *H*-2

(±)-**4ba** 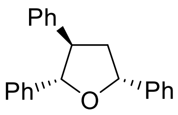

^{1}H NMR (600 MHz, $CDCl_3$): δ = 7.60 (d, J = 7.5, 2H), 7.45–7.38 (m, 3H), 7.31-7.28 (m, 4H), 7.24-7.20 (m, 3H), 7.05-6.98 (m, 3H), 5.41 (d, J = 8.3 Hz, 1H), 5.23 (dd, J = 10.0 Hz, J = 6.1 Hz, 1H), 3.97 (dd, J = 17.1 Hz, J = 8.2 Hz, 1H), 2.82–2.77 (m, 1H), 2.33–2.28 (m, 1H).

^{13}C NMR (APT, 150 MHz, $CDCl_3$): δ = 141.7 (C), 140.6 (C), 140.1 (C), 128.7 (CH), 128.4 (CH), 127.8 (CH), 127.6 (CH), 127.4 (CH), 126.7 (CH), 126.6 (CH), 126.1 (CH), 125.8 (CH), 84.4 (CH), 80.2 (CH), 50.9 (CH), 40.6 (CH_2).

1D-NOE-NMR of (±)-**4ba:**

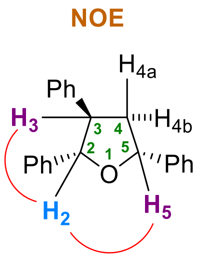

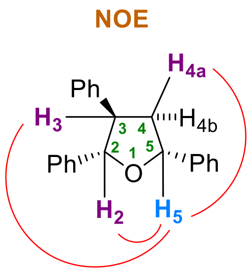

C#	δ_C	δ_H	Mult.	COSY	NOE-DIFF
**1**	-	-	-	-	-
**2**	84.4	5.41 (d, J = 8.3 Hz, 1H) (*H*-2)	CH	*H*-3	*H*-5, *H*-3
**3**	50.9	3.97 (dd, J = 17.1 Hz, J = 8.2 Hz, 1H) (*H*-3)	CH	*H*-2, *H*-4a	-
**4**	40.6	2.82–2.77 (m, 1H) (*H*-4a)<br>2.33–2.28 (m, 1H) (*H*-4b)	CH_2	*H*-5, *H*-4b, *H*-3<br>*H*-5, *H*-4a, *H*-3	-
**5**	80.2	5.23 (dd, J = 10.0 Hz, J = 6.1 Hz, 1H) (*H*-5)	CH	*H*-4a, *H*-4b	*H*-4a, *H*-3, *H*-2

4.16 (±)-*trans*:*cis*-2-Methyl-3,5-diphenyltetrahydrofuran ((±)-3ca) and (±)-*trans:trans*-2-methyl-3,5-diphenyltetrahydrofuran ((±)-4ca)

According to General Procedure 1, *trans*-β-methylstyrene oxide ((±)-**1c**; 134 mg, ca. 134 µL, 1.0 mmol, 1.00 equiv) was treated with styrene (**2a**; 417 mg, ca. 460 µL, 4.0 mmol, 4.00 equiv), Fe(TPP)Cl (21 mg, 30 µmol, 3 mol%), Zn dust (92 mg, 1.4 mmol, 1.40 equiv) and Et_3N (30.4 mg, ca. 42 µL, 0.3 mmol, 0.30 equiv) in anhydrous MeCN (0.5 mL), at 60 °C for 16 h under N_2. The crude product was purified by flash column chromatography on silica gel to afford (±)-**3ca** and (±)-**4ca** as a diasteromeric mixture (180 mg, 0.76 mmol, 76%), as a colorless and slightly viscous liquid. The diastereomeric ratio ((±)-**3ba** : (±)-**4ba**) was determined to be 69:31 by ^{1}H NMR spectroscopy.

TLC: R_f = 0.56 (silica gel; *n*-hexane/CH_2Cl_2, 1:1, v/v) (±)-**3ca** and (±)-**4ca**

FTIR (KBr): $\tilde{\nu}_{max}$ (cm^{-1}) = 3650 (w), 3085 (w), 3062 (m), 3027 (s), 2970 (s), 2928 (m), 2867 (m), 2340 (w), 1948 (w), 1877 (w), 1807 (w), 1752 (w), 1602 (m), 1584 (w), 1543 (w), 1495 (s), 1452 (s), 1370 (m), 1343 (w), 1325 (w), 1303 (w), 1287 (w), 1239 (w), 1213 (w), 1172 (w), 1155 (w), 1135 (m), 1101 (s), 1042 (s), 1028 (s), 965 (w), 951 (w), 910 (w), 871 (w), 751 (s), 699 (s), 636 (w), 614 (w) 568 (w), 534 (w), 510 (w).

GCMS: t_R = 30.12 min ((±)-**3ca**), t_R = 30.24 min ((±)-**4ca**); *m/z* (%) = 194 ($[M-44]^+$, 100), 179 (36), 165 (7), 115 (28), 105 (11), 91 (14), 77 (12).

HRMS (ESI): *m/z* calcd for $[C_{17}H_{18}NaO]([M+Na]^+)$: 261.1255; found: 261.1282.

(±)-**3ca** 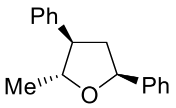

^{1}H NMR (600 MHz, $CDCl_3$): δ = 7.41–7.40 (m, 2H), 7.38–7.29 (m, 4), 7.28–7.18 (m, 4H), 5.20 (dd, J = 8.2 Hz, J = 5.9 Hz, 1H), 4.11–4.07 (m, 1H), 2.95 (q, J = 8.9 Hz, 1H), 2.59–2.54 (m, 1H), 2.37–2.33 (m, 1H), 1.36 (d, J = 6.0 Hz, 3H).

^{13}C NMR (APT, 150 MHz, $CDCl_3$): δ = 143.6 (C), 141.3 (C), 128.6 (CH), 128.3 (CH), 127.6 (CH), 127.2 (CH), 126.7 (CH), 125.7, 82.9 (CH), 79.9 (CH), 52.2 (CH), 43.7 (CH_2), 19.0 (CH_3).

1D-NOE-NMR of (±)-**3ca:**

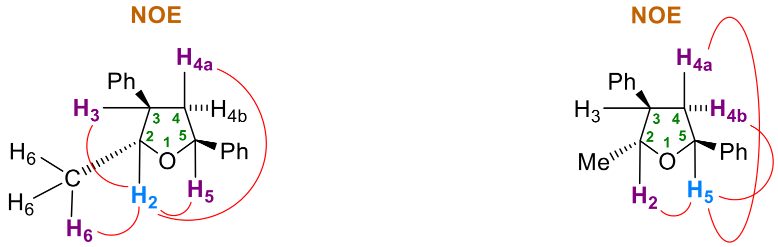

C#	δ_C	δ_H	Mult.	COSY	NOE-DIFF
**1**	-	-	-	-	-
**2**	82.9	4.11–4.07 (m, 1H) (*H*-2)	CH	*H*-6, *H*-3	*H*-6, *H*-5, *H*-4a, *H*-3
**3**	52.2	2.95 (q, J = 8.9 Hz, 1H) (*H*-3)	CH	*H*-4a, *H*-4b, *H*-2,	-
**4**	43.7	2.59–2.54 (m, 1H) (*H*-4a)<br>2.37–2.33 (m, 1H) (*H*-4b)	CH_2	*H*-5, *H*-4b, *H*-3<br>*H*-5, *H*-4a, *H*-3	-
**5**	79.9	5.20 (dd, J = 8.2, 5.9 Hz, 1H) (*H*-5)	CH	*H*-4a, *H*-4b	*H*-4a, *H*-4b, *H*-2
**6**	19.0	1.36 (d, J = 6.0 Hz, 3H) (*H*-6)	CH_3	*H*-2	

(±)-**4ca** 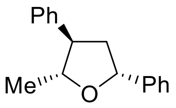

^{1}H NMR (600 MHz, $CDCl_3$): δ = 7.46–7.45 (m, 2H), 7.38–7.29 (m, 4), 7.28–7.18 (m, 4H), 5.01 (dd, J = 10.0 Hz, J = 6.2 Hz, 1H), 4.49–4.44 (m, 1H), 3.67 (dd, J = 17.1 Hz, J = 7.8 Hz, 1H), 2,70–2.65 (m, 1H), 2.19–2.14 (m, 1H), 0.93 (d, J = 6.4 Hz, 3H).

^{13}C NMR (APT, 150 MHz, $CDCl_3$): δ = 142.4 (C), 140.8 (C), 128.4 (CH), 128.3 (CH), 128.2 (CH), 127.3 (CH), 126.4 (CH), 125.7, 80.4 (CH), 78.3 (CH), 48.8 (CH), 40.7 (CH_2), 18.2 (CH_3).

1D-NOE-NMR of (±)-**4ca:**

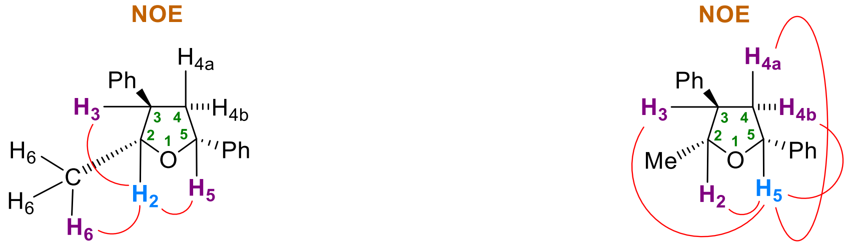

C#	δ_C	δ_H	Mult.	COSY	NOE-DIFF
**1**	-	-	-	-	-
**2**	78.3	4.49–4.44 (m, 1H) (*H*-2)	CH	*H*-6, *H*-3	*H*-6, *H*-5, *H*-3
**3**	48.8	3.67 (dd, J = 17.1, 7.8 Hz, 1H) (*H*-3)	CH	*H*-4a, *H*-4b, *H*-2,	-
**4**	40.7	2.70–2.65 (m, 1H) (*H*-4a)<br>2.19–2.14 (m, 1H) (*H*-4b)	CH_2	*H*-5, *H*-4b, *H*-3<br>*H*-5, *H*-4a, *H*-3	-
**5**	80.4	5.01 (dd, J = 10.0, 6.2 Hz, 1H) (*H*-5)	CH	*H*-4a, *H*-4b	*H*-4a, *H*-4b, *H*-3, *H*-2
**6**	18.2	0.93 (d, J = 6.4 Hz, 3H) (*H*-6)	CH_3	*H*-2	

4.17 (±)-*trans*:*trans*-2,4-Diphenyltetrahydrofuran-3-yl-phenylmethanone ((±)-3aj), (±)-*trans*:*cis*-2,4-Diphenyltetrahydrofuran-3-yl-phenylmethanone ((±)-4aj), (±)-*cis*:*cis*-2,4-Diphenyltetrahydrofuran-3-yl-phenylmethanone ((±)-3'aj) and (±)-*cis*:*trans*-2,4-Diphenyltetrahydrofuran-3-yl-phenylmethanone ((±)-4'aj)

According to General Procedure 1, styrene oxide ((±)-**1a**; 120 mg, 114 µL, 1.0 mmol, 1.00 equiv) was treated with *trans*-chalcone (**2j**; 833 mg, 4.0 mmol, 4.00 equiv), Fe(TPP)Cl (21 mg, 30 µmol, 3 mol%), Zn dust (92 mg, 1.4 mmol, 1.40 equiv) and Et_3N (30.4 mg, ca. 42 µL, 0.3 mmol, 0.30 equiv) in anhydrous MeCN (0.5 mL), at 60 °C for 16 h under N_2. The crude product was purified by flash column chromatography on silica gel, affording (±)-**3aj**, (±)-**4aj**, (±)-**3'aj** and (±)-**4'aj** in 78% overall yield (256 mg, 0.78 mmol) as a colorless solid. Thus, (±)-**3aj** together with (±)-**4aj** as well as (±)-**3'aj** together with (±)-**4'aj** could be isolated as mixtures of diastereomers. The diastereomeric ratio ((±)-**3aj**(±)-**4aj**) : ((±)-**3'aj** : (±)-**4'aj**) was determined to be 59:41 by column chromatography. The diastereomeric ratio (±)-**3aj** : (±)-**4aj** was determined to be 57:43 and the diastereomeric ratio (±)-**3'aj** : (±)-**4'aj** was determined to be 70:30 by ^{1}H NMR spectroscopy.

TLC: R_f = 0.27 (silica gel; *n*-hexane/CH_2Cl_2, 1:1, v/v) (±)-**3aj** and (±)-**4aj**

R_f = 0.18 (silica gel; *n*-hexane/CH_2Cl_2, 1:1, v/v) (±)-**3'aj** and (±)-**4'aj**

FTIR (KBr): $\tilde{\nu}_{max}$ (cm^{-1}) = 3372 (w), 3084 (m), 3060 (m), 3028 (m), 2943 (s), 2886 (s), 1960 (w), 1898 (w), 1811 (w), 1697 (s), 1672 (s), 1594 (s), 1579 (m), 1492 (s), 1447 (s), 1388 (m), 1371 (m), 1356 (m), 1308 (m), 1280 (m), 1220 (s), 1175 (m), 1157 (m), 1085 (s), 1058 (s), 1004 (s), 922 (m), 874 (m), 844 (m), 754 (s), 699 (s), 665 (s), 650 (s), 617 (w), 589 (w), 555 (m), 539 (m), 515 (m), 494 (w), 466 (w).

GCMS: t_R = 36.23 min ((±)-**3aj** and (±)-**4aj**), t_R = 36.91 min, t_R = 36.98 min ((±)-**3'aj** and (±)-**4'aj**); *m/z* (%) = 223 ([M-105]$^+$, 100), 207 ([M−121]$^+$, 17), 147 (17), 116 (16), 105 (66), 91 (12), 77 (36).

HRMS (ESI): *m/z* calcd for [$C_{23}H_{20}NaO_2$]([M+Na]$^+$): 351.1361; found: 351.1360.

(±)-**3aj**

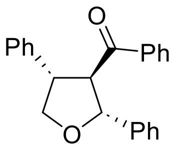

^{1}H NMR (600 MHz, CDCl$_3$): δ = 7.65–7.63 (m, 1H), 7.49–7.39 (m, 3H), 7.36-7.18 (m, 9H), 7.11-7.06 (m, 1H), 7.03-7.01 (m, 1H), 5.35 (d, *J* = 8.6 Hz, 1H), 4.49 (dd, *J* = 8.9 Hz, *J* = 8.1 Hz, 1H), 4.35-4.31 (m, 1H), 4.08 (t, *J* = 8.3 Hz, 1H), 3.91 (td, *J* = 8.0 Hz, *J* = 5.9 Hz, 1H).

^{13}C NMR (APT, 150 MHz, CDCl$_3$): δ = 198.1 (C), 142.1 (C), 140.6 (C), 137.0 (C), 133.4 (CH), 129.0 (CH), 128.7 (CH), 128.6 (CH), 128.5 (CH), 128.5 (CH), 128.2 (CH), 128.0 (CH), 127.7 (CH), 127.5 (CH), 127.2 (CH), 126.0 (CH), 85.6 (CH), 75.6 (CH_2), 64.3 (CH), 52.2 (CH).

1D-NOE-NMR of (±)-**3aj:**

C#	δ_C	δ_H	Mult.	COSY	NOE-DIFF
**1**	-	-	-	-	-
**2**	85.6	5.35 (d, *J* = 8.6 Hz, 1H) (*H*-2)	CH	*H*-3	*H*-3, *H*-4, *H*-5b
**3**	64.3	4.08 (t, *J* = 8.3 Hz, 1H) (*H*-3)	CH	*H*-2, *H*-4	*H*-2, *H*-4
**4**	52.2	3.91 (td, *J* = 8.0 Hz, *J* = 5.9 Hz, 1H) (*H*-4)	CH	*H*-3, *H*-5a, *H*-5b	*H*-2, *H*-3, *H*-5a, *H*-5b
**5**	75.6	4.49 (dd, *J* = 8.9 Hz, *J* = 8.1 Hz, 1H) (*H*-5b)<br>4.35-4.31 (m, 1H) (*H*-5a)	CH_2	*H*-4, *H*-5a<br>*H*-4, *H*-5b	-

(±)-**4aj** 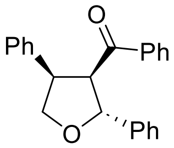

^{1}H NMR (600 MHz, CDCl$_3$): δ = 7.65–7.63 (m, 1H), 7.49–7.39 (m, 3H), 7.36-7.18 (m, 9H), 7.11-7.06 (m, 1H), 7.03-7.01 (m, 1H), 5.81 (d, J = 8.0 Hz, 1H), 4.64 (dd, J = 8.9 Hz, J = 6.6 Hz, 1H), 4.35-4.31 (m, 1H), 4.28 (dd, J = 9.3 Hz, J = 8.0 Hz, 1H), 4.03 (ddd, J = 9.3 Hz, J = 6.6 Hz, J = 5.5 Hz,1H).

^{13}C NMR (APT, 150 MHz, CDCl$_3$): δ = 198.8 (C), 142.3 (C), 138.5 (C), 137.6 (C), 133.0 (CH), 128.7 (CH), 128.6 (CH), 128.6 (CH), 128.5 (CH), 128.5 (CH), 128.4 (CH), 128.0 (CH), 127.7 (CH), 127.5 (CH), 127.2 CH), 125.9 (CH), 81.7 (CH), 75.2 (CH$_2$), 61.0 (CH), 50.7 (CH).

1D-NOE-NMR of (±)-**4aj:**

C#	δ_C	δ_H	Mult.	COSY	NOE-DIFF
**1**	-	-	-	-	-
**2**	81.7	5.81 (d, J = 8.0 Hz, 1H) (H-2)	CH	H-3	-
**3**	61.0	4.28 (dd, J = 9.3 Hz, J = 8.0 Hz, 1H) (H-3)	CH	H-2, H-4	H-4
**4**	50.7	4.03 (ddd, J = 9.3 Hz, J = 6.6 Hz, J = 5.5 Hz,1H) (H-4)	CH	H-3, H-5a, H-5b	H-3, H-5a, H-5b
**5**	75.2	4.64 (dd, J = 8.9 Hz, J = 6.6 Hz, 1H) (H-5b)<br>4.35-4.31 (m, 1H) (H-5a)	CH$_2$	H-4, H-5a<br>H-4, H-5b	-

(±)-**3'aj** 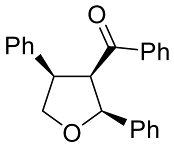

^{1}H NMR (600 MHz, CDCl$_3$): δ = 7.97–7.95 (m, 2H), 7.56–7.53 (m, 1H), 7.42 (t, J = 7.8 Hz, 2H), 7.27-7.15 (m, 6H), 7.09-7.06 (m, 2H), 6.99-6.97 (m, 2H), 5.70 (d, J = 3.5 Hz, 1H), 4.56–4.52 (m, 1H), 4.41 (t, J = 8.5 Hz, 1H), 3.93 (dd, J = 7.3 Hz, J = 3.5 Hz, 1H), 3.83 (dd, J = 16.0 Hz, J = 8.0 Hz, 1H).

^{13}C NMR (APT, 150 MHz, CDCl$_3$): δ = 198.1 (C), 138.3 (C), 137.0 (C), 134.9 (C), 133.6 (CH), 129.0 (CH), 129.0 (CH), 128.8 (CH), 128.8 (CH), 128.3 (CH), 128.0 (CH), 127.8 (CH), 127.1 (CH), 126.8 (CH), 85.7 (CH), 72.8 (CH$_2$), 53.5 (CH), 49.7 (CH).

1D-NOE-NMR of (±)-**3'aj:**

C#	δ_C	δ_H	Mult.	COSY	NOE-DIFF
**1**	-	-	-	-	-
**2**	85.7	5.70 (d, J = 3.5 Hz, 1H) (H-2)	CH	H-3	H-3, H-5b
**3**	53.5	3.93 (dd, J = 7.3 Hz, J = 3.5 Hz, 1H) (H-3)	CH	H-2, H-4	H-2, H-4 H-5a, H-5b
**4**	49.7	3.83 (dd, J = 16.0 Hz, J = 8.0 Hz, 1H) (H-4)	CH	H-3, H-5a, H-5b	H-2, H-3, H-5a, H-5b
**5**	72.8	4.56–4.52 (m, 1H) (H-5a)<br>4.41 (t, J = 8.5 Hz, 1H) (H-5b)	CH$_2$	H-4, H-5b<br>H-4, H-5a	-

(±)-**4'aj** 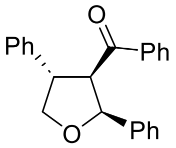

^{1}H NMR (600 MHz, $CDCl_3$): δ = δ = 7.83–7.81 (m, 2H), 7.51–7.48 (m, 1H), 7.35 (t, *J* = 7.8 Hz, 2H), 7.27-7.15 (m, 6H), 7.09-7.06 (m, 2H), 6.81-6.79 (m, 2H), 5.43 (d, *J* = 7.3 Hz, 1H), 4.56–4.52 (m, 1H), 4.18 (dd, *J* = 10.3 Hz, *J* = 8.8 Hz, 1H), 3.80 (dd, *J* = 16.0 Hz, *J* = 7.3 Hz, 1H), 3.73 (td, *J* = 10.1 Hz, *J* = 7.3 Hz, 1H).

^{13}C NMR (APT, 150 MHz, $CDCl_3$): δ = 197.8 (C), 140.4 (C), 138.5 (C), 135.2 (C), 133.6 (CH), 129.1 (CH), 129.0 (CH), 128.8 (CH), 128.8 (CH), 128.6 (CH), 128.0 (CH), 128.0 (CH), 127.2 (CH), 127.2 (CH), 87.3 (CH), 76.0 (CH_2), 56.2 (CH), 55.5 (CH).

1D-NOE-NMR of (±)-**4'aj:**

**C#**	**δ_C**	**δ_H**	**Mult.**	**COSY**	**NOE-DIFF**
**1**	-	-	-	-	-
**2**	87.3	5.43 (d, *J* = 7.3 Hz, 1H) (*H*-2)	CH	*H*-3	-
**3**	56.2	3.80 (dd, *J* = 16.0 Hz, *J* = 7.3 Hz, 1H) (*H*-3)	CH	*H*-2, *H*-4	*H*-2, *H*-5a
**4**	55.5	3.73 (td, *J* = 10.1 Hz, *J* = 7.3 Hz, 1H) (*H*-4)	CH	*H*-3, *H*-5a, *H*-5b	*H*-5a
**5**	76.0	4.56–4.52 (m, 1H) (*H*-5a)<br>4.18 (dd, *J* = 10.3 Hz, *J* = 8.8 Hz, 1H) (*H*-5b)	CH_2	*H*-4, *H*-5a<br>*H*-4, *H*-5b	-

5. References

[1] A. D. Adler, F. R. Longo, J. D. Finarelli, J. Goldmacher, J. Assour, L. Korsakoff, *J. Org. Chem.* **1967**, *32*, 476.

[2] Z. Gross, S. Ini, *J. Org. Chem.* **1997**, *62*, 5514–5521.

[3] A. Shirazi, H. M. Goff, *Inorg. Chem.* **1982**, *21*, 3420–3425.

[4] J. T. Groves, W. J. Kruper, R. C. Haushalter, W. M. Butler, *Inorg. Chem.* **1982**, *21*, 1363–1368.

[5] A. Hille, I. Ott, A. Kitanovic, I. Kitanovic, H. Alborzinia, E. Lederer, S. Wölfl, N. Metzler-Nolte, S. Schäfer, W. S. Sheldrick, C. Bischof, U. Schatzschneider, R. Gust, *J. Biol. Inorg. Chem.* **2009**, *14*, 711–725.

[6] K. Oyaizu, E. L. Dewi, E. Tsuchida, *Inorg. Chim. Acta* **2001**, *321*, 205–208.

[7] C. S. Marvel, S. A. Aspey, E. A. Dudley, *J. Am. Chem. Soc.* **1956**, *78*, 4905–4909.

[8] A. Simion, C. Simion, T. Kanda, S. Nagashima, Y. Mitoma, T. Yamada, K. Mimura, M. Tashiro, *J. Chem. Soc., Perkin Trans. 1* **2001**, 2071–2078.

[9] S. Ini, M. Kapon, S. Cohen, Z. Gross, *Tetrahedron Asymmetry* **1996**, *7*, 659–662.

[10] L. Huang, Y. Chen, G.-Y. Gao, X. P. Zhang, *J. Org. Chem.* **2003**, *68*, 8179–8184.

[11] R. L. Halterman, S.-T. Jan, H. L. Nimmons, D. J. Standlee, M. A. Khan, *Tetrahedron* **1997**, *53*, 11257–11276.

[12] Y. Chen, K. B. Fields, X. P. Zhang, *J Am Chem Soc* **2004**, *126*, 14718–14719.

[13] Y. Chen, X. P. Zhang, *J. Org. Chem.* **2007**, *72*, 5931–5934.

[14] A. Fürstner, A. Leitner, M. Méndez, H. Krause, *J Am Chem Soc* **2002**, *124*, 13856–13863.

[15] N. Takenaka, Y. Huang, V. H. Rawal, *Tetrahedron* **2002**, *58*, 8299–8305.

[16] P. Muthupandi, S. K. Alamsetti, G. Sekar, *Chemical Communications* **2009**, 3288–3290.

[17] K. P. Bryliakov, E. P. Talsi, *Chem. Eur. J.* **2007**, *13*, 8045–8050.

[18] A. D. Rodríguez, O. M. Cóbar, O. L. Padilla, *J. Nat. Prod.* **1997**, *60*, 915–917.

[19] N. D. Buezo, I. Alonso, J. C. Carretero, *J. Am. Chem. Soc.* **1998**, *120*, 7129–7130.

[20] X. Liang, K. Wei, Y.-R. Yang, *Chem. Commun.* **2015**, *51*, 17471–17474.

[21] F. Zhao, N. Li, T. Zhang, Z.-Y. Han, S.-W. Luo, L.-Z. Gong, *Angew. Chem. Int. Ed.* **2017**, *56*, 3247–3251.

[22] H. R. Lee, S. Y. Kim, M. J. Park, Y. S. Park, *Org. Biomol. Chem.* **2021**, *19*, 7655–7663.

6. NMR Spectra

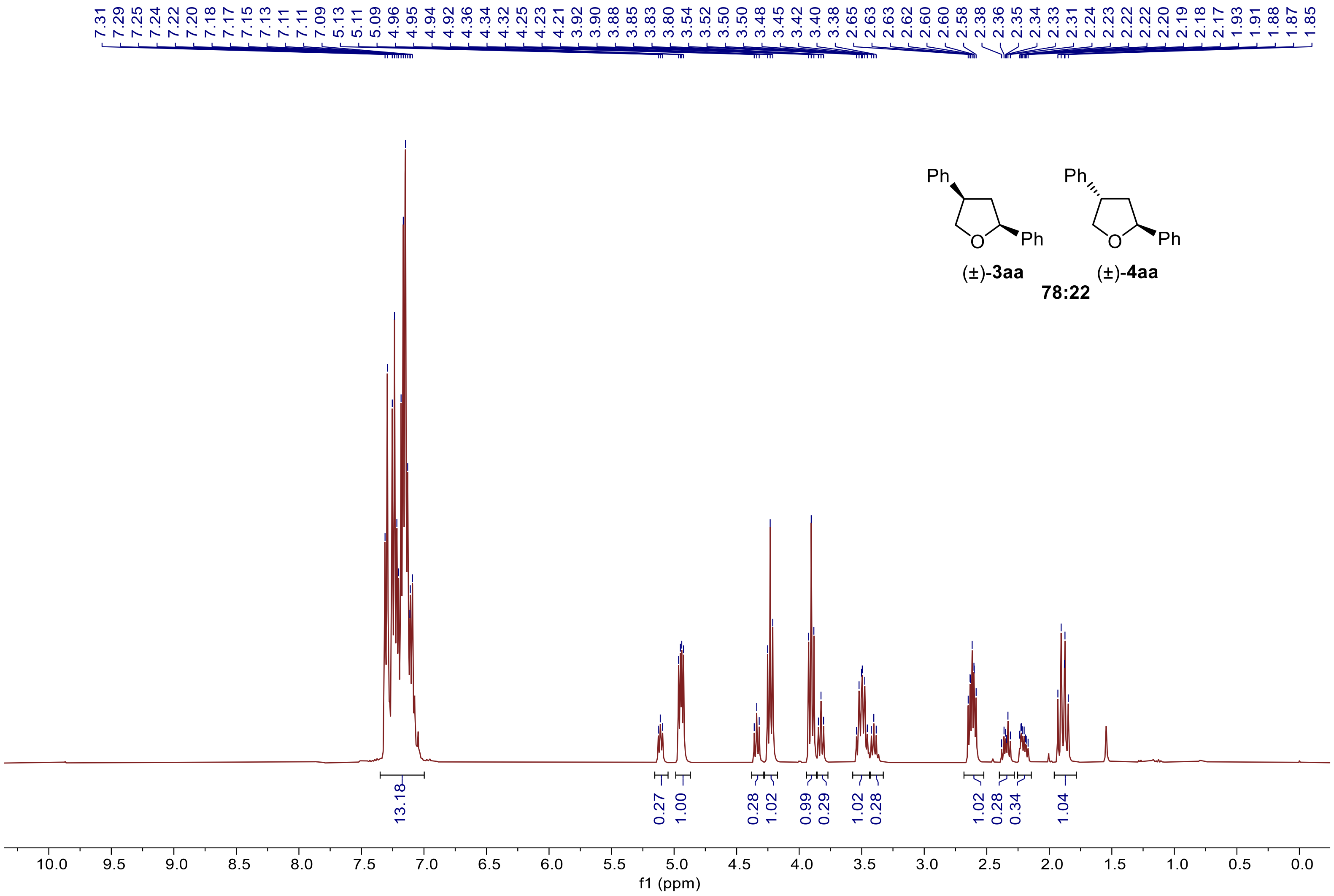

Figure S3. ^{1}H NMR Spectrum of (±)-**3aa** and (±)-**4aa** (500 MHz, $CDCl_3$).

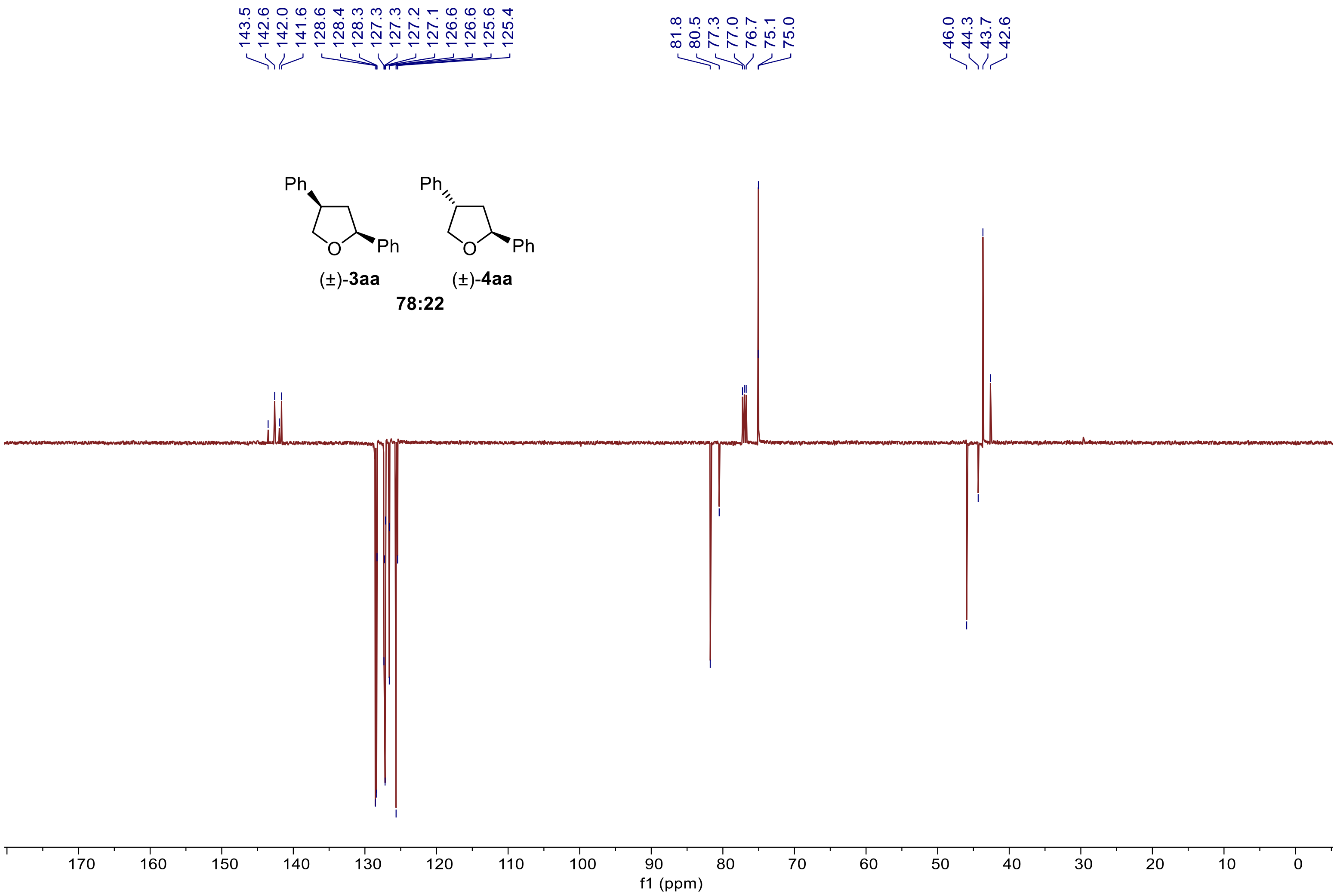

Figure S4. $^{13}C\{^{1}H\}$ NMR Spectrum of (±)-**3aa** and (±)-**4aa** (APT, 125 MHz, $CDCl_3$).

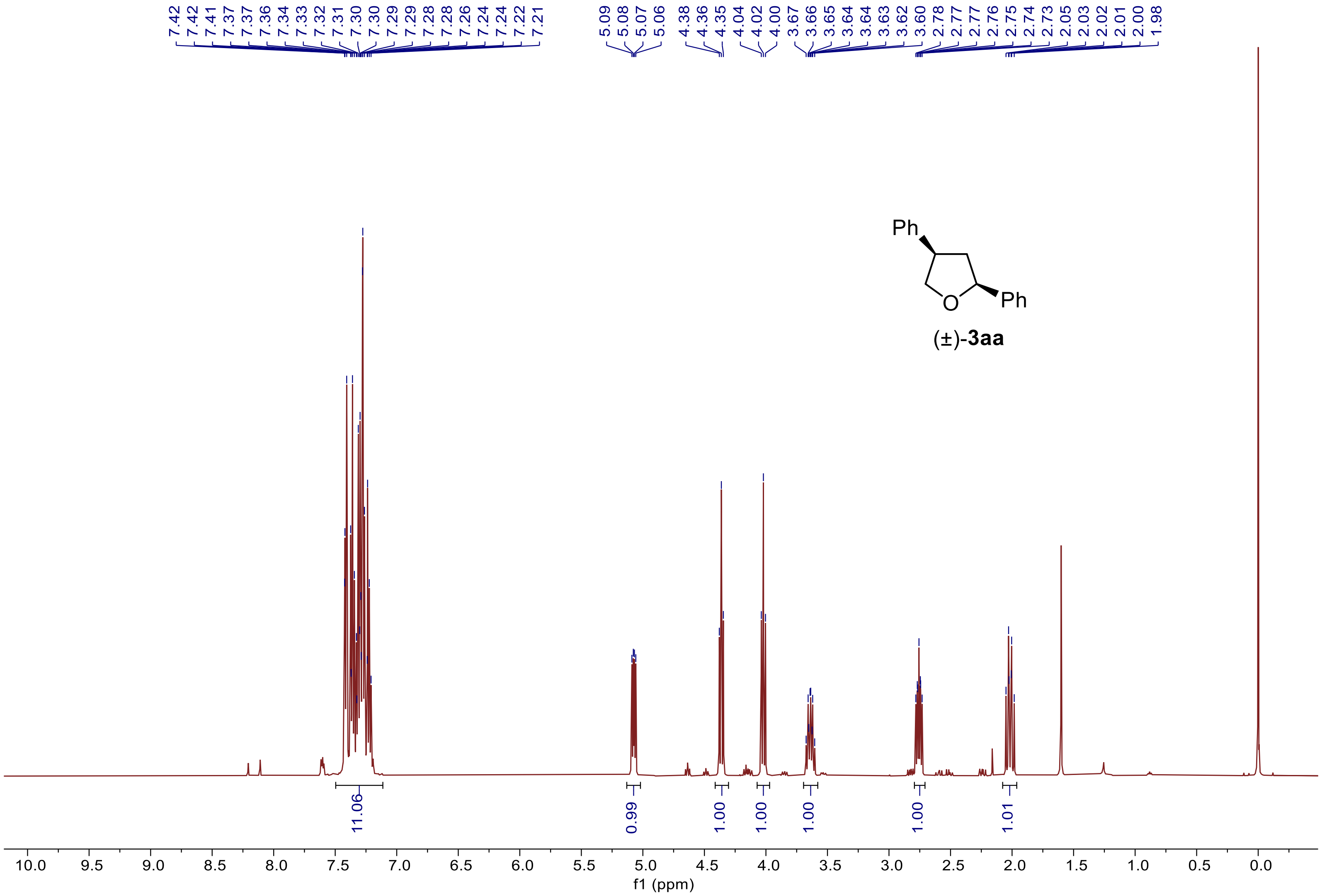

Figure S5. ^{1}H NMR Spectrum of (±)-**3aa** (500 MHz, $CDCl_3$).

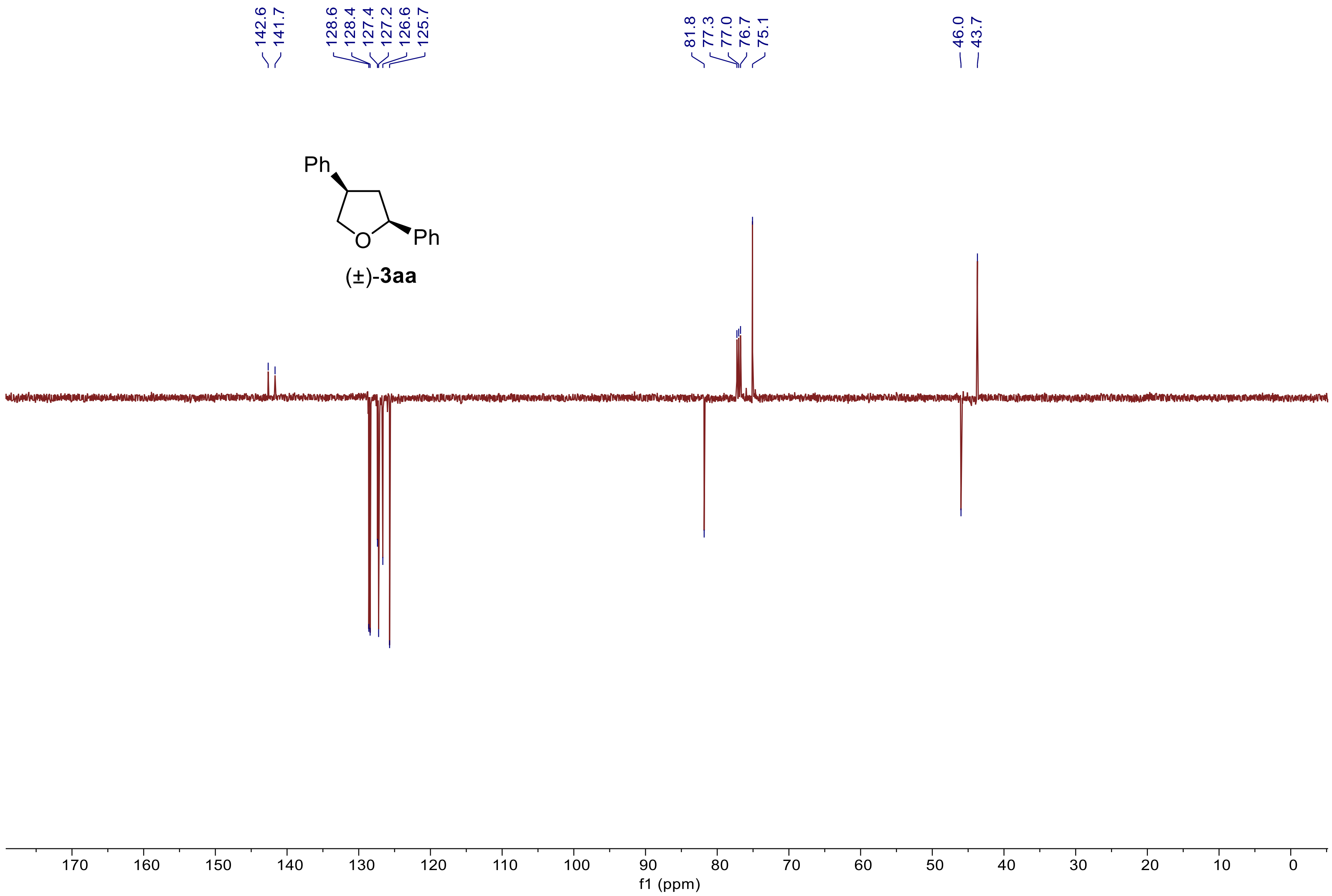

Figure S6. $^{13}C\{^{1}H\}$ NMR Spectrum of (±)-**3aa** (APT, 125 MHz, $CDCl_3$).

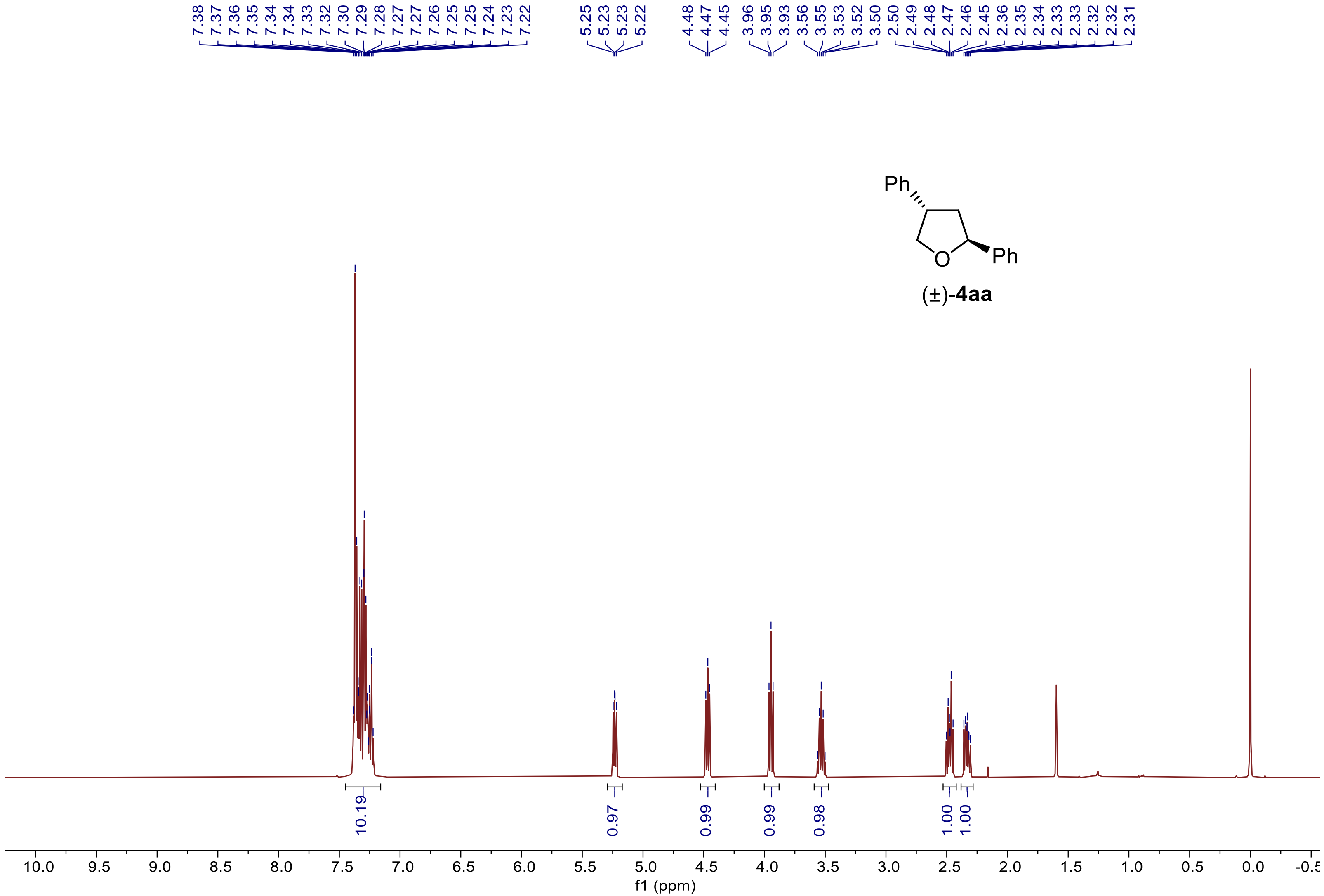

Figure S7. ^{1}H NMR Spectrum of (±)-**4aa** (500 MHz, $CDCl_3$).

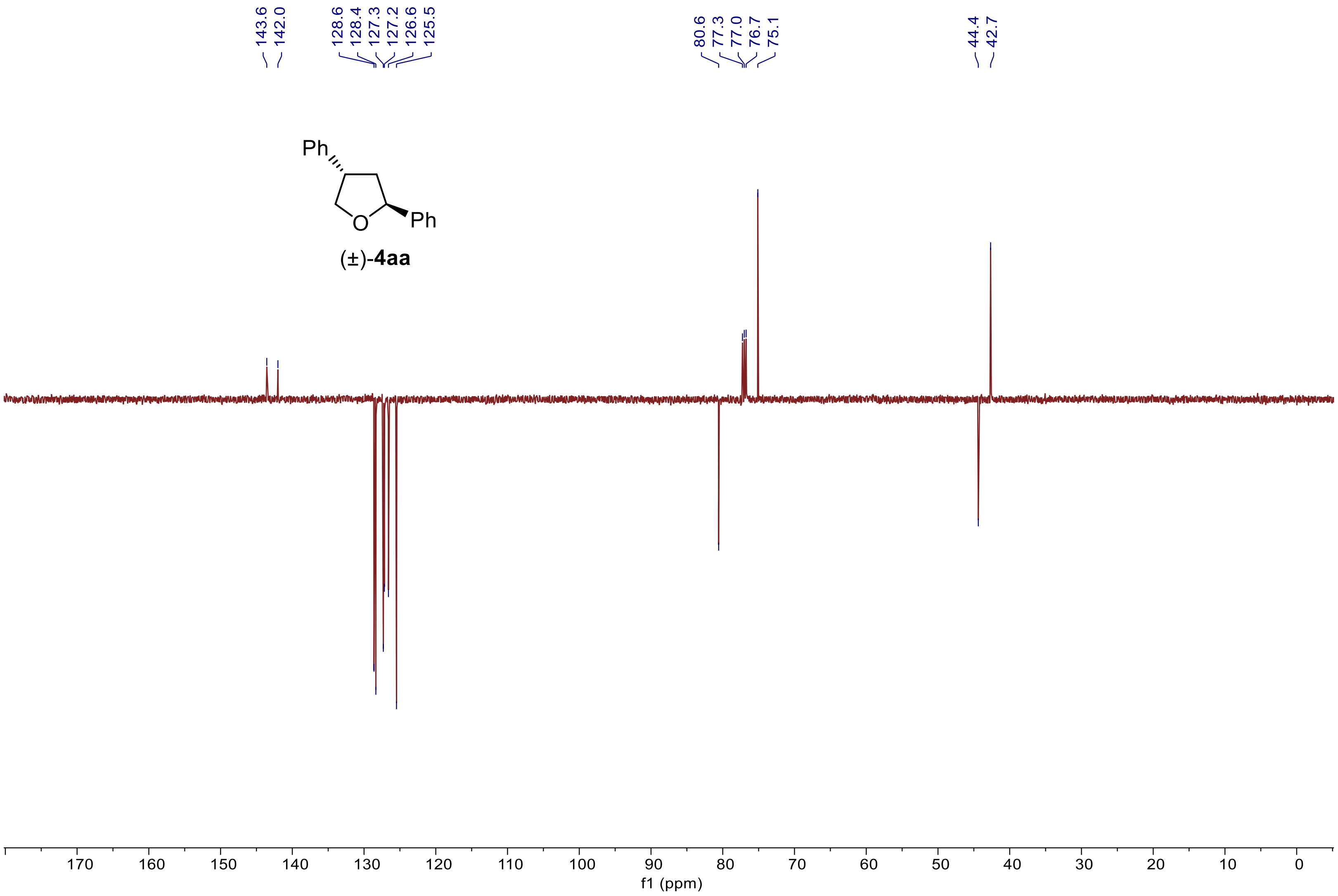

Figure S8. $^{13}C\{^{1}H\}$ NMR Spectrum of (±)-**4aa** (APT, 125 MHz, $CDCl_3$).

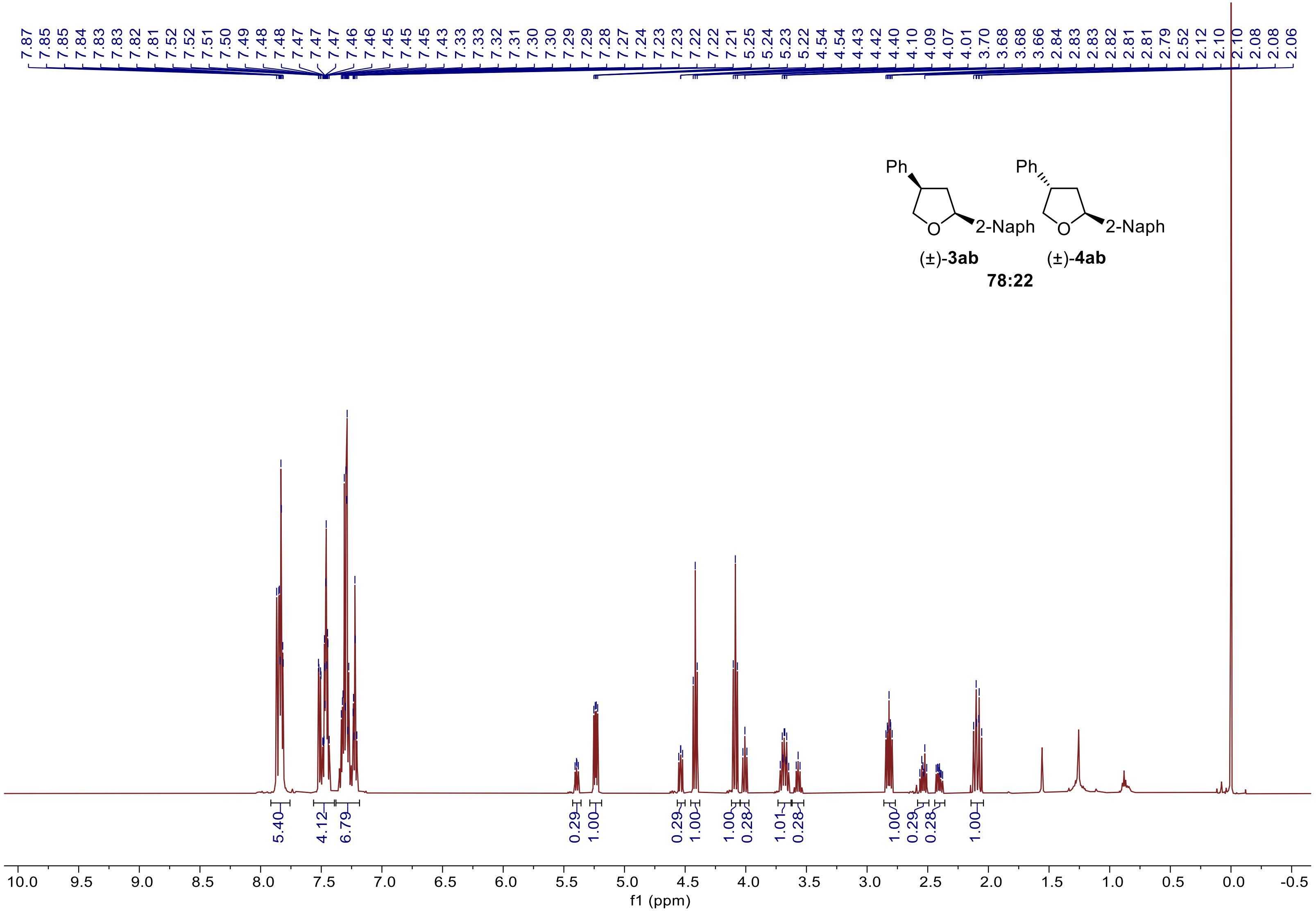

Figure S9. ^{1}H NMR Spectrum of (±)-**3ab** and (±)-**4ab** (500 MHz, $CDCl_3$).

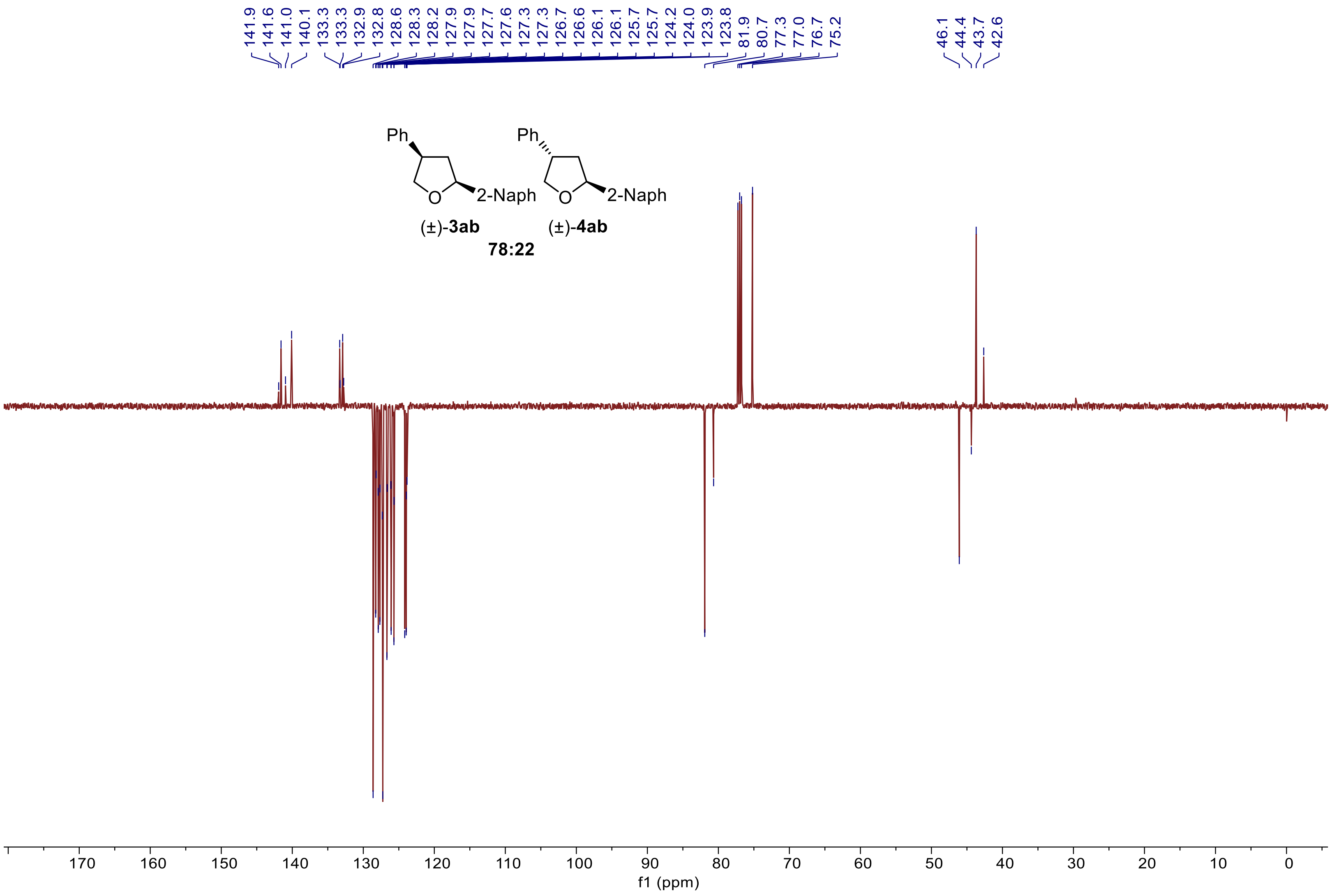

Figure S10. $^{13}C\{^{1}H\}$ NMR Spectrum of (±)-**3ab** and (±)-**4ab** (APT, 125 MHz, $CDCl_3$).

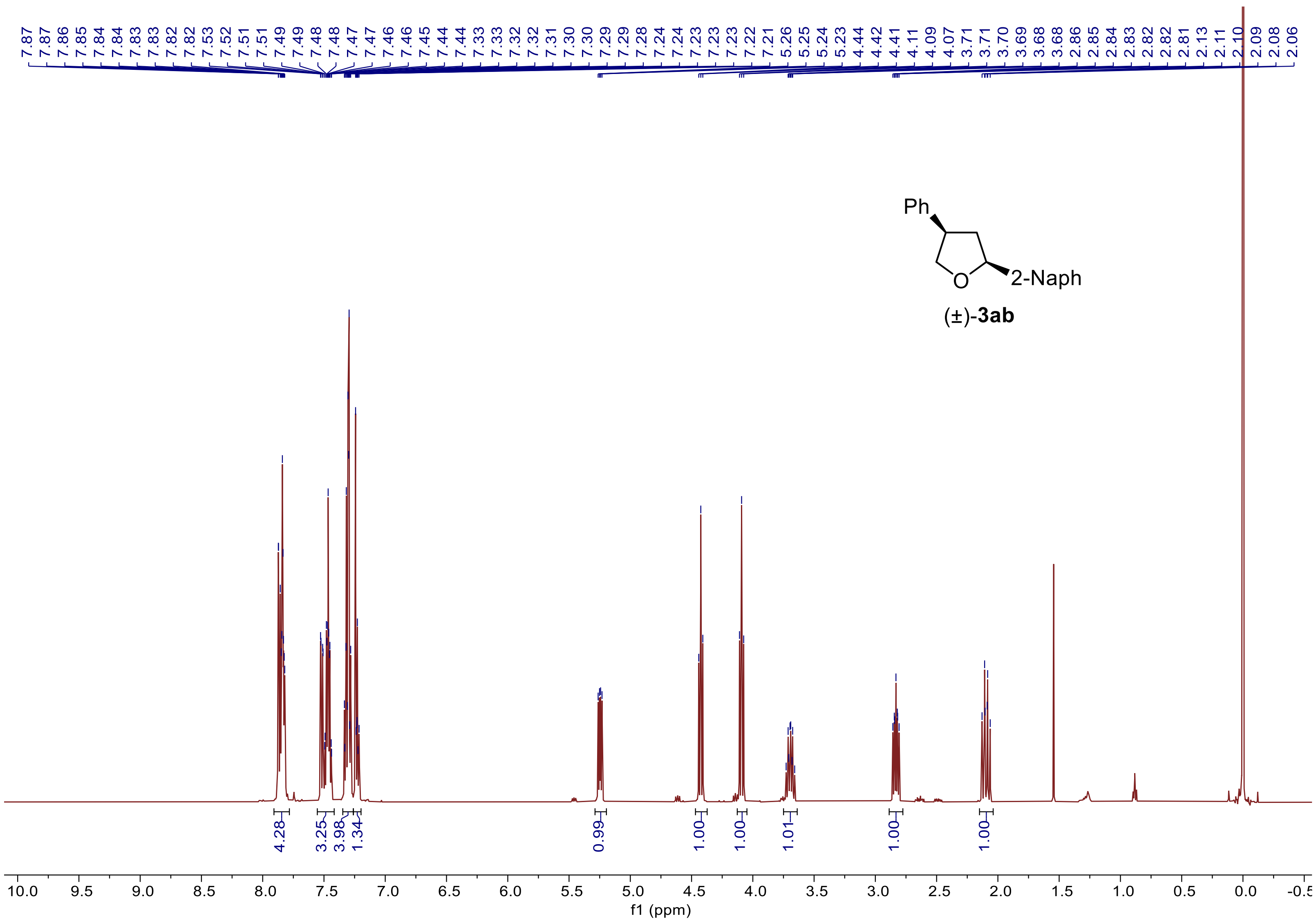

Figure S11. ^{1}H NMR Spectrum of (±)-**3ab** (500 MHz, $CDCl_3$).

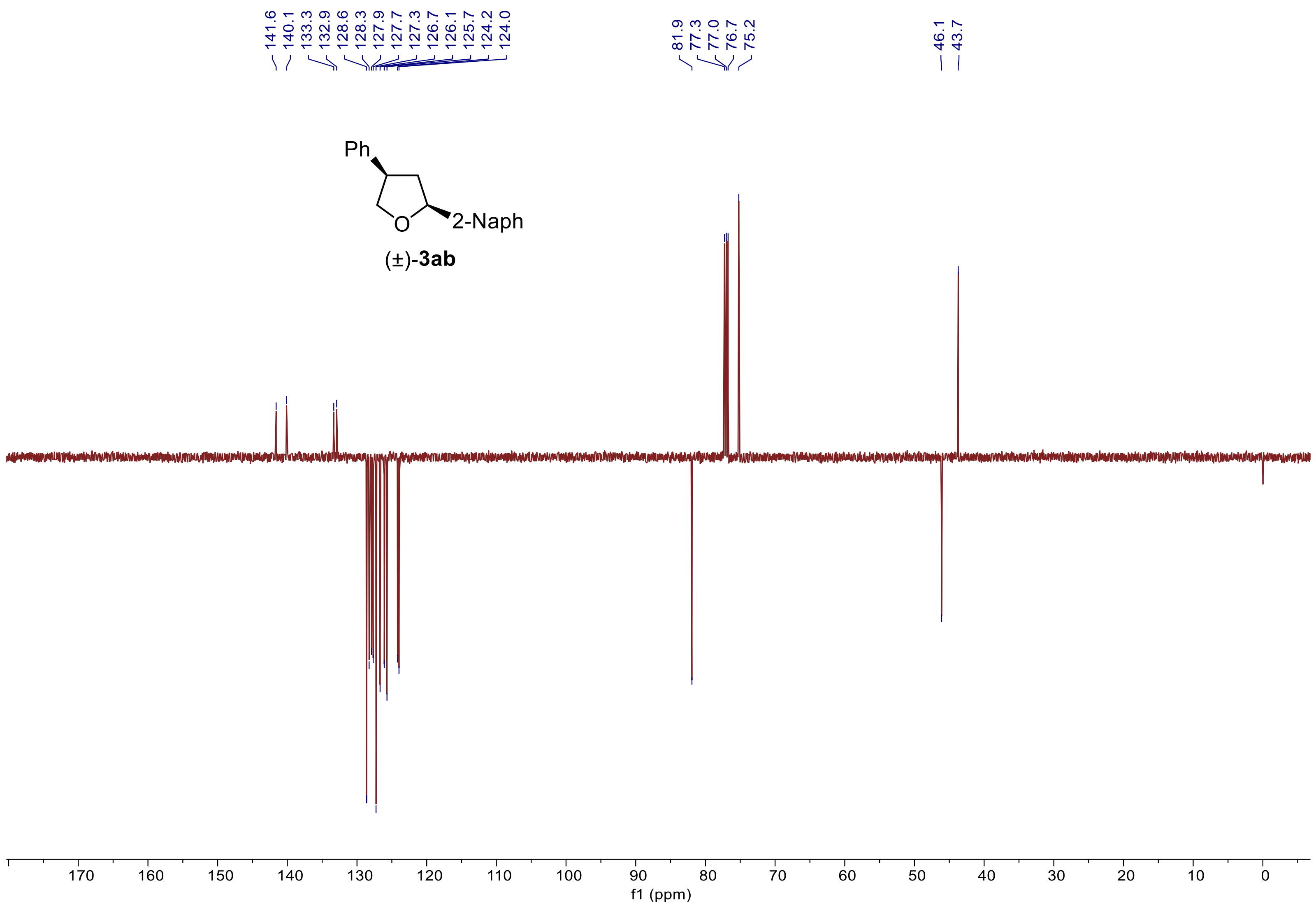

Figure S12. $^{13}C\{^{1}H\}$ NMR Spectrum of (±)-**3ab** (APT, 125 MHz, $CDCl_3$).

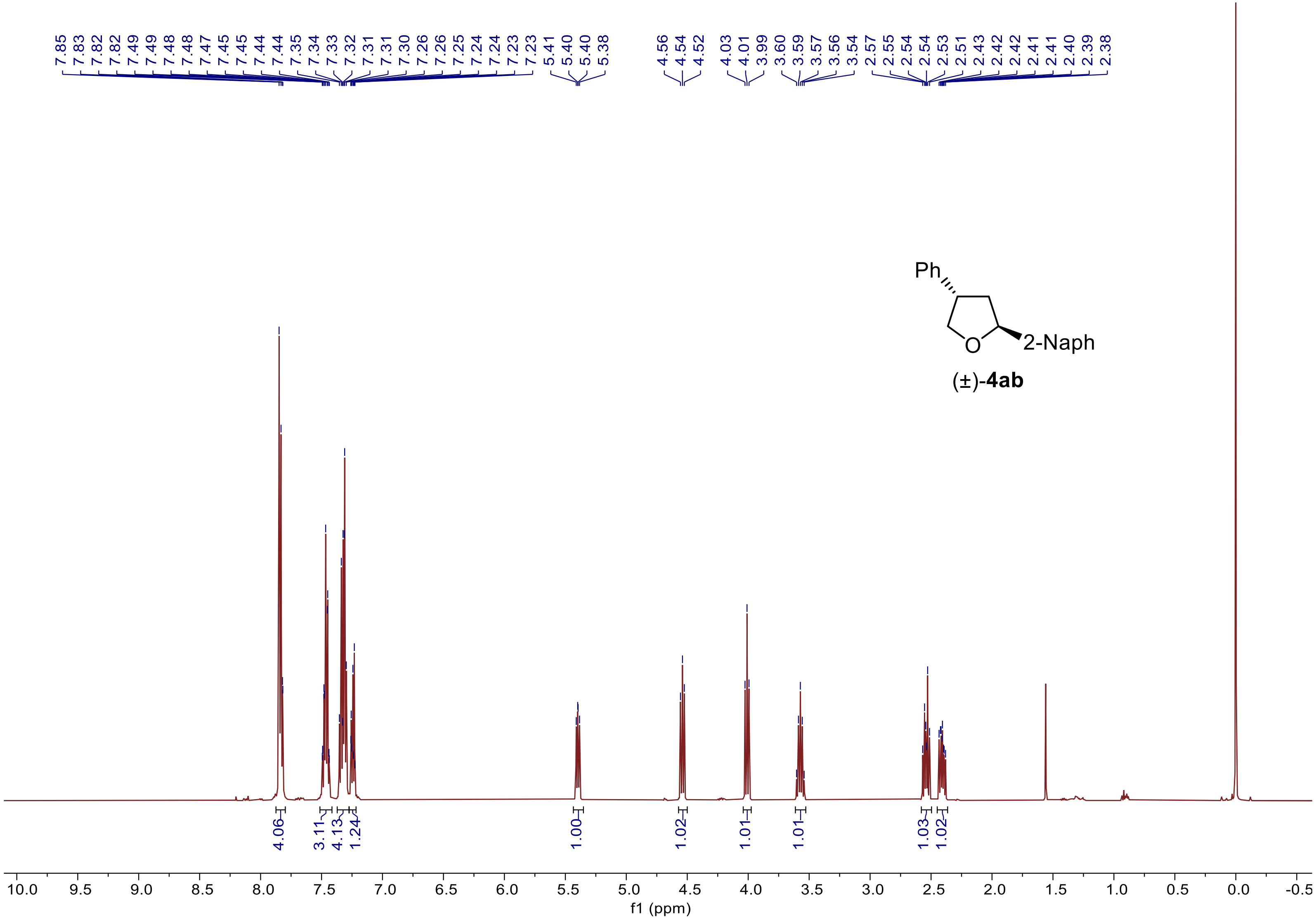

Figure S13. ^{1}H NMR Spectrum of (±)-**4ab** (500 MHz, $CDCl_3$).

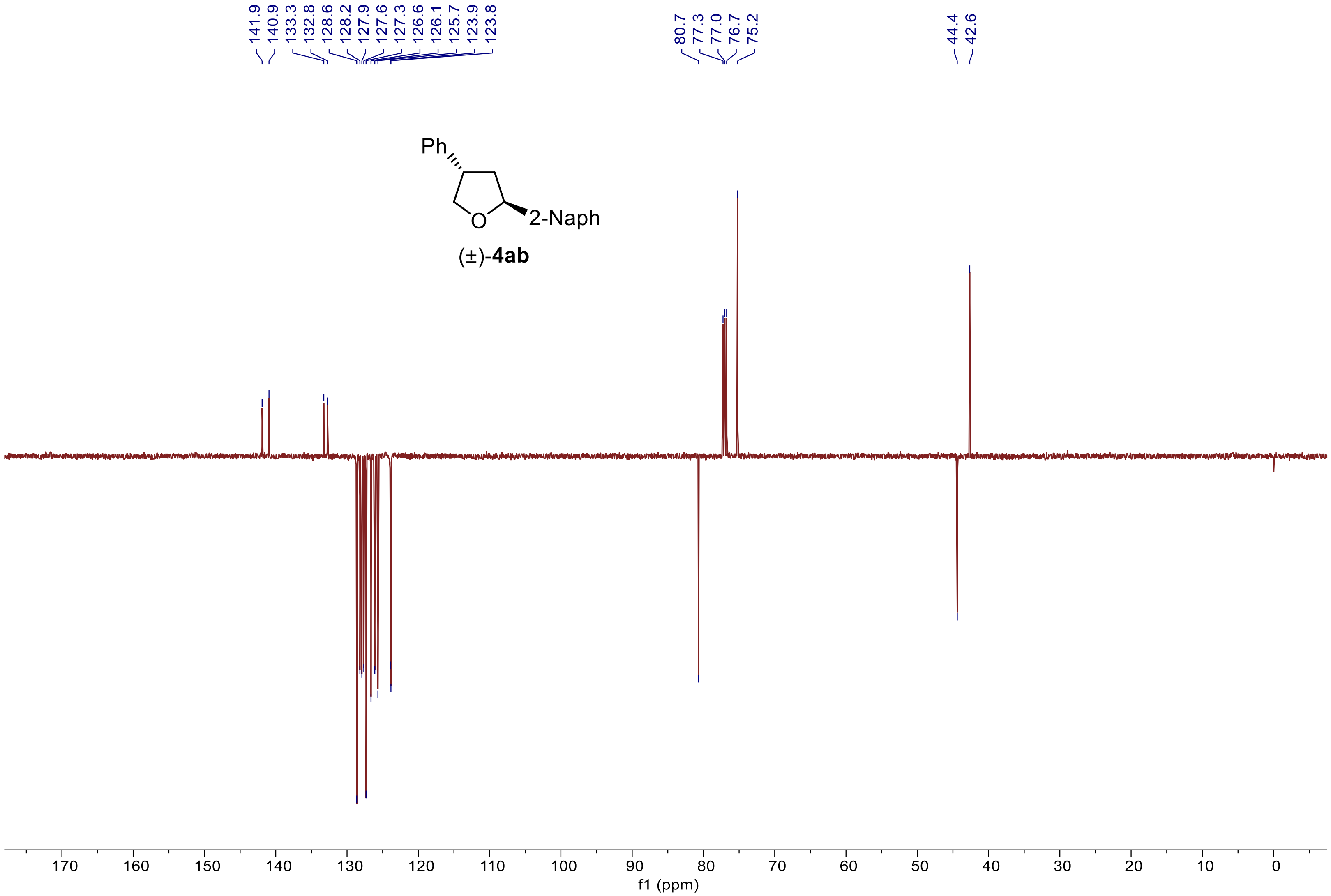

Figure S14. $^{13}C\{^{1}H\}$ NMR Spectrum of (±)-**4ab** (APT, 125 MHz, $CDCl_3$).

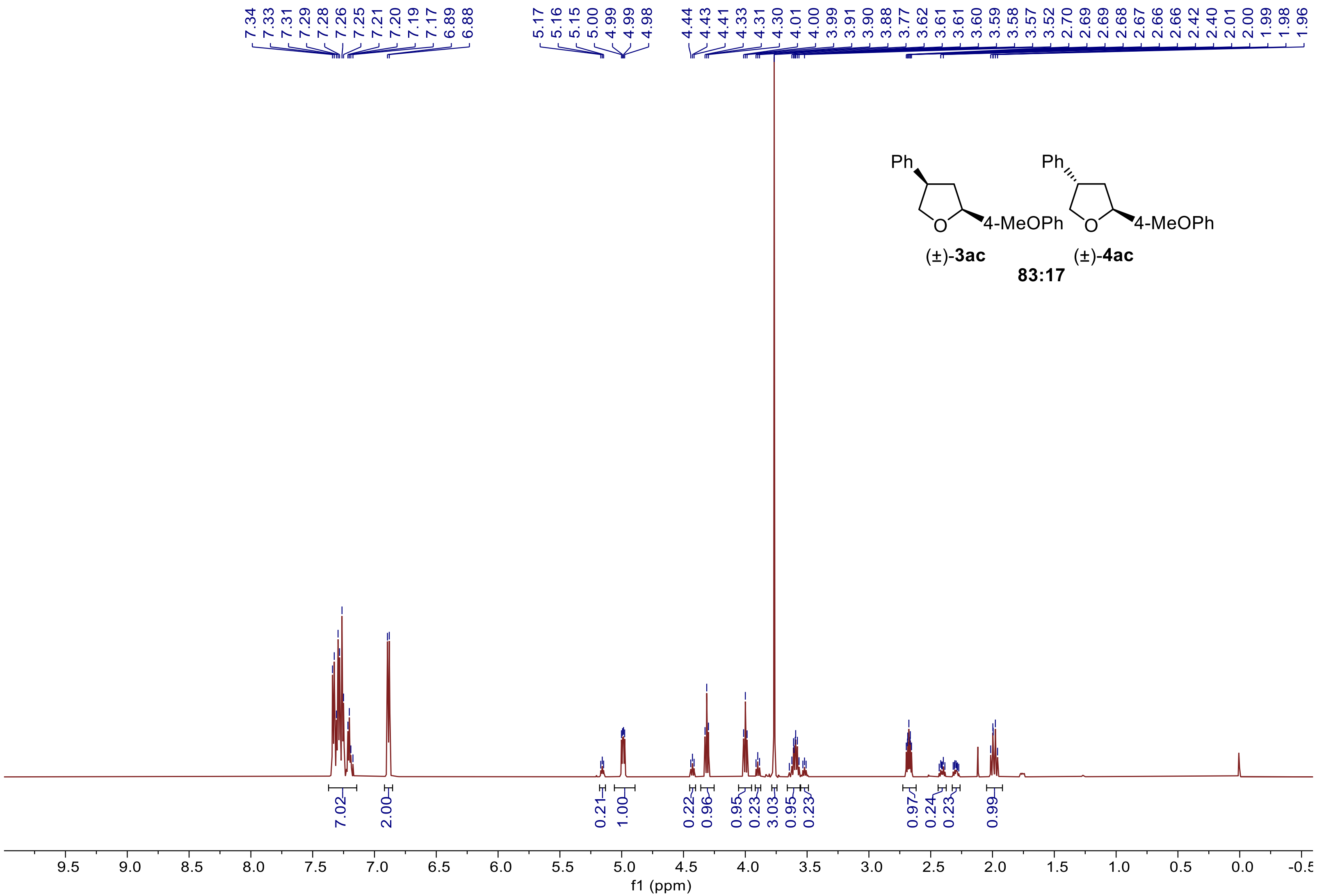

Figure S15. ^{1}H NMR Spectrum of (±)-**3ac** and (±)-4**ac** (500 MHz, $CDCl_3$).

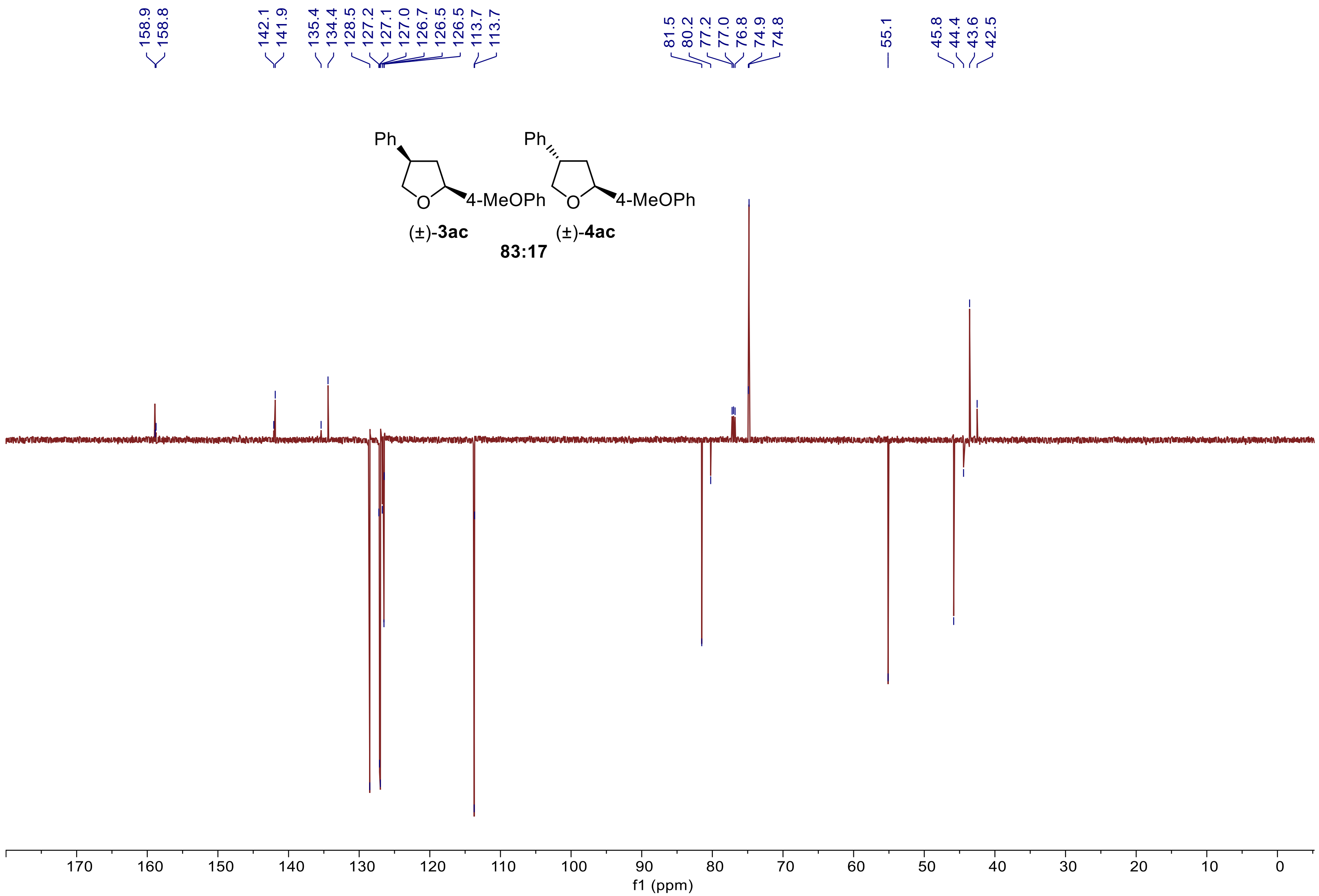

Figure S16. $^{13}C\{^{1}H\}$ NMR Spectrum of (±)-**3ac** and (±)-**4ac** (APT, 125 MHz, $CDCl_3$).

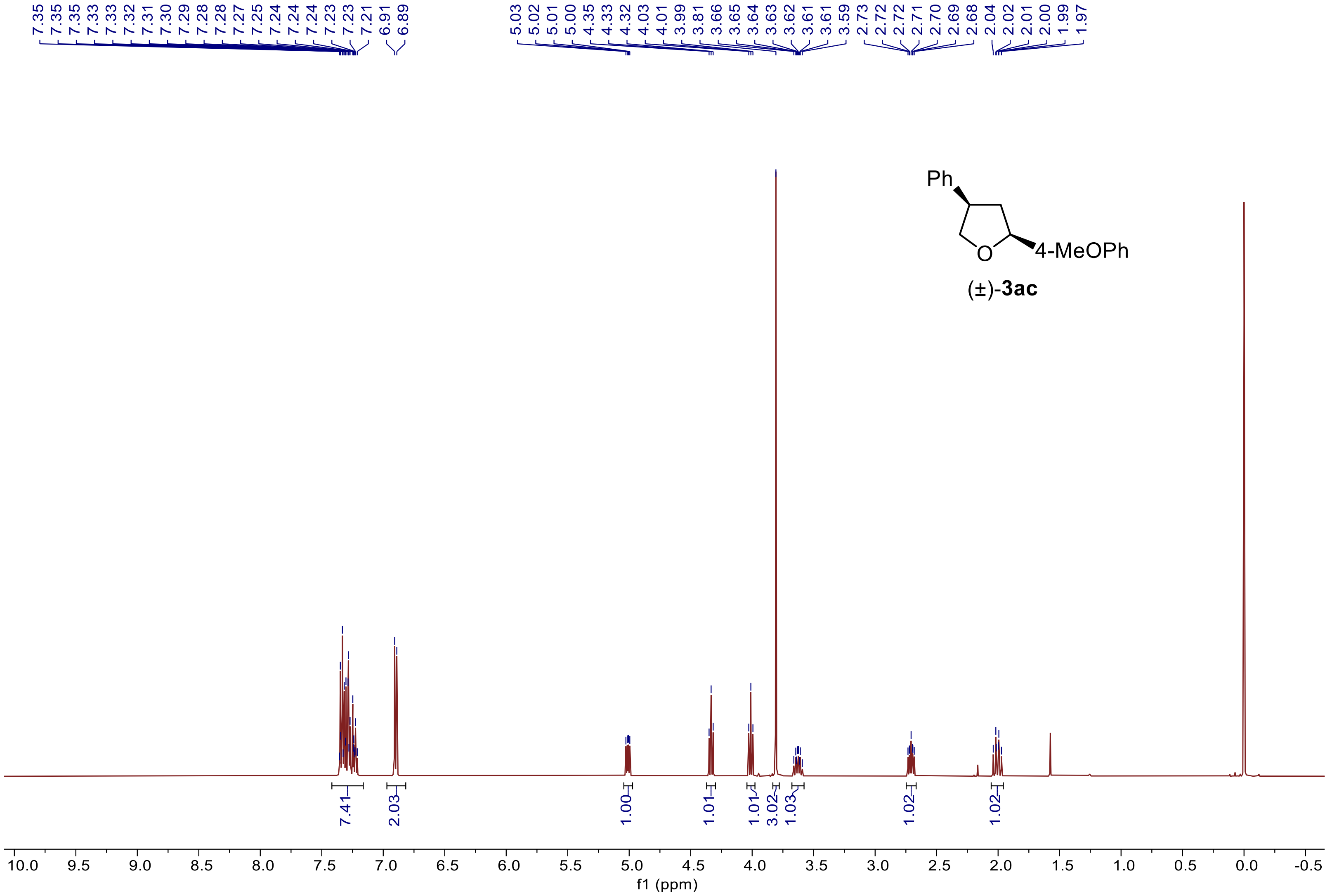

Figure S17. ^{1}H NMR Spectrum of (±)-**3ac** (500 MHz, $CDCl_3$).

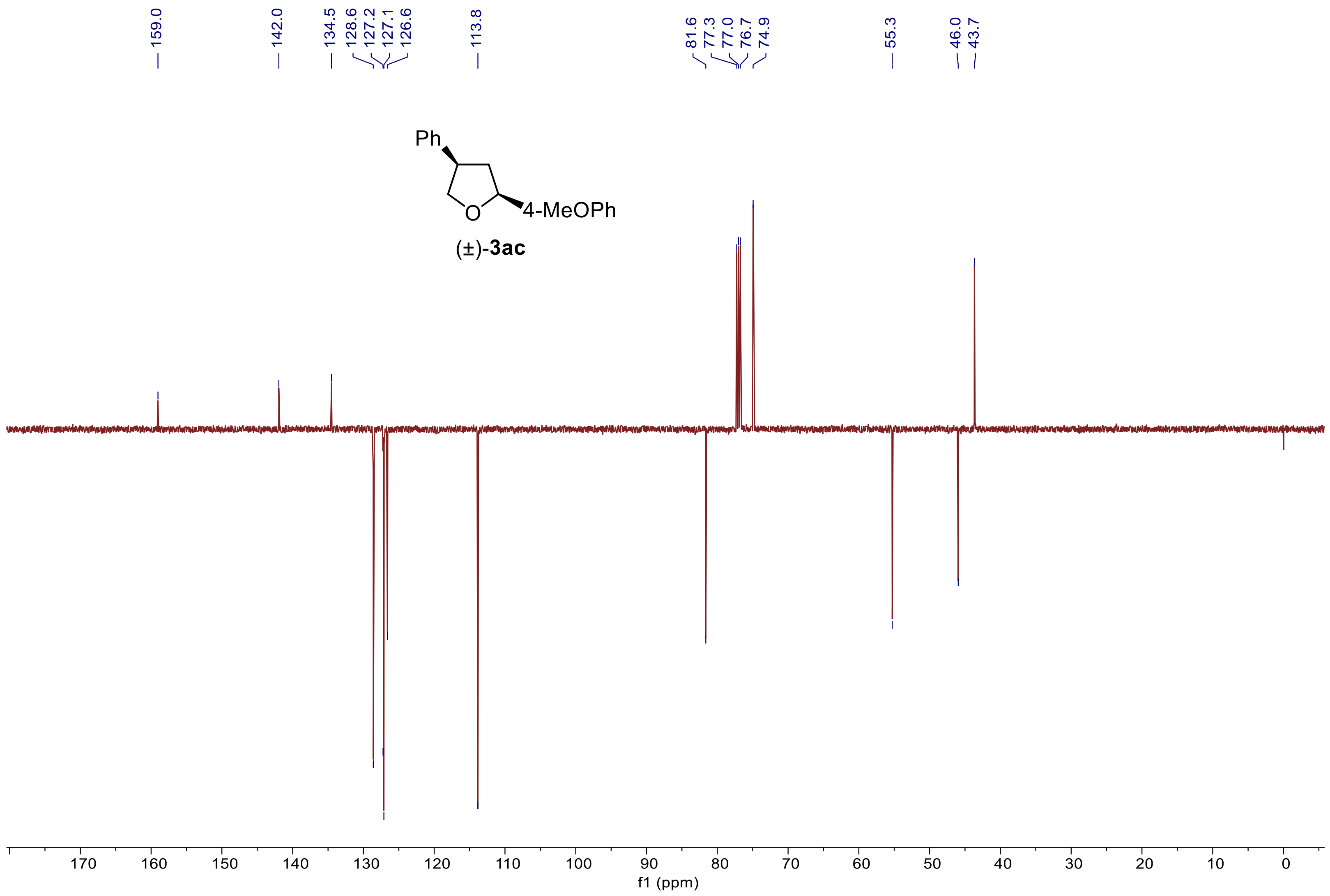

Figure S18. $^{13}C\{^{1}H\}$ NMR Spectrum of (±)-**3ac** (APT, 125 MHz, $CDCl_3$).

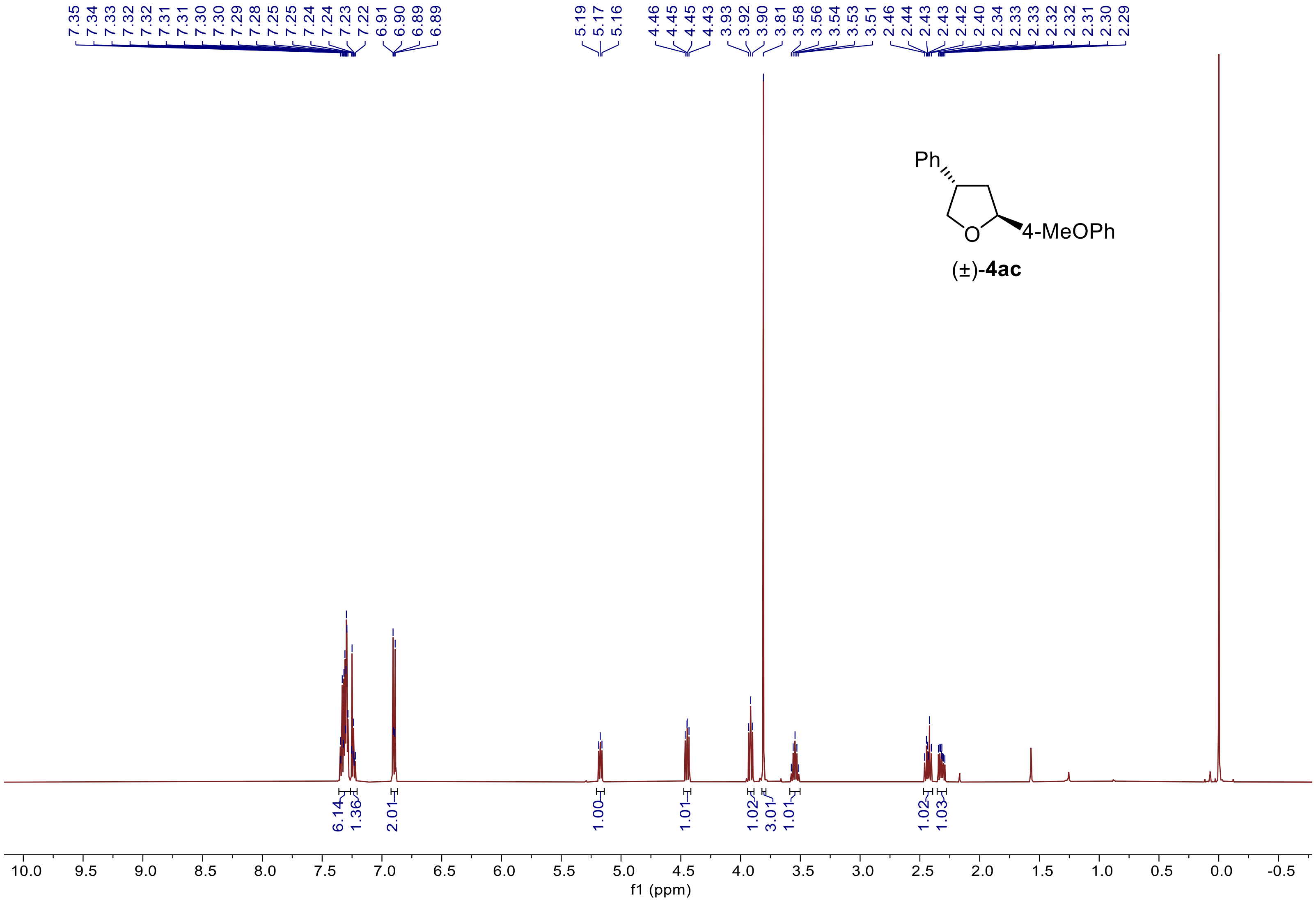

Figure S19. ^{1}H NMR Spectrum of (±)-**4ac** (500 MHz, $CDCl_3$).

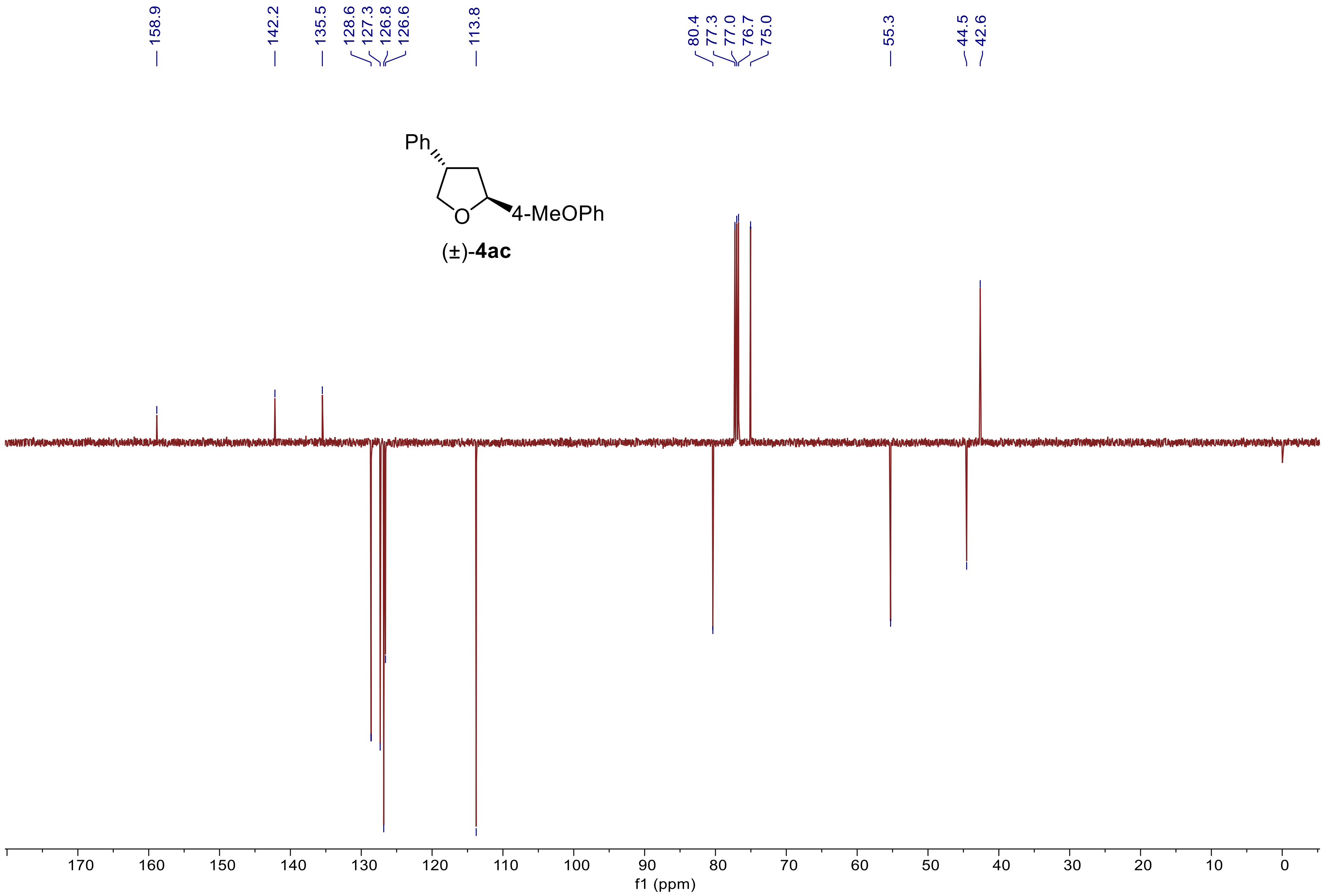

Figure S20. $^{13}C\{^{1}H\}$ NMR Spectrum of (±)-**4ac** (APT, 125 MHz, $CDCl_3$).

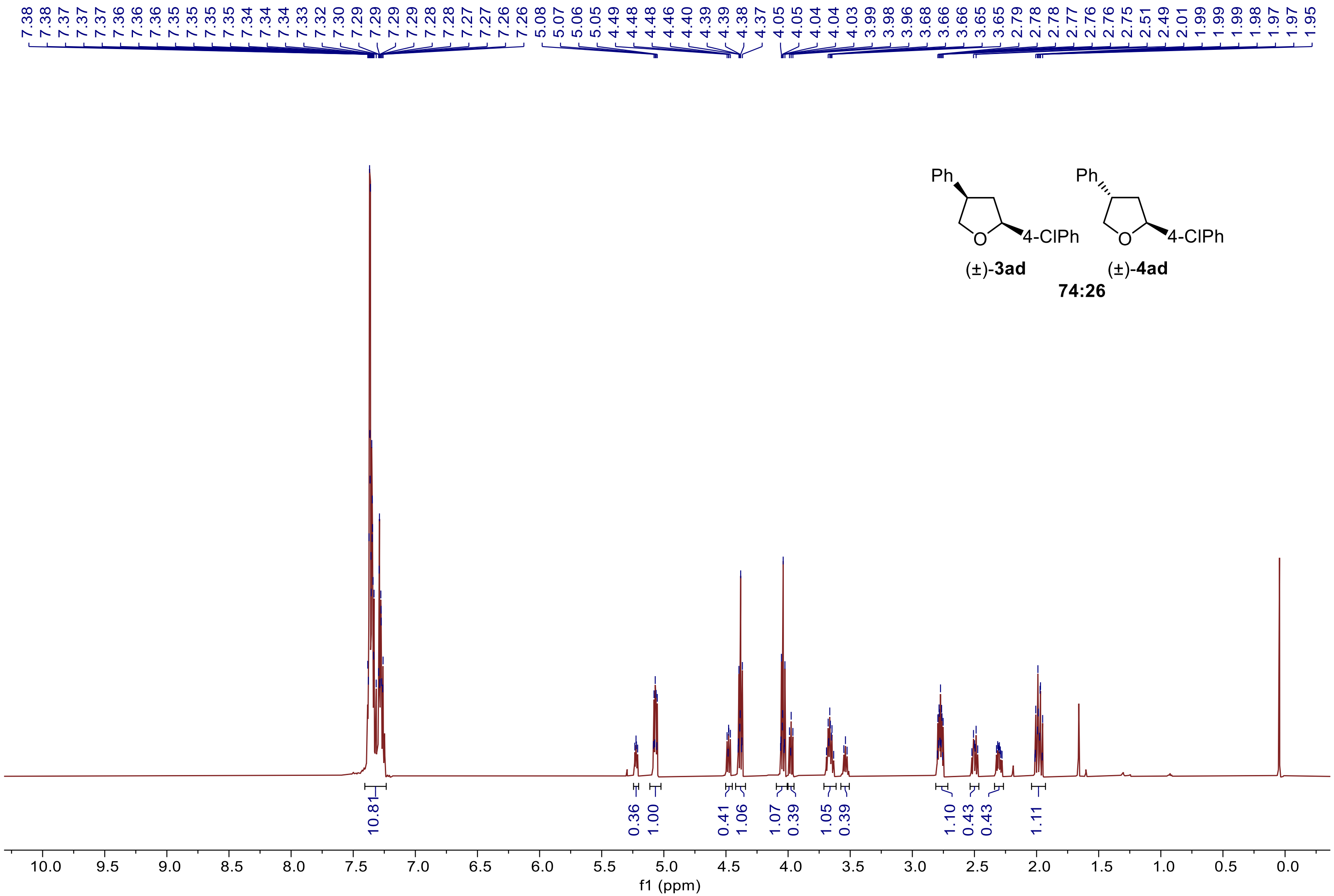

Figure S21. ^{1}H NMR Spectrum of (±)-**3ad** and (±)-4**ad** (500 MHz, $CDCl_3$).

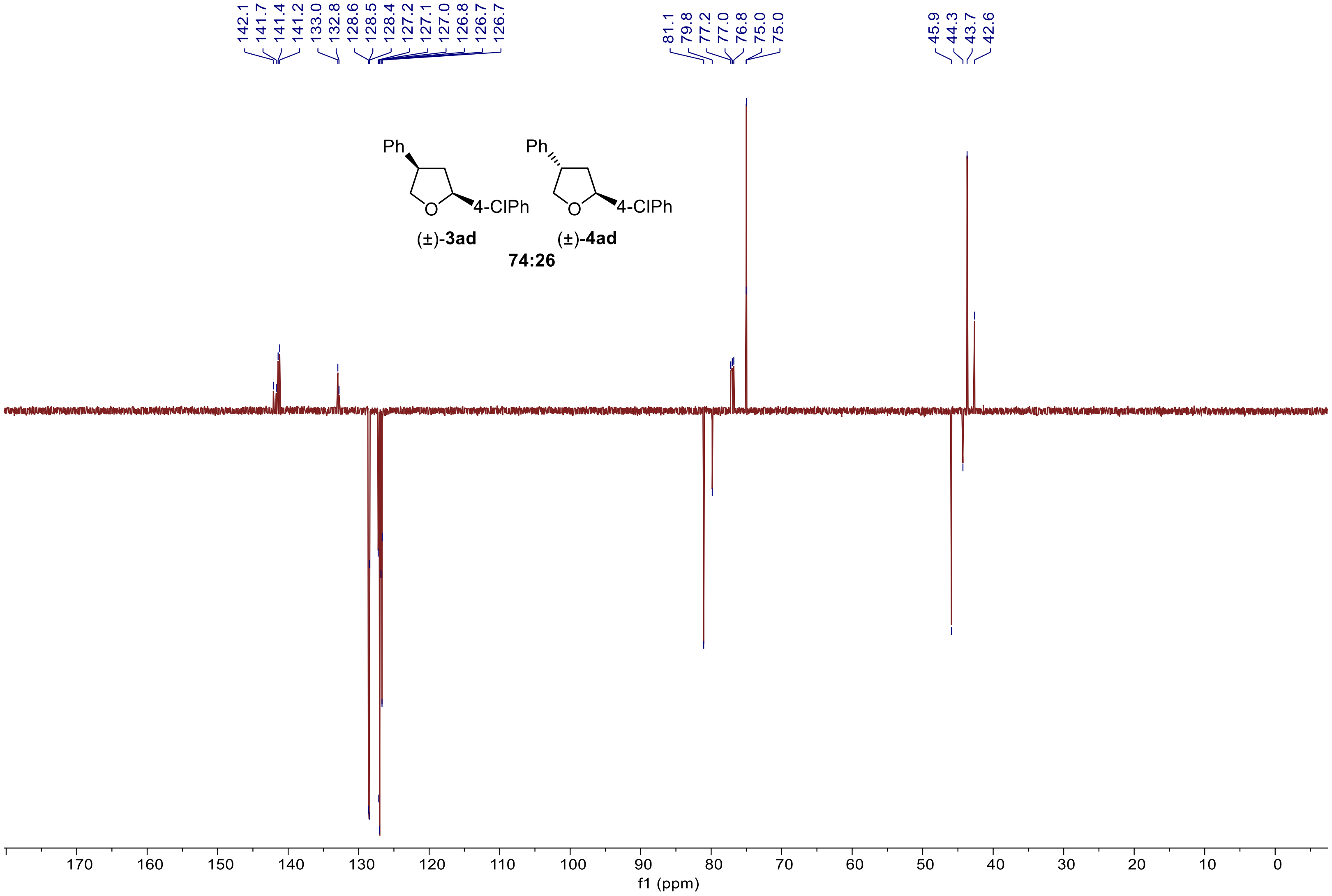

Figure S22. $^{13}C\{^1H\}$ NMR Spectrum of (±)-**3ad** and (±)-**4ad** (APT, 125 MHz, $CDCl_3$).

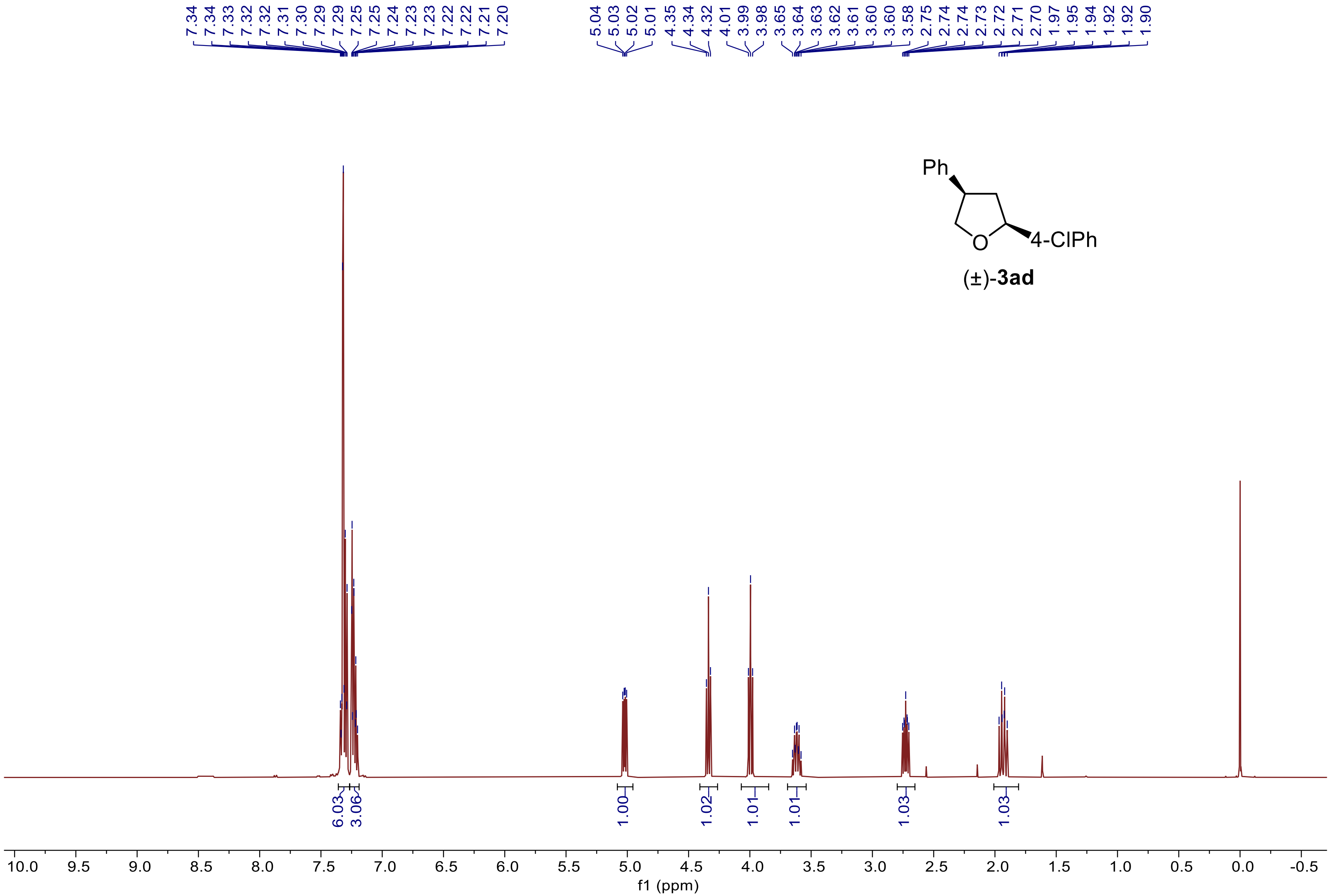

Figure S23. ^{1}H NMR Spectrum of (±)-**3ad** (500 MHz, $CDCl_3$).

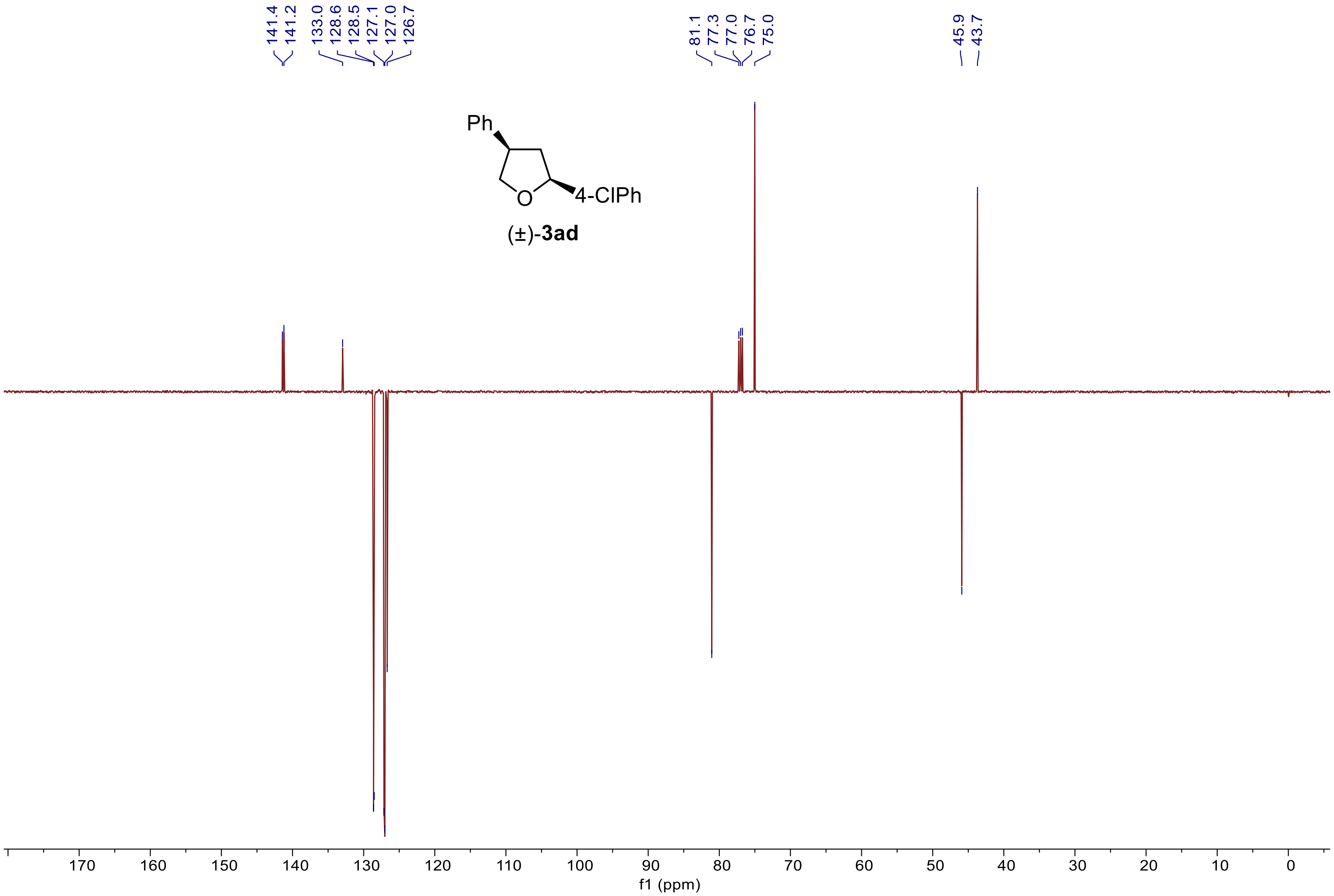

Figure S24. $^{13}C\{^1H\}$ NMR Spectrum of (±)-**3ad** (APT, 125 MHz, $CDCl_3$).

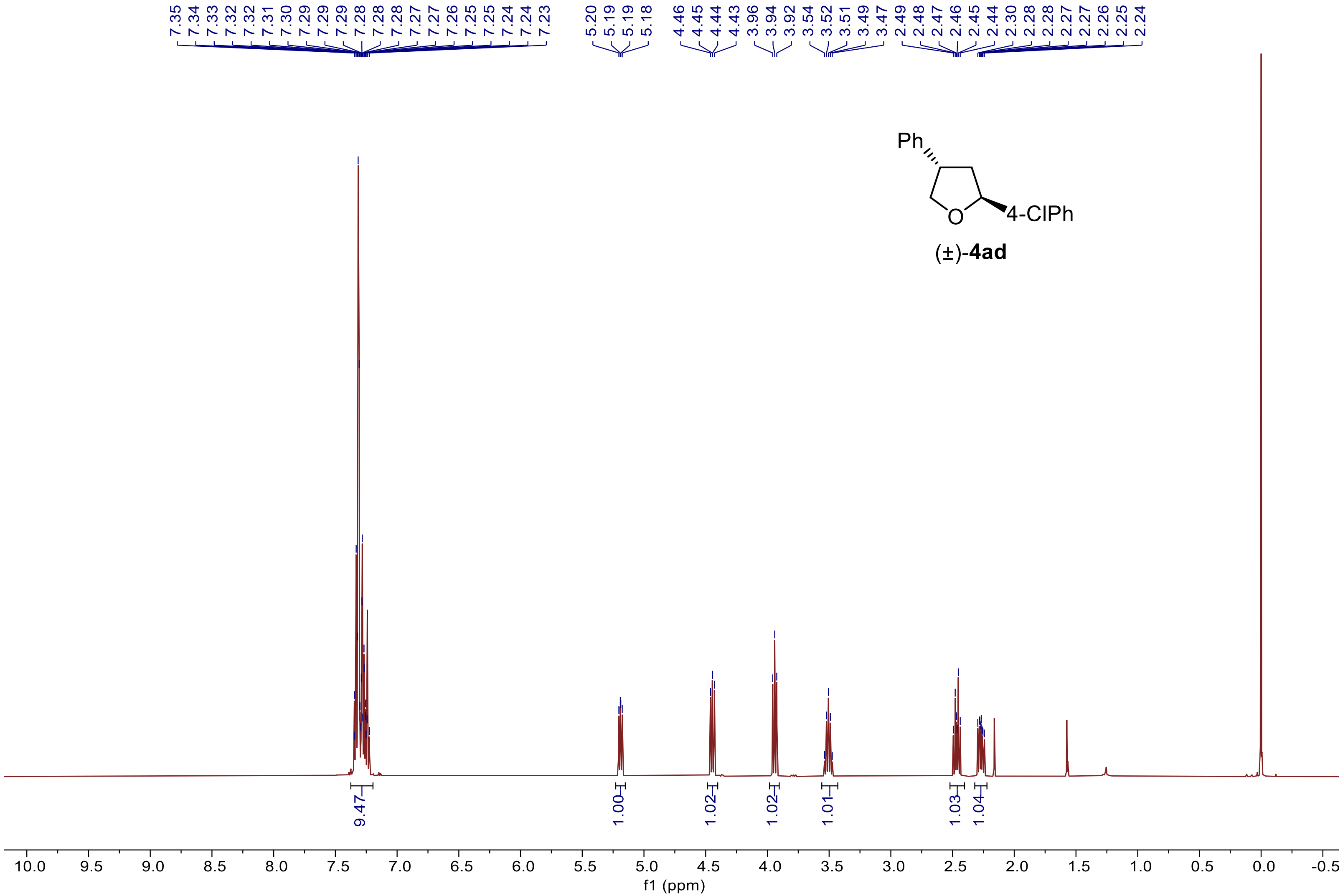

Figure S25. ^{1}H NMR Spectrum of (±)-**4ad** (500 MHz, $CDCl_3$).

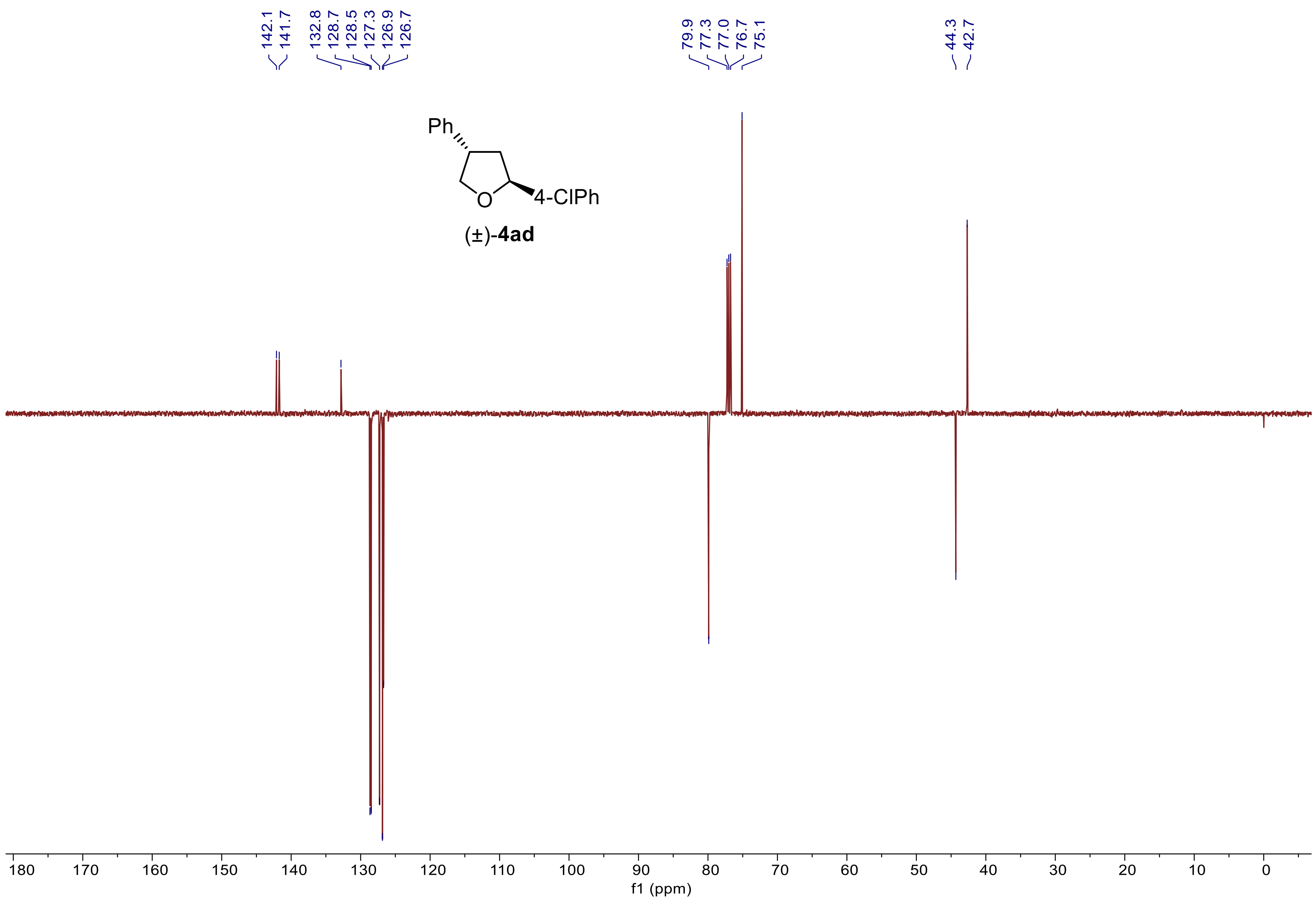

Figure S26. $^{13}C\{^{1}H\}$ NMR Spectrum of (±)-**3ad** (APT, 125 MHz, $CDCl_3$).

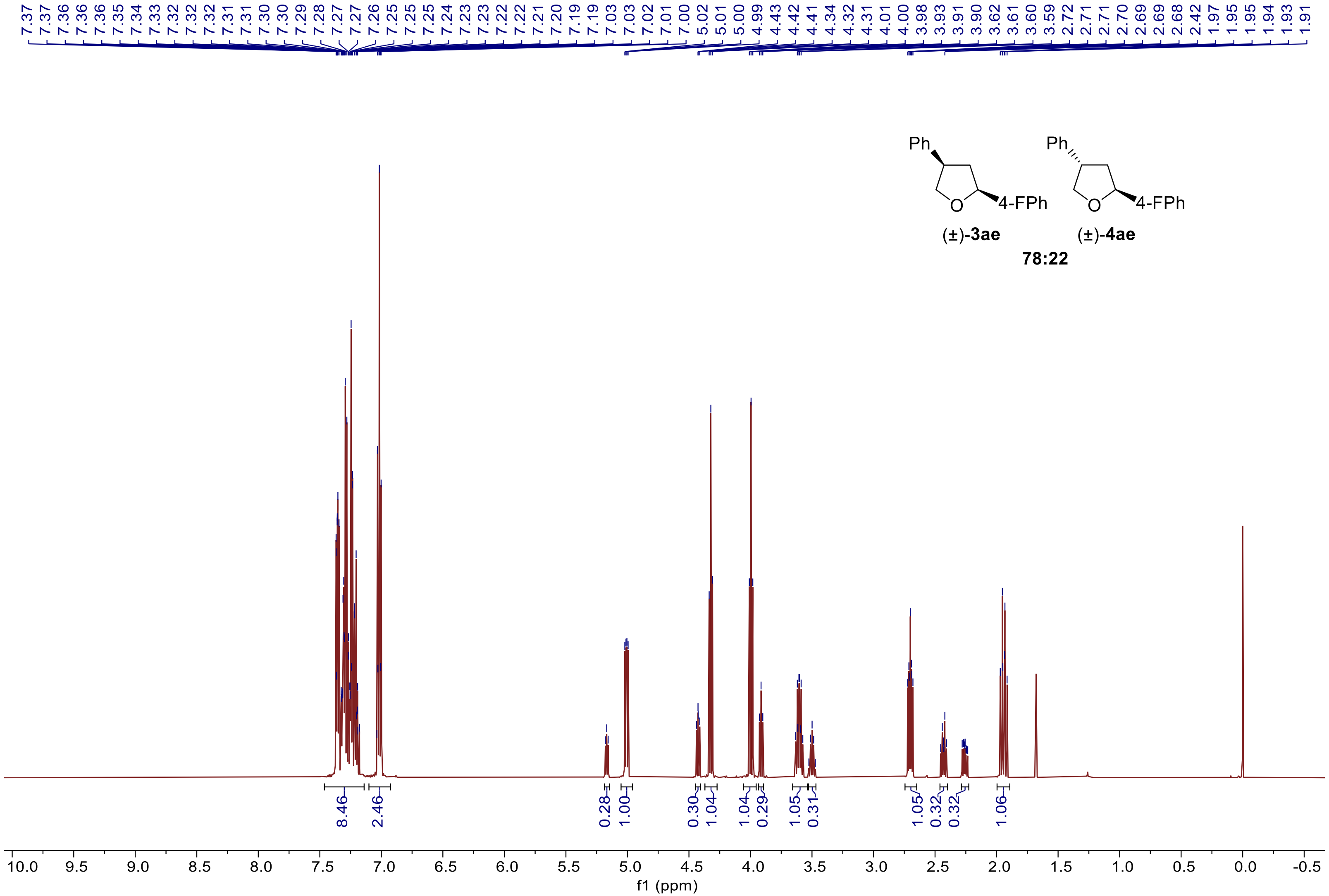

Figure S27. ^{1}H NMR Spectrum of (±)-**3ae** and (±)-4**ae** (600 MHz, $CDCl_3$).

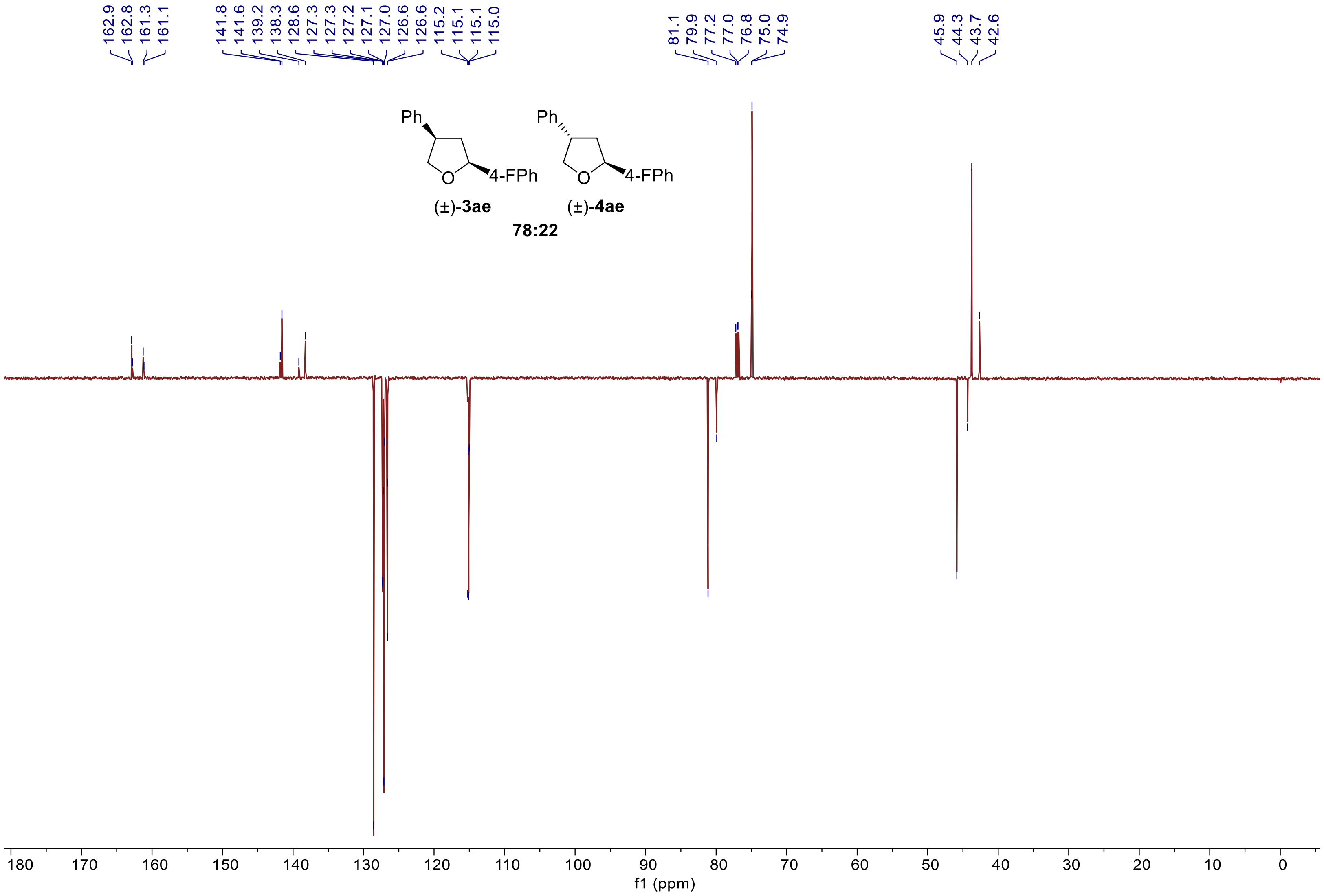

Figure S28. $^{13}C\{^{1}H\}$ NMR Spectrum of (±)-**3ae** and (±)-**4ae** (APT, 150 MHz, $CDCl_3$).

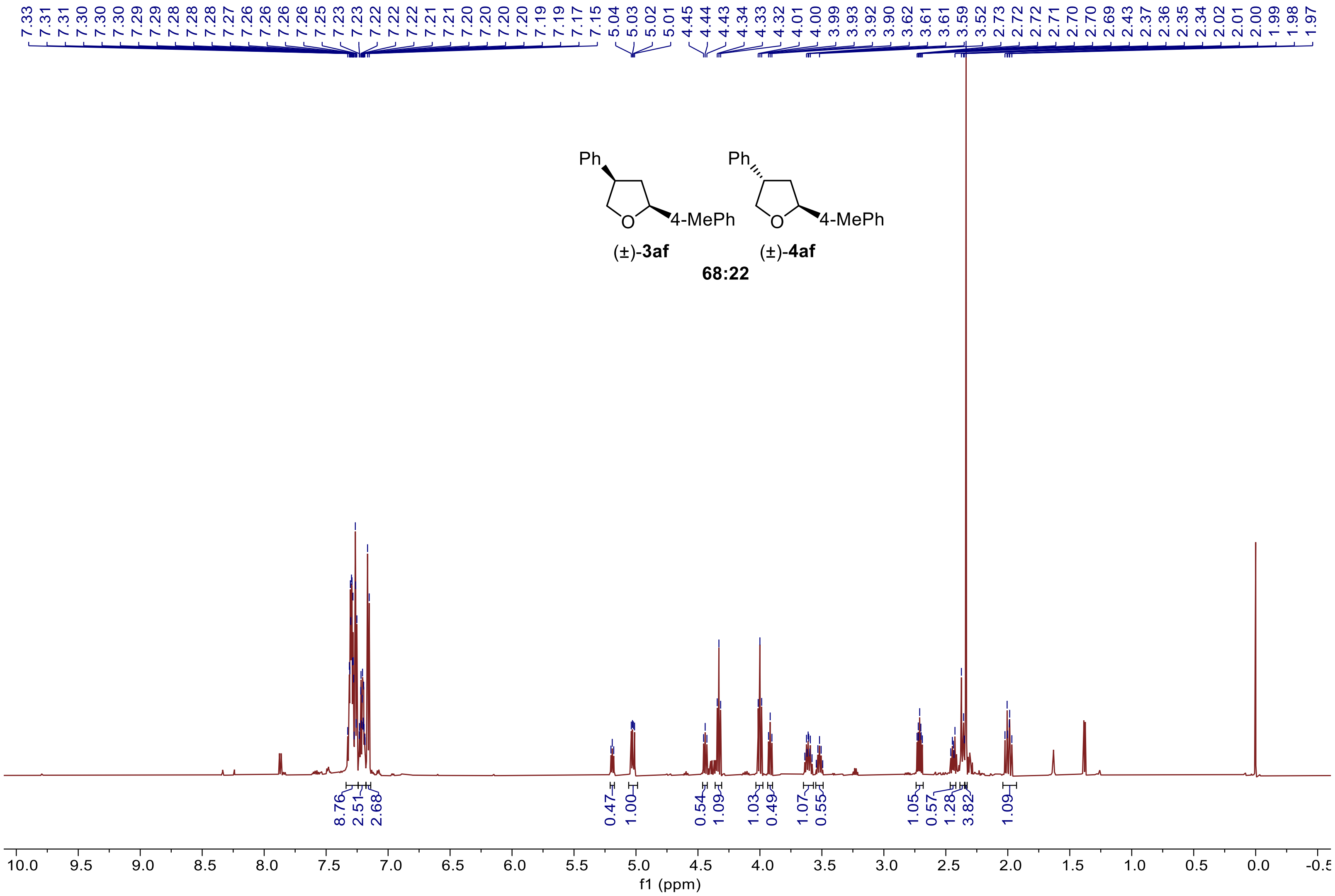

Figure S29. ^{1}H NMR Spectrum of (±)-**3af** and (±)-4**af** (600 MHz, $CDCl_3$).

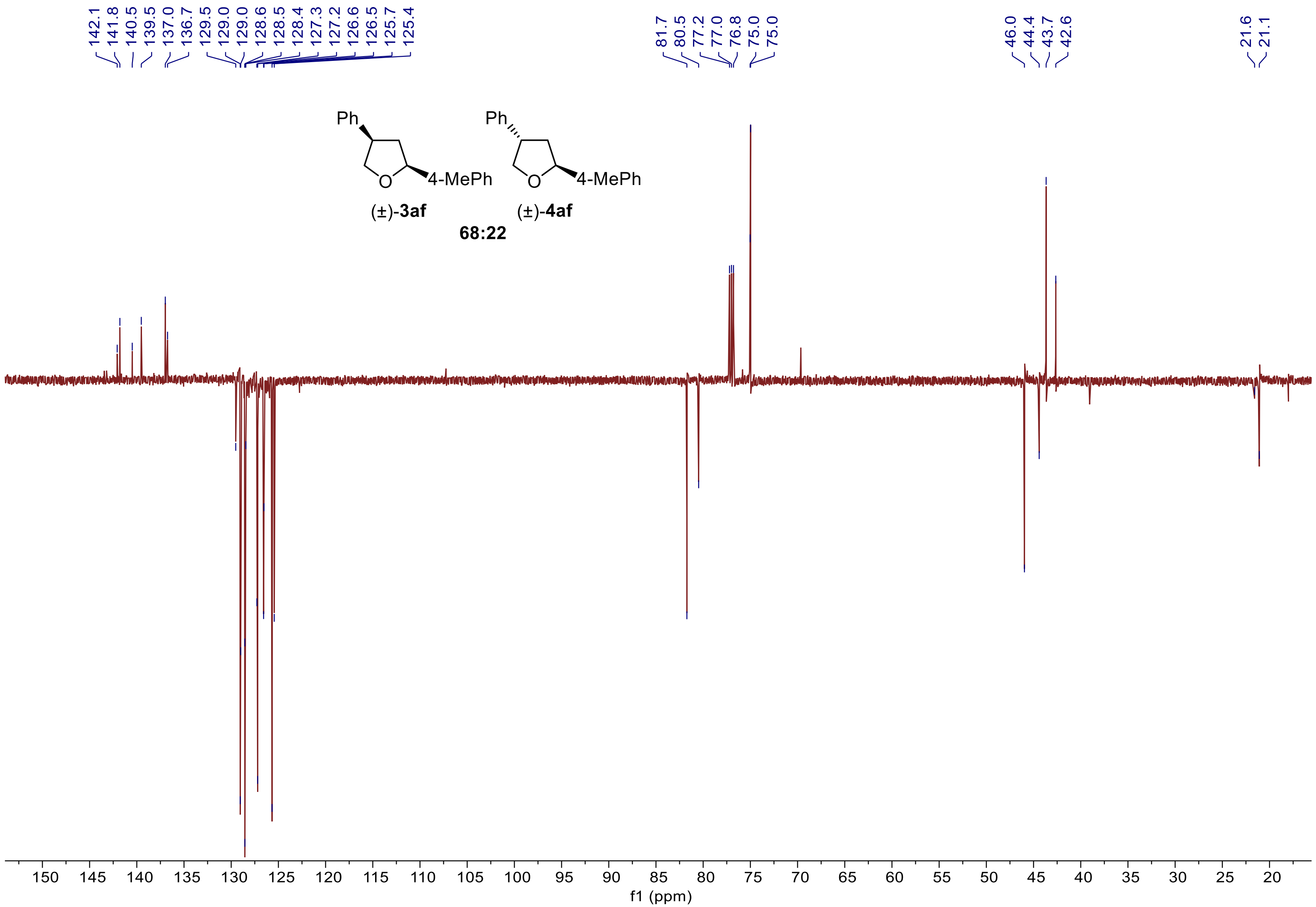

Figure S30. $^{13}C\{^{1}H\}$ NMR Spectrum of (±)-**3af** and (±)-**4af** (APT, 150 MHz, $CDCl_3$).

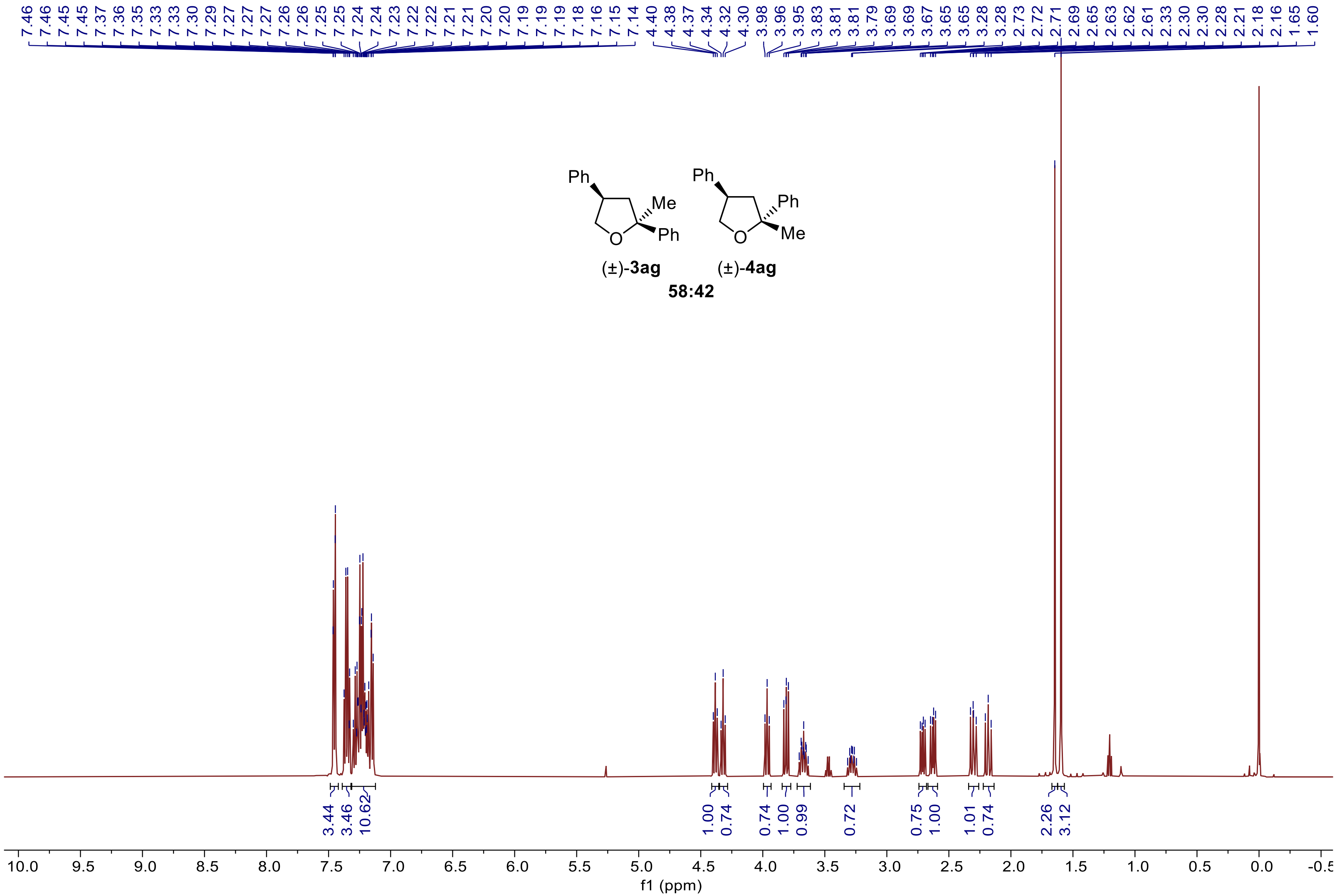

Figure S31. ^{1}H NMR Spectrum of (±)-**3ag** and (±)-4**ag** (500 MHz, $CDCl_3$).

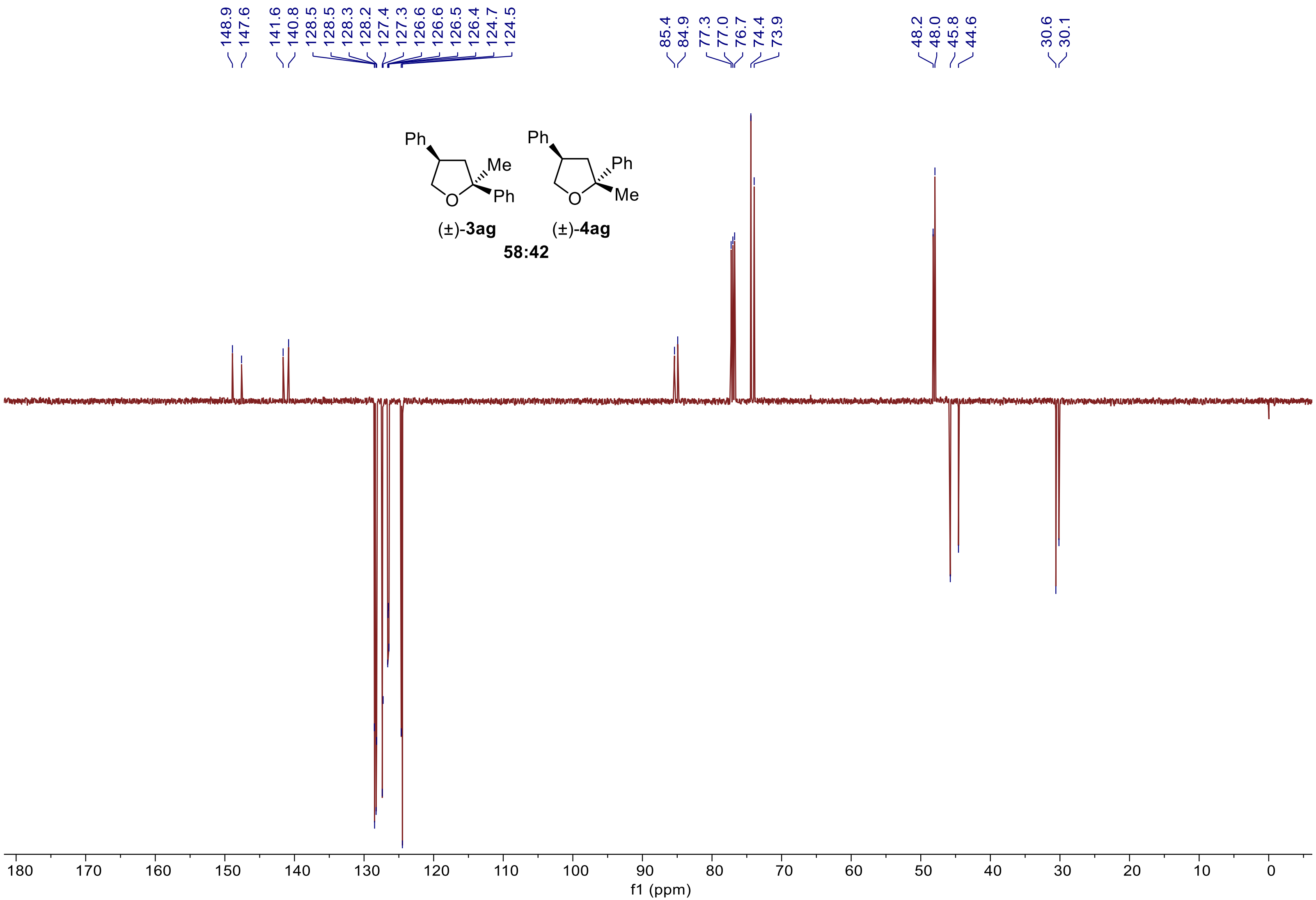

Figure S32. $^{13}C\{^{1}H\}$ NMR Spectrum of (±)-**3ag** and (±)-**4ag** (APT, 125 MHz, $CDCl_3$).

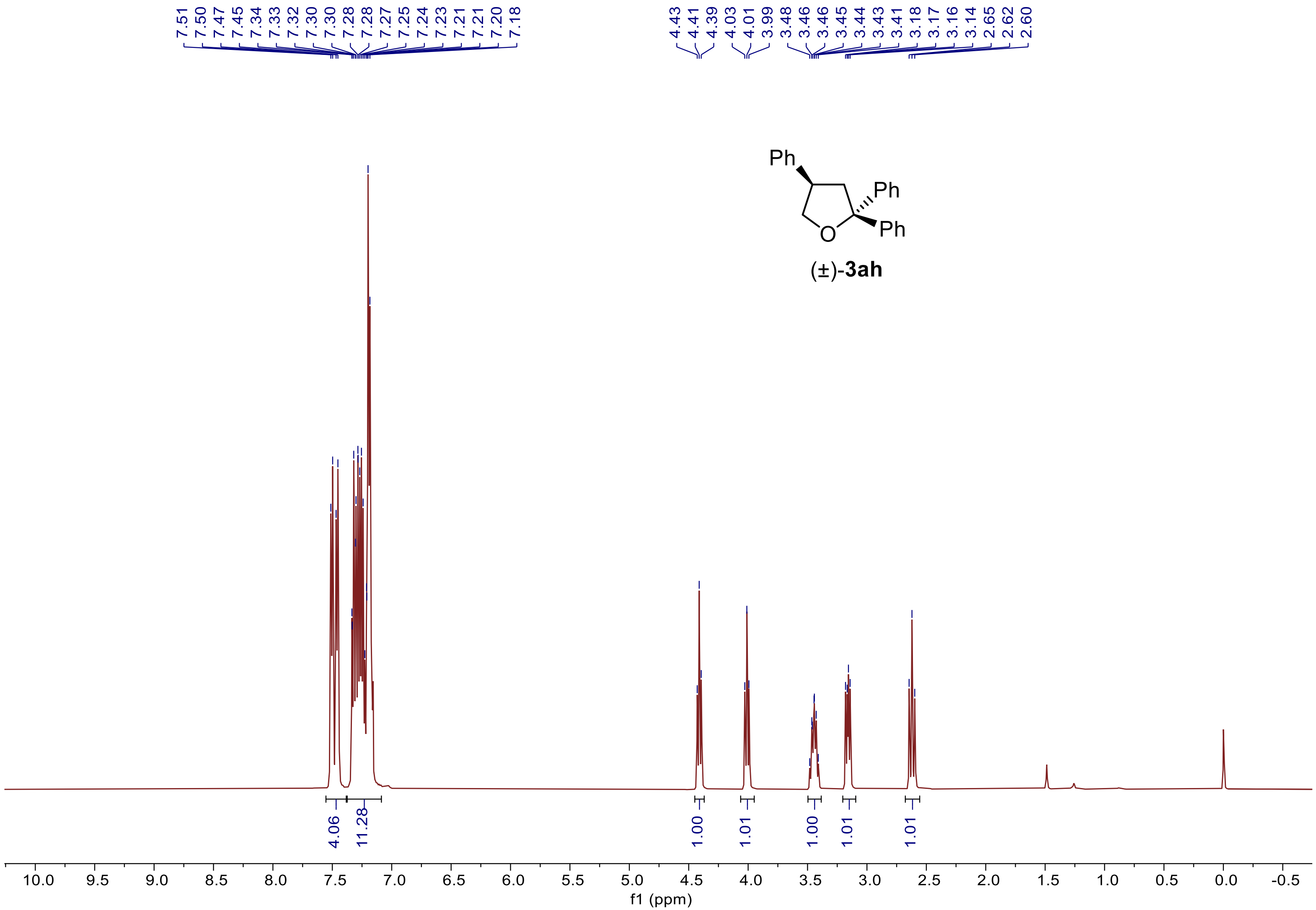

Figure S33. ^{1}H NMR Spectrum of (±)-**3ah** (500 MHz, $CDCl_3$).

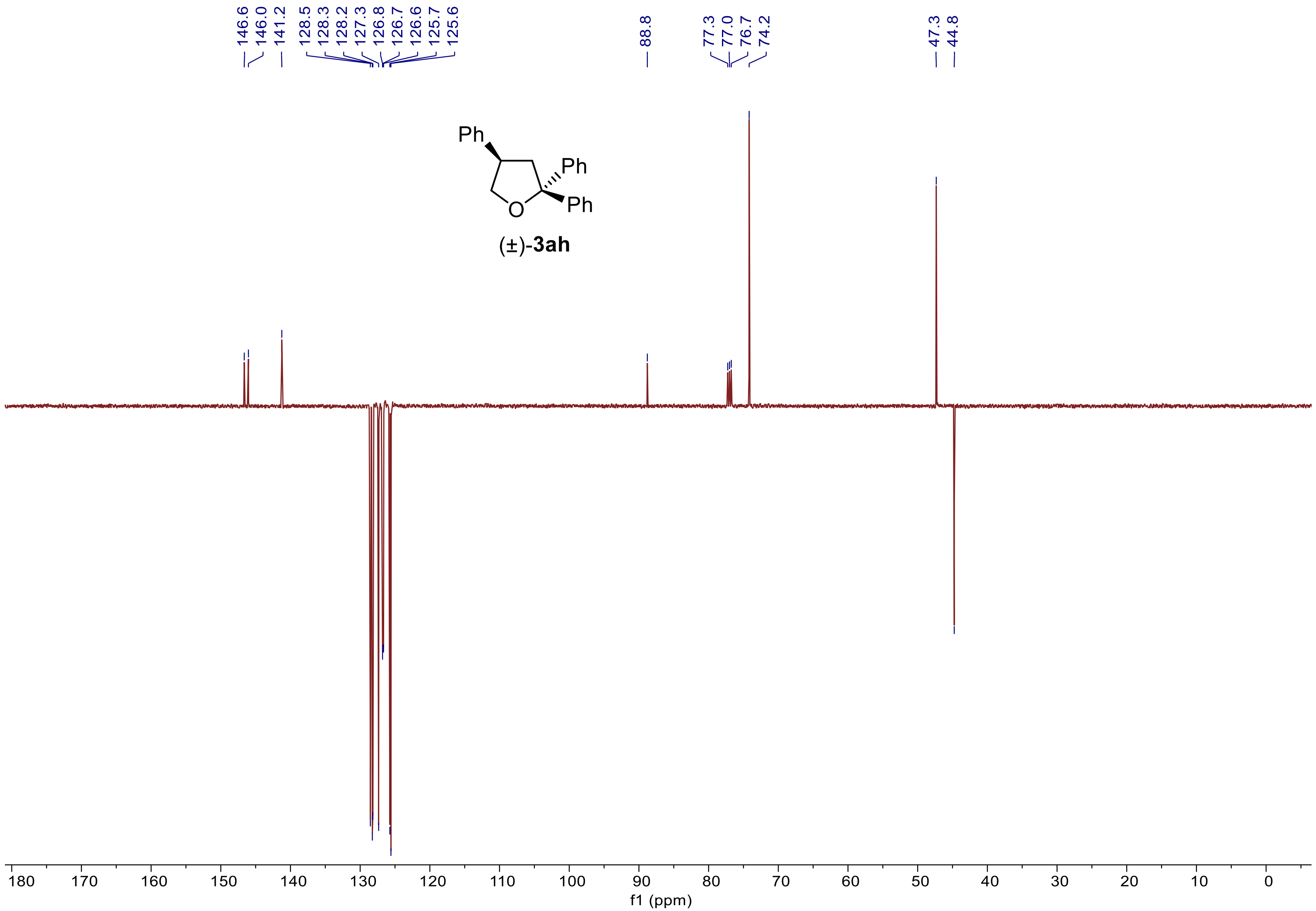

Figure S34. $^{13}C\{^1H\}$ NMR Spectrum of (±)-**3ah** (APT, 125 MHz, $CDCl_3$).

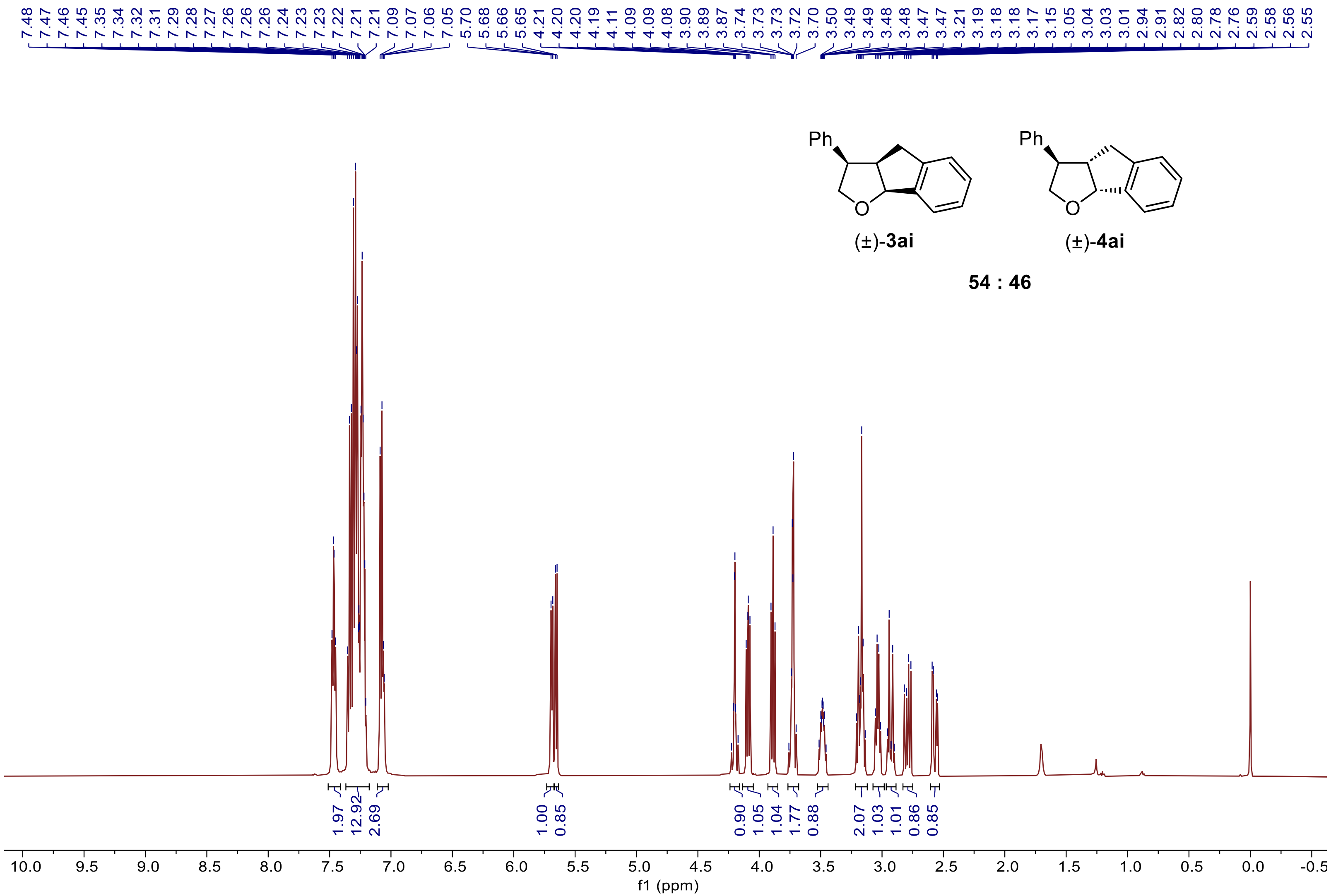

Figure S35. ^{1}H NMR Spectrum of (±)-**3ai** and (±)-4**ai** (500 MHz, $CDCl_3$).

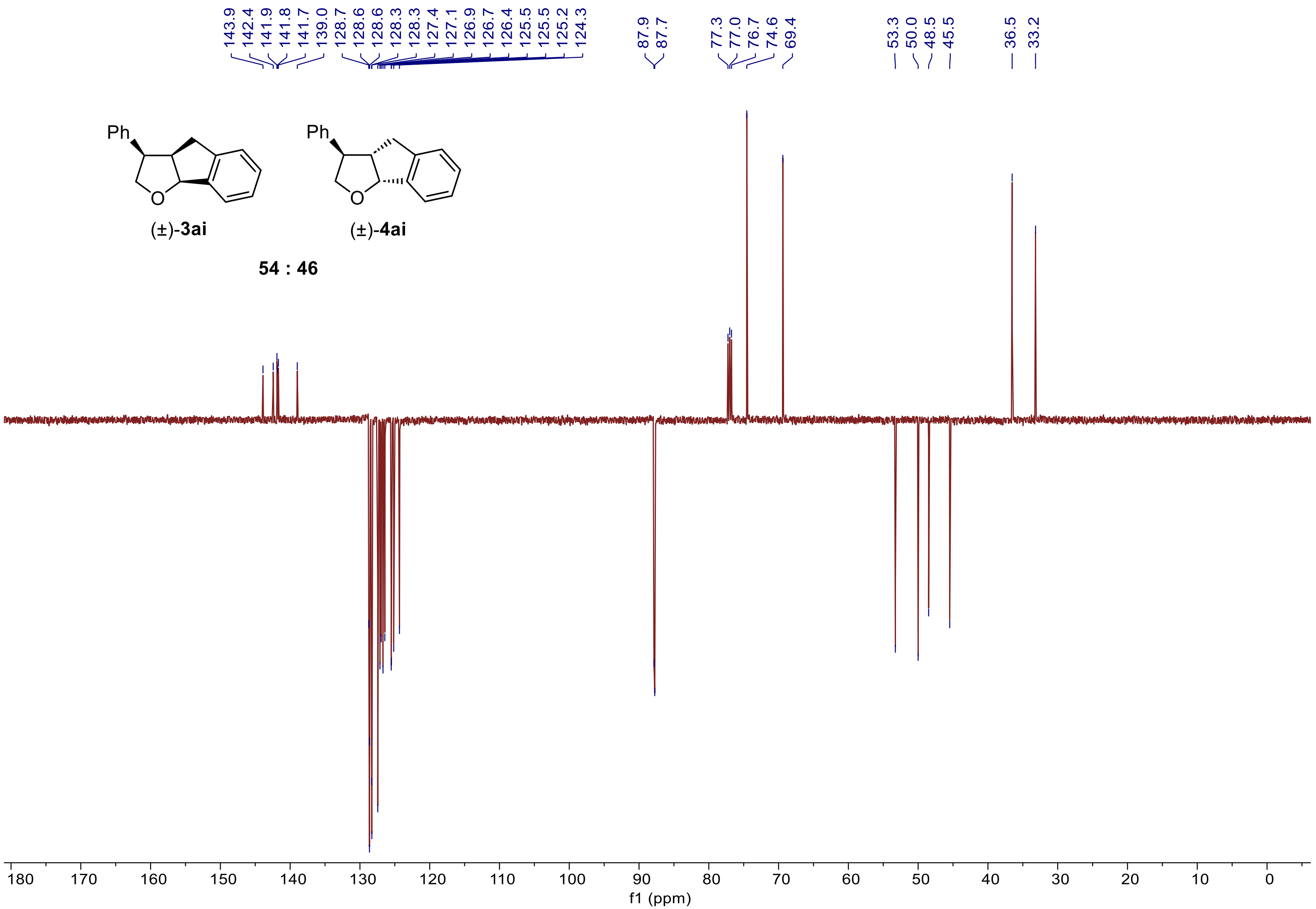

Figure S36. $^{13}C\{^1H\}$ NMR Spectrum of (±)-**3ai** and (±)-**4ai** (APT, 125 MHz, $CDCl_3$).

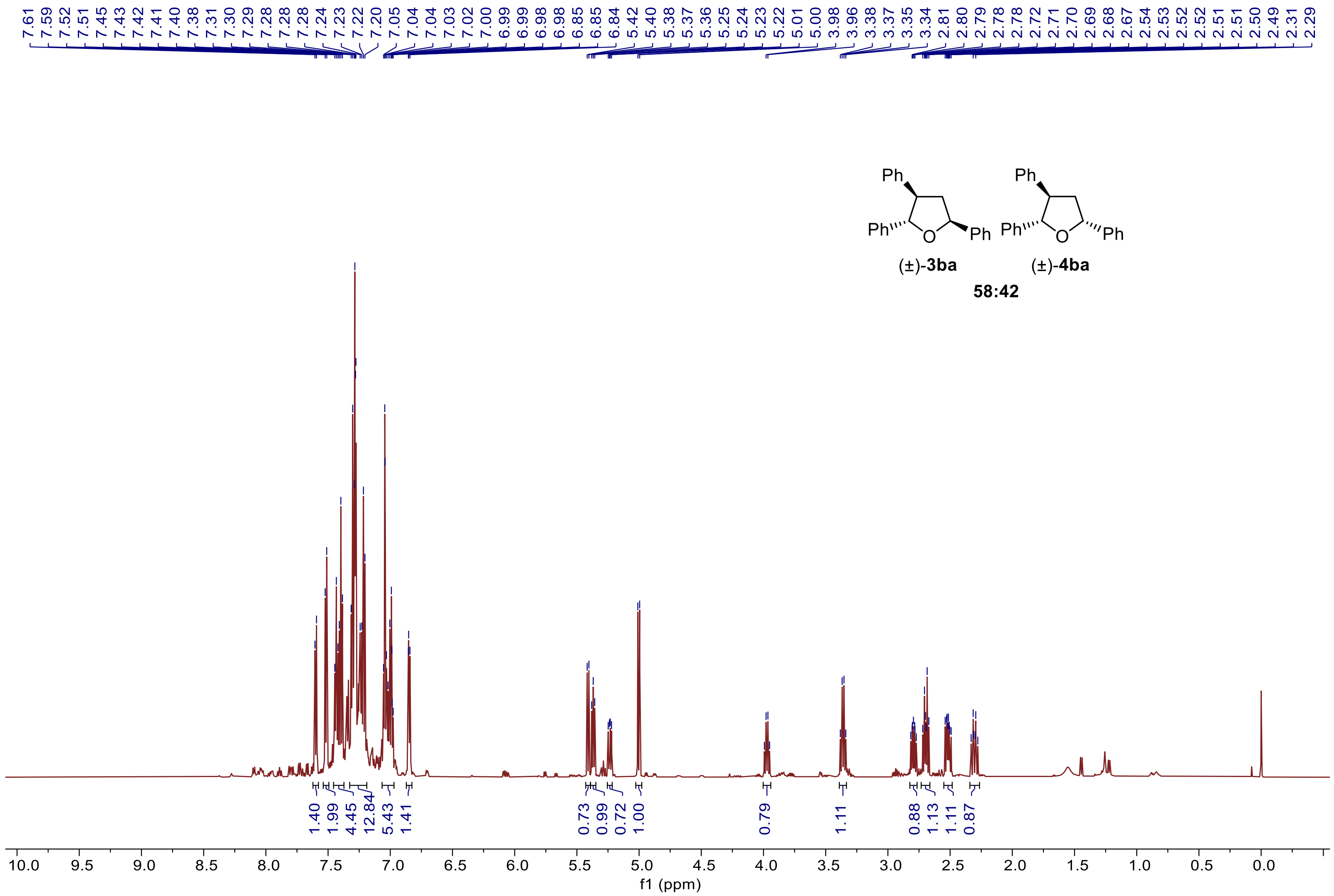

Figure S37. ^{1}H NMR Spectrum of (±)-**3ba** and (±)-**4ba** (600 MHz, $CDCl_3$).

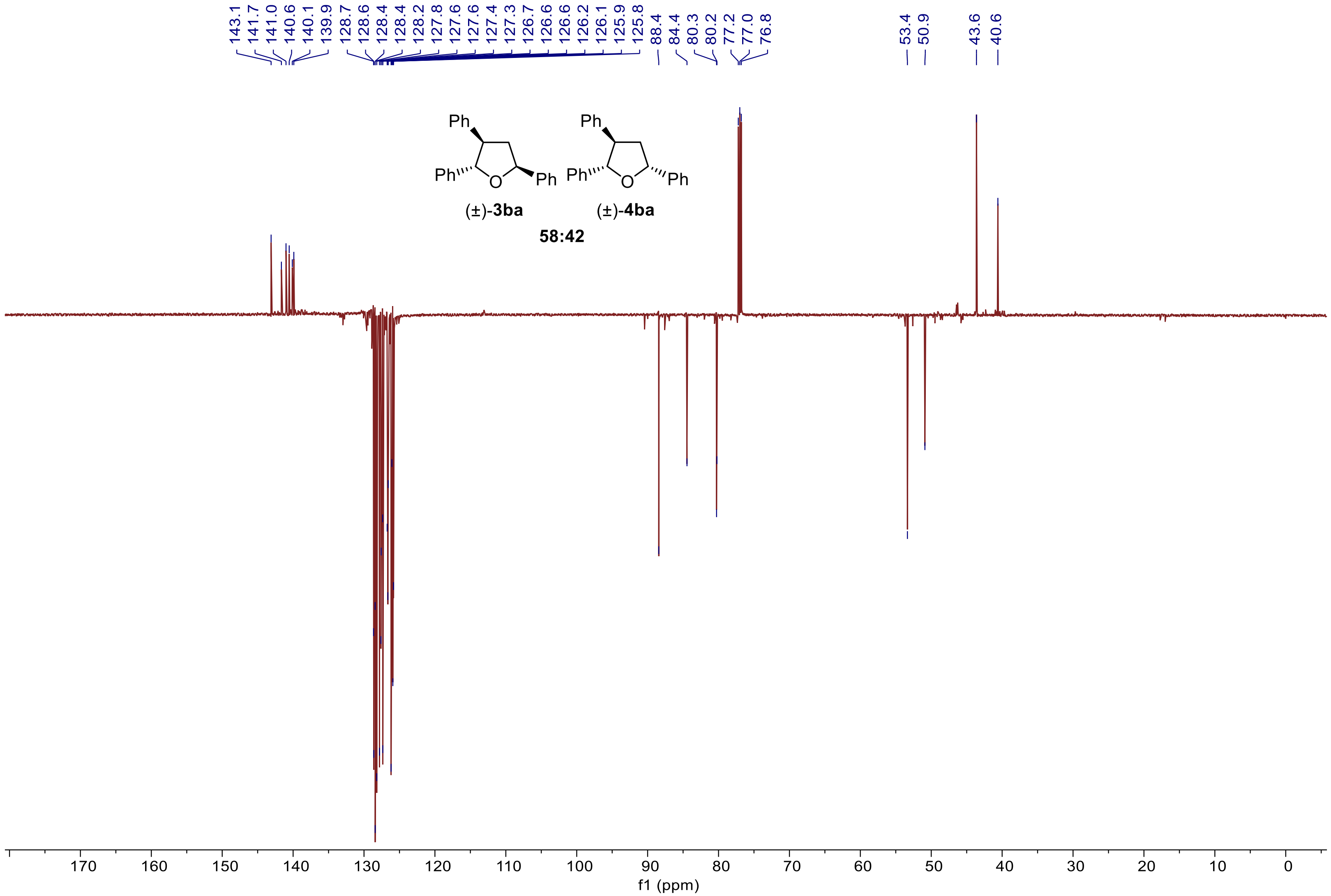

Figure S38. $^{13}C\{^{1}H\}$ NMR Spectrum of (±)-**3ba** and (±)-**4ba** (APT, 150 MHz, $CDCl_3$).

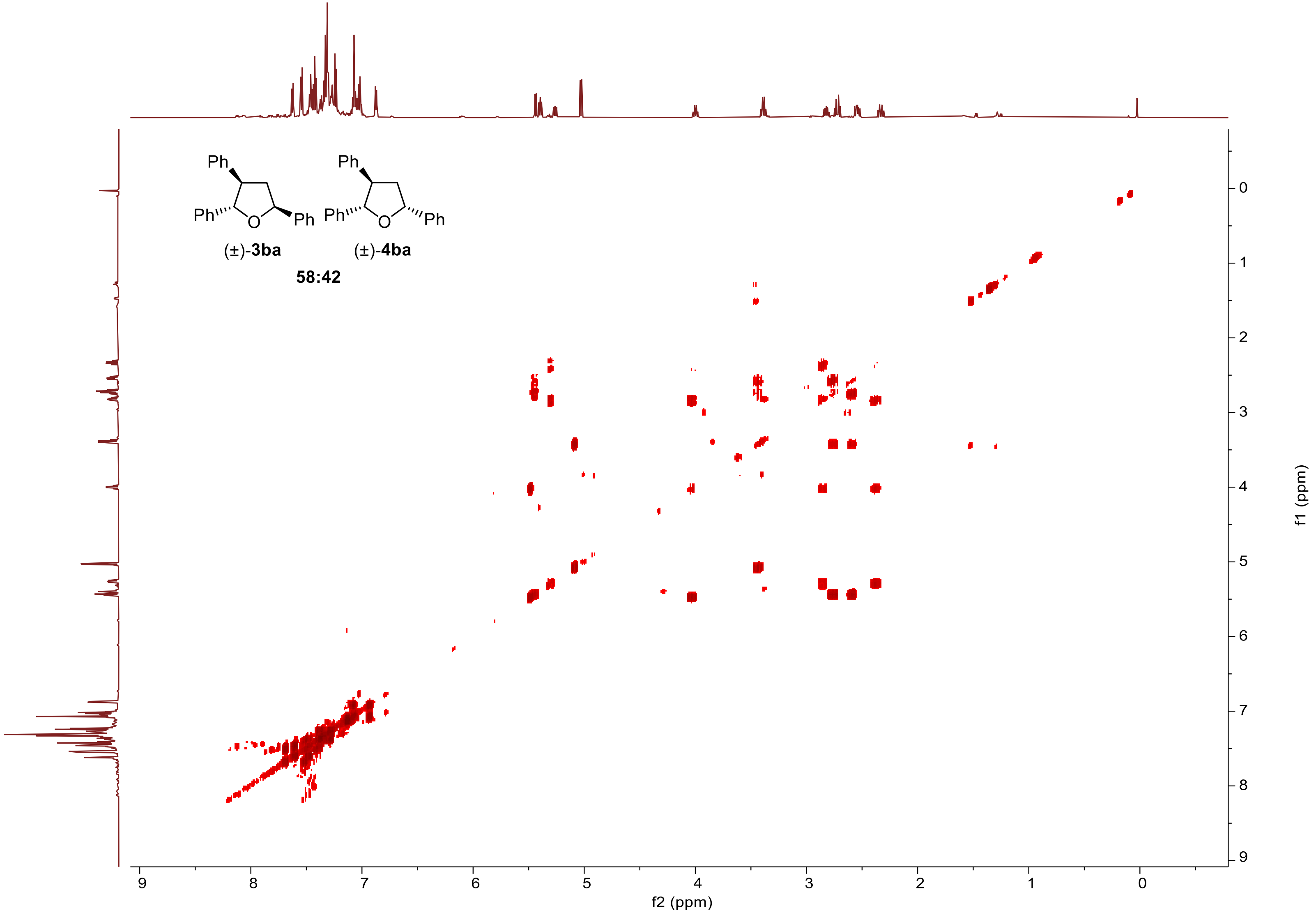

Figure S39. COSY NMR Spectrum of (±)-**3ba** and (±)-**4ba** ($CDCl_3$).

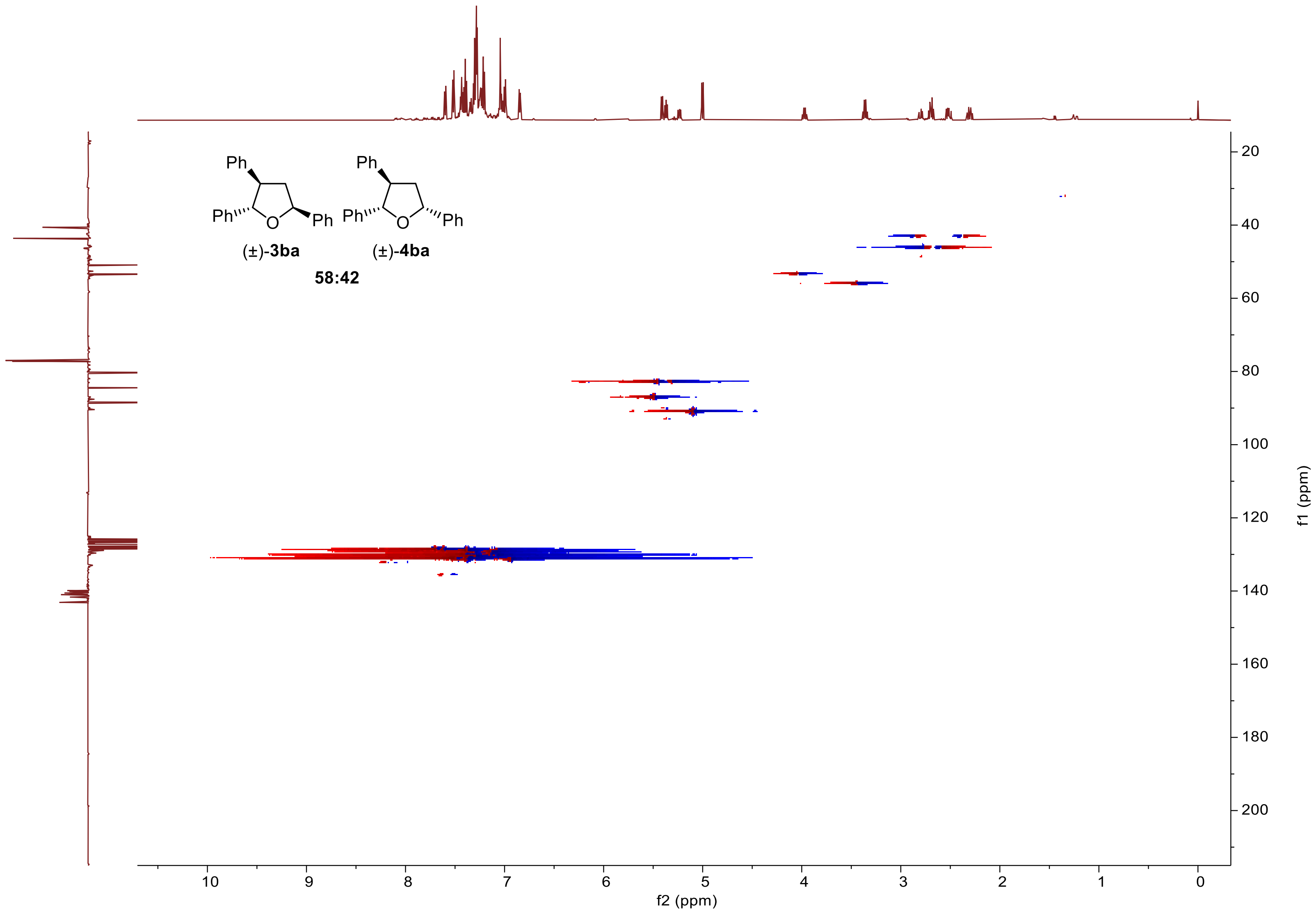

Figure S40. HSQC NMR Spectrum of (±)-**3ba** and (±)-**4ba** ($CDCl_3$).

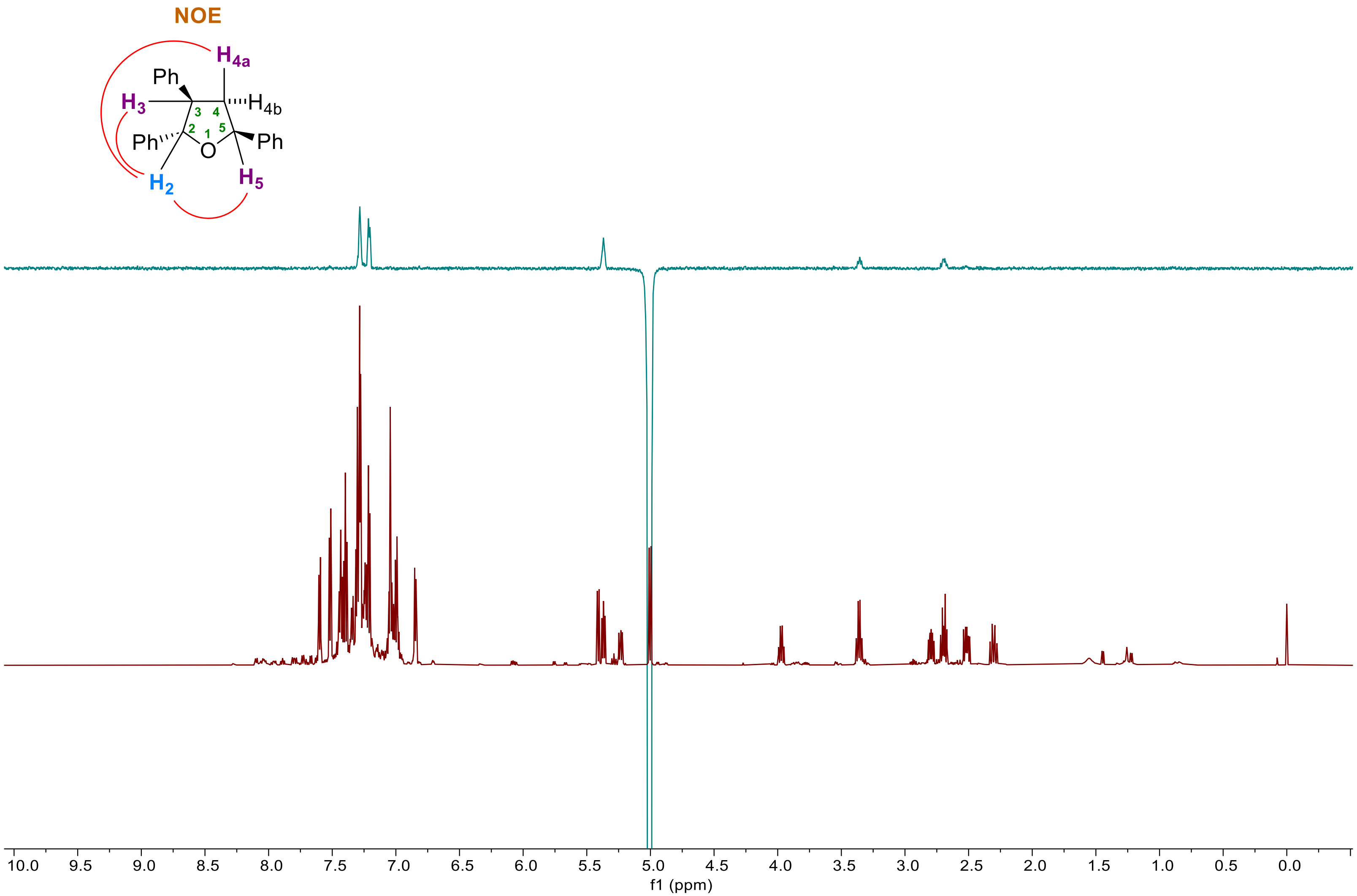

Figure S41. NOE-DIFF spectrum of the (±)-**3ba** and (±)-**4ba** ($CDCl_3$); increase in H-5, H-4a and H-3 signals after irradiation of the C***H*** (H-2) signal at 5.00 ppm.

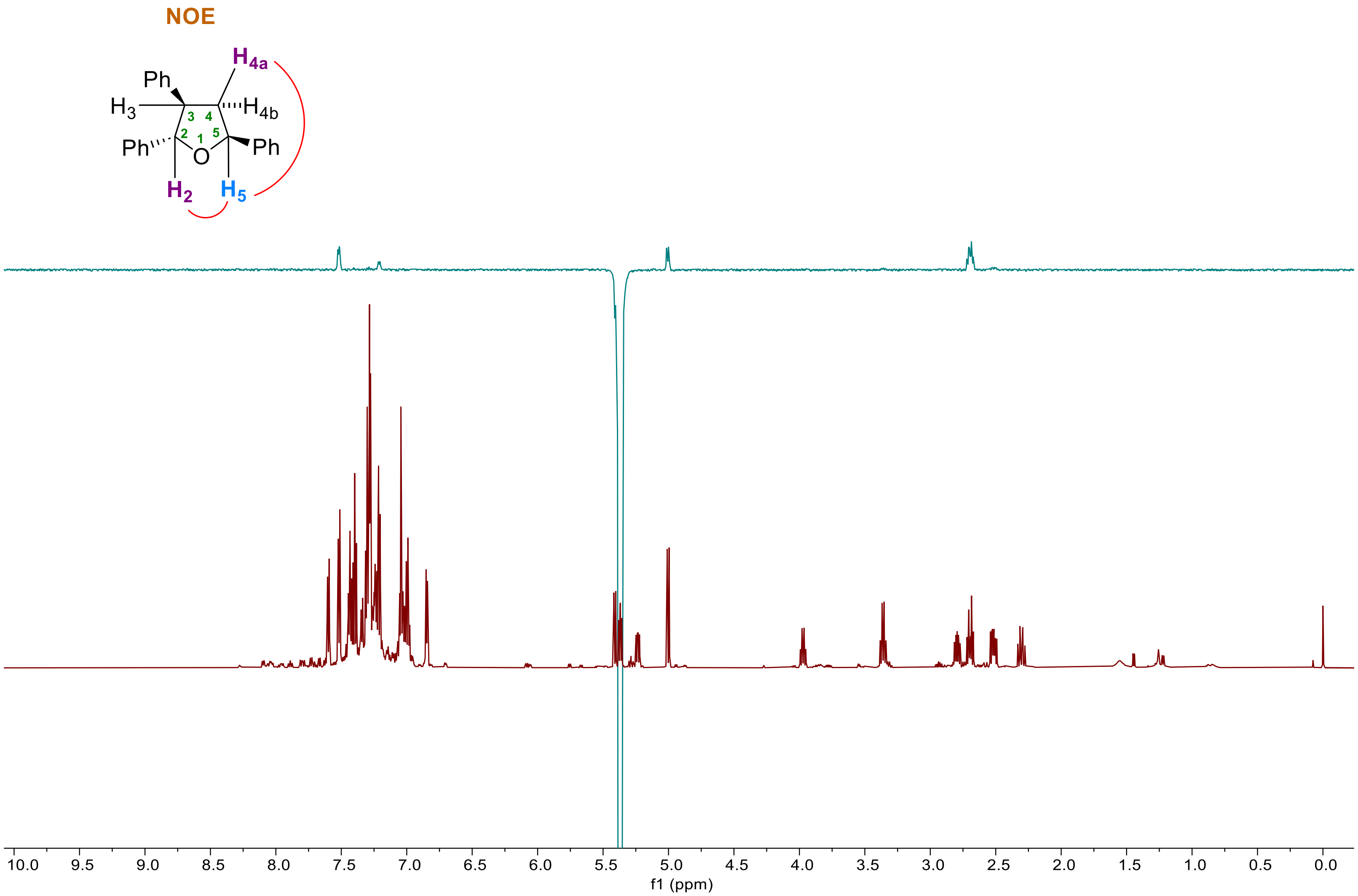

Figure S42. NOE-DIFF spectrum of the (±)-**3ba** and (±)-**4ba** ($CDCl_3$); increase in H-2 and H-4a signals after irradiation of the C***H*** (H-5) signal at 5.37 ppm.

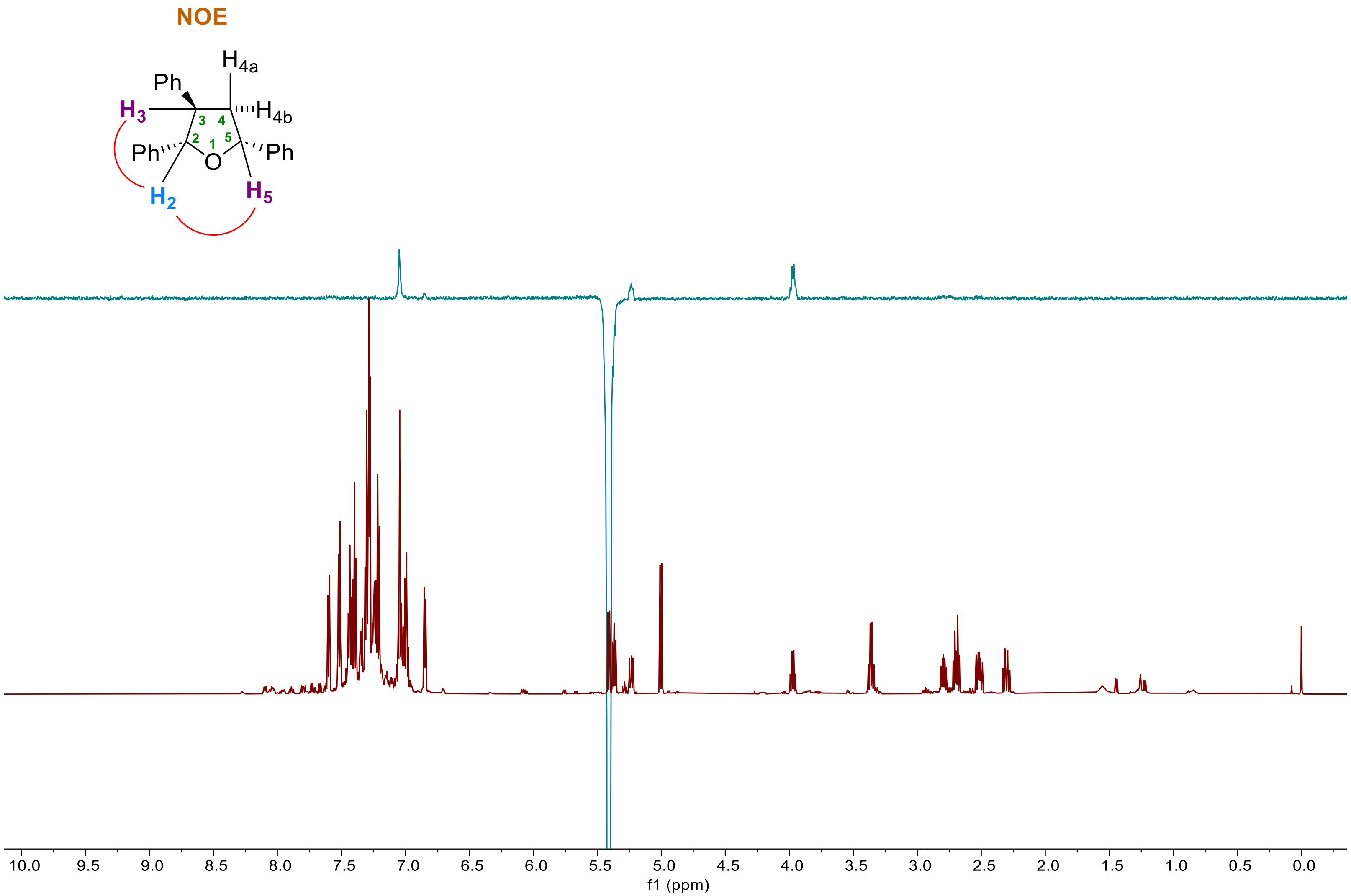

Figure S43. NOE-DIFF spectrum of the (±)-**3ba** and (±)-**4ba** ($CDCl_3$); increase in H-3 and H-5 signals after irradiation of the C***H*** (H-2) signal at 5.41 ppm.

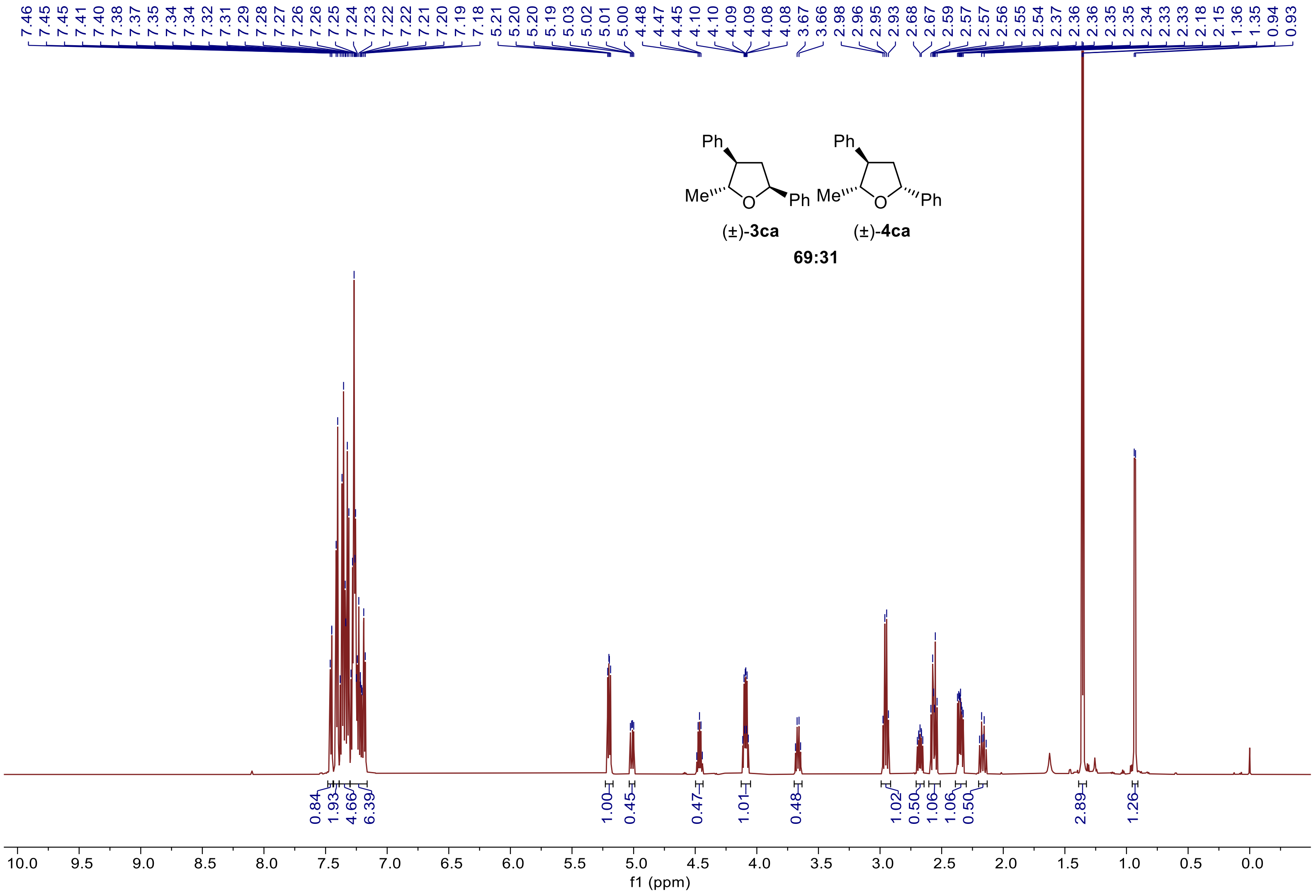

Figure S44. ^{1}H NMR Spectrum of (±)-**3ca** and (±)-**4ca** (600 MHz, $CDCl_3$).

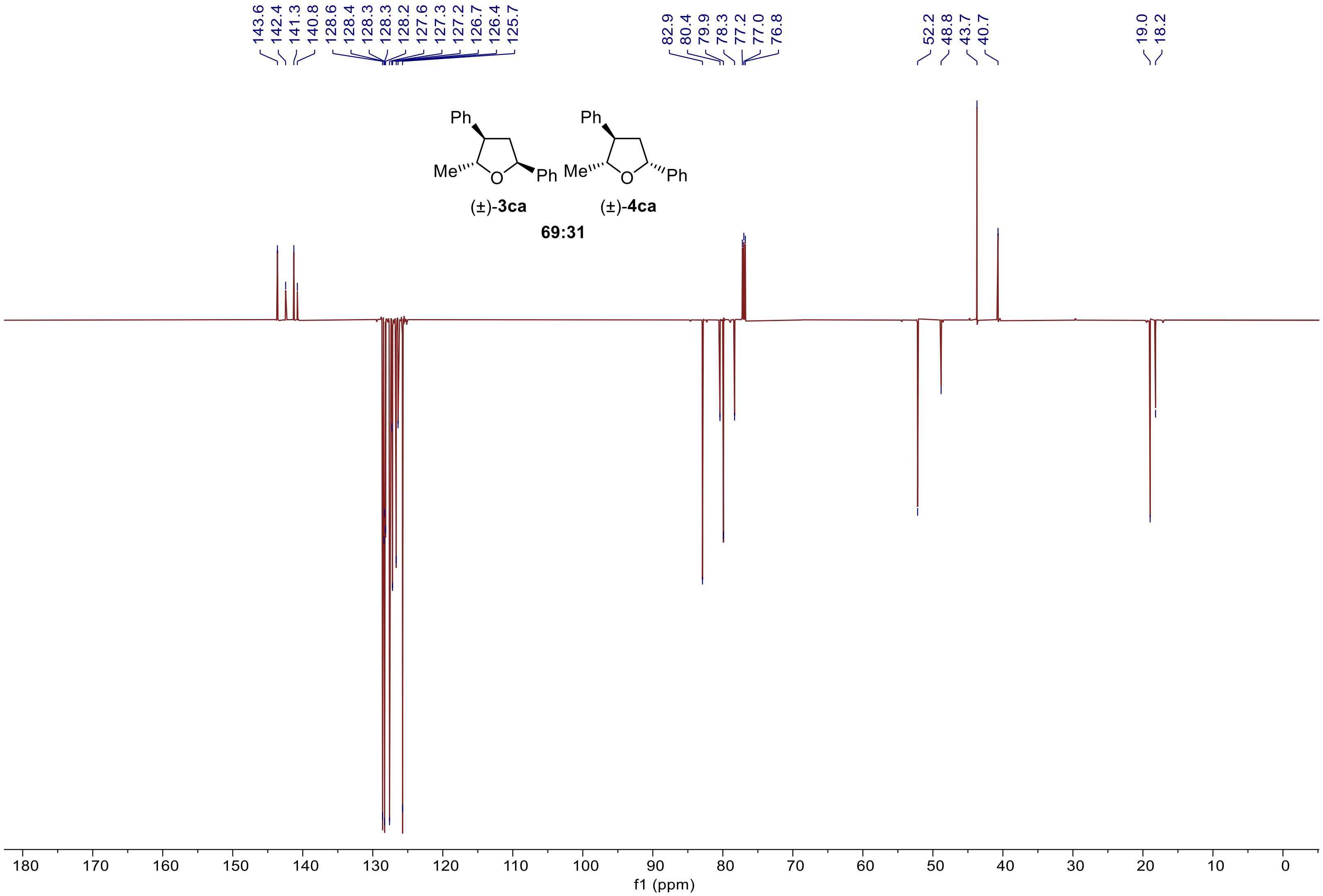

Figure S45. $^{13}C\{^{1}H\}$ NMR Spectrum of (±)-**3ca** and (±)-**4ca** (APT, 150 MHz, $CDCl_3$).

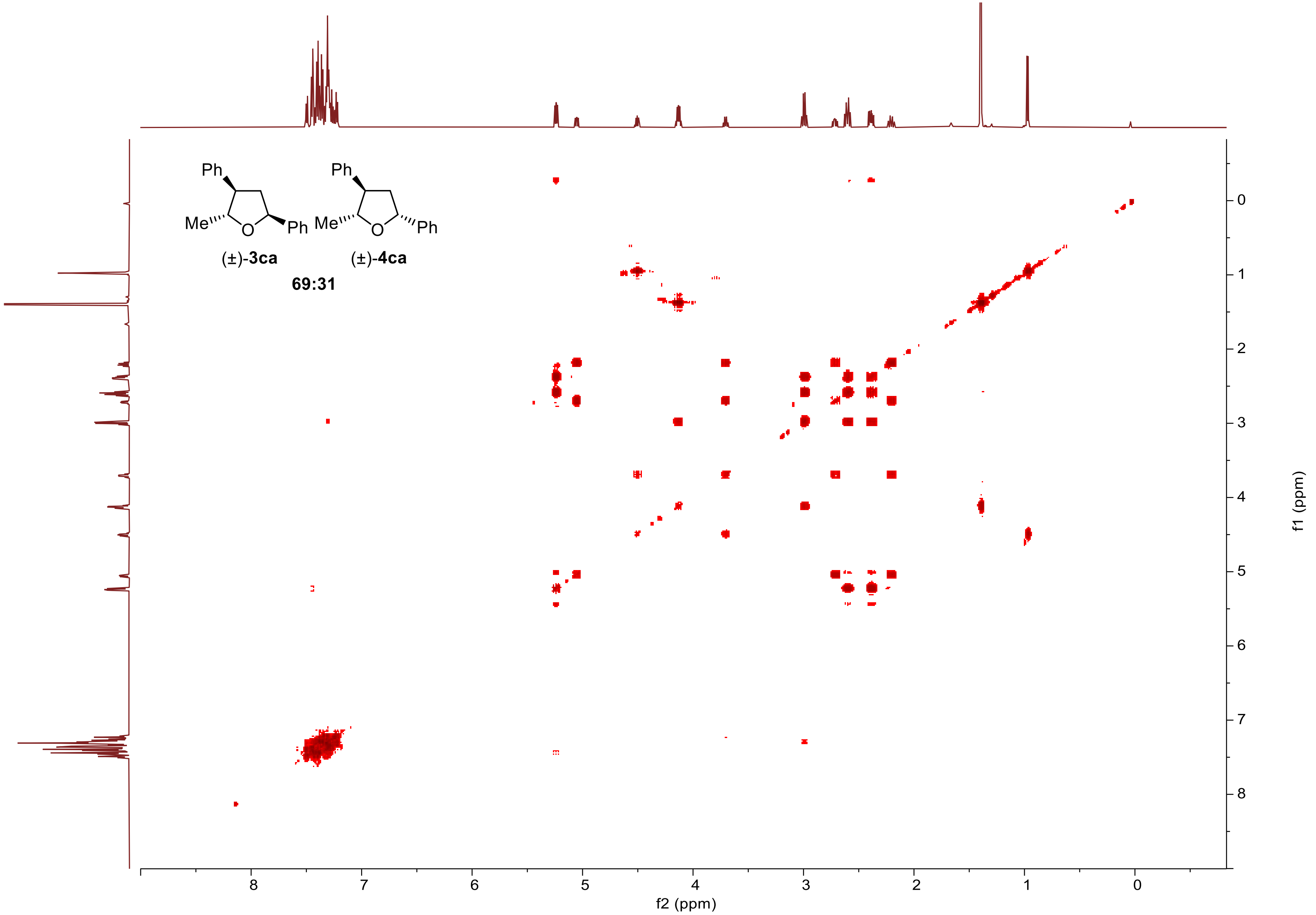

Figure S46. COSY NMR Spectrum of (±)-**3ca** and (±)-**4ca** ($CDCl_3$).

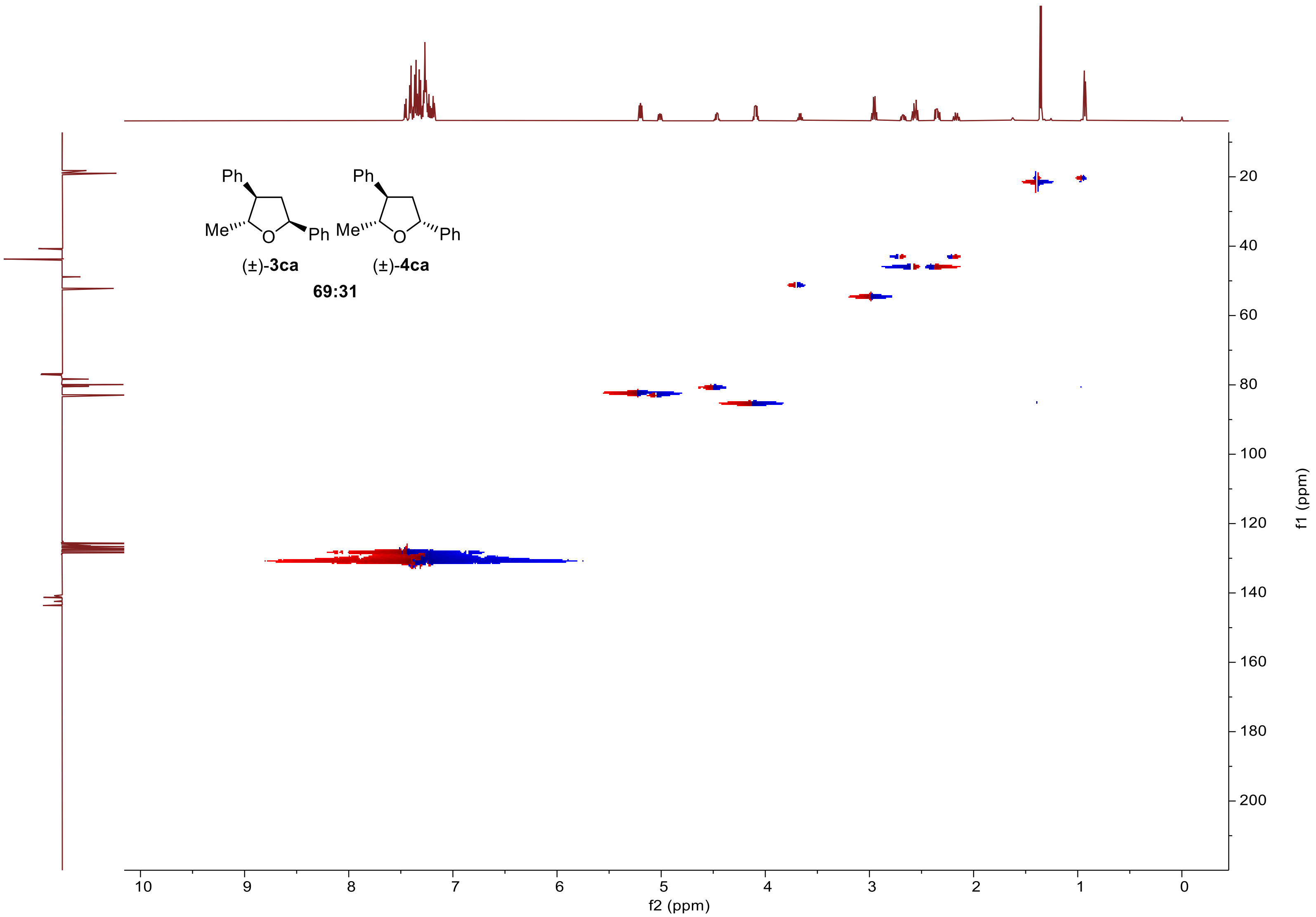

Figure S47. HSQC NMR Spectrum of (±)-**3ca** and (±)-**4ca** ($CDCl_3$).

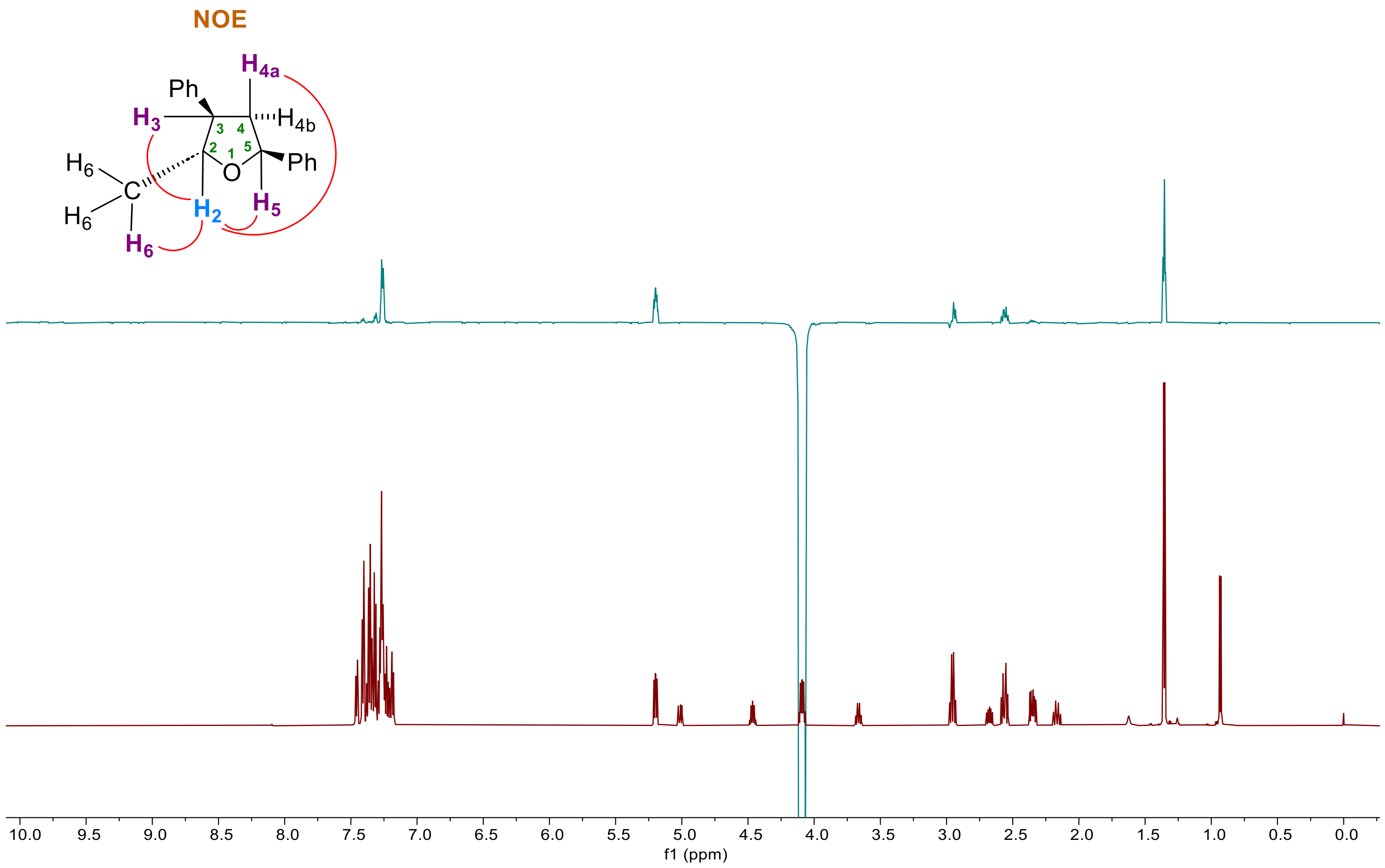

Figure S48. NOE-DIFF spectrum of the (±)-**3ca** and (±)-**4ca** ($CDCl_3$); increase in H-6, H-5, H-4a and H-3 signals after irradiation of the C***H*** (H-2) signal at 4.11-4.07 ppm.

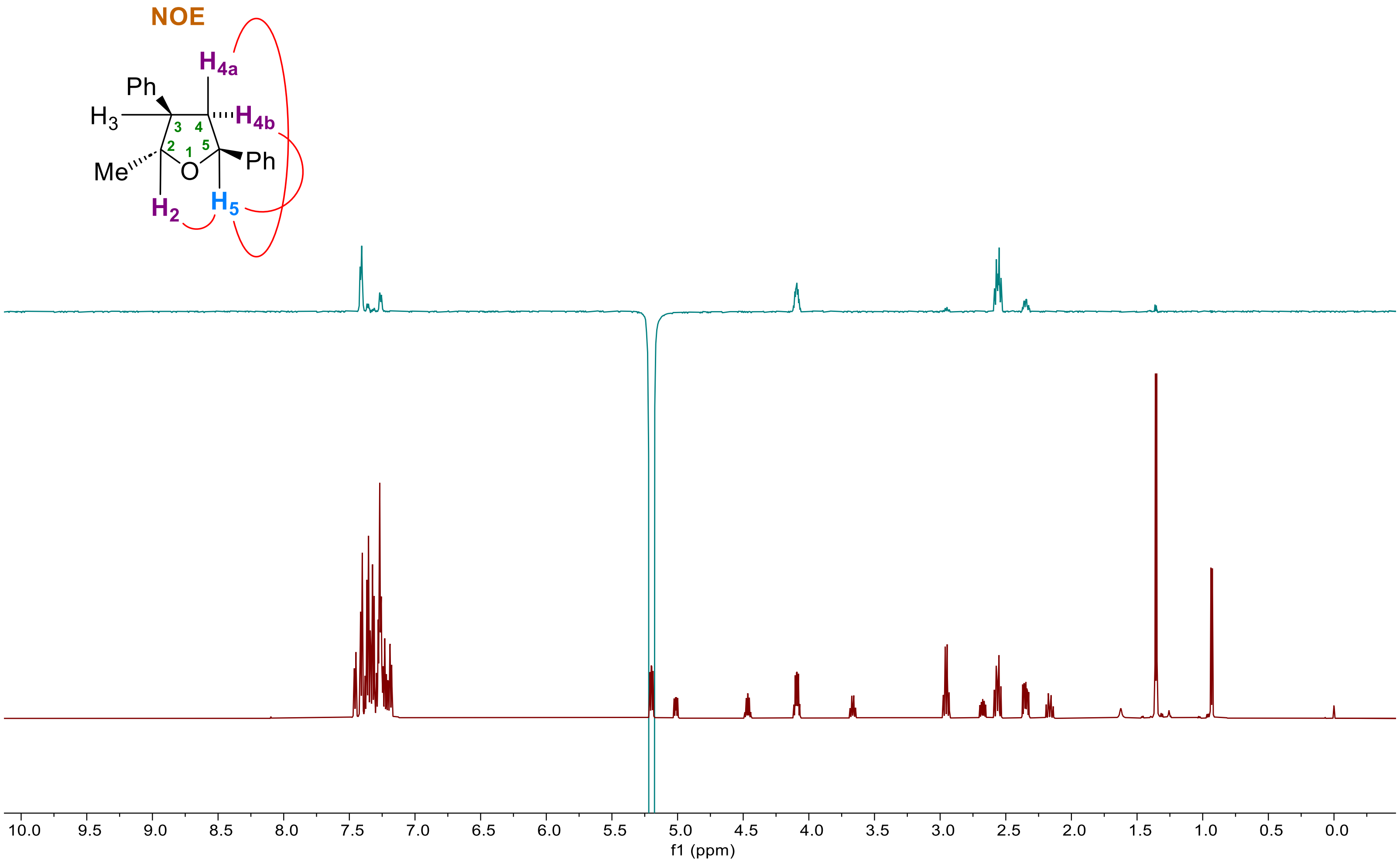

Figure S49. NOE-DIFF spectrum of the (±)-**3ca** and (±)-**4ca** ($CDCl_3$); increase in H-4a, H-4b and H-2 signals after irradiation of the C***H*** (H-5) signal at 5.20 ppm.

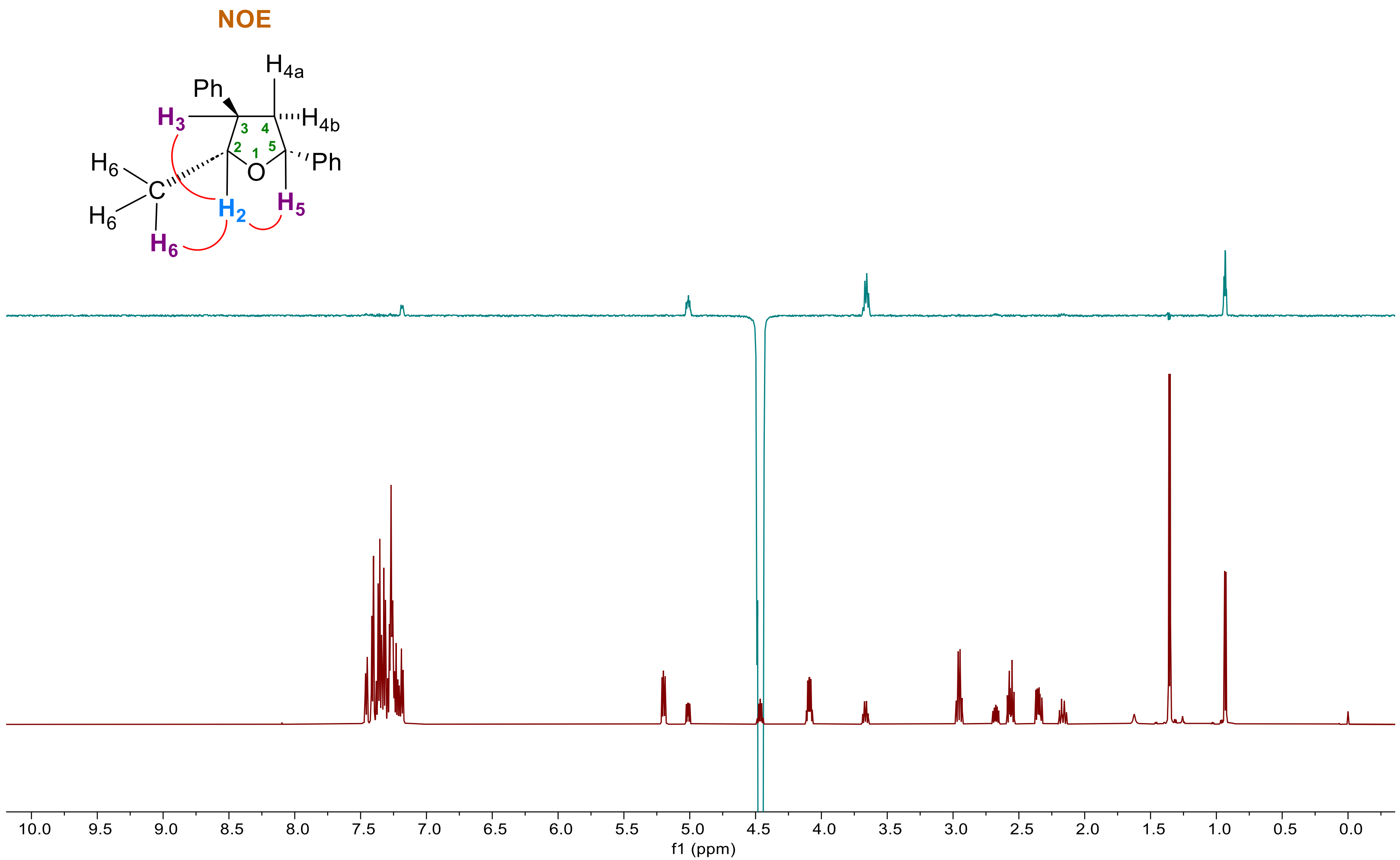

Figure S50. NOE-DIFF spectrum of the (±)-**3ca** and (±)-**4ca** ($CDCl_3$); increase in H-6, H-5 and H-3 signals after irradiation of the C***H*** (H-2) signal at 4.49-4.44 ppm.

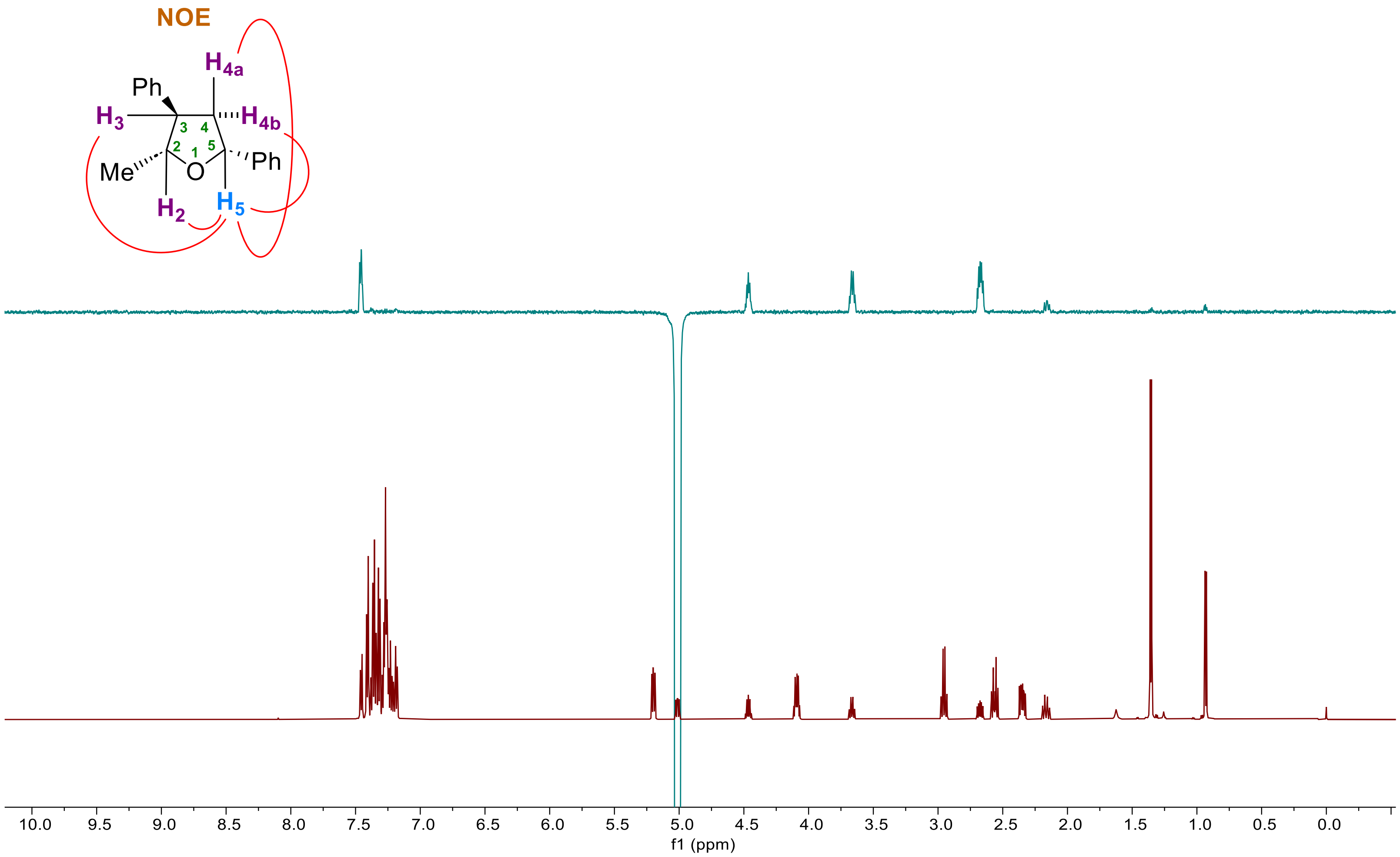

Figure S51. NOE-DIFF spectrum of the (±)-**3ca** and (±)-**4ca** ($CDCl_3$); increase in H-4a, H-4b, H-3 and H-2 signals after irradiation of the C***H*** (H-5) signal at 5.01 ppm.

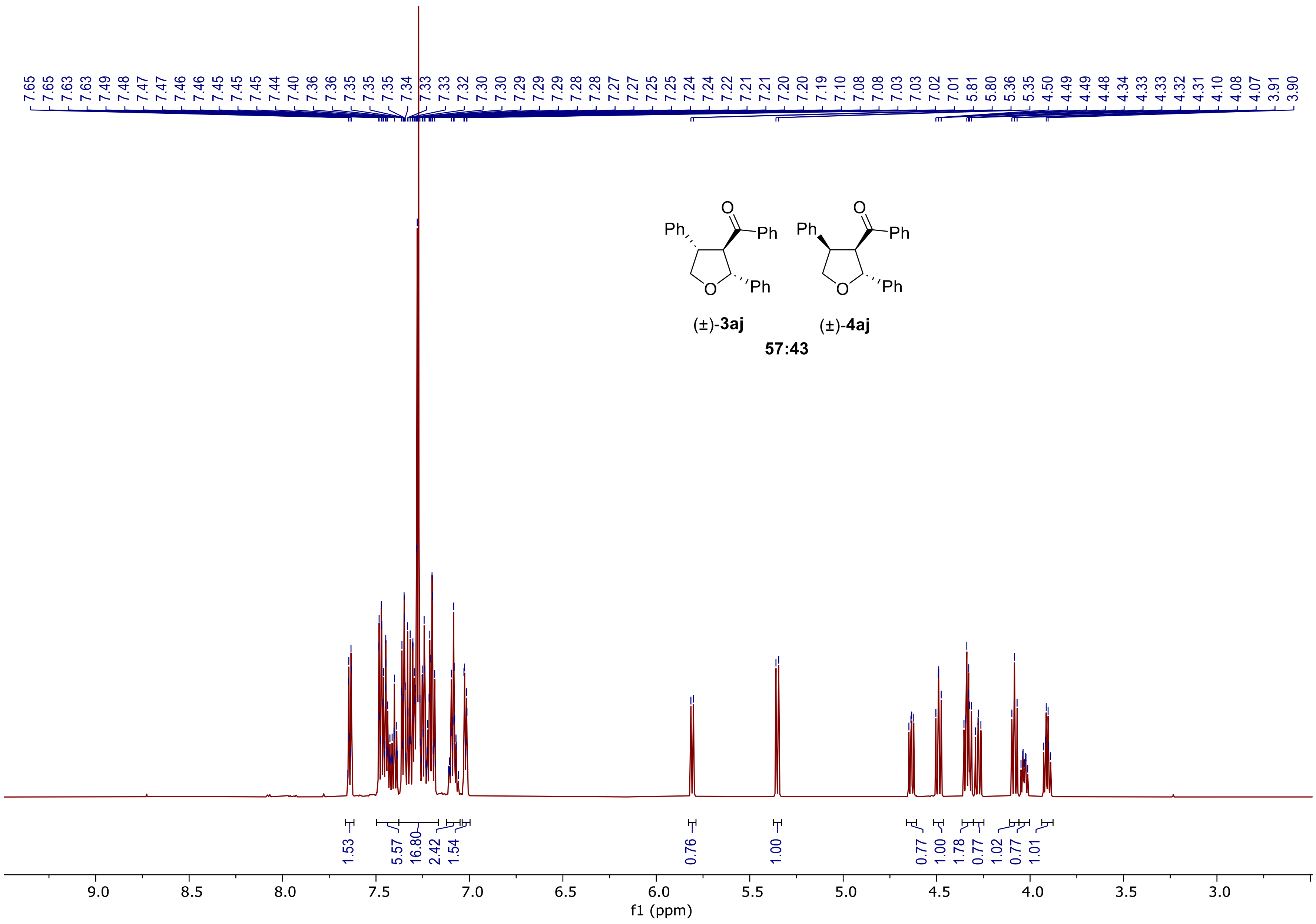

Figure S52. ^{1}H NMR Spectrum of (±)-**3aj** and (±)-**4aj** (600 MHz, $CDCl_3$).

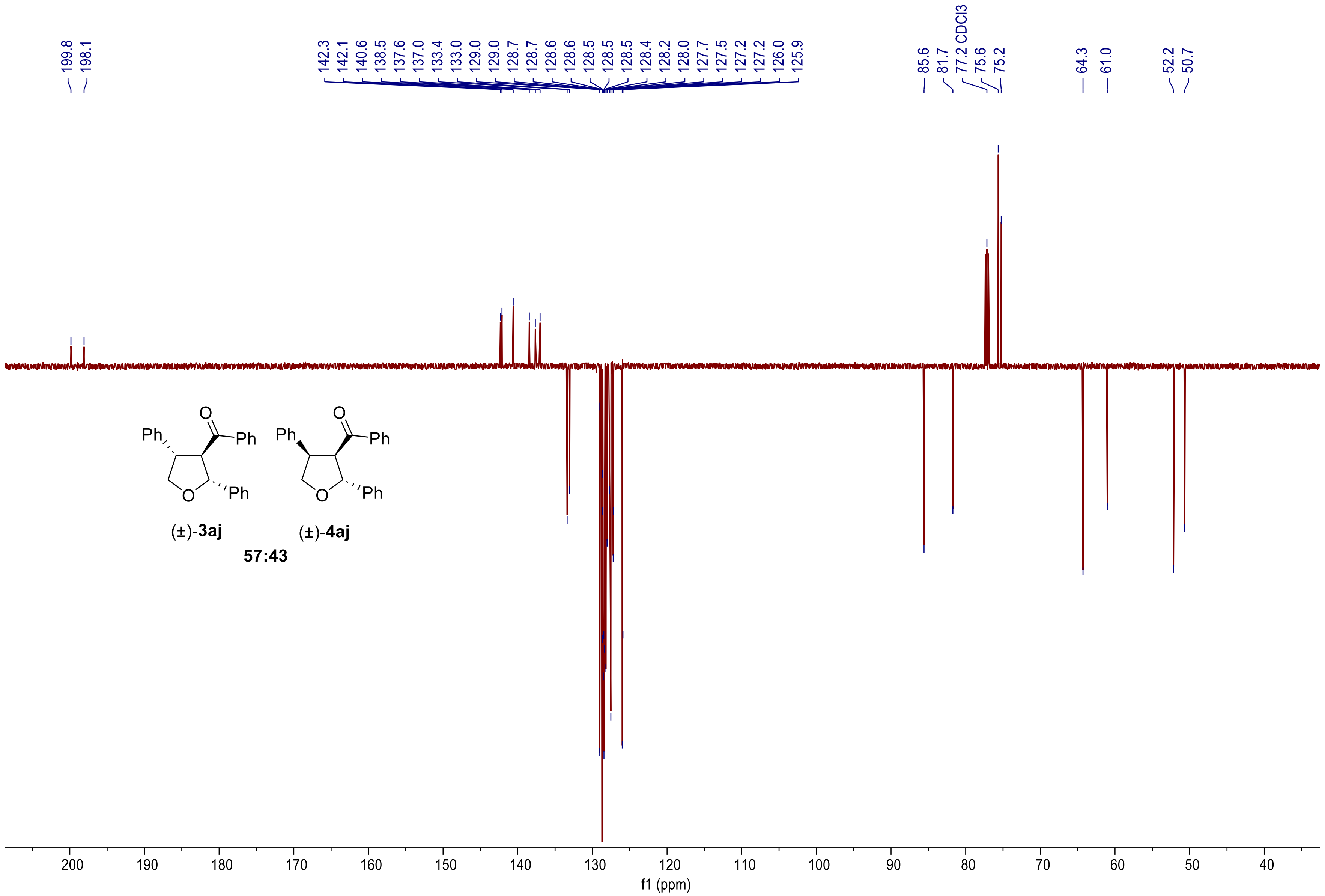

Figure S53. $^{13}C\{^{1}H\}$ NMR Spectrum of (±)-**3aj** and (±)-**4aj** (APT, 150 MHz, $CDCl_3$).

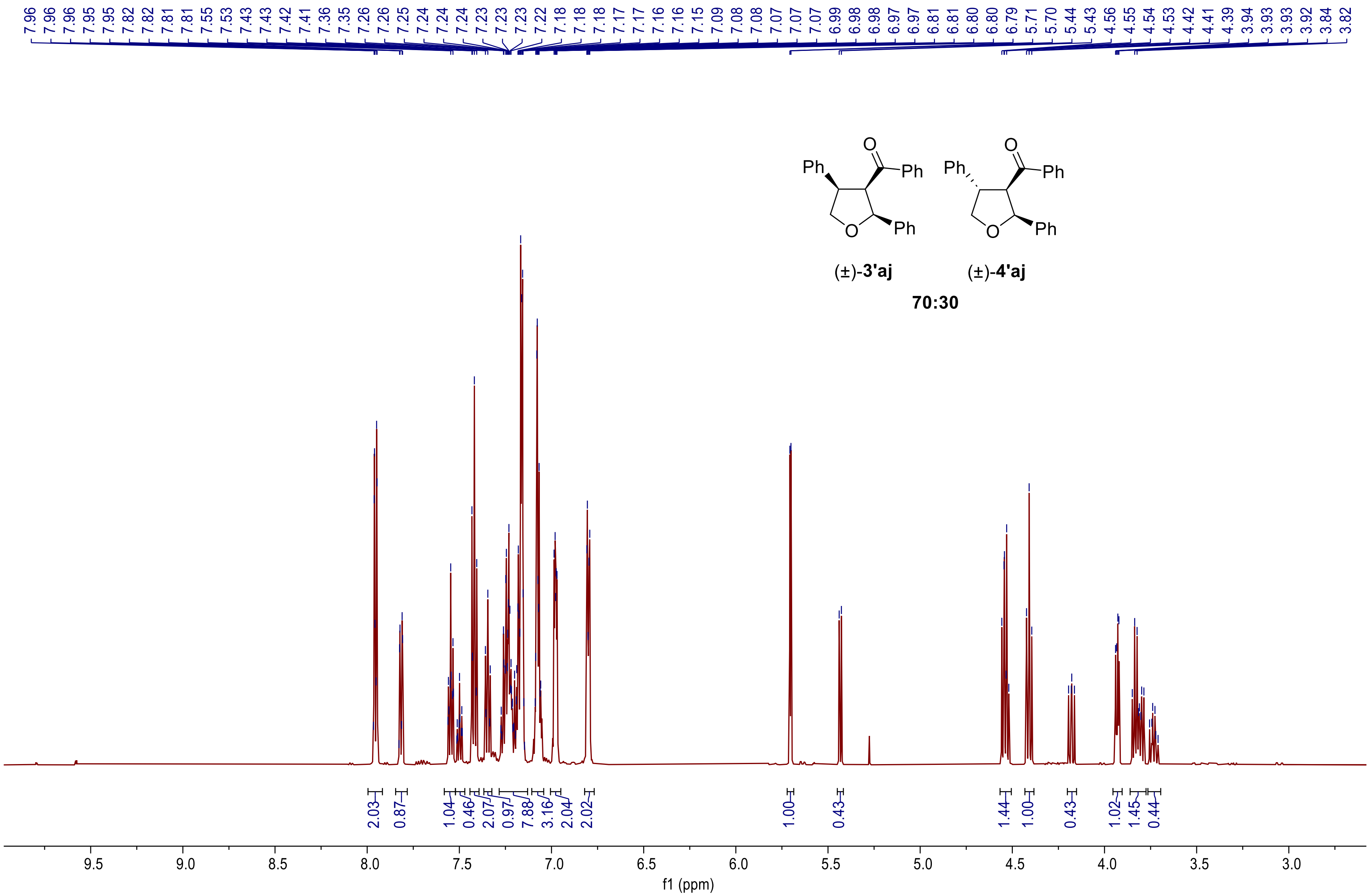

Figure S54. ^{1}H NMR Spectrum of (±)-**3'aj** and (±)-**4'aj** (600 MHz, $CDCl_3$).

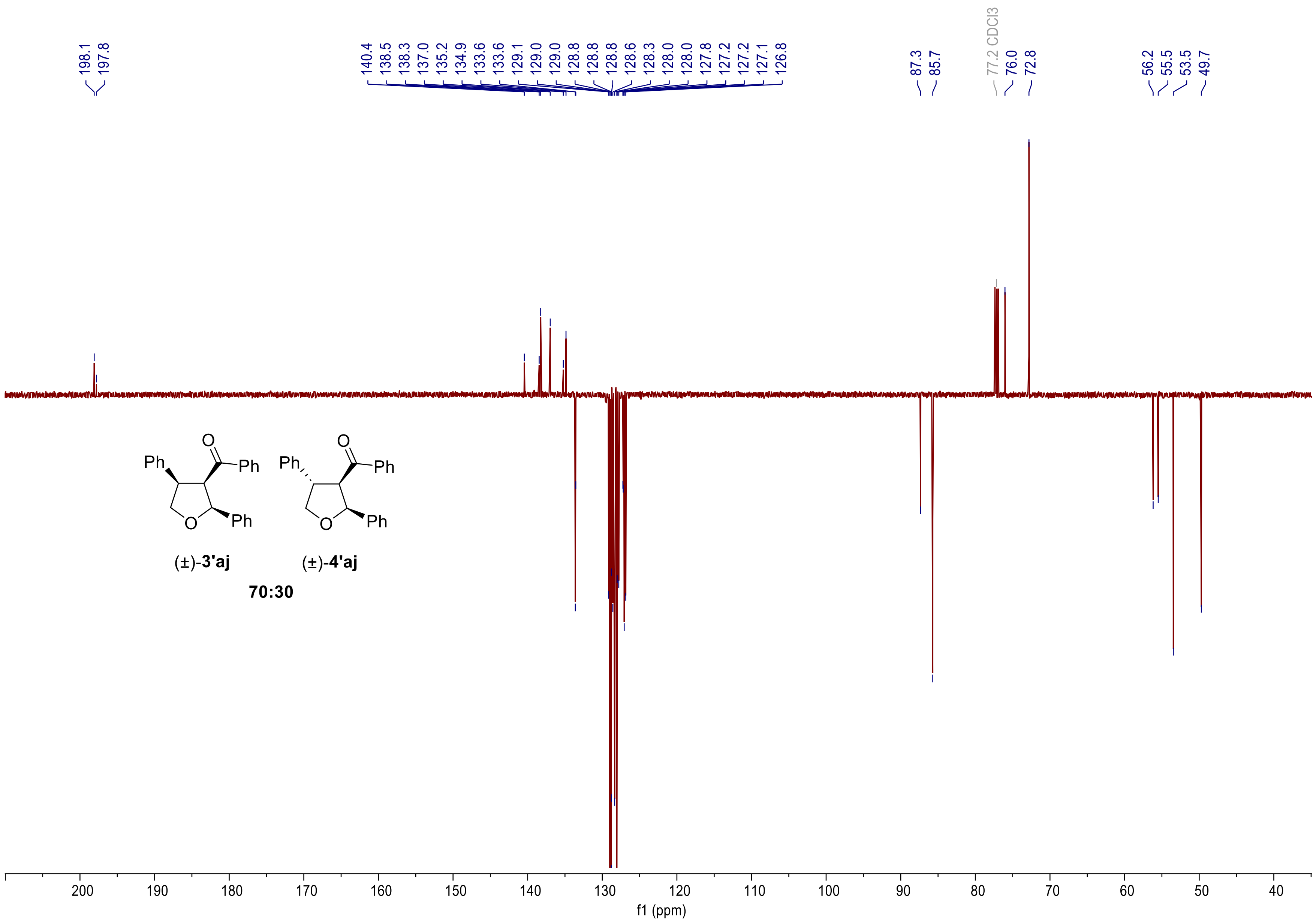

Figure S55. $^{13}C\{^{1}H\}$ NMR Spectrum of (±)-**3'aj** and (±)-**4'aj** (APT, 150 MHz, $CDCl_3$).

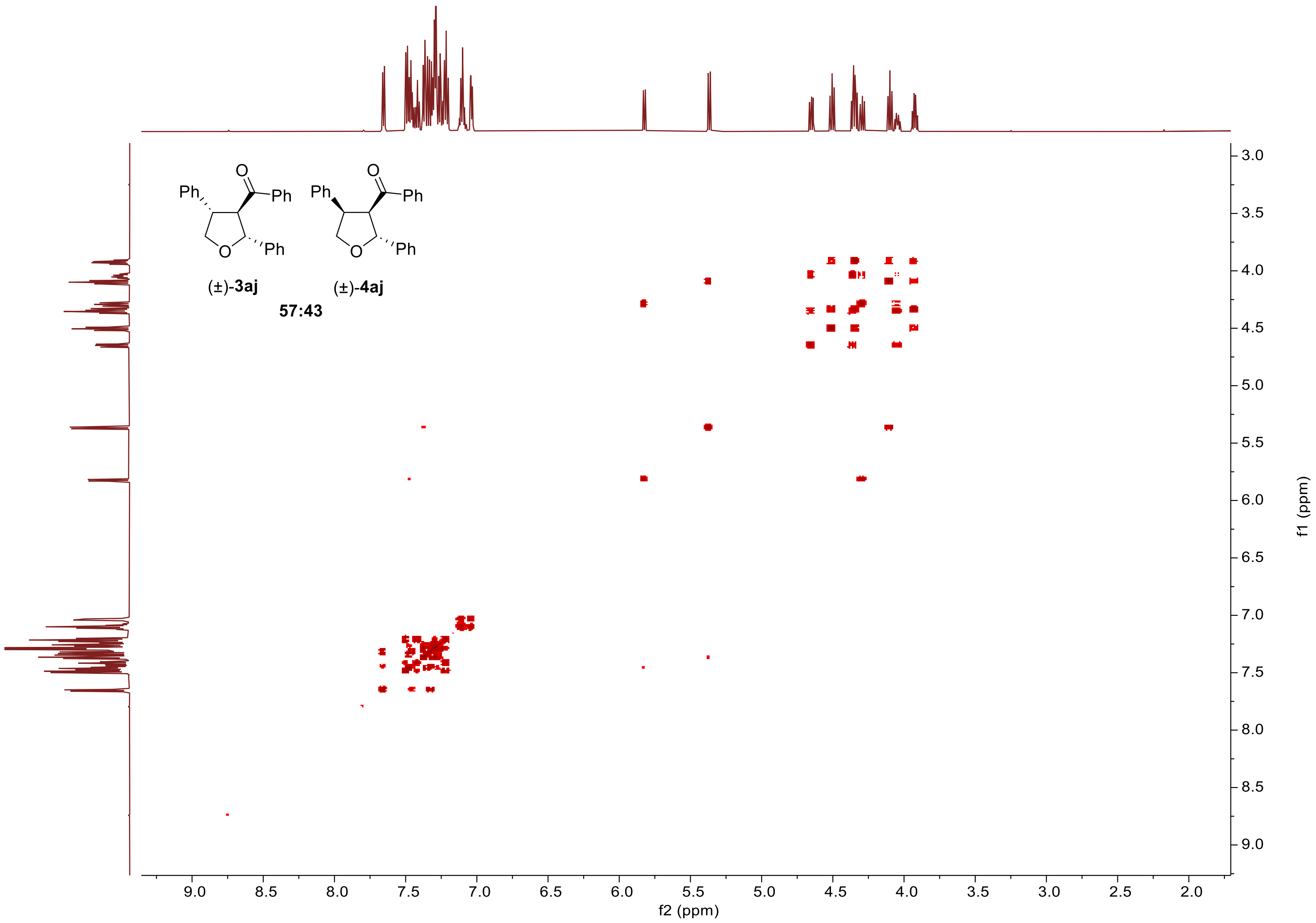

Figure S56. COSY NMR Spectrum of (±)-**3aj** and (±)-**4aj** ($CDCl_3$).

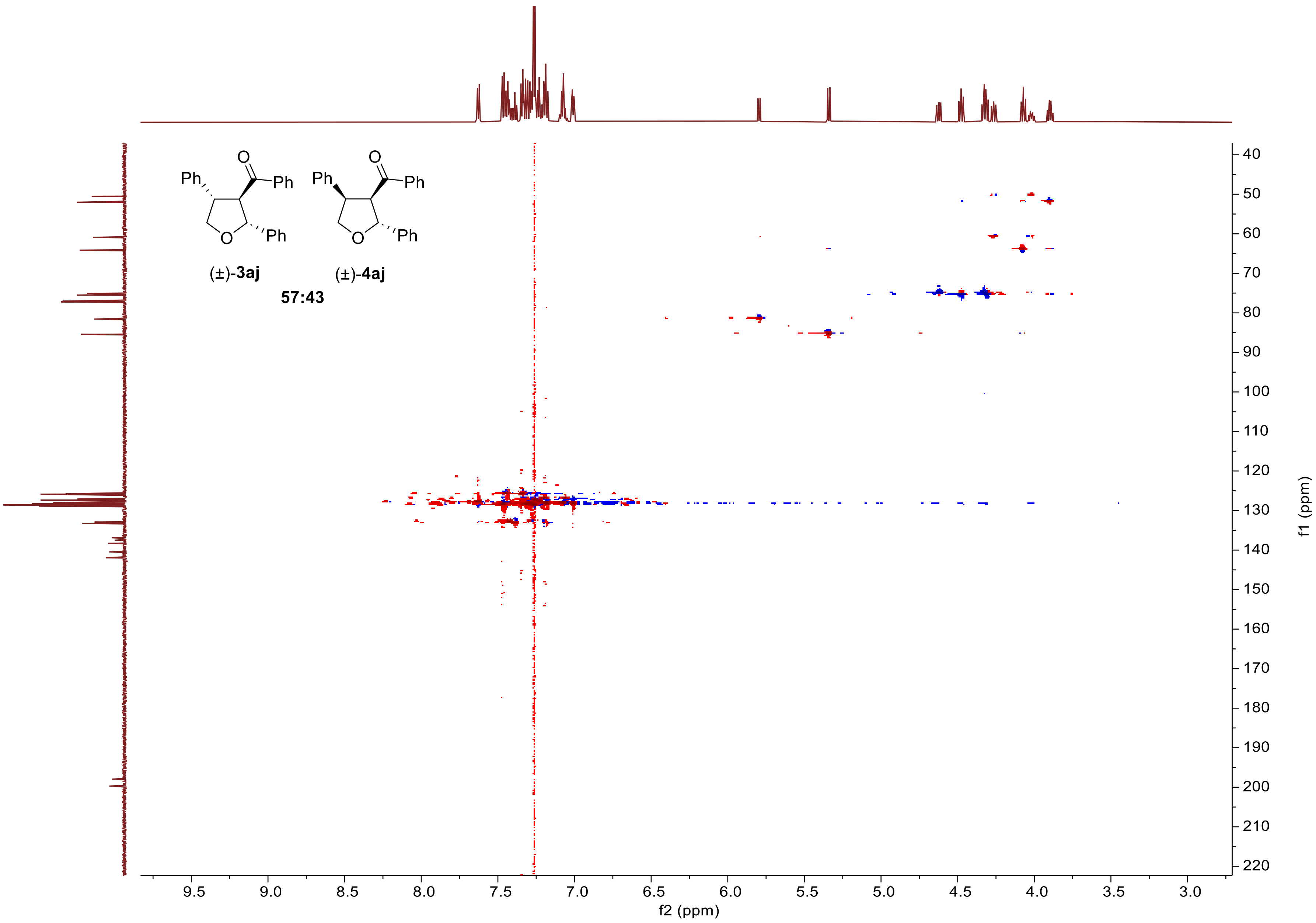

Figure S57. HSQC NMR Spectrum of (±)-**3aj** and (±)-**4aj** ($CDCl_3$).

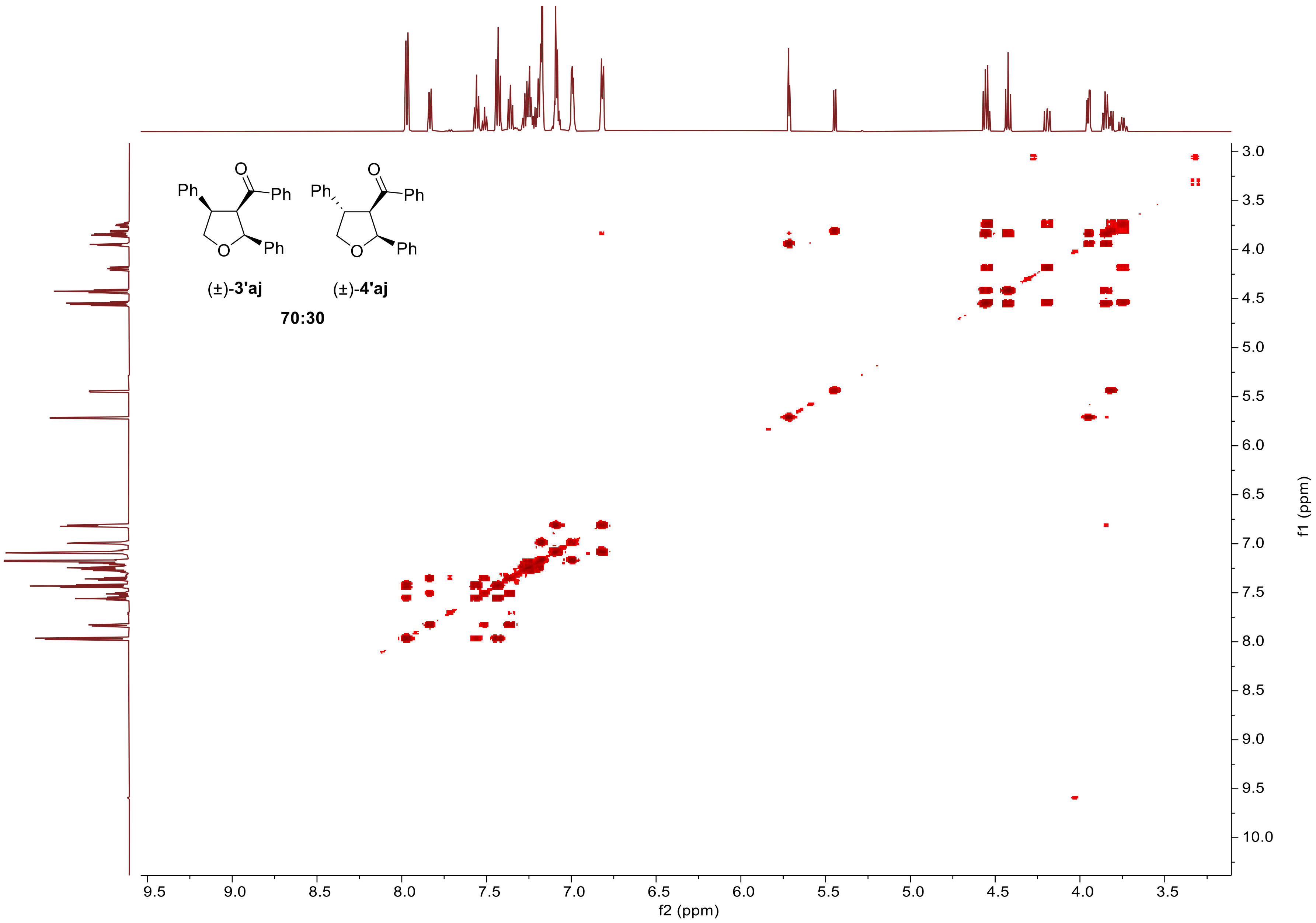

Figure S58. COSY NMR Spectrum of (±)-**3'aj** and (±)-**4'aj** ($CDCl_3$).

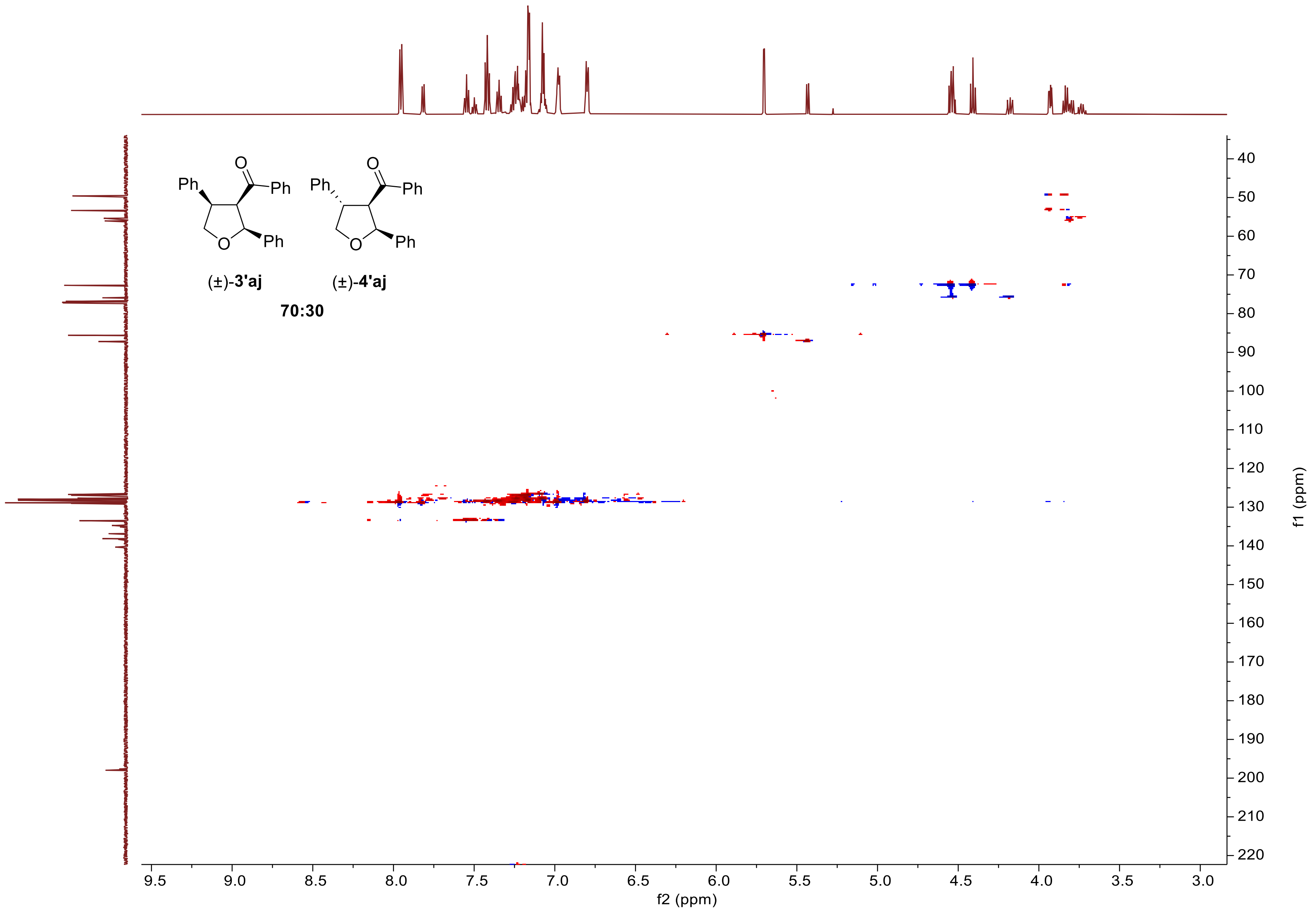

Figure S59. HSQC NMR Spectrum of (±)-**3'aj** and (±)-**4'aj** ($CDCl_3$).

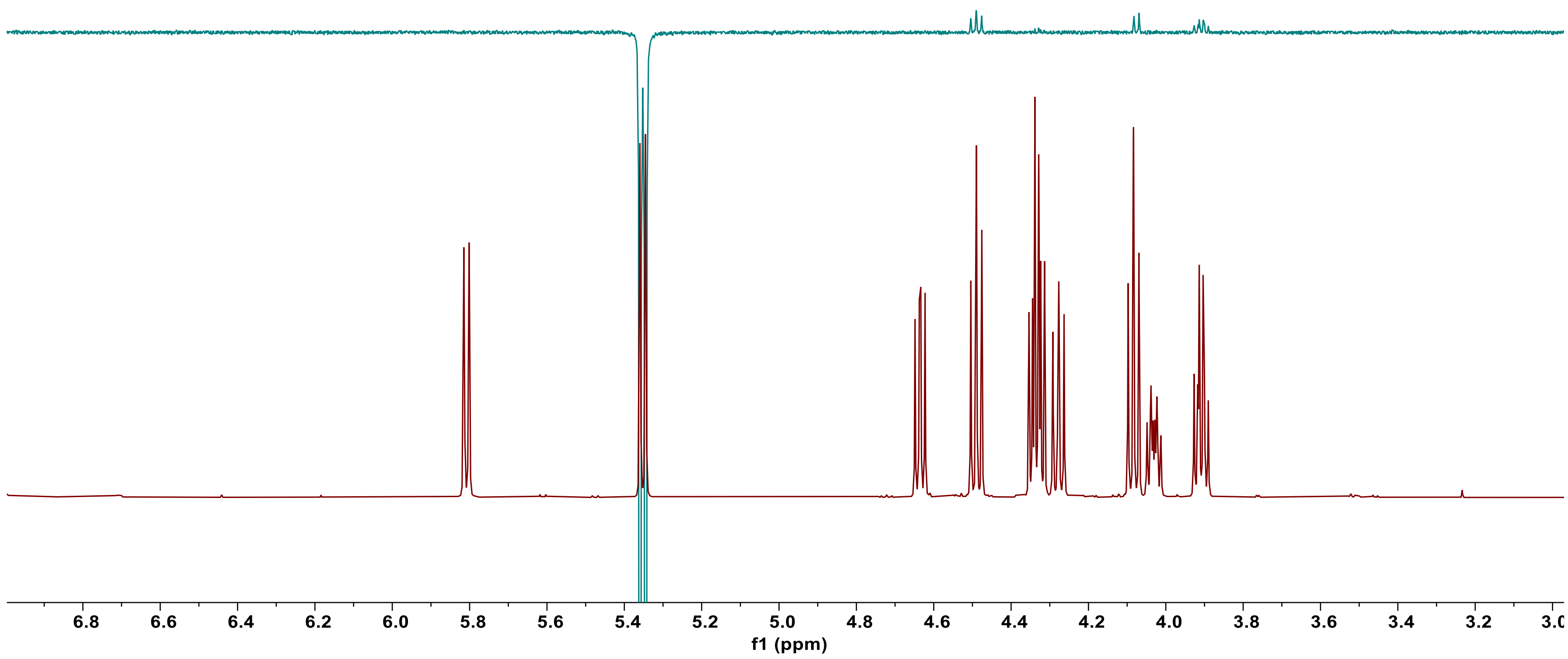

Figure S60. NOE-DIFF spectrum of the (±)-**3aj** and (±)-**4aj** ($CDCl_3$); increase in H-3, H-4 and H-5b signals after irradiation of the C***H*** (H-2) signal at 5.35 ppm.

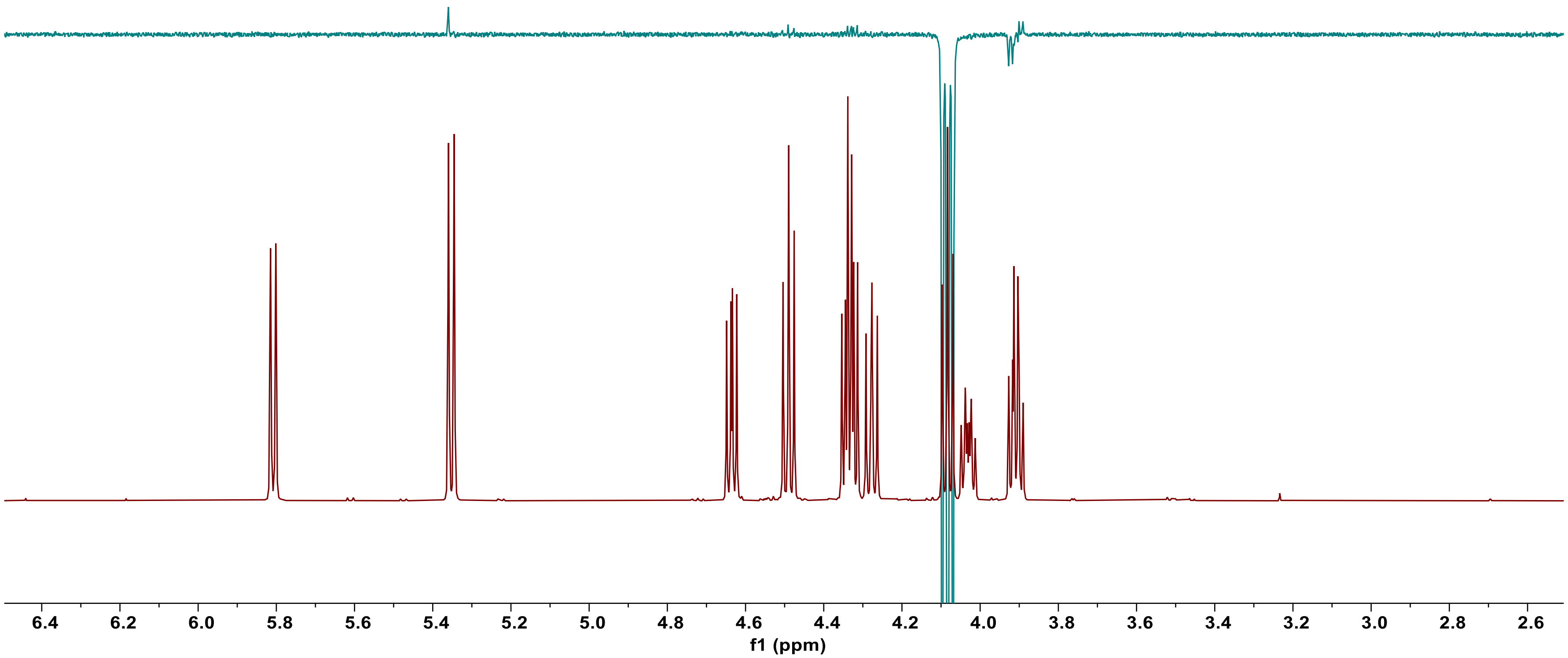

Figure S61. NOE-DIFF spectrum of the (±)-**3aj** and (±)-**4aj** ($CDCl_3$); increase in H-2 and H-4 signals after irradiation of the C***H*** (H-3) signal at 4.08 ppm.

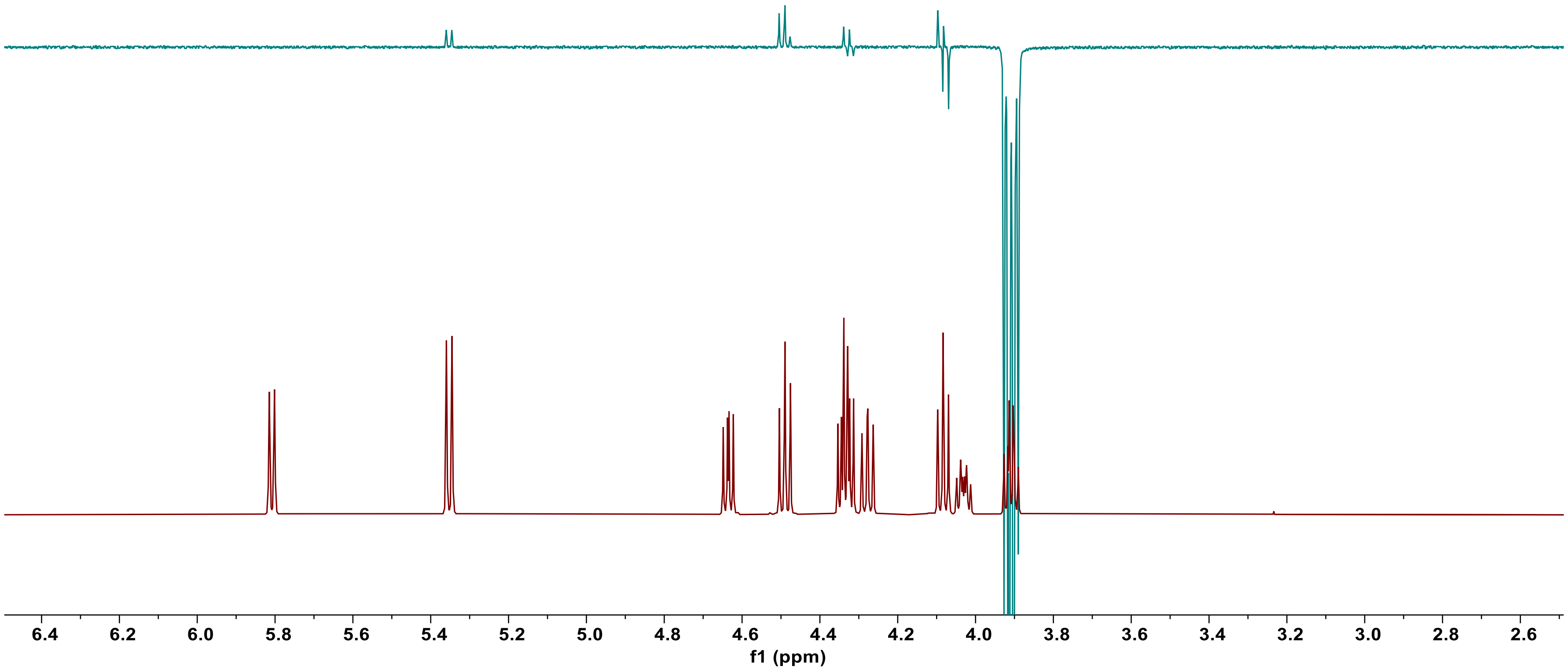

Figure S62. NOE-DIFF spectrum of the (±)-**3aj** and (±)-**4aj** ($CDCl_3$); increase in H-2, H-3, H-5a and H-5b signals after irradiation of the C***H*** (H-4) signal at 3.91 ppm.

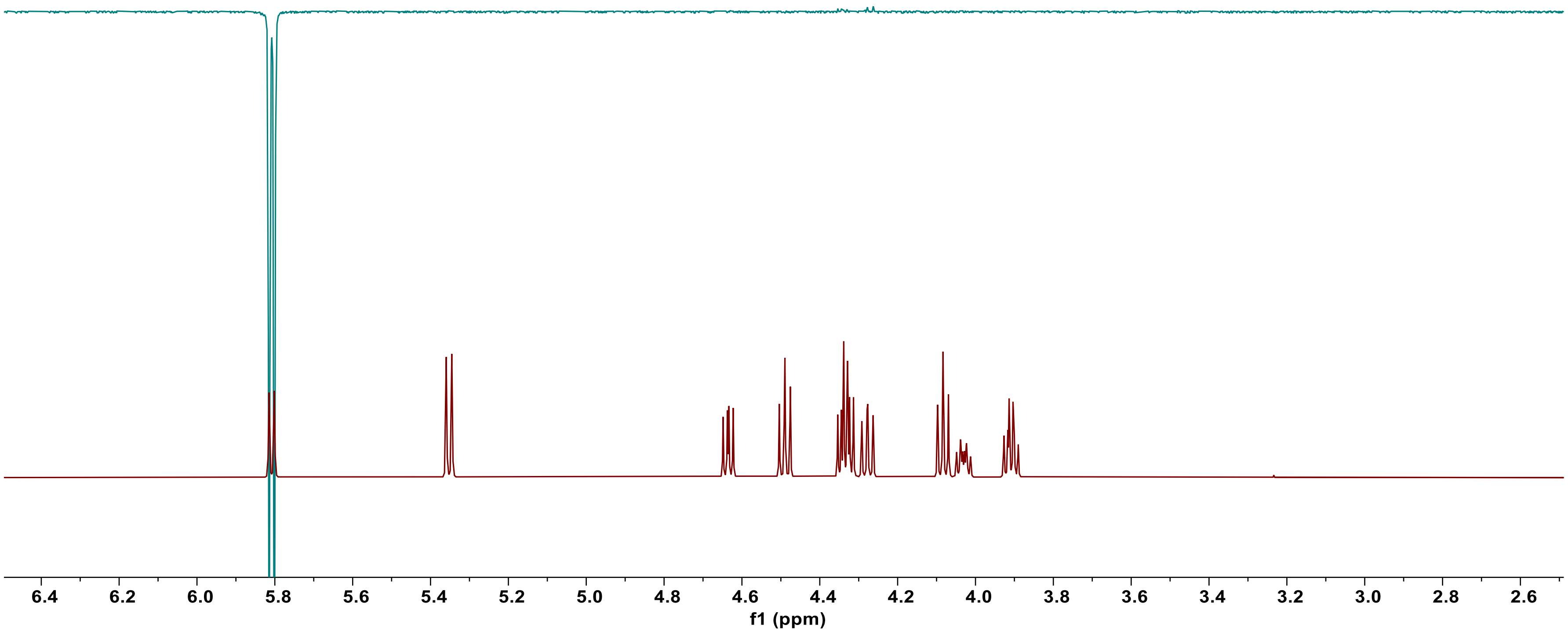

Figure S63. NOE-DIFF spectrum of the (±)-**3aj** and (±)-**4aj** ($CDCl_3$); no increase in signals after irradiation of the C***H*** (H-2) signal at 5.81 ppm.

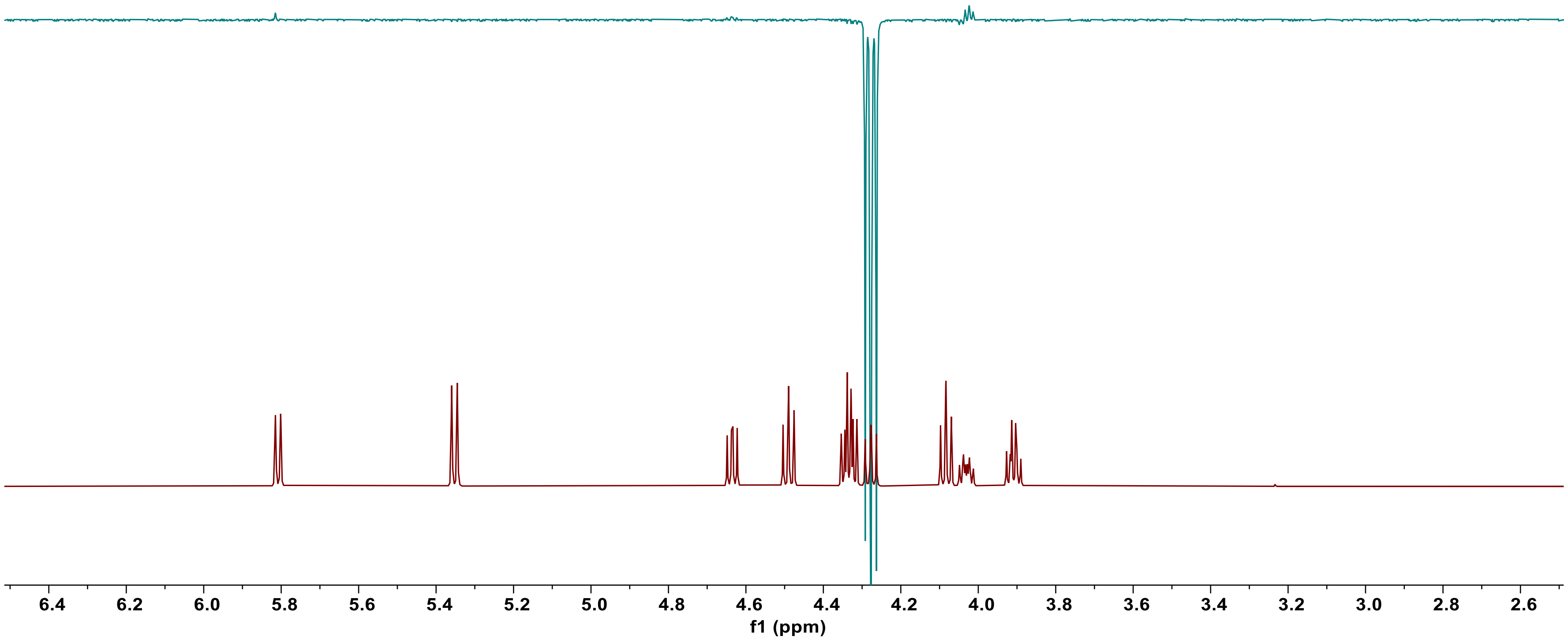

Figure S64. NOE-DIFF spectrum of the (±)-**3aj** and (±)-**4aj** ($CDCl_3$); increase in H-4 signal after irradiation of the C***H*** (H-3) signal at 4.28 ppm.

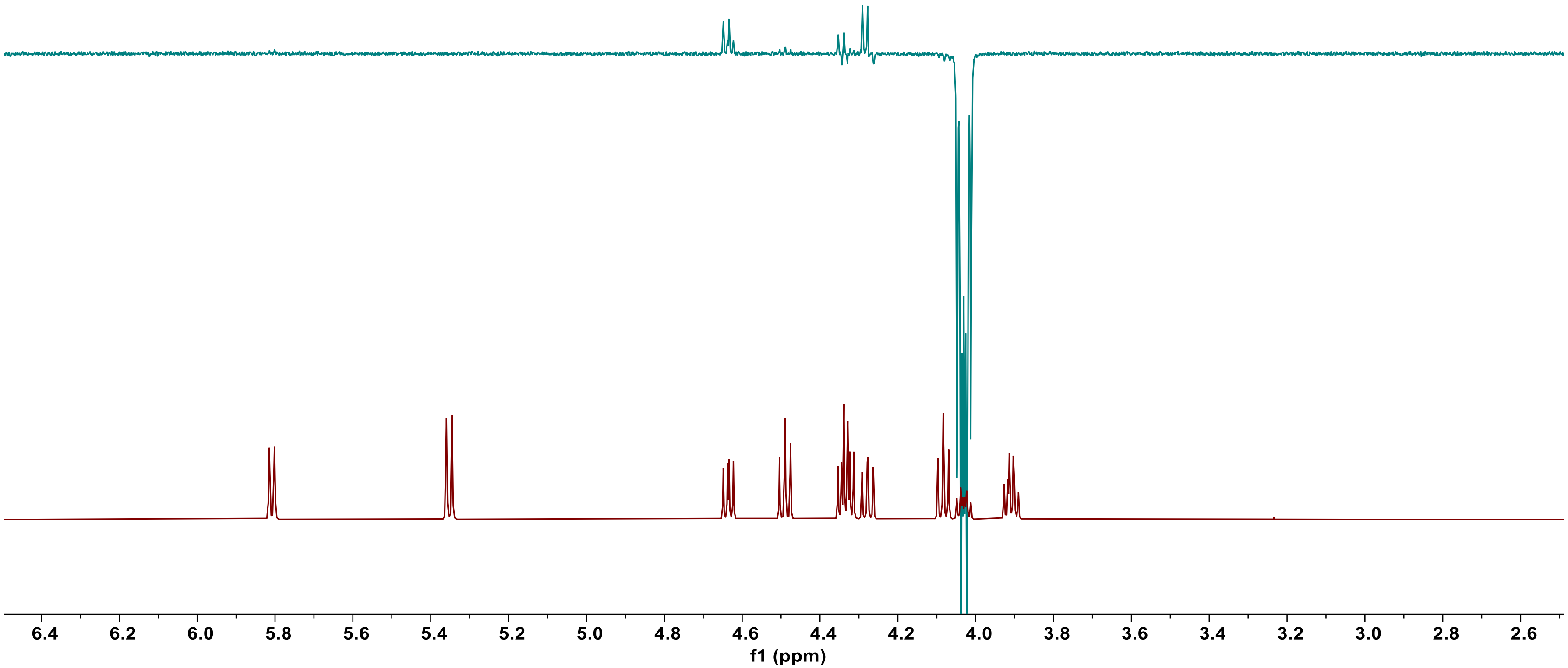

Figure S65. NOE-DIFF spectrum of the (±)-**3aj** and (±)-**4aj** ($CDCl_3$); increase in H-3, H-5a and H-5b signals after irradiation of the C***H*** (H-4) signal at 4.03 ppm.

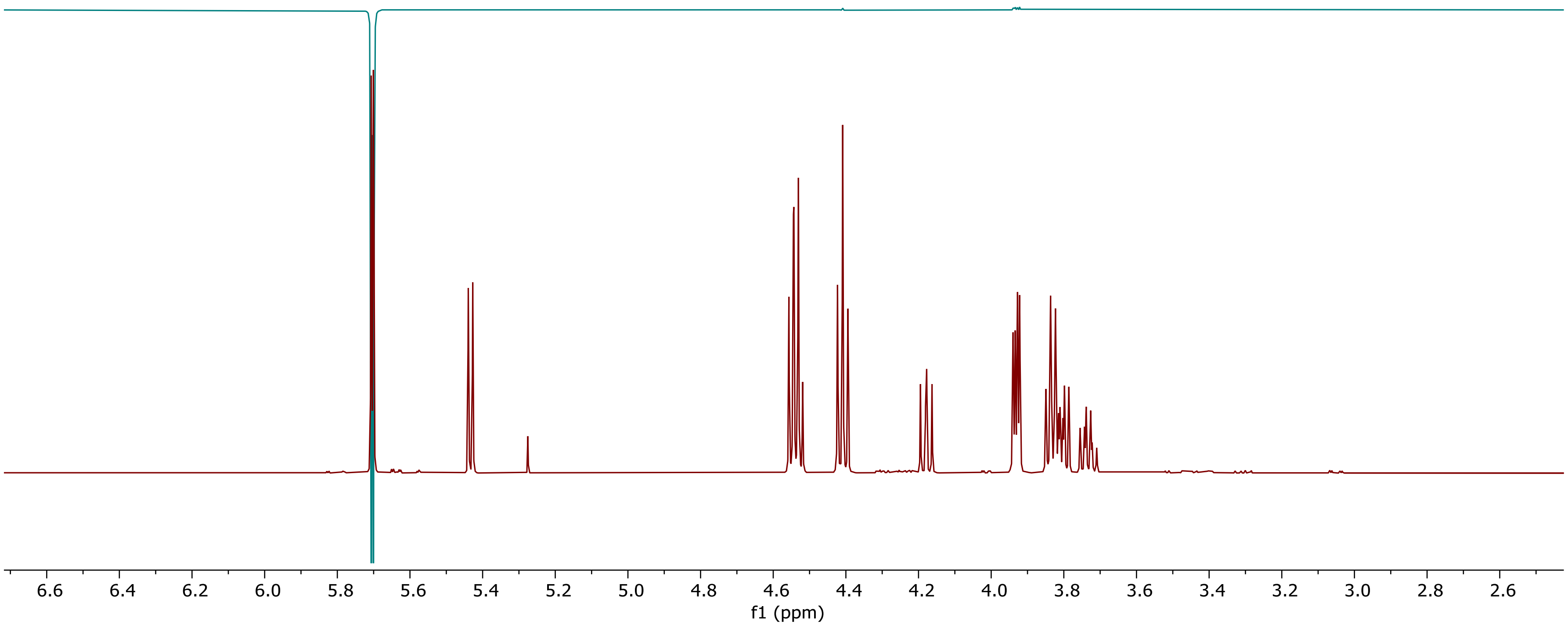

Figure S66. NOE-DIFF spectrum of the (±)-**3'aj** and (±)-**4'aj** ($CDCl_3$); no increase in signals after irradiation of the C***H*** (H-2) signal at 5.70 ppm.

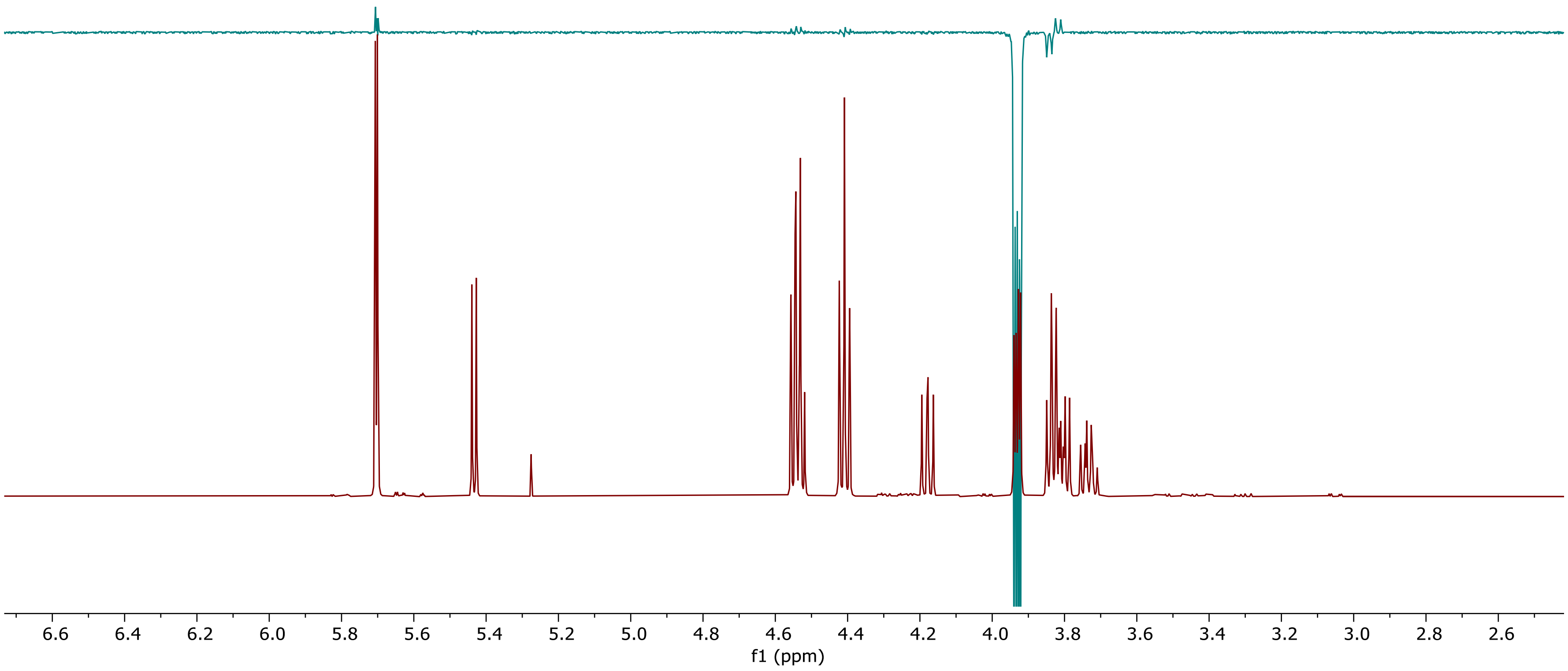

Figure S67. NOE-DIFF spectrum of the (±)-**3'aj** and (±)-**4'aj** ($CDCl_3$); increase in H-2, H-4, H-5a and H-5b signals after irradiation of the C***H*** (H-3) signal at 3.93 ppm.

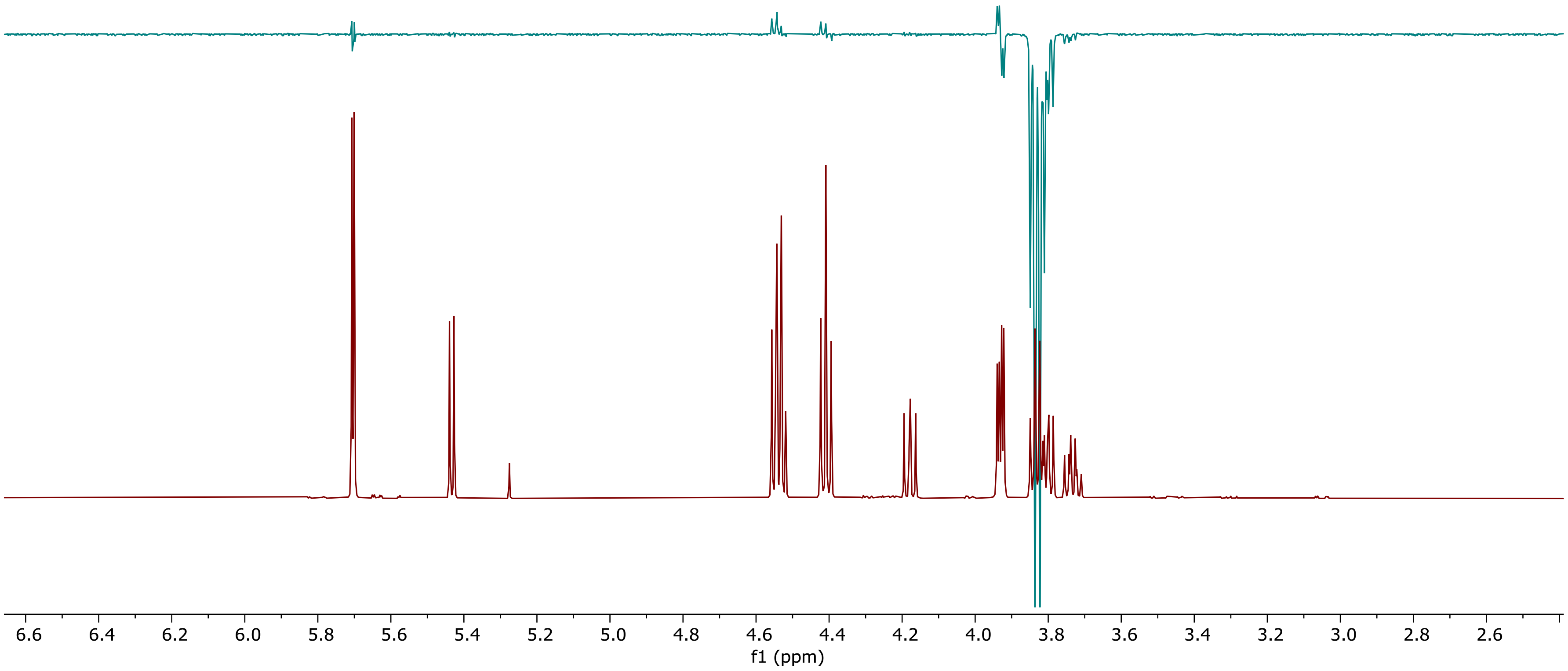

Figure S68. NOE-DIFF spectrum of the (±)-**3'aj** and (±)-**4'aj** ($CDCl_3$); increase in H-2, H-3, H-5a and H-5b signals after irradiation of the C***H*** (H-4) signal at 3.83 ppm.

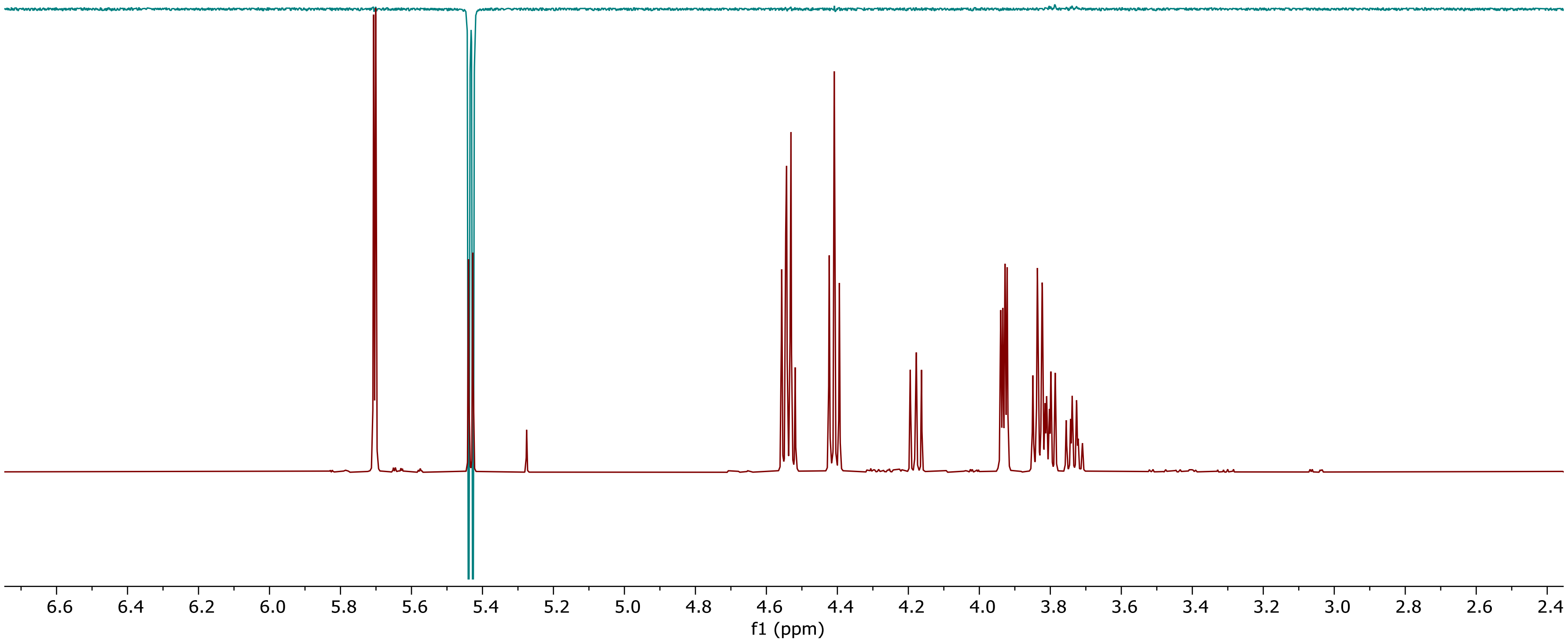

Figure S69. NOE-DIFF spectrum of the (±)-**3'aj** and (±)-**4'aj** ($CDCl_3$); no increase in signals after irradiation of the C***H*** (H-2) signal at 5.43 ppm.

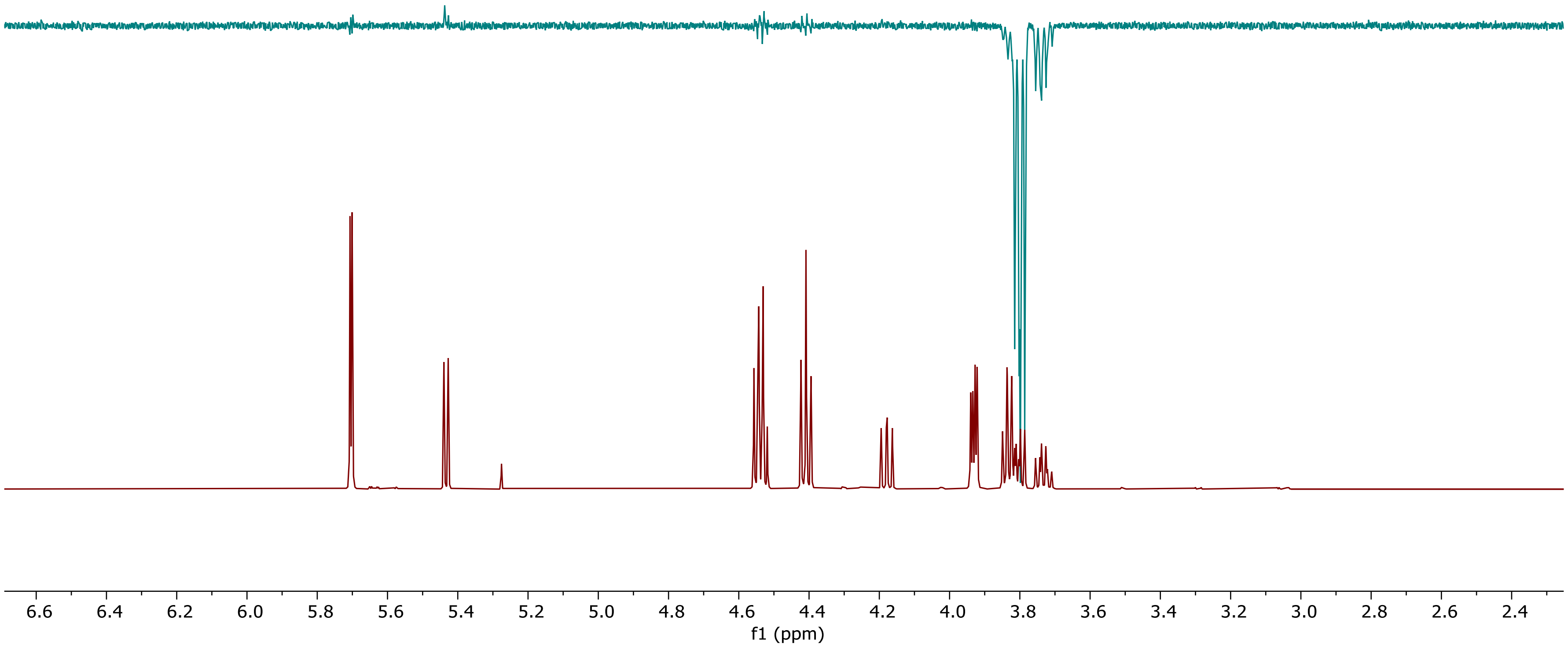

Figure S70. NOE-DIFF spectrum of the (±)-**3'aj** and (±)-**4'aj** ($CDCl_3$); increase in H-2 and H-5a signals after irradiation of the C***H*** (H-3) signal at 3.80 ppm.

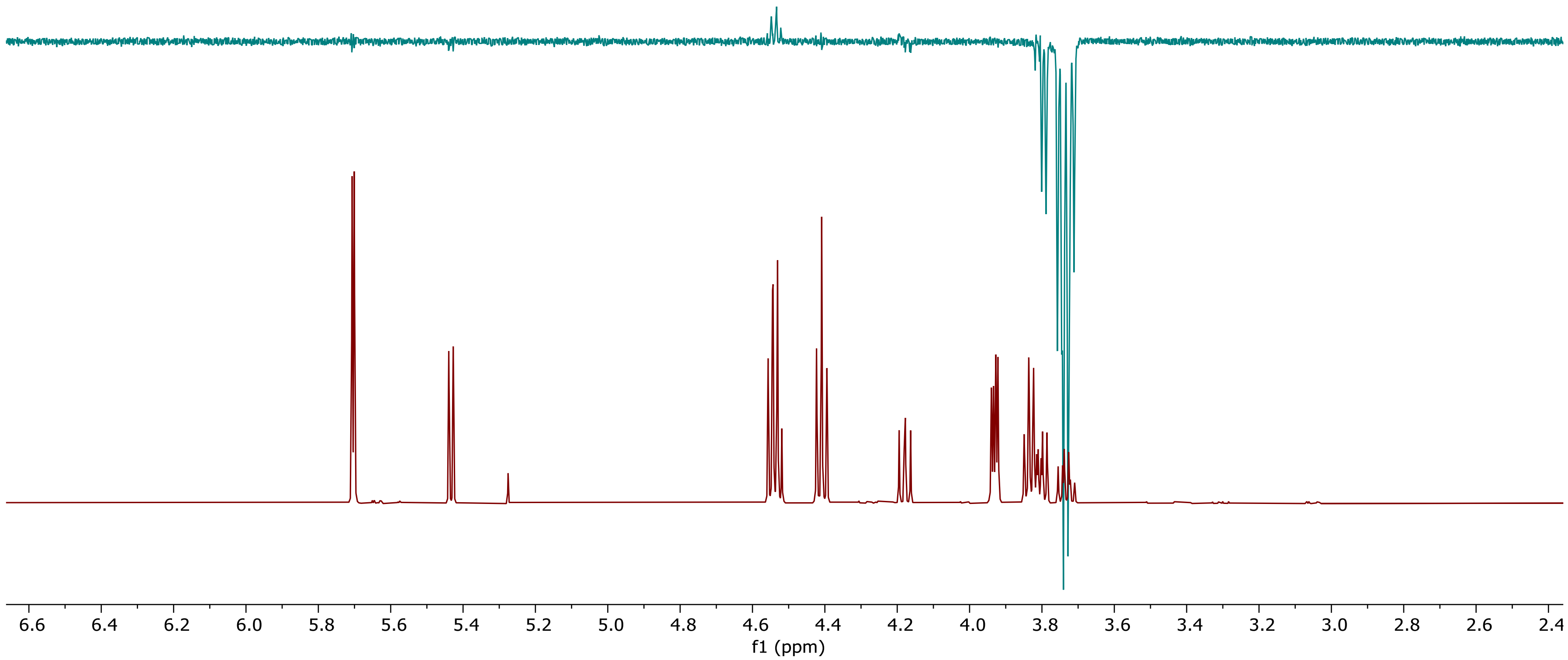

Figure S71. NOE-DIFF spectrum of the (±)-**3'aj** and (±)-**4'aj** ($CDCl_3$); increase in H-5a signal after irradiation of the C***H*** (H-4) signal at 3.73 ppm.

7. HPLC Chromatograms

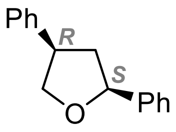

(2*S*,4*R*)-**3aa**

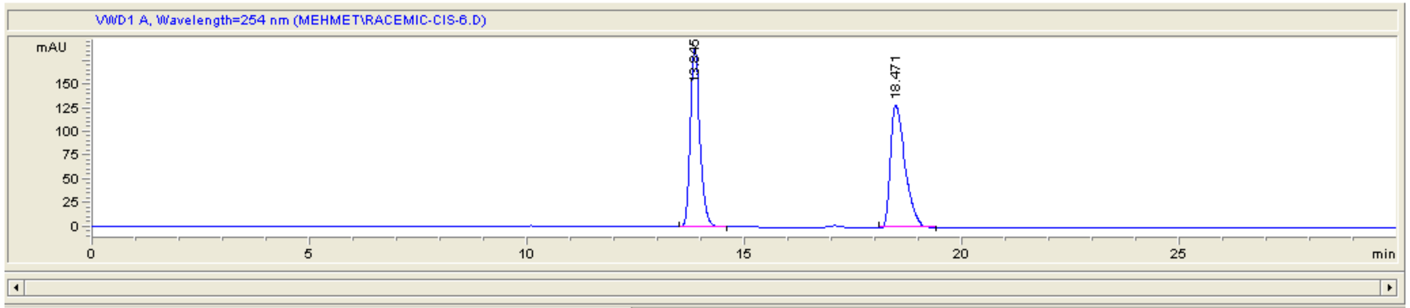

File Information

LC-File	RACEMIC-CIS-6.D
File Path	C:\CHEM32\1\DATA\MEHMET\
Date	09-Feb-11, 12:40:45
Sample	racemic-cis-6
Sample Info	racemic-cis (2,4-diphenylfurane)

#	Time	Area	Height	Width	Area%	Symmetry
1	13.845	2948.1	188.9	0.2403	49.963	0.696
2	18.471	2952.5	129	0.3471	50.037	0.488

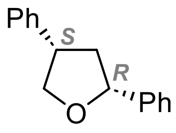

(2*R*,4*S*)-**3aa**

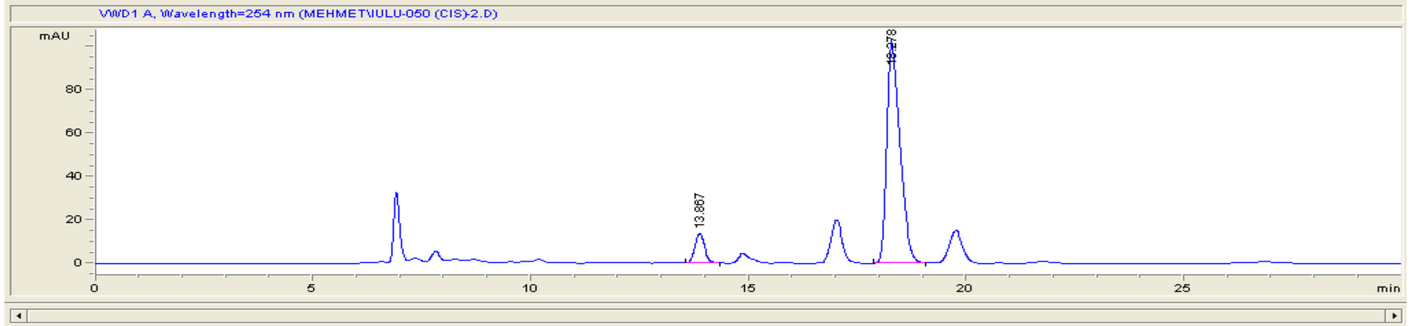

File Information

LC-File	IULU-050 (CIS)-2.D
File Path	C:\CHEM32\1\DATA\MEHMET\
Date	02-Feb-11, 13:10:20
Sample	Iulu-050 (cis)-2
Sample Info	reaction mix. Iulu-050 (cis)

#	Time	Area	Height	Width	Area%	Symmetry
1	13.867	201.7	13.5	0.2311	8.443	0.842
2	18.278	2187.6	102.8	0.3255	91.557	0.57

HPLC: Daicel CHIRALCEL OD-H (4.60 mm ID x 250 mm column length); *n*-hexane/*i*-PrOH (95:5), 0.5 mL/min; 254 nm (UV/Vis); t_R = 13.8 min ((2*R*,4*S*)-**3aa**), t_R = 18.3 min ((2*S*,4*R*)-**3aa**)

Figure S72. HPLC Chromatograms of (±)-**3aa** and (2*S*,4*R*)-**3aa.**

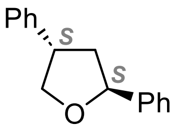

(2*S*,4*S*)-**4aa**

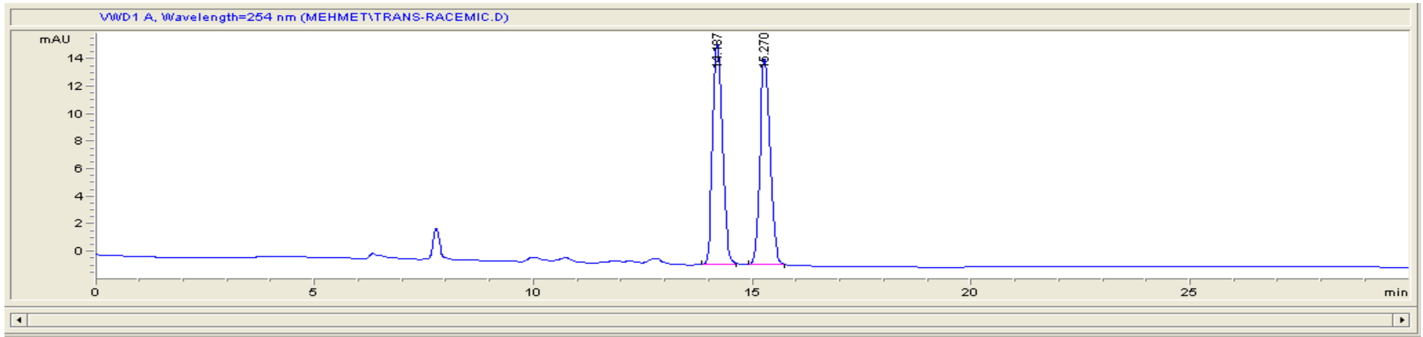

File Information

LC-File	TRANS-RACEMIC.D
File Path	C:\CHEM32\1\DATA\MEHMET\
Date	02-Feb-11, 09:43:58
Sample	TRANS-RACEMIC.D
Sample Info	trans-racemic (2,4-diphenylfurane)

#	Time	Area	Height	Width	Area%	Symmetry
1	14.187	244.6	16.1	0.2373	50.037	0.856
2	15.27	244.2	15	0.254	49.963	0.862

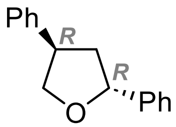

(2*R*,4*R*)-**4aa**

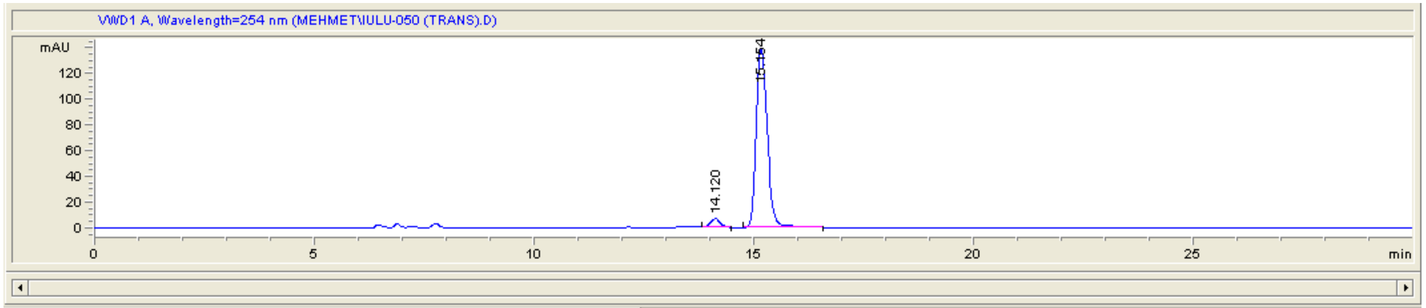

File Information

LC-File	IULU-050 (TRANS).D
File Path	C:\CHEM32\1\DATA\MEHMET\
Date	02-Feb-11, 10:23:01
Sample	Iulu-050 (trans)
Sample Info	reaction mix. Iulu-050 (trans)

#	Time	Area	Height	Width	Area%	Symmetry
1	14.12	107.2	6.9	0.2382	4.217	0.955
2	15.154	2434	139.4	0.2677	95.783	0.677

HPLC: Daicel CHIRALCEL OD-H (4.60 mm ID x 250 mm column length); *n*-hexane/*i*-PrOH (95:5), 0.5 mL/min; 254 nm (UV/Vis); t_R = 14.1 min ((2*R*,4*R*)-**4aa**), t_R = 15.1 min ((2*S*,4*S*)-**4aa**)

Figure S73. HPLC Chromatograms of (±)-**4aa** and (2*S*,4*S*)-**4aa.**

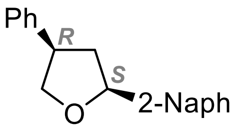

(2*S*,4*R*)-**3ab**

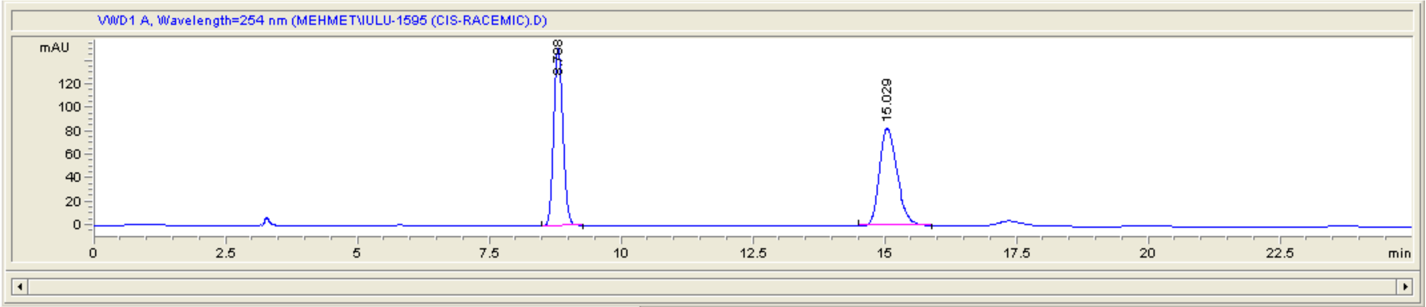

File Information

LC-File	IULU-1595 (CIS-RACEMIC).D
File Path	C:\CHEM32\1\DATA\MEHMET\
Date	14-Feb-12, 10:52:14
Sample	lulu-1595 (cis-racemic)
Sample Info	lulu-159 (cis-racemic-ODH)

#	Time	Area	Height	Width	Area%	Symmetry
1	8.788	1780.2	151.7	0.182	48.359	0.853
2	15.029	1901	83.1	0.3513	51.641	0.706

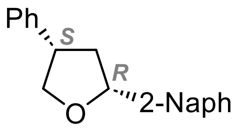

(2*R*,4*S*)-**3ab**

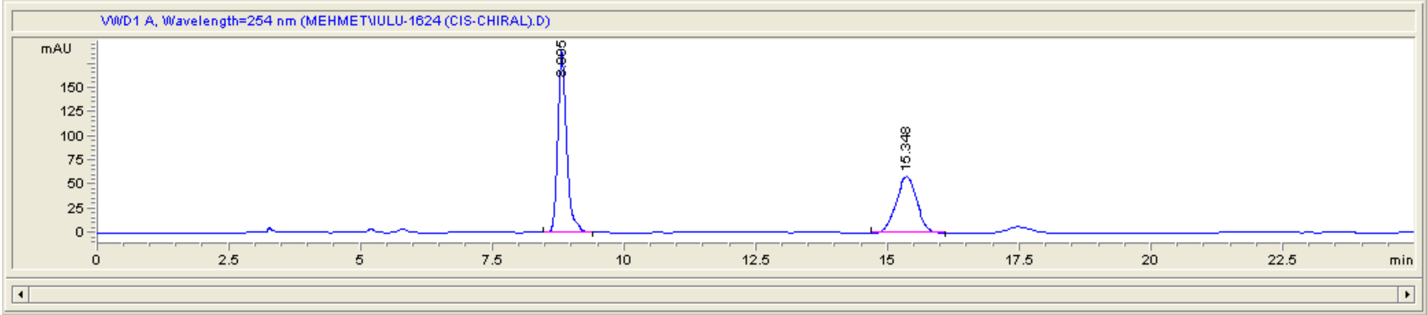

File Information

LC-File	IULU-1624 (CIS-CHIRAL).D
File Path	C:\CHEM32\1\DATA\MEHMET\
Date	14-Feb-12, 11:25:52
Sample	lulu-1624 (cis-chiral)
Sample Info	lulu-162 (cis-chiral-ODH)

#	Time	Area	Height	Width	Area%	Symmetry
1	8.805	2322.6	189.6	0.1881	59.345	0.775
2	15.348	1591.1	58.1	0.4227	40.655	1.014

HPLC: Daicel CHIRALCEL OD-H (4.60 mm ID x 250 mm column length); *n*-hexane/*i*-PrOH (95:5), 1.0 mL/min; 254 nm (UV/Vis); t_R = 8.8 min ((2*S*,4*R*)-**3ab**), t_R = 15.3 min ((2*R*,4*S*)-**3ab**)

Figure S74. HPLC Chromatograms of (±)-**3ab** and (2*S*,4*R*)-**3ab.**

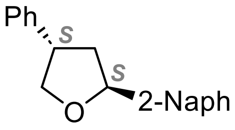

(2*S*,4*S*)-**4ab**

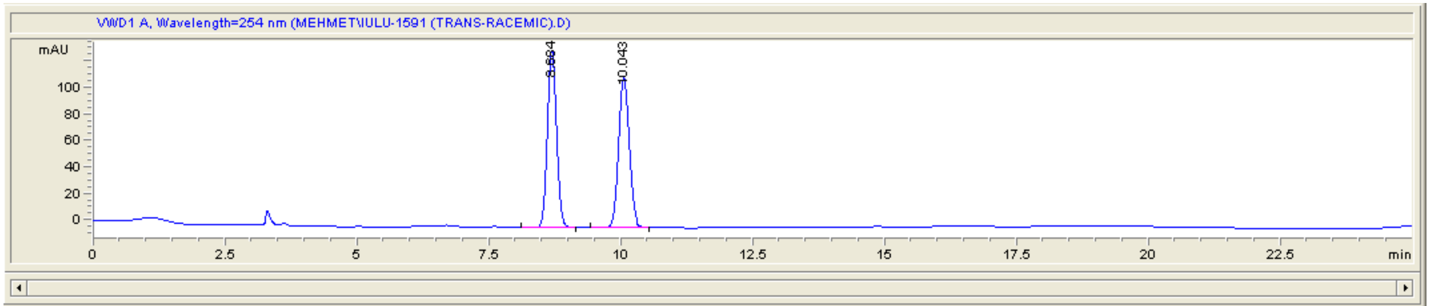

File Information

LC-File	IULU-1591 (TRANS-RACEMIC).D
File Path	C:\CHEM32\1\DATA\MEHMET\
Date	09-Feb-12, 08:57:28
Sample	lulu-1591 (trans-racemic)
Sample Info	lulu-159 (trans-racemic-ODH)

#	Time	Area	Height	Width	Area%	Symmetry
1	8.684	1572.3	134	0.1834	50.236	0.911
2	10.043	1557.5	113.3	0.2143	49.764	0.892

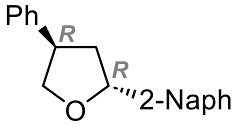

(2*R*,4*R*)-**4ab**

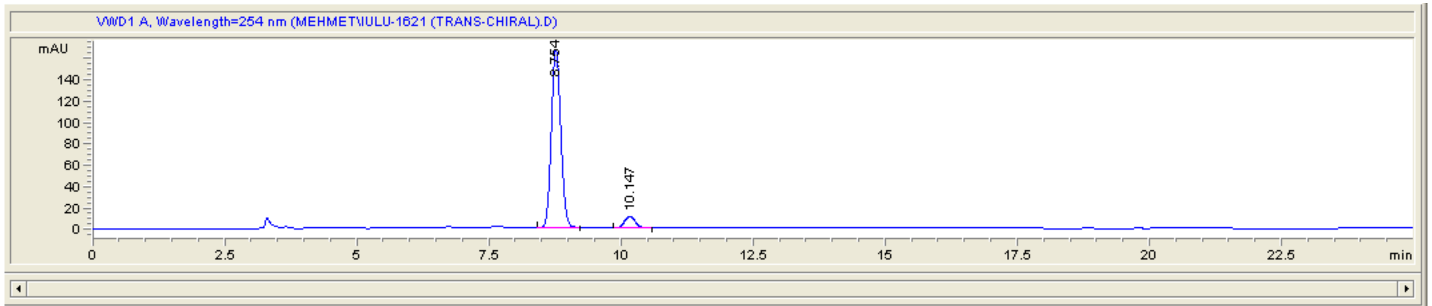

File Information

LC-File	IULU-1621 (TRANS-CHIRAL).D
File Path	C:\CHEM32\1\DATA\MEHMET\
Date	09-Feb-12, 09:43:03
Sample	lulu-1621 (trans-chiral)
Sample Info	lulu-162 (trans-chiral-ODH)

#	Time	Area	Height	Width	Area%	Symmetry
1	8.754	2009.9	167.1	0.1869	93.432	0.869
2	10.147	141.3	10.5	0.2116	6.568	0.973

HPLC: Daicel CHIRALCEL OD-H (4.60 mm ID x 250 mm column length); *n*-hexane/*i*-PrOH (95:5), 1.0 mL/min; 254 nm (UV/Vis); t_R = 8.7 min ((2*S*,4*S*)-**4ab**), t_R = 10.1 min ((2*R*,4*R*)-**4ab**)

Figure S75. HPLC Chromatograms of (±)-**4ab** and (2*S*,4*S*)-**4ab**.

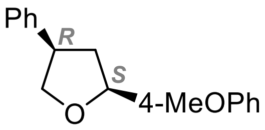

(2*S*,4*R*)-**3ac**

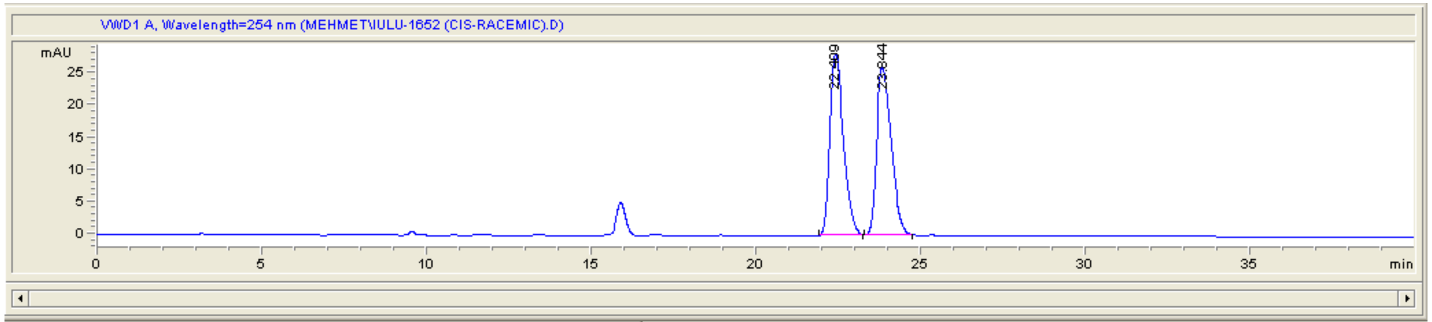

File Information

LC-File	IULU-1652 (CIS-RACEMIC).D
File Path	C:\CHEM32\1\DATA\MEHMET\
Date	27-Feb-12, 09:58:39
Sample	lulu-1652(cis-racemic)
Sample Info	lulu-165 (cis-racemic-ODH)

#	Time	Area	Height	Width	Area%	Symmetry
1	22.409	809.3	28.4	0.4422	50.196	0.618
2	23.844	803	26.1	0.4784	49.804	0.652

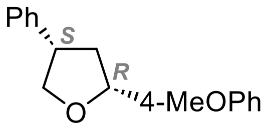

(2*R*,4*S*)-**3ac**

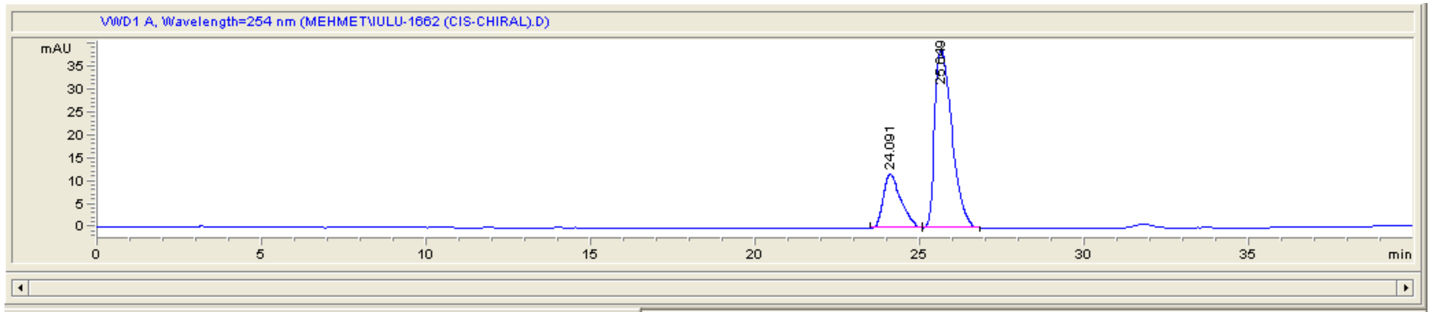

File Information

LC-File	IULU-1662 (CIS-CHIRAL).D
File Path	C:\CHEM32\1\DATA\MEHMET\
Date	27-Feb-12, 10:53:23
Sample	lulu-1662 (cis-chiral)
Sample Info	lulu-166 (cis-chiral-ODH)

#	Time	Area	Height	Width	Area%	Symmetry
1	24.091	429.3	11.6	0.5406	23.342	0.552
2	25.649	1409.8	39	0.5556	76.658	0.549

HPLC: Daicel CHIRALCEL OD-H (4.60 mm ID x 250 mm column length); *n*-hexane/*i*-PrOH (99:1), 1.0 mL/min; 254 nm (UV/Vis); t_R = 24.1 min ((2*R*,4*S*)-**3ac**), t_R = 25.7 min ((2*S*,4*R*)-**3ac**)

Figure S76. HPLC Chromatograms of (±)-**3ac** and (2*S*,4*R*)-**3ac.**

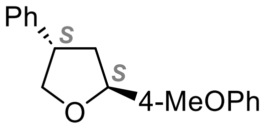

(2*S*,4*S*)-**4ac**

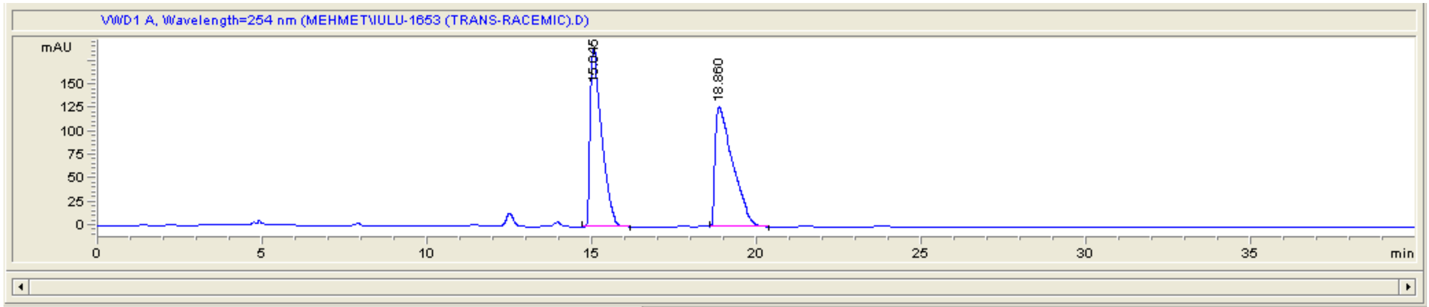

File Information

LC-File	IULU-1653 (TRANS-RACEMIC).D
File Path	C:\CHEM32\1\DATA\MEHMET\
Date	28-Feb-12, 08:41:31
Sample	lulu-1653 (trans-racemic)
Sample Info	lulu-165 (trans-racemic-ADH)

#	Time	Area	Height	Width	Area%	Symmetry
1	15.045	4716.7	190.4	0.3721	50.148	0.368
2	18.86	4688.8	127.5	0.531	49.852	0.246

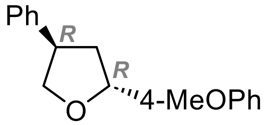

(2*R*,4*R*)-**4ac**

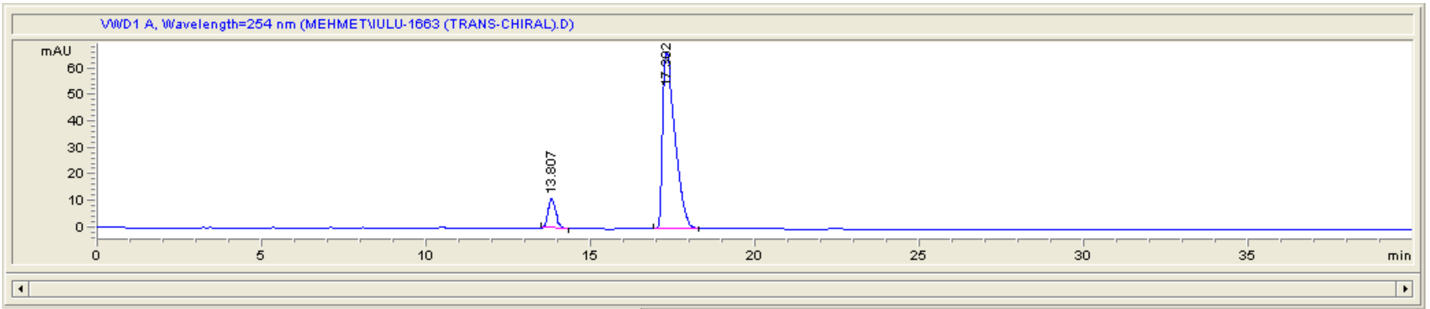

File Information

LC-File	IULU-1663 (TRANS-CHIRAL).D
File Path	C:\CHEM32\1\DATA\MEHMET\
Date	28-Feb-12, 09:23:58
Sample	lulu-1663 (trans-chiral)
Sample Info	lulu-166 (trans-chiral-ADH)

#	Time	Area	Height	Width	Area%	Symmetry
1	13.807	179.6	11	0.2532	9.283	0.741
2	17.302	1754.8	66.9	0.4035	90.717	0.395

HPLC: Daicel CHIRALPAK AD-H (4.60 mm ID x 250 mm column length); *n*-hexane/*i*-PrOH (99:1), 1.0 mL/min; 254 nm (UV/Vis); t_R = 13.8 min ((2*R*,4*R*)-**4ac**), t_R = 17.3 min ((2*S*,4*S*)-**4ac**)

Figure S77. HPLC Chromatograms of (±)-**4ac** and (2*S*,4*S*)-**4ac.**

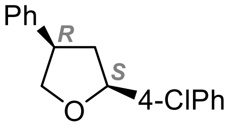

(2*S*,4*R*)-**3ad**

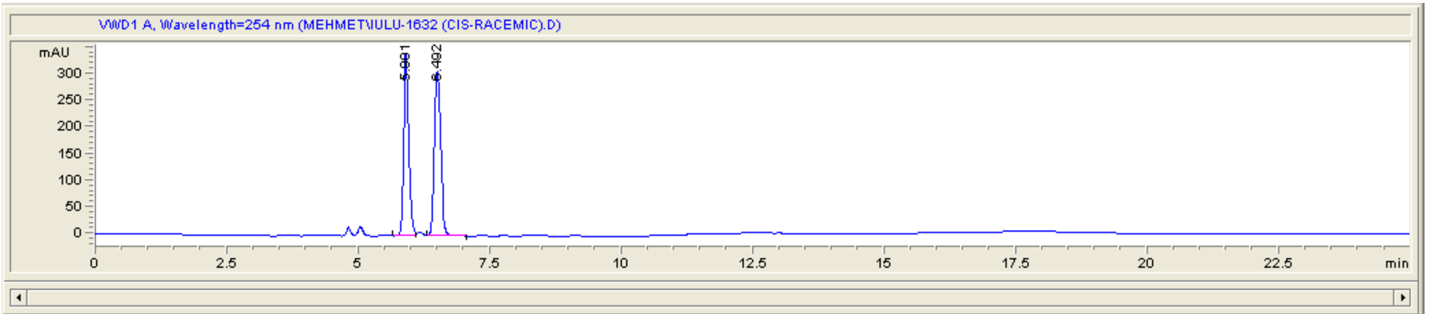

File Information

LC-File	IULU-1632 (CIS-RACEMIC).D
File Path	C:\CHEM32\1\DATA\MEHMET\
Date	06-Mar-12, 18:59:24
Sample	lulu-1632 (cis-racemic)
Sample Info	lulu-163 (cis-racemic-ODH)

#	Time	Area	Height	Width	Area%	Symmetry
1	5.901	2549.1	343.3	0.1143	49.949	0.783
2	6.492	2554.3	308.5	0.1272	50.051	0.744

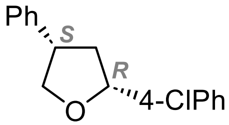

(2*R*,4*S*)-**3ad**

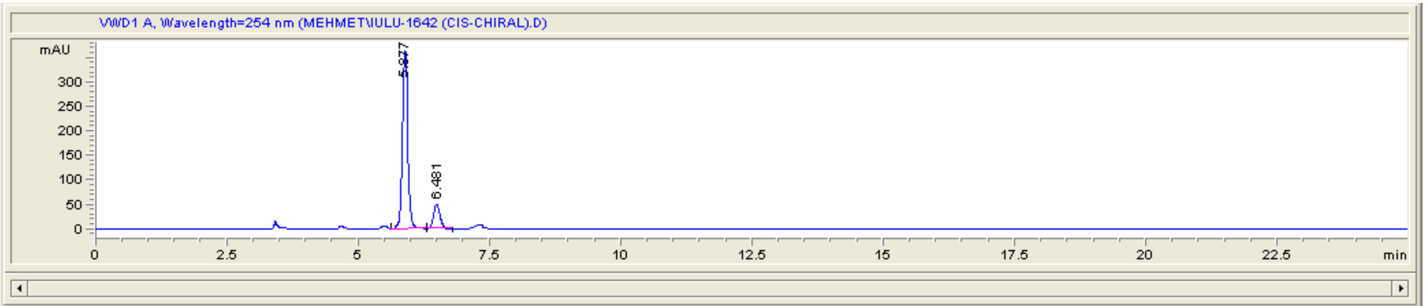

File Information

LC-File	IULU-1642 (CIS-CHIRAL).D
File Path	C:\CHEM32\1\DATA\MEHMET\
Date	06-Mar-12, 19:31:02
Sample	lulu-1642 (cis-chiral)
Sample Info	lulu-164 (cis-chiral-ODH)

#	Time	Area	Height	Width	Area%	Symmetry
1	5.877	2765.5	364.8	0.1161	87.050	0.797
2	6.481	411.4	50.6	0.1269	12.950	0.87

HPLC: Daicel CHIRALPAK OD-H (4.60 mm ID x 250 mm column length); *n*-hexane/*i*-PrOH (95:5), 1.0 mL/min; 254 nm (UV/Vis); t_R = 5.9 min ((2*S*,4*R*)-**3ad**), t_R = 6.5 min ((2*R*,4*S*)-**3ad**)

Figure S78. HPLC Chromatograms of (±)-**3ad** and (2*S*,4*R*)-**3ad**.

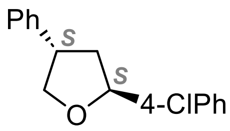

(2*S*,4*S*)-**4ad**

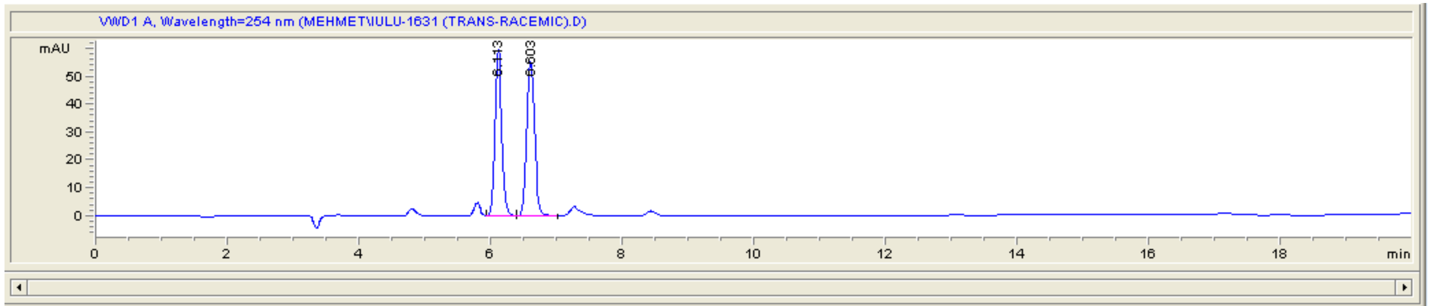

File Information

LC-File	IULU-1631 (TRANS-RACEMIC).D
File Path	C:\CHEM32\1\DATA\MEHMET\
Date	18-Feb-12, 16:38:34
Sample	lulu-1631 (trans-racemic)
Sample Info	lulu-163 (trans-racemic-ODH)

#	Time	Area	Height	Width	Area%	Symmetry
1	6.113	452.3	59.9	0.1171	49.772	0.844
2	6.603	456.4	55.2	0.1284	50.228	0.83

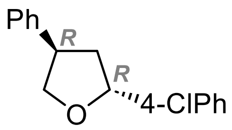

(2*R*,4*R*)-**4ad**

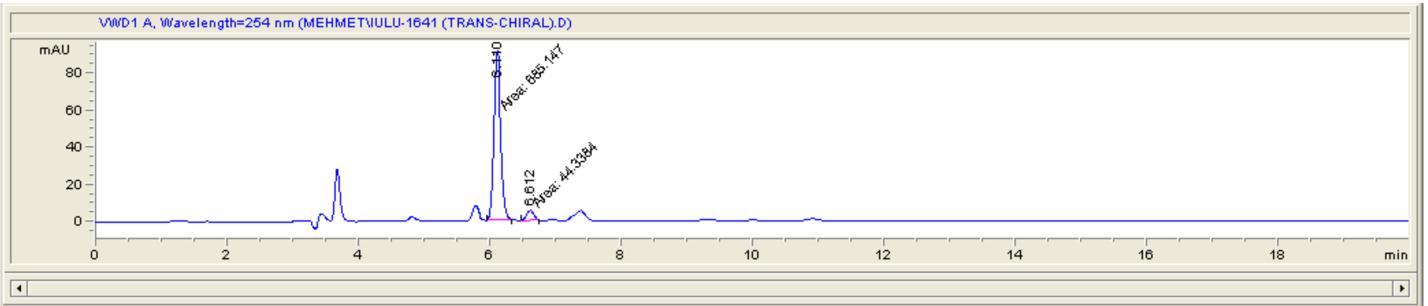

File Information

LC-File	IULU-1641 (TRANS-CHIRAL).D
File Path	C:\CHEM32\1\DATA\MEHMET\
Date	18-Feb-12, 17:00:34
Sample	lulu-1641 (trans-chiral)
Sample Info	lulu-164 (trans-chiral-ODH)

#	Time	Area	Height	Width	Area%	Symmetry
1	6.11	685.1	91.6	0.1247	93.922	0.846
2	6.612	44.3	5.6	0.1331	6.078	0.991

HPLC: Daicel CHIRALPAK OD-H (4.60 mm ID x 250 mm column length); *n*-hexane/*i*-PrOH (95:1), 1.0 mL/min; 254 nm (UV/Vis); t_R = 6.1 min ((2S,4S)-**4ad**), t_R = 6.6 min ((2*R*,4*R*)-**4ad**)

Figure S79. HPLC Chromatograms of (±)-**4ad** and (2*S*,4*S*)-**4ad.**

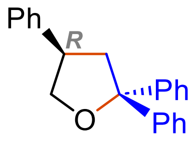

(*R*)-**3ah**

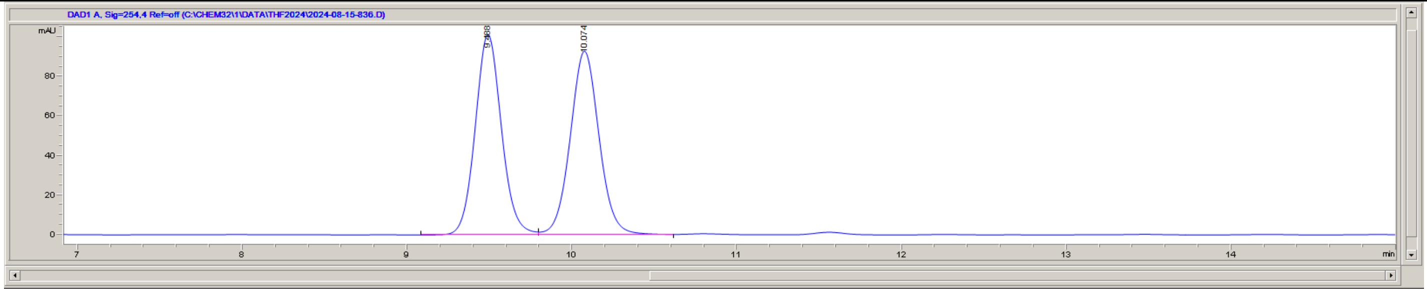

#	Time	Type	Area	Height	Width	Area%	Symmetry
1	9.488	BV	1115.8	101	0.1689	49.840	0.904
2	10.074	VB	1123	92.4	0.1858	50.160	0.94

File Information

LC-File	2024-08-15-836.D
File Path	C:\CHEM32\1\DATA\THF2024
Date	15-Aug-24, 12:23:06
Sample	rac-triPhTHF
Sample Info	rac-triPhTHF, AD-H, 95:5 Hexane/IPA, 0,5mL/min, 254nm, 15/8/20

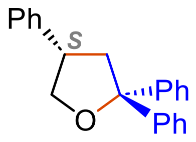

(*S*)-**3ah**

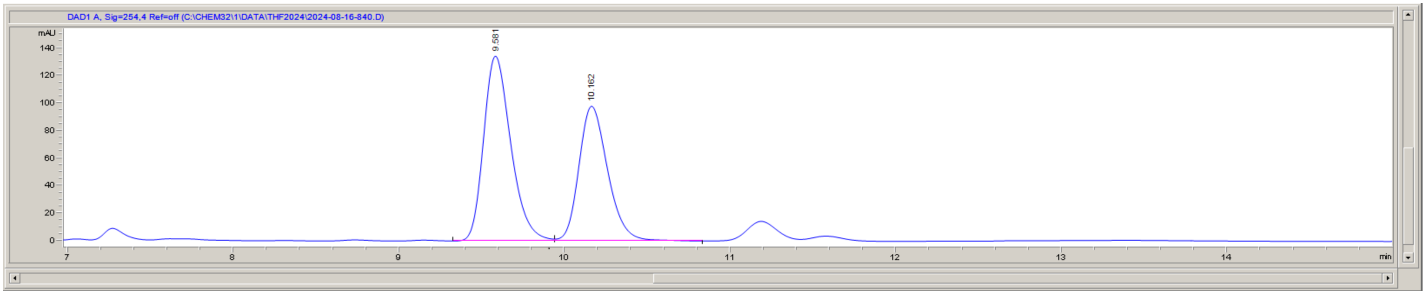

#	Time	Type	Area	Height	Width	Area%	Symmetry
1	9.581	VV	1542.3	134.9	0.1774	56.475	0.722
2	10.162	VB	1188.7	98.3	0.1851	43.525	0.745

File Information

LC-File	2024-08-16-840.D
File Path	C:\CHEM32\1\DATA\THF2024
Date	16-Aug-24, 16:10:28
Sample	rac-triPhTHF
Sample Info	chiral-triPhTHF, AD-H, 95:5 Hexane/IPA, 0,5mL/min, 254nm, 16/8/2

HPLC: Daicel CHIRALPAK AD-H (4.60 mm ID x 250 mm column length); *n*-hexane/*i*-PrOH (95:1), 0.5 mL/min; 254 nm (UV/Vis); t_R = 9.6 min ((4*R*)-**3ah**), t_R = 10.2 min ((4*S*)-**3ah**)

Figure S80. HPLC Chromatograms of (±)-**3ah** and (4*R*)-**3ah.**

Authorship Contribution Statement

Mehmet Ulutürk: Investigation, Validation. Mehmet Göllü: Investigation, Validation. Tahir Tilki: Supervision. Erkan Ertürk: Funding acquisition, Project administration, Investigation, Writing of original draft, Editing of the manuscript.
